# Feasibility trial of a transdiagnostic individual lifestyle evaluation and intervention (Lev-i) for health behavior change

**DOI:** 10.1371/journal.pone.0339500

**Published:** 2026-01-12

**Authors:** Douglas Sjöwall, Annika Brar, Anna Fladvad, Ulrika Långh, Tatja Hirvikoski

**Affiliations:** 1 Center of Neurodevelopmental Disorders at Karolinska Institutet (KIND), Department of Women’s and Children’s Health, Centre for Psychiatry Research, Karolinska Institutet & Region Stockholm, Stockholm, Sweden; 2 Habilitation and Health, Stockholm Health Care Services, Region Stockholm, Sweden; 3 Division of Psychology, Department of Clinical Neuroscience, Karolinska Institutet, Stockholm, Sweden; University of Modena and Reggio Emilia, ITALY

## Abstract

**Purpose:**

Individuals with disabilities face challenges in adopting and maintaining healthy behaviors. Existing interventions are often diagnosis-specific, profession-bound, and limited to single health behaviors, restricting a broader applicability. This study evaluated the feasibility and acceptability of Lev-individual (Lev-i), a transdiagnostic and interprofessional intervention designed to promote health behaviors among individuals with disabilities.

**Method:**

This feasibility study was conducted in an outpatient disability healthcare setting, involving 25 adult participants with long-term disabilities. Lev-i consists of three structured sessions focusing on goal setting, motivational strategies, and behavior change techniques that can be used across ten health domains. Data on treatment completion, acceptability and secondary outcomes was collected through self-reports for participants and professionals before, during, and after the intervention.

**Results:**

The treatment completion rate was 23 out of 25 (92%), with high treatment satisfaction (M = 3.61 out of maximum 4). Participants reported significant increases in treatment credibility and expectancy of change (6.93 pre, 8.27 post, *p* < .01), as well as high treatment satisfaction. Professionals rated the intervention as relevant and feasible, though many comments indicated a need for simplification of the study protocol and clearer intervention materials. No serious adverse events were reported. For secondary outcomes, goal attainment scores indicated that participants on average reached their goals. Also, there was a significant improvement in dietary habits (*p* < .05, *d* = 0.55), and a non-significant trend toward increased physical activity (*d* = 0.39).

**Conclusion:**

Findings suggest that Lev-i is a feasible and acceptable intervention for promoting health behaviors among individuals with disabilities. However, further refinements may enhance feasibility for both patients and professionals before conducting a randomized controlled trial. ClinicalTrials.gov (NCT05889936).

## Introduction

Individuals with enduring disabilities often face biopsychosocial barriers [1] in maintaining healthy behaviors which can contribute to somatic and mental health problems [2]. One barrier is the lack of feasible and effective interventions available to individuals with disabilities [3,4]. Most existing interventions focus on single health behaviors and are tailored to specific diagnoses or professional roles [5–7], limiting their applicability across various health behaviors, diverse disability groups and healthcare settings. There is a need for flexible, transdiagnostic, and interprofessional interventions that can support individuals in making sustainable changes across multiple health behaviors.

### Improving health behaviors remains a public health challenge

Non-communicable diseases are rising globally, causing significant human suffering and straining healthcare systems [8,9]. A third of the world’s disease burden is linked to inadequate physical activity and poor diet [10], which are also strongly associated with mental health issues, further increasing healthcare demands [11]. Improving health behaviors is therefore important for preventing both physical and mental illness, reducing premature mortality, and easing the burden on healthcare systems. This is especially important regarding people with disabilities, as having a disability is generally associated with poorer health behaviors [12–17] and increase in mortality [18,19].

Several interventions have been developed to successfully promote healthier lifestyles, primarily focusing on people without disabilities. For example, a 2023 systematic review and meta-analysis found that physical activity interventions led to a modest but significant increase in activity levels over a 24-month period, highlighting the potential for long-term behavior change [5]. Another systematic review examined dietary behavior change interventions and found several techniques to be effective for young adults to improve their diet [7]. Furthermore, a systematic review highlighted several effective behavioral support methods for smoking cessation [6]. Importantly, successful treatment components include motivational interviewing and cognitive behavior therapy-based techniques such as self-monitoring, goal setting, and structured follow-ups. However, fewer studies of interventions to improve health behaviors exist for individuals with disabilities [20,21] especially including real world clinical settings. Most programs are diagnosis-specific and resource-intensive. Few interventions integrate multiple health behaviors or are designed for scalability and implementation in clinical settings. Consequently, while individual interventions have been evaluated, few are implemented, and evidence-based approaches therefore often fail to reach individuals with disabilities [22,23]. Recent reviews therefore emphasize the need for theory-based, transdiagnostic approaches that can be adapted across disability groups and settings [24–26]. As an illustration of this problem, Sweden introduced national guidelines emphasizing the importance of prioritizing health behaviors, particularly in individuals with disabilities, as early as 2011. However, despite clear and compelling arguments for addressing these major public health problems, it is concluded in the newly updated guidelines, that the challenge of providing sufficient support for a healthier lifestyle, remains [27].

### What’s the problem?

Several critical factors contribute to the lack of implementation of health behavior interventions for individuals with disabilities. Identified barriers for implementation include lack of feasible and effective interventions for individuals with disabilities, not including relevant stakeholders, competing responsibilities and time constraints [28]. Another review, focusing on implementation of guidelines in primary care, identified political and institutional barriers, healthcare provider knowledge, time, patient attitudes, and guideline clarity [29]. Moreover, a possible barrier, especially within the disability context, is the paucity of co-creation approaches [23]. Our analyses, refined through iterative discussions with key stakeholders—including healthcare management, patient representatives from non-governmental organizations, and professionals—aligned closely with the conclusions of these reviews. All stakeholders emphasized the significance of addressing health behaviors. Patient representatives highlighted the need for psychological support and improved coordination among healthcare providers, while management prioritized an affordable and scalable intervention. Healthcare staff sought a structured, manualized approach that integrated well with their existing practices. Altogether, there is a clear need for an intervention that can disrupt the status quo by addressing the persistent gaps in implementing evidence-based interventions, particularly for individuals with disabilities. Since the implementation of health behavior promotion interventions is very limited for people with disabilities, it is crucial that we continue investigating various aspects of feasibility — including acceptability — to ensure that the interventions are appropriate for the target populations.

## What’s new?

Against this background, we question designing separate interventions for each health behavior, tailored to individual diagnostic groups, and further segmented by healthcare profession. The sheer scale of such an approach would make implementation unmanageable [30,31]. Instead, we crafted the Lev-individual (Lev-i) to be transdiagnostic, interprofessional and applicable across multiple health behaviors. In line with other healthcare disciplines [32], we thus adhere to the principles of common protocols for both diverse disability groups and health behaviors, while providing individualizations of goal setting and strategies.

### Aims

To evaluate the feasibility and support the continued development of the Lev-i intervention, we conducted a study assessing treatment completion, credibility, and satisfaction as primary outcomes in two settings involving individuals with enduring disabilities. Although not central to main aim focusing on feasibility, we also conducted secondary analysis of goal attainment, health behaviors, and quality of life. A rationale for these analyses was to obtain preliminary effect sizes to be used when planning a future randomized controlled trial.

## Methods

### Study setting and design

This was an open feasibility study done within three healthcare services: the habilitation services in Stockholm and Malmö as well as a municipal support service (LSS/SoL-hälsan) in Stockholm, Sweden. Habilitation services provide specialized healthcare for individuals with long-term disabilities, while municipal services offer primary care services in the home environment for individuals living in group or supported housing. As can be seen in Table 1, the intervention was delivered individually by healthcare professionals representing five different professions. The study was approved by the Regional Ethics Committee (Etikprövningsmyndigheten) in Stockholm 2022-02920-01. All participants provided written informed consent prior to participation, in accordance with the Declaration of Helsinki. Data was collected 2023-01-25-2023-12-21 in Stockholm and Malmö at baseline (before the intervention) and immediately post the intervention using self-assessments from both patients and professionals administrating the intervention. All participants gave their written informed consent before inclusion in the study. The study adhered to the CONSORT 2010 Checklist. The study was registered at ClinicalTrials.gov (NCT05889936). The execution of this study deviated from the protocol described in the ethical application in two ways. 1) Our initial plan was to recruit a larger number of participants and include a control group in order to prepare for a potential randomized controlled trial. However, recruitment of clinicians proceeded more slowly than anticipated, and we therefore proceeded with a more standard feasibility design, without a control group and with a smaller sample size. 2) Due to the limited sample size, we also omitted the planned analysis of differences between healthcare professions in how the applied behavioral analysis was conducted. Neither of these changes violated the CONSORT checklist.

**Table 1:** Healthcare professions providing the intervention (n = 15).

Profession	*n*	%
Social worker	5	33
Physiotherapist	4	27
Psychologist	3	20
Occupational therapist	2	13
Nurse	1	7

*Note.* Percentages are rounded to one decimal.

### Participants and recruitment

The Lev-i intervention was presented at a national conference for habilitation services, where several regions expressed interest in participating in the study. To ensure manageability during the initial phase, we decided to begin with a limited number of services. Within these selected services, healthcare management determined which professionals would be included in the study. Participating professionals identified and invited patients at their centers whom they believed could benefit from and were motivated to work on their health behaviors. The number of approached individuals was not systematically recorded. The inclusion criteria required participants to be 18 years or older and to have a long-term disability, such as autism, intellectual disability, or a movement disability and speak Swedish. The goal was to recruit a referred sample representative of a typical outpatient habilitation setting, where comorbidities, such as Attention-Deficit/Hyperactivity Disorder (ADHD), anxiety and depression, are prevalent and therefore not considered exclusion criteria.

Exclusion criteria included individuals who required a support person to understand or complete the intervention. Additionally, individuals with severe mental or psychosocial instability that, in the professional’s judgment, would hinder participation were excluded. Examples of such conditions included severe depression or anxiety conditions, substance use issues, manic episodes, psychosis, suicide risk, or severe life circumstances, such as homelessness.

### The intervention

The Lev-i intervention is a brief, transdiagnostic, and interprofessional program designed to support individuals in making meaningful changes in health behaviors. It was developed following an intervention development framework [33] integrating stakeholder input from patients, professionals, and healthcare management aiming for feasibility and effectiveness. Lev consists of three sessions conducted over 10–20 weeks. The intervention allows participants individualized goals on one or two out of ten key health behaviors: physical activity, diet, tobacco use, alcohol use, illegal drug use, sexual health, sleep, meaningful activities, social relations, and screen health. Between sessions, participants were given structured home assignments designed to promote the application of session content in daily life. The first assignment involved engaging in activities that brought the participant closer to the goal set during the session. The second assignment focused on trying out strategies identified through a functional analysis conducted in the second session. The third assignment followed the same structure as the first but was based on the new goal established during the third session. To support adherence, the professional contacted each participant by phone approximately two weeks after each session to follow-up on their progress with the home assignment. The intervention follows a structured yet adaptable framework, ensuring that healthcare workers with various professional backgrounds can support behavior change without requiring in-depth expertise in all ten health behaviors. Instead, the focus is on providing support for goal-setting, follow-through, and overcoming biopsychosocial barriers, as participants often know what they need to do but struggle with execution.

#### Core components of Lev-i.

Lev is built on behavioral science principles, incorporating:

1. **Screening and Awareness** – The Lifestyle evaluation – screening (Lev-Screening or Lev-s) tool helps participants assess their current health behaviors and prioritize areas for change [34].
2. **Psychoeducation and Goal Setting** – Participants receive information about the chosen health behaviors and set **SMART goals** (Specific, Measurable, Attractive, Realistic, Time-bound) to create actionable plans [35].
3. **Motivational and Behavioral Strategies** – The intervention integrates Motivational Interviewing (MI), Applied Behavior Analysis (ABA), and Functional Mapping to help participants identify and address obstacles.
4. **Follow-up and Habit Formation** – Regular check-ins ensure sustained engagement, promoting long-term adherence to new health behaviors.

#### Professionals training and implementation.

Lev-i is designed for interprofessional use, enabling psychologists, occupational therapists, physiotherapists, and other professionals to integrate it into their practice. Training for professionals is provided through a five-module education, combining workshops, and supervised case discussions to enhance treatment fidelity and implementation. Training runs in parallel with seeing the first participant, allowing each module to be practiced before execution. Professionals also reflect on their experience after delivering each module.

#### Key innovations of Lev-i.

Unlike traditional interventions that focus on single health behaviors, specific diagnoses, or professional roles, Lev-i offers a flexible, transdiagnostic and scalable approach. The screening, its brief format, individualized focus, and structured follow-up make it feasible for diverse healthcare settings, including supporting individuals with disabilities and complex health needs [34].

#### Community involvement.

To ensure the intervention’s acceptability, we actively involved stakeholders from various disability advocacy groups, healthcare organizations, and regional healthcare programs throughout the development process. This collaborative approach ensured that diverse perspectives were integrated into the intervention design, enhancing its potential for real-world application and its alignment with the needs of the target population.

### Measurements

#### Demographic data.

Participants completed a demographic self-assessment form [36] to gather relevant background information. The results, including demographic and clinical characteristics, are summarized in Table 2.

**Table 2:** Patient characteristics for the participants who started the Lev-i intervention (n = 25).

**Mean age (SD, Range)**	37 (12, 20–63)
**Gender female *n* (%)**	13 (52%)
**Highest education *n* (%)**	
9-year compulsory school or less	4 (16%)
High school	17 (71%)
University degree or higher	3 (13%)
**Occupation *n* (%)**	
Employed/student	9 (36%)
Unemployed/sick leave/disability pension part or fulltime	16 (64%)
**Living situation *n* (%)**	
Alone	7 (29%)
With partner and/or children	5 (21%)
Parents, sibling, group home	12 (50%)
Disability*	
Autism	15 (60%)
Intellectual disability	7 (28%)
Movement disability	3 (12%)
Self-reported mental and physical health problems*	
ADHD	8 (32%)
Depression	5 (20%)
Anxiety	5 (20%)
PTSD	1 (4%)
Bipolar disorder	1 (4%)
Physical health problems	16 (64%)

*Note.* * Participants may belong to multiple sub-categories.

ADHD (Attention-Deficit/Hyperactivity Disorder).

PTSD (Post Traumatic Stress Disorder).

#### Primary outcomes.

**Treatment completion** was defined as the proportion of participants who attended at least two out of three intervention sessions, meeting the predefined threshold for adherence. **Acceptability** was investigated by measuring treatment credibility, satisfaction, and safety. ***Treatment credibility and expectancy of change*** was assessed using a modified version of the Treatment Credibility Scale (TCS) [37], administered before (Cronbach’s alfa 0.77, *n* = 24) and after the Lev-i intervention. The modified TCS comprises five items, each rated on a visual analog scale from 1 (e.g., “Not at all logical”) to 10 (e.g., “Very logical”), with the total score calculated as the mean of all item ratings—higher scores indicating greater perceived credibility. The questions included:

- *How logical/reasonable/sensible does the Lev-i intervention seem to you?*
- *How effective do you think this intervention is for helping you live healthier?*
- *How confident would you be in recommending this intervention to a friend wanting to change the same health behavior?*
- *How effective do you think this intervention is for other health behaviors?*
- *How much improvement do you expect to achieve from this intervention?*

Treatment credibility and expectancy of change was also assessed pre and post intervention by professionals who administrated Lev-i. Similarly, the TCS for professionals included five items, rated on a visual analog scale from 1 (e.g., “Not at all logical”) to 10 (e.g., “Very logical”), with the total score calculated as the mean of all item ratings—higher scores indicating greater perceived credibility. The questions included:

- *How logical/reasonable/sensible do you think this intervention (Lev-i) seems?*
- *How confident are you that this intervention will be successful in helping the participant live healthier?*
- *To what extent would you trust this intervention to recommend it to someone who wants to live healthier?*
- *How successful do you think this type of intervention would be for individuals with disabilities?*
- *How much improvement (in terms of individual goal achievement) do you expect participants to experience as a result of this intervention?*

***Treatment satisfaction*** was assessed using a modified version of the Evaluation Questionnaire [38] at the end of each session. The modified questionnaire includes the following eight statements, each rated on a 5-point Likert scale ranging from 0 (“Do not agree at all”) to 4 (“Totally agree”) with the total score calculated as the mean of all item ratings—higher scores indicating greater perceived satisfaction:

- *After today’s appointment, my knowledge of how I can change my health behaviors has increased.*
- *I will benefit from what we went through during today’s meeting.*
- *The content of the meeting felt relevant based on my own experiences.*
- *I understood the advice and strategies I received to help me change my health behaviors.*
- *I feel confident in how to use the advice and strategies.*
- *I will use the advice and strategies.*
- *I felt heard, and the practitioner understood my situation.*
- *I was actively involved and contributed with suggestions on how I can achieve my goals.*

A revised 12-item evaluation form, adapted from the Patient Evaluation Form (PEF) [39], was administered after the final session to assess participants’ overall perceptions of Lev-i. Five of the items utilized a Likert scale ranging from 0 (“Do not agree at all”) to 4 (“Totally agree”), capturing participants’ levels of agreement with various statements about the intervention. Patients also rated the Lev-i as a whole using one item on a 4-point scale from 0 (“Not approved”) to 3 (“Very well approved”). Cronbach’s alpha was not used to assess the internal consistency for items using the Likert scale due to the limited variance in responses. Given that participants’ responses were predominantly clustered around higher values, the assumptions required for a reliable Cronbach’s alpha estimate were not met. Additionally, four items in the PEF were open-ended, inviting participants to elaborate on their experiences.

- *I feel that participating in the program helped me in the following way*
- *How could the program have been improved?*
- *What could I have done differently?*
- *Is there anything else you would like to add?*

Treatment satisfaction was also assessed by the professionals who administered the Lev-i intervention. Two items were assessed after each session; each rated on a 5-point Likert scale ranging from 0 (“Do not agree at all”) to 4 (“Totally agree”) —higher scores indicating greater perceived treatment satisfaction:

- *The content in this session felt relevant.*
- *The difficulty level of conveying this session’s content was acceptable for me as a clinician.*

***Safety*** was measured by noting adverse events or serious adverse events, registered in the individual’s case report form. Adverse events were defined as spontaneous reports of any inconvenience that participants reported, and serious adverse events were anything that required inpatient hospitalization.

#### Secondary outcomes.

Preliminary effectiveness was evaluated using professionals’ assessments for goal attainment, self-assessment health behaviors, and quality of life.

**Goal attainment** was measured using a modified version of the Goal Attainment Scale (GAS) [40]. This version consists of four steps, with scores ranging from 0 (Present level of performance), 1 (Progress), 2 (Expected level of outcome), 3 (Somewhat more than expected), 4 (Much more than expected). In contrast to the traditional GAS, which ranges from −2 to +2, we used a 0–4 scoring system to enhance clarity and interpretability for participants. Importantly, we defined the lowest scale step as “no progress” rather than “getting worse,” as this framing aligns with the intervention’s focus on positive behavior change and prevents potential discouragement associated with negative scoring.

**The Lev-screening** (Lev-s) is a brief, 33-item tool designed to assess ten core health behaviors: physical activity, diet, alcohol use, tobacco use, illegal drug use, sleep, social relations, meaningful activities, sexual health, and screen health [34]. These behaviors align with the focus areas of the Lev-i intervention, ensuring a consistent framework for assessment and intervention. Each health behavior was assessed using a small number of items designed to capture different aspects of the behavior rather than measuring a single underlying construct. Since Cronbach’s alpha assumes that items reflect a unidimensional latent trait, it would not be an appropriate metric to evaluate reliability. Items are rated on a 4-point scale ranging from 0 (“High risk”) to 3 (“Healthy habits”), with higher scores indicating healthier behaviors. The results are presented using a traffic-light system to identify potential health risks and prioritize intervention targets.

**Quality of life** was measured using the WHOQOL-BREF [41] consisting of 26 items covering four domains of quality of life: Physical Health, Psychological Well-Being, Social Relationships, and Environment. Participants rated each item on a 5-point Likert scale ranging from 1 (e.g., “Very poor”) to 5 (e.g., “Very good”), with higher scores indicating better perceived quality of life. The internal consistency of the measure, as indicated by Cronbach’s alpha from baseline assessments (n = 24), was: Physical Health = .61, Psychological Well-Being = .66, Social Relationships = .78, and Environment = .73.

### Data analyses

Outliers were screened using boxplots and extreme outliers (values exceeding 3 × interquartile range) were inspected for data entry errors or implausibility. Removing the few outliers that were detected did not alter the findings, and they were therefore retained. Four participants had missing post-testing data for the Lev-s due to a misunderstanding at one of the participating centers. Three additional participants had missing post-data because they did not complete the intervention. Patterns of missing data were examined descriptively by reviewing participants’ diagnoses and other background characteristics to explore potential systematic patterns.

To evaluate changes in outcome variables between pre- and post-intervention assessments, we examined mean change including 95% confidence intervals as well as paired samples t-tests. Histograms were used to assess the distribution of variables for normality. We applied an intention-to-treat (ITT) approach where all participants with baseline data were included in the analyses. For participants who did not complete post-testing, baseline values were carried forward (i.e., last observation carried forward). We also conducted per-protocol (PP) analyses, including only participants who completed the intervention and provided full pre- and post-assessment data. Effect sizes for the contrasts were reported using Cohen’s d (0.2 = small, 0.5 = medium, 0.8 = large; [42]). The alpha level was set at p < .05. IBM SPSS Statistics version 28 was used for all analyses.

The responses to these open-ended items were analyzed using an inductive content analysis [43]. One of the authors (DS) coded the data and a second author (TH) checked the coding. The agreement was very high, and differences typically revolved around the correct naming of the category rather than the inherent content.

## Results

### Demographic and clinical characteristics

The sample consisted of 25 participants with a broad age range and an equal gender distribution. Most had completed high school, though a smaller proportion had a university degree. The majority were unemployed or on sick leave, and living arrangements varied, with a significant portion residing with family or in group homes. Clinically, neurodevelopmental conditions were common, particularly autism, ADHD and intellectual disability. Many participants also reported co-occurring mental health problems such as depression and anxiety, as well as physical illnesses. This highlights the complex health challenges within the sample. There were no clear systematic patterns in missingness related to participants’ diagnoses or demographic characteristics. Data used in the pre-post comparisons was roughly normally distributed.

### Feasibility

#### Treatment completion.

**Fig 1:**
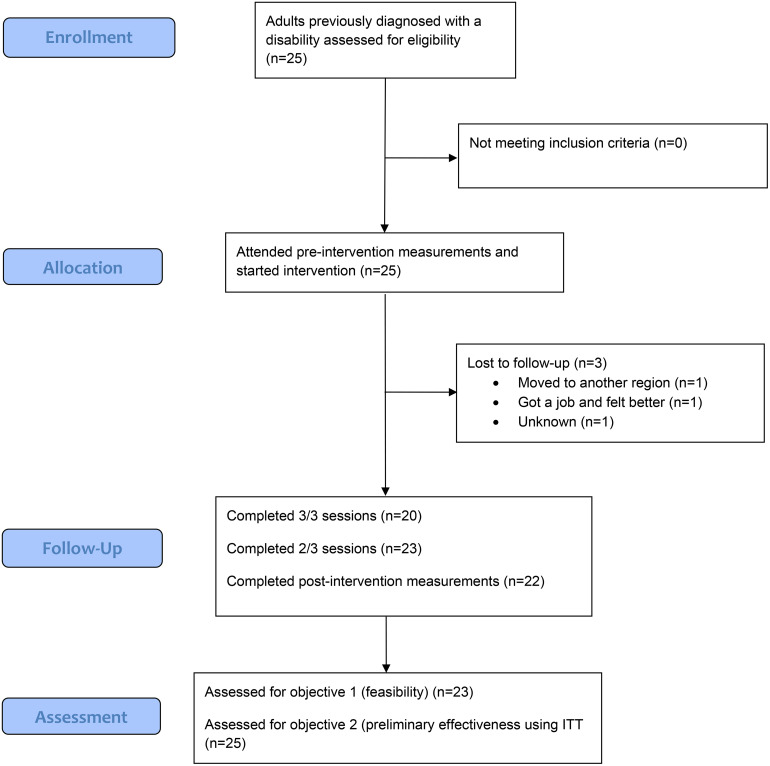
Flowchart participants.

**Fig 2:**
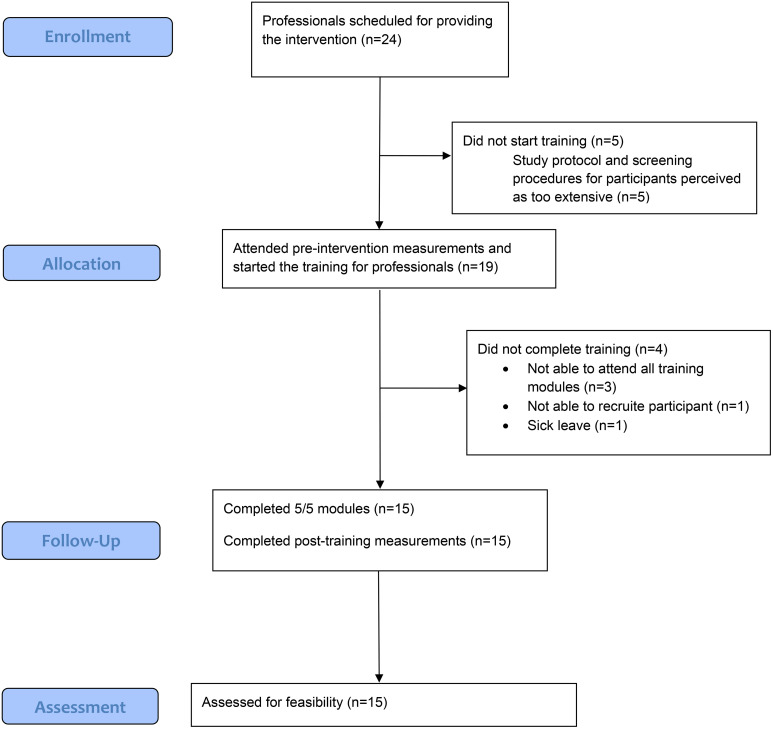
Flowchart professionals.

Of the 25 participants, 92% (*n* = 23) completed at least 2/3 sessions of the Lev-i intervention, predetermined as the cutoff for completion (see Fig 1 flowchart for participants). Of the 25 participants, 80% (*n* = 20) completed all three sessions. Furthermore, 80% (*n* = 20) completed at least 2/3 of the home assignments. Of the 24 professionals who had signed up for training, 5 withdrew before the study due to finding the screening and research protocol too extensive. Of the 19 professionals who started the training, 4 did not complete all modules or did not administrate the whole Lev-intervention to at least one patient (predetermined as the cutoff for implementation). The remaining 15 professionals completed all training sessions and administered Lev-i to at least one patient (Fig 2).

### Acceptability

#### Treatment credibility and expectancy of change.

As can be seen in Table 3, patients reported significantly higher treatment credibility and expectancy of change post (8.27/10) Lev-i intervention compared to pre (6.93/10) (*p* < 0.01). For professionals, no significant change between pre (6.94) and post measures (6.71) was observed.

**Table 3:** Pre- and post-measures of treatment credibility and expectations using the Treatment Credibility Scale (1-10 rating).

Pre-intervention Mean (SD)		Post-intervention Mean (SD)	Mean change [*95% CI*]	*t value*	Effect size *d (95% CI)*
Participants	6.93 (1.29) n = 22	8.27 (1.38) n = 22	1.34 [0.69–1.99]	4.29***	0.85 [0.33–1.35]
Professionals	6.94 (0.61) n = 14	6.71 (1.09) n = 14	−0.23 [-0.68–0.22]	1.09	0.29 [−0.24–0.82]

*Note*. *** *p* < 0.001. Effect sizes refer to Cohen’s *d,* brackets include the 95% confidence interval [CI].

#### Treatment satisfaction.

Ratings across items and sessions were consistently high, M = 3.62, on a 0–4-point scale, reflecting high patient satisfaction (see Table 4). Participants reported high levels of understanding advice and active participation. Ratings for “being listened to” were particularly notable, remaining near ceiling levels across sessions (M = 3.92–4.00). Regarding the open-ended question, *“Is there something else you want to mention?*”, very few responses were provided, with only 11 comments across all three sessions. Of these, 3 comments highlighted difficulties in answering questions, and 5 indicated that Lev-i had helped them.

**Table 4:** Session evaluations for patients and professionals using the modified Evaluation Questionnaire.

	Session 1	Session 2	Session 3
	Mean (SD)	Mean (SD)	Mean (SD)
**Participants**	**n = 24**	**n = 22**	**n = 21**
Knowledge increased	3.08 (0.88)	3.45 (0.60)	3.29 (1.01)
Content was useful	3.54 (0.78)	3.68 (0.48)	3.67 (0.48)
Content was relevant	3.54 (0.72)	3.77 (0.53)	3.81 (0.40)
Understood advice	3.58 (0.83)	3.73 (0.70)	3.80 (0.41)
Confident how to use advice	3.21 (0.83)	3.59 (0.59)	3.65 (0.75)
Will use advice	3.54 (.78)	3.48 (.93)	3.65 (0.59)
Was listened to	3.92 (0.28)	4.00 (0.00)	3.95 (0.22)
Participated actively	3.62 (0.71)	3.62 (0.74)	3.65 (0.81)
**Professionals**	**n = 15**	**n = 15**	**n = 15**
Content relevant	3.73 (0.46)	3.53 (0.52)	3.46 (0.66)
Difficulty level acceptable	3.07 (0.88)	2.93 (0.83)	2.92 (0.76)

*Note.* 0 (“Do not agree at all”) to 4 (“Totally agree”).

Professionals expressed satisfaction with the session content, rating the statement “The content in this session felt relevant” between 3.46 and 3.73 on a 0–4-point scale. Notably, while still high, the second item, “The difficulty level of conveying this session’s content was acceptable for me as a professional”, received slightly lower ratings, ranging from 2.92 to 3.07. The qualitative analysis of open-ended responses further confirmed that some professionals found the difficulty level of the sessions challenging. Several professionals noted that the material was difficult to convey within the intended number of sessions. This issue was particularly evident when working with patients with intellectual disability.

In addition to self-assessment of the session evaluations, satisfaction of the Lev-i intervention as a whole was collected after the last session (see Table 5). Overall, participant ratings were high, M = 3.52, on a 0–4-point scale, reflecting high satisfaction for the Lev- i intervention as a whole. Patients also rated the Lev-i as a whole using one item (0 = “Not approved”, 1 = “Approved”, 2 = “Well approved” or 3 = “Very well approved”) with a mean score of 2.65.

**Table 5:** Treatment satisfaction measured using the Patient Evaluation Form after completing Lev (n = 21).

Item	Mean (SD)
Overall, the intervention has been clearly focused on how I can live a healthier life	3.71 (0.56)
My knowledge of how I can adopt healthier lifestyle habits has increased	3.43 (0.75)
I can better manage problems related to my lifestyle habit	3.14 (0.96)
I have had the opportunity to provide my own input during the intervention	3.81 (0.40)
I can imagine participating in a similar intervention in the future	3.52 (0.93)

*Note*. 0=not at all, 4=yes, absolutely.

The content analysis of participants’ responses to the open-ended questions assessing the Lev-intervention as a whole, supported the high satisfaction reported in the quantitative assessments (see Table 6). Many participants highlighted that Lev-i helped them become more aware of their health behaviors and some that they succeeded in making behavioral changes. However, some patients expressed that they could have engaged more actively, while others noted the need for more clarity and accessibility of intervention materials. The need for clarity and accessibility was confirmed in the comments from professionals. The vast majority of these comments expressed the need to make materials and sessions easier for both the participants and professionals, especially for participants with intellectual disabilities.

**Table 6:** Content analysis of the responses of the open-ended questions regarding the intervention as a whole.

Question	Theme	Example quote
I feel that participating in the intervention helped me in this way	*n* = 19 individuals Total number of answers n = 19	
	Health behavior change *n* = 4	“Using screens less. Getting started with household tasks.”
	Increased awareness of my health behaviors *n* = 7	“It has helped me put my health behaviors into a better perspective and made me think more about my diet in general.”
	Self-determination *n* = 4	“Setting clearer goals. Understanding how I need to think in order to live in alignment with those goals.”
How could the intervention have been improved?	*n* = 13 individuals Total number of answers *n* = 13	
	Clarity and accessibility of educational materials *n* = 6	“Better questions. There has been too much text. I don’t understand everything.”
What could you have done differently?	*n* = 12 individuals Total number of answers *n* = 12	
	Self-determination *n* = 12	“I could have help on more to the new habits, but I am going to work on them again.”
Is there something else you want to add?	*n* = 9 individuals Total number of answers *n* = 9	
	Clarity and accessibility of educational materials *n* = 4	“More tips and inspiration would have been appreciated. Some of the questions were difficult.”
	Positive feedback *n* = 3	“Thank you for everything. Fantastic support. Fantastic psychologist. Great program.”

*Note*. Three answers were required to form a category. Uncategorized answers were typically along the lines of ‘I don’t know’ or similar.“

#### Adverse events and serious adverse events.

There were no serious adverse events reported. One patient working with goals related to a healthier diet, experienced stomach pain due to the new diet and routines for when eating. Yet another patient reported feeling inadequate when struggling to change health behaviors.

### Secondary outcomes

Goal attainment averaged 2.1 on a scale where 0 indicated no progress, 2 meant the goal was reached, and 4 represented much more progress than expected. The most common health behaviors that participants chose to work with was physical activity, diet and screen health. Notably, open-ended responses on treatment satisfaction revealed that several health behaviors were addressed indirectly while working toward the primary goal—for example, taking walks with friends or reducing evening screen time to improve sleep. As participants focused on one or two health behavior at a time, broad changes were not expected. As seen in Table 7, dietary habits improved significantly (*p* < 0.05, *d* = 0.55), and there was a non-significant increase in physical activity (*d* = 0.39) and sleep (*d* = 0.36). No notable effects were observed in other behaviors. There were small improvements across most domains of quality of life (physical, psychological, social, and environmental), but none reached statistical significance. The results of the PP analyses were comparable to the results of the ITT analyses, though effect sizes tended to be slightly larger in the per-protocol analyses.

**Table 7:** Secondary outcomes for patients using Lev-screening for Health behaviors and WHOQOL-BREF for Quality of life.

Outcome measures	Baseline Mean (SD)	Post-intervention Mean (SD)	Mean change [*95% CI*]	*n*	*t* value	Effect size *d* [*95% CI*]
***Health behaviors***						
Physical activity	1.39 (0.58)	1.59 (0.51)	0.21 [−0.02–0.43]	25	1.93	*d* = 0.39 [−0.03–0.81]
Diet	1.02 (0.68)	1.28 (0.66)	0.27 [0.06–0.47]	25	2.67*	*d* = 0.55 [−0.11–0.97]
Alcohol	2.55 (0.58)	2.62 (0.56)	0.07 [−0.11–0.25]	25	0.79	*d = *0.16 [−0.24–0.56]
Tobacco	2.00 (1.33)	2.00 (1.35)	0.00 [−0.18–0.18]	25	0.00	*d* = 0.00 [−0.40–0.40]
Illegal drugs	2.75 (0.78)	2.88 (0.61)	0.13 [−0.09–0.34]	25	1.19	*d* = 0.24 [−0.16–0.65]
Sleep	1.43 (0.79)	1.66 (0.74)	0.22 [−0.04–0.50]	25	1.77	*d *= 0.36 [−0.05–0.77]
Social relations	2.33 (0.65)	2.32 (0.62)	0.10 [−0.11–0.09]	25	0.21	*d* = 0.04 [−0.44–0.36]
Meaningful activities	1.93 (0.67)	1.97 (0.70)	0.04 [−0.24–0.32]	25	0.31	*d* = 0.06 [−0.33–0.46]
Sexual health	1.47 (1.29)	1.57 (1.24)	0.10 [−0.33–0.52]	22	0.46	*d* = 0.10 [−0.33–0.53]
Screen health	1.21 (0.93)	1.38 (.93)	0.17 [−0.28–0.61]	25	0.78	*d = *0.16 [−0.25–0.56]
***Quality of life***						
Physical	11.73 (2.34)	12.01 (2.43)	0.27 [−0.62–1.17]	25	0.63	*d* = 0.13 [−0.27–0.52]
Psychological	11.84 (2.79)	12.05 (3.10)	0.21 [−0.63–1.06]	25	0.52	*d* = 0.10 [−0.29–0.50]
Social	13.01 (4.11)	13.31 (3.73)	0.29 [−0.68–1.27]	25	0.62	*d* = 0.12 [−0.27–0.52]
Environment	13.59 (2.72)	13.90 (2.65)	0.31 [−0.28–0.91]	25	1.09	*d* = 0.22 [−0.18–0.61]

Note. * *p* < 0.05.

High values signify higher quality of life and healthier behaviors.

For health behaviors 0–1 indicate high risk for ill health, 1–2 risk for ill health, 2–3 healthy habits.

Mean change equals difference between Baseline and Post-intervention, brackets include the 95% confidence interval [CI].

Effect sizes refer to Cohen’s *d,* brackets include the 95% confidence interval [CI].

## Discussion

The present feasibility study evaluated the Lev-i intervention, a transdiagnostic and interprofessional approach designed to facilitate health behavior changes among individuals with disabilities. Our findings provide valuable insights into the acceptability, feasibility, and preliminary effectiveness of this intervention, contributing to the broader discourse on health promotion strategies tailored to diverse populations and healthcare settings.

### Feasibility

Improving health behaviors is crucial for preventing both physical and mental illness, reducing premature mortality, especially in individuals with disabilities [12–19]. However, individuals with disabilities are underserved [22,23] and in general, healthcare services are suggested to place a higher priority on health behavior interventions [27]. To remedy this problem, we developed Lev-i together with multiple stakeholders to be a transdiagnostic and interprofessional intervention that can support individuals in making changes across multiple health behaviors. In this study we began investigating the feasibility of Lev-i in a sample of adults with enduring disabilities as well as high levels of psychiatric co-occurrence. The high treatment completion rate (92% attending at least two out of three sessions) suggests that Lev-i is feasible within the studied contexts. The completion rate was high compared to interventions in similar outpatient contexts [44–46] demonstrating that the Lev-i can be completed by participants with disabilities. Importantly however, not all professionals scheduled to participate in the study started or completed the training. Hence, recruitment progressed more slowly than anticipated, and the target sample size was not reached within the planned timeframe. This highlights a potential challenge for scaling up the intervention in future trials and suggests that additional strategies to enhance recruitment efficiency will be needed.

### Acceptability

To get a more elaborated view on how the Lev-i was received we studied treatment credibility and expectancy of change, treatment satisfaction and safety. First, treatment credibility and expectancy of change was interpreted as satisfactory [47]. The increase in participants perceived credibility of Lev-i suggests a positive was positively affected by taking part in the intervention. The treatment credibility and expectancy of change was similar to a group intervention conducted in a similar healthcare context [44].

Patient treatment satisfaction ratings were consistently high, suggesting that the intervention was well-received. Notably, participants reported feeling heard and actively involved in the process, key elements in fostering motivation and adherence to behavior change interventions [48–50]. Indications of increased awareness and behavioral change were noted in the content analysis of the open-ended questions. However, some individuals highlighted difficulties in answering certain questions, which suggests that further refinement of the assessment materials may be necessary to improve accessibility for all participants, particularly those with intellectual disability. Professionals also expressed high levels of satisfaction, although about half of the professionals noted challenges in conveying the material within the allocated session time in the open-ended questions. One issue appeared to be that they found the intervention and study protocol too extensive and hard to follow, especially when working with participants with intellectual disability.

First, and most importantly, the findings suggest a need to revise both the Lev-i materials and study protocol to better support participants and professionals. Although, several professionals and participated in the development of Lev-i in line with guidelines for development of complex healthcare interventions [33], we did not manage fully accommodate the needs of the professionals. Second, the current format may be less suitable for individuals with intellectual disabilities, despite instructions during training for professionals to adapt sessions through clarification or repetition. Third, substantial variation of engagement and completion across healthcare centers indicates that non-participation may also stem from organizational factors. This aligns with findings from other studies, which have identified factors such as lack of time, and perceptions of research as not being part of the professionals role, as barriers to participation in clinical trials [51]. Also pointing to organizational factors, a recent systematic review emphasized the importance of assessing organizational readiness for change, highlighting that factors such as motivation, capacity, perceived importance of the intervention, are critical for successful implementation [28]. Furthermore, several sectors of healthcare struggle to shift from reactive to preventive practices. Integrating health behavior interventions often receives low priority when resources are scarce, although such efforts have been widely recognized as a valuable long-term strategy—benefiting both individual well-being and healthcare system efficiency [52].

### Preliminary effectiveness

While the primary aim of this study was to assess feasibility and acceptability, preliminary effectiveness data provide encouraging signs that Lev-i may facilitate meaningful behavior change. Goal attainment scores suggest that most participants made substantial progress toward their health behavior goals. Also, improvements on were noted several health behaviors even though broad changes were not expected as participants worked with one or two health behavior at a time. Given the lack of feasible and effective interventions available to individuals with disabilities [3,4] the preliminary effects warrant further investigation.

### Future revisions of the Lev-i intervention

While Lev-i showed high completion among participants and received high satisfaction ratings, feedback from professionals and patients suggests that a clearer version of the materials is needed. Some of the critiques likely stemmed from the demands of participating in the research, but it is clear that substantial revisions are necessary. These should focus on making the materials easier to use, preparing professionals to tailor the content to each patient, and clarifying that sessions can be split or repeated if needed. Also, Lev-i may benefit from strengthening professionals training to support more individualized delivery.

### Limitations

This feasibility study has several limitations that should be considered when interpreting the results. First, the sample size was small and drawn from a limited number of clinical settings, which restricts the generalizability of the findings. Second, the lack of a control group and reliance on self-reported outcomes prevent causal conclusions regarding the effectiveness of the intervention. These issues highlight the need for further development, evaluation in larger and more diverse samples, and testing using controlled designs. Finally, the trial was retrospectively registered at ClinicalTrials.gov after recruitment had started. Although the registration was completed before data analyses were initiated, the study followed the same design and outcomes as approved in the ethics application. Prospective registration would nevertheless have been preferable to further strengthen transparency and adherence to reporting standards.

## Conclusion

This feasibility study suggests that Lev-i is a promising transdiagnostic intervention for supporting health behavior change among individuals with disabilities. High completion rates, strong participant satisfaction, and initial improvements in key health behaviors indicate potential for further evaluation with controlled design. However, further refinement of materials and delivery is needed to enhance usability, particularly for individuals with intellectual disability.

## Supporting information

S1 File Study protocol . (PDF)

S2 File CONSORT checklist for pilot and feasibility trials. (DOC)

## References

[1] Wade DT, Halligan PW. The biopsychosocial model of illness: a model whose time has come. Clin Rehabil. 2017;31(8):995–1004. doi: 10.1177/0269215517709890 28730890

[2] World Health Organization. Disability. WHO; 2023. Available from: https://www.who.int/news-room/fact-sheets/detail/disability-and-health

[3] Gréaux M, Moro MF, Kamenov K, Russell AM, Barrett D, Cieza A. Health equity for persons with disabilities: a global scoping review on barriers and interventions in healthcare services. Int J Equity Health. 2023;22(1):236. doi: 10.1186/s12939-023-02035-w 37957602 PMC10644565

[4] Krahn GL, Walker DK, Correa-De-Araujo R. Persons with disabilities as an unrecognized health disparity population. Am J Public Health. 2015;105(Suppl 2):S198-206. doi: 10.2105/AJPH.2014.302182 25689212 PMC4355692

[5] Gasana J, O’Keeffe T, Withers TM, Greaves CJ. A systematic review and meta-analysis of the long-term effects of physical activity interventions on objectively measured outcomes. BMC Public Health. 2023;23(1):1697. doi: 10.1186/s12889-023-16541-7 37660119 PMC10474717

[6] Rigotti NA, Kruse GR, Livingstone-Banks J, Hartmann-Boyce J. Treatment of tobacco smoking: a review. JAMA. 2022;327(6):566–77. 35133411 10.1001/jama.2022.0395

[7] Ashton LM, Sharkey T, Whatnall MC, Williams RL, Bezzina A, Aguiar EJ, et al. Effectiveness of Interventions and Behaviour Change Techniques for Improving Dietary Intake in Young Adults: A Systematic Review and Meta-Analysis of RCTs. Nutrients. 2019;11(4). 10.3390/nu11040825 PMC6520715 30979065

[8] Zhang Y-B, Pan X-F, Chen J, Cao A, Xia L, Zhang Y, et al. Combined lifestyle factors, all-cause mortality and cardiovascular disease: a systematic review and meta-analysis of prospective cohort studies. J Epidemiol Community Health. 2021;75(1):92–9. doi: 10.1136/jech-2020-214050 32892156

[9] Muka T, Imo D, Jaspers L, Colpani V, Chaker L, van der Lee SJ, et al. The global impact of non-communicable diseases on healthcare spending and national income: a systematic review. Eur J Epidemiol. 2015;30(4):251–77. doi: 10.1007/s10654-014-9984-2 25595318

[10] Candari CJ, Cylus J, Nolte E. Assessing the economic costs of unhealthy diets and low physical activity: An evidence review and proposed framework. Copenhagen (Denmark): European Observatory on Health Systems and Policies; 2017. 28787114

[11] Zaman R, Hankir A, Jemni M. Lifestyle factors and mental health. Psychiatr Danub. 2019;31(Suppl 3):217–20. 31488729

[12] Aram J, Slopen N, Arria AM, Liu H, Dallal CM. Drug and alcohol use disorders among adults with select disabilities: The national survey on drug use and health. Disabil Health J. 2023;16(3):101467. doi: 10.1016/j.dhjo.2023.101467 37088676

[13] Påhlsson-Notini A, Liu S, Tideman M, Latvala A, Serlachius E, Larsson H, et al. Substance use-related problems in mild intellectual disability: A Swedish nationwide population-based cohort study with sibling comparison. JCPP Adv. 2024;4(2):e12225. doi: 10.1002/jcv2.12225 38827981 PMC11143951

[14] van Amsterdam J, van der Velde B, Schulte M, van den Brink W. Causal Factors of Increased Smoking in ADHD: A Systematic Review. Subst Use Misuse. 2018;53(3):432–45. doi: 10.1080/10826084.2017.1334066 29039714

[15] Boltri M, Sapuppo W. Anorexia Nervosa and Autism Spectrum Disorder: A Systematic Review. Psychiatry Res. 2021;306:114271. doi: 10.1016/j.psychres.2021.114271 34798485

[16] Ahlberg R, Garcia-Argibay M, Hirvikoski T, Boman M, Chen Q, Taylor MJ, et al. Shared familial risk factors between autism spectrum disorder and obesity - a register-based familial coaggregation cohort study. J Child Psychol Psychiatry. 2022;63(8):890–9. doi: 10.1111/jcpp.13538 34881437

[17] Al Lihabi A. A literature review of sleep problems and neurodevelopment disorders. Front Psychiatry. 2023;14:1122344. doi: 10.3389/fpsyt.2023.1122344 36911135 PMC9995546

[18] Hirvikoski T, Mittendorfer-Rutz E, Boman M, Larsson H, Lichtenstein P, Bölte S. Premature mortality in autism spectrum disorder. Br J Psychiatry. 2016;208(3):232–8. doi: 10.1192/bjp.bp.114.160192 26541693

[19] Hirvikoski T, Boman M, Tideman M, Lichtenstein P, Butwicka A. Association of Intellectual Disability With All-Cause and Cause-Specific Mortality in Sweden. JAMA Netw Open. 2021;4(6):e2113014. doi: 10.1001/jamanetworkopen.2021.13014 34156453 PMC8220491

[20] SBU. Beteendeinriktade insatser inom vård, omsorg och socialtjänst för att öka fysisk aktivitet. Stockholm: Statens beredning för medicinsk och social utvärdering (SBU); 2025. Available from: https://www.sbu.se/387

[21] Nyanchoka M, Alade OT, Petkovic J, Duque T, Wieland LS. A review of health equity considerations in Cochrane reviews of lifestyle interventions for cardiovascular health in adults. J Clin Epidemiol. 2024;176:111546. doi: 10.1016/j.jclinepi.2024.111546 39343415

[22] Molloy R, Brand G, Munro I, Pope N. Seeing the complete picture: A systematic review of mental health consumer and health professional experiences of diagnostic overshadowing. J Clin Nurs. 2023;32(9–10):1662–73. doi: 10.1111/jocn.16151 34873769

[23] Hatzikiriakidis K, Ayton D, O’Connor A, Carmody S, Patitsas L, Skouteris H, et al. The delivery of healthy lifestyle interventions for people with disability living in supported accommodation: a scoping review of intervention efficacy and consumer involvement. Disabil Health J. 2023;16(2):101444. doi: 10.1016/j.dhjo.2023.101444 36792486

[24] Shahane V, Kilyk A, Srinivasan SM. Effects of physical activity and exercise-based interventions in young adults with autism spectrum disorder: A systematic review. Autism. 2024;28(2):276–300. doi: 10.1177/13623613231169058 37128159

[25] Rana D, Westrop S, Jaiswal N, Germeni E, McGarty A, Ells L, et al. Lifestyle modification interventions for adults with intellectual disabilities: systematic review and meta-analysis at intervention and component levels. J Intellect Disabil Res. 2024;68(5):387–445. doi: 10.1111/jir.13098 38414293

[26] Ward JH, McBride A, Price A, Delgado TN. Psychosocial interventions for improving the physical health of young people and adults with attention deficit hyperactivity disorder: a scoping review. BMC Psychiatry. 2024;24(1):569. doi: 10.1186/s12888-024-06009-2 39164688 PMC11337789

[27] Swedish National Board of Health and Welfare. Nationella riktlinjer 2024 - Vård vid ohälsosamma levandsvanor - Prioriteringsstöd till dig som beslutar om resurser för sjukdomsprevention och behandling: Socialstyrelsen; 2024. Available from: https://www.socialstyrelsen.se/kunskapsstod-och-regler/regler-och-riktlinjer/nationella-riktlinjer/riktlinjer-och-utvarderingar/levnadsvanor/

[28] Hatzikiriakidis K, Ayton D, O’Connor A, Callaway L, Carmody S, Skouteris H, et al. Barriers and enablers to the implementation of healthy lifestyle interventions for people with disability living in supported accommodation: A systematic review using the consolidated framework for implementation research. Disabil Health J. 2023;16(2):101442. doi: 10.1016/j.dhjo.2023.101442 36740546

[29] Wang T, Tan J-YB, Liu X-L, Zhao I. Barriers and enablers to implementing clinical practice guidelines in primary care: an overview of systematic reviews. BMJ Open. 2023;13(1):e062158. doi: 10.1136/bmjopen-2022-062158 36609329 PMC9827241

[30] Porter J, Boyd C, Skandari MR, Laiteerapong N. Revisiting the Time Needed to Provide Adult Primary Care. J Gen Intern Med. 2023;38(1):147–55. doi: 10.1007/s11606-022-07707-x 35776372 PMC9848034

[31] Johansson M, Guyatt G, Montori V. Guidelines should consider clinicians’ time needed to treat. BMJ. 2023;380:e072953. doi: 10.1136/bmj-2022-072953 36596571

[32] Newby JM, McKinnon A, Kuyken W, Gilbody S, Dalgleish T. Systematic review and meta-analysis of transdiagnostic psychological treatments for anxiety and depressive disorders in adulthood. Clin Psychol Rev. 2015;40:91–110. doi: 10.1016/j.cpr.2015.06.002 26094079

[33] O’Cathain A, Croot L, Duncan E, Rousseau N, Sworn K, Turner KM, et al. Guidance on how to develop complex interventions to improve health and healthcare. BMJ Open. 2019;9(8):e029954. doi: 10.1136/bmjopen-2019-029954 31420394 PMC6701588

[34] Sjöwall D, Stålhand F, Schettini G, Gustavsson P, Hirvikoski T. Global screening of health behaviors: Introducing Lev-screening (Lev-s)-development and psychometric evaluation. PLoS One. 2024;19(12):e0315565. doi: 10.1371/journal.pone.0315565 39724222 PMC11670939

[35] Bailey RR. Goal Setting and Action Planning for Health Behavior Change. Am J Lifestyle Med. 2017;13(6):615–8. doi: 10.1177/1559827617729634 31662729 PMC6796229

[36] Hirvikoski T, Lindholm T, Nordenström A, Nordström A-L, Lajic S. High self-perceived stress and many stressors, but normal diurnal cortisol rhythm, in adults with ADHD (attention-deficit/hyperactivity disorder). Horm Behav. 2009;55(3):418–24. doi: 10.1016/j.yhbeh.2008.12.004 19162030

[37] Borkovec TD, Nau SD. Credibility of analogue therapy rationales. J Behav Ther Exp Psychiatry. 1972;3(4):257–60. doi: 10.1016/0005-7916(72)90045-6

[38] Bramham J, Young S, Bickerdike A, Spain D, McCartan D, Xenitidis K. Evaluation of group cognitive behavioral therapy for adults with ADHD. J Atten Disord. 2009;12(5):434–41. doi: 10.1177/1087054708314596 18310557

[39] Hesslinger B, Tebartz van Elst L, Nyberg E, Dykierek P, Richter H, Berner M, et al. Psychotherapy of attention deficit hyperactivity disorder in adults--a pilot study using a structured skills training program. Eur Arch Psychiatry Clin Neurosci. 2002;252(4):177–84. doi: 10.1007/s00406-002-0379-0 12242579

[40] Ruble L, McGrew JH, Toland MD. Goal attainment scaling as an outcome measure in randomized controlled trials of psychosocial interventions in autism. J Autism Dev Disord. 2012;42(9):1974–83. doi: 10.1007/s10803-012-1446-7 22271197 PMC3358457

[41] Skevington SM, Lotfy M, O’Connell KA, WHOQOL Group. The World Health Organization’s WHOQOL-BREF quality of life assessment: psychometric properties and results of the international field trial. A report from the WHOQOL group. Qual Life Res. 2004;13(2):299–310. doi: 10.1023/B:QURE.0000018486.91360.00 15085902

[42] Cohen J. Statistical power analysis for the behavioral sciences. New York: Routledge Academic; 1988.

[43] Hsieh H-F, Shannon SE. Three approaches to qualitative content analysis. Qual Health Res. 2005;15(9):1277–88. doi: 10.1177/1049732305276687 16204405

[44] Hidalgo N, Sjöwall D, Agius H, Byström C, Brar A, Borg J, et al. Psychoeducational group intervention for intellectually able adults with autism and their close relations (Prisma) - an open feasibility study. BMC Psychiatry. 2022;22(1):556. doi: 10.1186/s12888-022-04134-4 35986348 PMC9389708

[45] Sjöwall D, Berglund M, Hirvikoski T. Computerized working memory training for adults with ADHD in a psychiatric outpatient context-a feasibility trial. Appl Neuropsychol Adult. 2025;32(1):196–204. doi: 10.1080/23279095.2022.2162900 36639362

[46] Mazzotti E, Barbaranelli C. Dropping out of psychiatric treatment: a methodological contribution. Acta Psychiatr Scand. 2012;126(6):426–33. doi: 10.1111/j.1600-0447.2012.01872.x 22616956

[47] Pahnke J, Jansson-Fröjmark M, Andersson G, Bjureberg J, Jokinen J, Bohman B, et al. Acceptance and commitment therapy for autistic adults: A randomized controlled pilot study in a psychiatric outpatient setting. Autism. 2023;27(5):1461–76. doi: 10.1177/13623613221140749 36510817 PMC10291362

[48] Thompson-Hodgetts S, Ryan J, Coombs E, Brown HM, Xavier A, Devlin C, et al. Toward understanding and enhancing self-determination: a qualitative exploration with autistic adults without co-occurring intellectual disability. Front Psychiatry. 2023;14:1250391. doi: 10.3389/fpsyt.2023.1250391 37743989 PMC10514482

[49] Ntoumanis N, Ng JYY, Prestwich A, Quested E, Hancox JE, Thøgersen-Ntoumani C, et al. A meta-analysis of self-determination theory-informed intervention studies in the health domain: effects on motivation, health behavior, physical, and psychological health. Health Psychol Rev. 2021;15(2):214–44. doi: 10.1080/17437199.2020.1718529 31983293

[50] Cole SA, Sannidhi D, Jadotte YT, Rozanski A. Using motivational interviewing and brief action planning for adopting and maintaining positive health behaviors. Prog Cardiovasc Dis. 2023;77:86–94. doi: 10.1016/j.pcad.2023.02.003 36842453

[51] Smith KV, Thew GR. Conducting research in clinical psychology practice: Barriers, facilitators, and recommendations. Br J Clin Psychol. 2017;56(3):347–56. doi: 10.1111/bjc.12142 28569400 PMC5575503

[52] AbdulRaheem Y. Unveiling the Significance and Challenges of Integrating Prevention Levels in Healthcare Practice. J Prim Care Community Health. 2023;14:21501319231186500. doi: 10.1177/21501319231186500 37449436 PMC10350749

