# Supplementary material for: Feasibility trial of a transdiagnostic individual lifestyle evaluation and intervention (Lev-i) for health behavior change
Source: PLoS One. 2026 Jan 12;21(1):e0339500. doi: 10.1371/journal.pone.0339500 (PMC12795380; doi:10.1371/journal.pone.0339500)
Supplement: S1 File — (PDF) [file pone.0339500.s001.pdf]

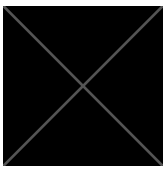

Lev-projektet: Kartläggning, bedömning och intervention av hälsorelaterade levnadsvanor hos vuxna med och utan funktionsnedsättning.

2022-02920-01
Grundansökan
Grundansökan
Avslutad

Stig Douglas Filip Sjöwall

1.2 Ansvarig huvudman för forskningen (forskningshuvudman)

Region Stockholm (232100-0016)

1.3 Behörig företrädare för forskningshuvudman

Joakim Lavesson

1.3.1 Behörig företrädare – titel som innebär ett verksamhetsansvar

Verksamhetschef Habilitering & Hälsa, Region Stockholm

1.4 Har projektet fler forskningshuvudmän?

Nej

1.5 Hemvist för forskningen

Habilitering & Hälsa, Region Stockholm

1.6 Huvudansvarig forskare för projektet (kontaktperson)

Douglas Sjöwall

1.6.1 Institution/hemvist som huvudansvarig forskare är verksam vid

Institutionen för kvinnor och barns hälsa, Karolinska institutet. FoUU-enheten vid Habilitering & Hälsa, Region Stockholm.

1.7 Är den huvudansvariga forskaren disputerad?

Ja

1.8 Andra medverkande:

Tatja Hirvikoski

Frågor för avgiftskategori

1.9 Hur många forskningshuvudmän kommer att ingå i forskningsprojektet?

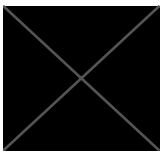

En

1.9.1 [Om Flera] Har samtliga forskningspersoner ett omedelbart samband med endast en av forskningshuvudmännen?

Nej

1.10 Avser forskningen klinisk läkemedelsprövning?

Nej

1.11 Ska endast befintliga personuppgifter behandlas i projektet?

Nej

2.1 Avser ansökan forskning som inbegriper äggdonation?

Nej

2.2 Avser ansökan forskning med läkemedel för genterapi eller somatisk cellterapi eller läkemedel som innehåller genetiskt modifierade organismer?

Nej

2.3 Avser ansökan forskning med xenogen cellterapi?

Nej

2.4 Kommer joniserande strålning ingå i forskningsprojektet?

Nej

2.5 Kommer biologiskt material från människor att nyinsamlas för projektet?

Nej

2.6 Planerar projektet att använda biologiskt material från människor från en eller flera befintliga provsamlingar?

Nej

2.7 Avser forskningen klinisk prövning eller en prestandastudie av medicinteknisk produkt/medicinteknisk produkt för in vitro-diagnostik?

Nej

2.8 Gör en egen bedömning och ange på vilka punkter nedan som forskningen omfattas av 3-4 §§ etikprövningslagen. Observera att

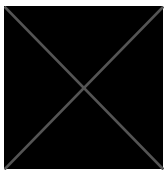

myndigheten kan komma att göra en annan bedömning.

✓ 3 § 1 Forskningen kommer att samla in känsliga personuppgifter.
✓ 4 § 2 Forskningen utförs enligt en metod som syftar till att påverka forskningspersonen fysiskt eller psykiskt, eller så innebär forskningen en uppenbar risk att skada forskningspersonen.

2.8.1 [Om 3 § 1] Gör en egen bedömning och ange vilken typ av känsliga personuppgifter som kommer att behandlas i projektet. Observera att myndigheten kan komma att göra en annan bedömning.

✓ hälsa
✓ en persons sexualliv eller sexuella läggning

2.9 Önskas ett rådgivande yttrande?

Ja

2.10 Söker projektet förtur med motivering att projektet har tydlig potential att ge nytta i närtid för behandling och förebyggande av COVID-19?

Nej

3.1 Skriv en populärvetenskaplig sammanfattning av forskningsprojektet.

Ohälsosamma levnadsvanor leder enligt WHO till psykiskt och fysiskt lidande. Situationen är särskilt allvarlig hos personer med funktionsnedsättning som dör i förtid av behandlingsbara livsstilsrelaterade sjukdomar. I det här projektet utvärderar vi ett kartläggningsverktyg (Lev-s) och en intervention (Lev) som syftar till att motverka och behandla ohälsosamma levnadsvanor. Dessa verktyg kan administreras av olika vårdprofessioner till alla som behöver stöd för att skapa hälsosamma levnadsvanor men har utvecklats särskilt i åtanke för personer med funktionsnedsättning. Lev-s innehåller 33 frågor om 10 levnadsvanor och Lev innehåller tre sessioner som bygger på psykoedukation, motiverande samtal samt tillämpad beteendeanalys. Lev-s och Lev följer socialstyrelsens och region Stockholms riktlinjer för prevention och behandling av ohälsosamma levnadsvanor och har utvecklats och testats under 2 år i ett samarbete mellan forskare, behandlare och patientorganisationer. Vi som söker etiskt godkännande har en stor samlad erfarenhet av utveckling av interventioner, mätinstrument och har genomfört och publicerat studier gällande hälsa för olika funktionsnedsättningar. Mätverktyget och interventionen som utvärderas är ett viktigt steg i att utveckla genomförbara, effektiva och evidensbaserade metoder för att motverka ohälsa hos personer med funktionsnedsättning.

3.2 Vad är det vetenskapliga syftet med projektet?

Syftet är att generera kunskap och metoder för att motverka ohälsosamma levnadsvanor hos personer med funktionsnedsättning. Utvärdering av ett kartläggningsverktyg för levnadsvanor (Lev-s) och en manualiserad, transdiagnostisk intervention (Lev) är ett fokus men syftet är även att generera ny kunskap om funktionsnedsättning och levnadsvanor. Projektet inkluderar endast personer över 18 år som inte är beroende av en medföljare.

3.3 Vilka är de vetenskapliga frågeställningarna?

Studie 1: Psykometriska egenskaper hos Lev-s.

a) Vad är reliabiliteten för Lev-s ?
b) Vad är överensstämmelsen mellan Lev-s som självskattning och som intervju?
c) Hur korrelerar olika levnadsvanor med livskvalitet?

Studie 2: Levnadsvanor hos vuxna med och utan funktionsnedsättning.

a) Hur skiljer sig levnadsvanor mellan olika funktionsnedsättningar och i jämförelse med personer utan funktionsnedsättning?
b) Vilken är effekten av kön, ålder, SES på levnadsvanor hos individer med funktionsnedsättning?
c) Vilka levnadsvanor uppfattas som viktigast av personer med och utan funktionsnedsättning?
d) Vilken är upplevelsen av stöd från samhället gällande levnadsvanor?
e) Vilka hinder uppfattar personer med och utan funktionsnedsättning gällande att leva hälsosamt?

Studie 3: En transdiagnostisk intervention för hälsosammare levnadsvanor (Lev): En öppen kontrollerad genomförbarhetsstudie.

a) Hur många av vårdpersonalen som fått utbildning i Lev började använda Lev inom 6 månader? Hur många deltagare som erbjöds Lev deltog?
b) Vilka anledningar fanns till att inte delta?
c) Hur många faser och hemuppgifter slutförde deltagarna?
d) Finns det några skillnader mellan vårdprofessionerna när det gäller hur den tillämpade beteendeanalysen utfördes?
e) Uppfattades Lev som en trovärdig insats av vårdpersonal och deltagare?
f) Fanns det någon del av Lev som upplevdes mindre tillfredsställande av deltagare och vårdpersonal?
g) Leder Lev till några oönskade effekter?
h) Leder Lev till hälsosammare vanor?
i) I vilken utsträckning uppnåddes individuella mål?
j) Leder Lev till ökad livskvalitet?

Studie 4: En tematisk analys av en funktionell analys av levnadsvanor hos vuxna med funktionsnedsättning.

a) Vilka generella teman bakom ohälsosamma och hälsosamma vanor kan hittas hos personer med funktionsnedsättning?
b) Hur varierar teman för specifika levnadsvanor?
c) Hur varierar teman för specifika funktionsnedsättningar?
d) Hur varierar teman för frågor b och c beroende av kön?

4.1 Redogör för metod inkl. proceduren, tekniken eller behandlingen.

Centralt för projektets 4 studier är screeninginstrumentet Lev-s och interventionen Lev. Lev-s täcker tio levnadsvanor med totalt 33 frågor om sömn, kost, fysisk aktivitet, sociala relationer,

sexuell hälsa, fritidsaktiviteter och konsumtion av alkohol, tobak och illegala droger och tar ca 20-25 minuter att genomföra. Lev-s har utvecklats under två år tillsammans med behandlare och experter inom respektive levnadsvana och har utvärderats i en examensuppsats på psykologprogrammet på Karolinska Institutet med lovande resultat. Lev inkluderar tre sessioner innehållande psykoedukation, motiverande samtal och tillämpad beteendeanalys. Lev-s och Lev beskrivs i två manualer (se bilagor ”teoretisk” och ”praktisk manual”), innehåller en utbildning och har pilottestats på två Habiliteringscenter i Region Stockholm. Data samlas in via Habilitering & Hälsa i regions Stockholm, habiliteringar från andra regioner i Sverige, hälso- och sjukvårdsverksamhet i Stockholms stad, samt intresseorganisationer för de olika funktionsnedsättningar som deltar. Deltagare kommer även att rekryteras via sociala medier med hjälp av Karolinska institutets kommunikationsavdelning för att vi ska kunna få ett tillräckligt deltagarunderlag.

Studie 1: Psykometriska egenskaper hos Lev-s
Studie 1 omfattar minst 150 deltagare med och utan funktionsnedsättning. Data kommer att samlas främst via enkäter online, i videomöten eller på kliniker i förhållande till den vanliga kliniska praktiken. Deltagarna kan ha flera funktionshinder, inklusive fysiska. Reliabilitet undersöks genom test-retest av Lev-s med två veckors mellanrum. Vissa deltagarna gör Lev-s en gång som en självskattning och en gång när den administreras som en intervju. Bakgrundsinformation samlas in. Alla fyller även i WHOs frågeformulär WHOQOL-brief (Se bilaga WHOQOL) för att kunna undersöka hur Lev-s olika områden är relaterade till livskvalitet och välbefinnande.

Studie 2: Levnadsvanor hos vuxna med funktionsnedsättning.

Vi strävar efter att inkludera 200 kontroller (utan funktionsnedsättning) och 200 för varje grupp av funktionshinder (autism, intellektuell funktionsnedsättning, förvärvad hjärnskada, rörelsestörning, dövblindhet, döva). Deltagarna kan ha samsjukliga neuropsykologiska diagnoser (t.ex. ADHD) eller andra psykiska problem (t.e.x. ångest, depression). Det är svårt att förutse hur många vi kommer att nå och som accepterar att delta från varje grupp av funktionshinder. Rekrytering kommer att ske både genom plattformar på KI, Habilitering och intresseorganisationer för de olika patientgrupperna.

Studie 3: En transdiagnostisk intervention för hälsosammare levnadsvanor (Lev): En öppen kontrollerad genomförbarhetsstudie.

Studien kommer att registreras på clinicaltrials.org och följa CONSORTs checklista. Ålder, kön, subjektiv skattning av hälsa, funktionsnedsättning, behandling, sysselsättning samt högsta utbildningsnivå noteras i Lev - bakgrundsinformation. Deltagande noteras på sedvanligt sätt i ett flödesdiagram för både deltagare och behandlare. Vi kommer även genomföra en kvalitativ tematisk analys av motiv till att inte delta/avbryta. Deltagande undersöks även gällande slutförda sessioner och hemuppgifter. Lev är ämnad att kunna användas oavsett vårdprofession och vi kommer därför göra en tematisk analys av de beteenden och strategier som noteras i den funktionella analysen som är del av Lev. Det kommer ge oss svar på om olika vårdprofessioner genomför den på olika sätt och om vi behöver anpassa utbildning eller intervention som ett resultat av detta.

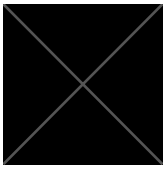

Förtroende för Lev mäts före och efter behandlingen för både deltagare och behandlare (se bilaga för TCS). Nöjdhet mäts efter varje session (se bilaga utvärdering session) och efter att behandlingen avslutats (se bilaga utvärdering helhet). Oönskade effekter mäts genom klagomål eller incidenter som rapporteras av deltagare. Behandlingseffekter mäts via Lev-s, måluppfyllelse (se bilaga måluppfyllelse) och livskvalitet (se bilaga WHOQOL-BREF). Lev-s och WHOQOL-BREF jämförs med kontrollgruppen från delstudie 1 och 2.

Studie 4: En tematisk analys av en funktionell analys av levnadsvanor hos vuxna med funktionsnedsättning.

Baserat på den funktionella analysen som genomförs i studie 3 analyseras data med en tematisk analys för att identifiera situationella och beteende prediktorer för olika funktionsnedsättningar. I denna studie inkluderas även de demografiska variabler. Syftet är att identifiera både generella och specifika faktorer som är kopplade till både hälsosamma och ohälsosamma levnadsvanor.

4.2 Redogör för på vilket sätt metoden skiljer sig från klinisk rutin eller den ordinarie behandlingen.

Lev-s och Lev kan genomföras som del av den ordinarie behandlingen. Att ställa frågor om levnadsvanor och hälsa genomförs i nuläget men omfattar inte lika många områden som i Lev-s samt sker inte på ett systematiskt sätt och lika på olika enheter. De huvudsakliga metoderna i Lev (psykoedukation, tillämpad beteendeanalys och motiverande samtal) utgör redan etablerade arbetssätt inom Habiliteringen. Det som avviker från klinisk rutin är mängden formulär och extra frågor som behöver besvaras för att kunna undersöka psykometriska egenskaper för Lev-s och genomförbarhet för Lev. De formulär som fyllas i som syftar till att mäta genomförbarhet har används vid likande forskningsprojekt vid habiliteringen. Både Lev-s har Lev har utvecklats tillsammans med behandlare från olika professioner och patientrepresentanter för olika funktionsnedsättningar och följer nationella och regionala vårdprogram gällande prevention av ohälsosamma levnadsvanor.

4.3 Redogör för tidigare erfarenheter (egna och/eller andras) av den använda proceduren, tekniken eller behandlingen.

Att ställa frågor om levnadsvanor och hälsa genomförs i nuläget av behandlare på Habiliteringen men omfattar inte lika många områden som i Lev-s och sker inte på ett systematiskt sätt. De huvudsakliga metoderna i Lev (psykoedukation, tillämpad beteendeanalys och motiverande samtal) utgör redan etablerade arbetssätt inom Habiliteringen. Behandlarna har erfarenhet av att ge snarlik behandling och får dessutom utbildning och handledning parallellt med att de träffar sina första försökspersoner.

Sökanden och medsökande har genomfört projekt med likande upplägg i samband med att nya interventioner utvecklats och genomförbarhet utvärderats. Huvudsökande arbetar som projektledare på FoUU-enheten på Habilitering & Hälsa, Region Stockholm som är den organisation från vilken huvuddelen av försökspersonerna kommer rekryteras ifrån. Han har främst utvärderat interventioner i skolmiljö men har under de senaste åren deltagit i utveckling och vetenskaplig utvärdering av en psykoedukativ insats för vuxna med autism och deras närstående där första manuset är under granskning för publicering (Hidalgo, Sjöwall et al., 2022).

Han har även deltagit som medförfattare i en multicenter RCT av en arbetsterapeutisk intervention som koordinator Habiliteringen i Regions Stockholm (Peny-Dahlstrand et al., 2022) Medsökande har lett flera likande projekt i Habiliterings- och psykiatrikontext (t. ex. Hirvikoski et al., 2017) och studerat hälsa hos flera olika funktionsnedsättningar (t. ex. Hirvikoski et al., 2021).

5.1 Förväntat startdatum för projektet:

2022-09-01

5.2 Förväntat slutdatum för projektet:

2025-12-31

5.3 Tidsplan för de olika delar som ingår i projektet:

Datainsamlingarna för delstudie 1 och 2 är planerade att starta under hösten 2022 och pågå under ca ett år. Delstudie 3 och 4 påbörjas i januari 2023 och väntas pågå ca 1 år. Datainsamlingen för studie 1 och 2 kommer avslutas först och i samband med det kommer data analyseras och sammanfattas i två vetenskapliga artiklar. Analys och författande av studie 3 och 4 sker efter att uppföljningen är klar. Studien kan genomföras helt på distans men med tanke på att Covid-19 pandemin lett till förseningar av tidigare datainsamlingar har vi satt det planerade avslutningen av datainsamlingen till slutet av 2025. Studie 1 och 2 är en datainsamling och studie 3 och 4 är en.

6.1 Redogör för datainsamling och datas karaktär.

Studie 1
Majoriteten av data kommer samlas in via e-enkäter utan att att deltagaren behöver uppge namn eller personnummer. För deltagare som ska fylla test-retest länkas data mellan T1 och T2 genom bakgrundsinformationen utan av vi vet vem denna person är. För dessa deltagare inhämtas inget samtycke. För ett delsample kommer data att samlas in genom att fylla i bedömningar online i videomöten eller på plats inom ramen för den ordinarie kliniska verksamheten. Dessa deltagare får fylla i samtycke. Alla deltagare kommer att fylla i Lev-s och WHOQOL samt information om livssituation (tidsåtgång ca 50 min) vid två tillfällen.

Studie 2 genomförs helt anonymt (dvs ej namn och personnummer). Deltagarna fyller i Lev-s och WHOQOL-BREF, bakgrunds information samt svar på frågor om vad de upplever som viktigast, vilket stöd och vilka hinder som finns för att leva hälsosamt.

Studie 3 och 4
Studie 3 och 4 inkluderar ca 100 vuxna deltagare med funktionsnedsättning. Deltagande vårdpersonal kommer att ge deltagarna information om studien och deltagare som samtycker kommer att fortsätta att göra Lev. Vårdpersonal får innan deltagande en utbildning online och stöd kommer även att ges under genomförandet av interventionen. Screeningen (Lev-s) och de tre sessionerna i Lev (inklusive psykoedukation, motiverande intervjuer och tillämpad beteendeanalys) kommer att administreras online i videomöten eller på kliniker. Deltagare och vårdpersonal kommer att fylla i bedömningar individuellt i slutet av var och en av de tre sessionerna. Frågeformulär är i pappersform eller elektroniska. Koordinatorn kommer att

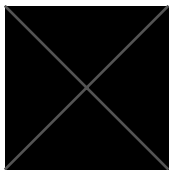

påminna deltagande vårdpersonal att skicka ut länkar (eller pappersformulär när så önskas). Huvudsökande och en doktorand/assistent kommer att samordna studierna, ge övning och handledning, analysera data och förbereda det första utkastet till manuskripten. Medsökande samt övriga forskare som deltagit i utvecklingen kommer att ingå i den efterföljande skrivprocessen.

6.2 Redogör för det statistiska underlaget för studiepopulationen/ undersökningsmaterialets storlek.

Studie 1: Det finns ingen konsensus kring antal deltagare för test-retest men vi går på förslaget att ha ungefär 5 gånger antalet items (Park et al., 2018) Med 33 frågor eftersträvar vi därför minst 150 deltagare.

Studie 2: Det är svårt att utvärdera vad som är en "kliniskt relevant" storlek på skillnad för respektive levnadsvana. Den ackumulerade effekten av ohälsosamma levnadsvanor över tid kan bli stor varför det är eftersträvansvärt att även kunna upptäcka små till medelstora effekter. Vi vill kunna upptäcka en en odds-ratio på ca 2 eller motsvarande en liten till medelstor effekt för Cohens d. För det eftersträvar vi ca 200 i kontrollgruppen och 200 i varje grupp med funktionsnedsättning. Skillnader i den storleken har observerats i tidigare studier för vuxna med autism för sömn (Croen et al., 2015) fysisk aktivitet (Hillier et al., 2020) och diet (Wier et al., 2021). För intellektuell funktionsnedsättning är skillnaderna till referensgrupp ännu större för fysisk aktivitet (Dario et al., 2016), diet (Gast et al., 2021).

Studie 3: De huvudsakliga analyserna i studie 3 gäller antal deltagare som genomför Lev samt nivå av acceptans av insatsen. I dessa analyser sker ingen jämförelse med kontrollgrupp men jämfört med tidigare genomförbarhetsstudier är n = 100 en relativt stor population. De de preliminära analyserna av effekter av interventionen där vi jämför med kontrollgrupp syftar till att ge underlag för powerberäkning inför evntuell RCT.

Studie 4: För de kvalitativa analyserna i studie 4 kommer vi inkludera ett relativt stor population jämfört med tidigare kvalitativa analyser och vi bör med god marginal kunna identifiera de vanligast förekommande teman för både personer med autism och mild intellektuell funktionsnedsättning.

6.3 Hur kommer undersökningsprocedurerna att dokumenteras?

Data samlas in via både via elektroniska och pappersenkäter. Under datainsamlingen på respektive klinik/center förvaras CRF och ifyllda enkäter inlåsta i ett journalskåp separerat från journaldata. Databas förvaras på ett usb-minne inlåst dels på den klinik/center där medarbetare som behöver tillgång till databasen arbetar, dels säkerhetskopia på Habilitering & Hälsa, Regions Stockholm och på KIND, Karolinska Institutet. Ingen obehörig kommer ta del av databasen.

6.4 Hur kommer insamlad data att hanteras och förvaras?

Data kommer att kodas när den matas in i databasen så att man inte kan identifiera någon enskild deltagare utifrån de uppgifter som finns där. Varje deltagare får ett nummer som kan kopplas till deltagaren genom kodnyckel (studie 3 och 4). Kodnyckeln förvaras separat från databasen på

Habilitering & Hälsa. Pappers och elektroniska data förvaras inlåsta på Habilitering & Hälsa. Data från studien gallras 10 år efter att studieresultaten publicerats.

7.1 Vilka risker kan ett deltagande medföra för de forskningspersoner som ingår i forskningsprojektet?

Frågorna och råden är av den karaktär som ges idag via vården och digitala plattformar så som 1177. Skillnaden är att dessa råd har justerats för att inte endast vara anpassade för en populationen utan funktionsnedsättning och att vi erbjuder ett utökad stöd i genomförande. Materialet har även pilottestats flera gånger och studie 3 utgör ytterligare ett tillfälle att undersöka om oönskade bieffekter (adverse events) uppstår. I utbildningen till Lev får behandlarna lära sig att inte skuldbelägga utan tvärt om validera och uppmärksamma tidigare och nuvarande ansträngningar. För att ta bort eventuella skuldkänslor och skam kring att man inte lever hälsosamt, ingår det i interventionen att både behandlare och deltagare får lära sig att levnadsvanor inte är egenskaper och att de flesta har några ohälsosamma vanor. Vi har bifogat manual för praktiskt genomförande så att granskare av denna ansökan ska kunna se konkreta exempel på hur vi vidtar åtgärder för att engagera, motivera samt öka förståelse var varför vi lever ohälsosamt.

Behandlarna får lära sig i utbildningen att betona möjligheten att må bättre snarare än risken att må sämre. I interventionen ingår det att sätta mål. Detta är i linje med de arbetssätt som används inom habilitering och även annan vård idag. Att inte nå sina mål kan resultera i att man känner sig besviken. Dock sätts dessa mål tillsammans med deltagaren och i utbildningen får behandlarna lära sig att sätta realistiska mål enligt SMART-principen. Betoningen ligger på att sätta mål som det är väldigt hög sannolikhet att deltagaren når för att bygga självförtroende inför kommande utmaningar. Dessutom ger interventionen, till skillnad från nuvarande rutin, ett ökat stöd för genomförande för att öka sannolikheten att målen nås.

Deltagande i studie 3 och 4 bygger på att deltagarna anmäler intresse och är motiverade att delta. Vidare screenas deltagaren för psykisk ohälsa innan de deltar och hänvisas till läkare/psykolog om de mår dåligt. Deltagandet är frivilligt och kan när som helst avbrytas. Efter frågorna (Lev-s) och i interventionsmaterialet (Lev) framgår det hur behandlaren ska kommunicera vid avhopp och att de ska utvärdera om det finns behov att ta kontakt med läkare/psykolog. Inför pilotstudien testades materialet. Då vissa deltagare kan uppleva vissa områden som om de privata informeras de noggrant om att de kan avstå att svara på frågor utan negativa konsekvenser. Deltagarna ges kontinuerlig möjlighet att uttrycka åsikter om studien och kan avbryta deltagande när de vill.

7.2 Vilken nytta kan ett deltagande medföra för de forskningspersoner som ingår i forskningsprojektet?

Personer med funktionsnedsättningar (exempelvis autism och intellektuell funktionsnedsättning) dör många år tidigare än personer utan funktionsnedsättning på grund av behandlingsbara levnadsvanerelaterade sjukdomar (Nationellt vårdprogram, Ohälsosamma levnadsvanor, 2022). Att förbättra levnadsvanor kan ge positiva effekter både på psykisk och fysisk hälsa. Interventionen följer Socialstyrelsens riktlinjer prevention och behandling av ohälsosamma levnadsvanor (2018) och det finns vetenskapligt stöd för vikten av var och en av de olika

levnadsvanorna som inkluderas i Lev. Även om Lev inte skulle ha positiva effekter på hälsan direkt kan det höja kunskap och bidra till större delaktighet och livskvalitet då Lev uppmuntrar aktivt deltagande i flera viktiga aktiviteter. Både socialstyrelsen (2018) och WHO (2020) betonar potentialen och vikten av att bli bättre på att ge stöd för hälsosammare levnadsvanor. Hos de deltagare som ingått i pilottestningen av lev har vi fått positiva indikationer på levnadsvanor. Även patientorganisationerna som deltagit i utvecklingen av Lev har uttryckt att detta är efterfrågat av deras patientgrupper. För att vi ska veta mer om Lev är säker, genomförbar och effektiv behövs de beskrivna vetenskapliga studierna.

7.3 Gör en värdering av förhållandet mellan riskerna och nyttan av projektet.

Fördelarna bedöms med god marginal överväga riskerna. Ökad kunskap kan förutom förbättringar av det aktuella interventionsmaterialet även leda till ökad kunskap om levnadsvanor hos personer med funktionsnedsättningar i allmänhet. Sökanden har även träffat patientrepresentanter för de större grupper med funktionsnedsättningar som deltar i studien för att säkerställa att interventionen och dess utformning är i linje med deras önskemål. Ett ytterligare motiv är att det saknas systematiska utvärderingar av interventioner för att adressera ohälsosamma levnadsvanor för personer med funktionsnedsättning. Genomförbarhetsstudien adresserar just om interventionen accepteras på ett tillfredställande sätt så att det finns underlag för en RCT och eventuell implementering. Ofta implementeras material av denna typ direkt vilket medför bristande evidens för genomförbarhet och effektivitet. Det finns emellertid alltid risk att deltagare reagerar negativt och detta måste utvärderas i dessa studier. För att minska risken för det har vi inkluderat en utbildning som beskriver hur behandlarna kan ge stöd och öka motivation även när den är låg. Vi har en screeningprocedur som gör att vi kan identifiera individer som mår för dåligt för att påbörja Lev. Vi har beskrivit i manualen för det praktiska genomförandet och inkluderar i utbildningen instruktioner för hur behandlarna ska följa upp deltagare som blir modfällda eller hoppar av. Vidare har vi eftersträvat att utveckla en kostnadseffektiv intervention som kan implementeras på flera håll inom svensk sjukvård. Insatsen är transdiagnostisk och kan ges av olika professioner med hälso- och sjukvårdsutbildning.

7.4 Beskriv hur projektet har utformats för att minimera riskerna för forskningspersonerna.

Interventionen förväntas inte ha några negativa konsekvenser på kort eller lång sikt utan tvärt om bidra till ökad psykisk och fysisk hälsa för den stora majoriteten av deltagarna. Hos de patientgrupper som inkluderas i studierna finns det dock risk för ökad psykisk ohälsa och vi har därför flera rutiner som ska minimera riskerna. Vi har screening där vi kan identifiera individer som bedöms må för dåligt för att göra Lev just nu. Dessa individer hänvisas till läkare eller psykolog vid behov. All personal som deltar i studien har en vårdutbildning och har vana att jobba med de inkluderade patientgrupperna på ett liknande sätt. Patienterna väljer om de vill delta och vi gör en bedömning om de är tillräckligt motiverade för att kunna nå sina mål. Utbildningen inkluderar beskrivning av hur man sätter attraktiva och rimliga mål för deltagren för att minska risken att man inte når dem. Vidare ges stöd flera gånger under insaten. För deltagare som avbryter eller mår sämre erbjuds stöd och de hänvisar till psykolog eller läkare. Genom att endast publicera anonymiserade data risker personerna inte att deras medverkan samt data kan kopplas till deras person. Vi är inte medvetna om andra vetenskapliga studier som skulle kunna ersätta de

studier som beskrivs i denna ansökan. Beträffande kontrollgrupp så kommer de utifall interventionen uppvisar goda resultat att på sikt erbjudas Lev. Nu får kontrollgruppen istället sedvanlig behandling.

7.5 Identifiera och precisera om eventuella etiska problem (nackdelar/fördelar) kan uppstå i ett vidare perspektiv genom forskningsprojektet.

Delstudierna möjliggör att på sikt kunna erbjuda evidensbaserade insatser för personer med funktionsnedsättning. Den möjliggör ökad kunskap om vilka levnadsvanor som är särskilt viktiga att uppmärksamma bland annat genom att deras relation till livskvalitet studeras. Det är redan känt att livslängden hos personer med ohälsosamma levnadsvanor medför runt 14 år tidigare död (Nationellt vårdprogram, Ohälsosamma levnadsvanor, 2022). Det finns redan flera rapporter och studier som pekar ut psykiska och fysiska funktionsnedsättningar som riskgrupper för sämre levnadsvanor. Riskerna är att forskningen möjliggör att man kan stigmatisera på basis av eventuell uppvisad korrelation mellan sämre levnadsvanor och funktionsnedsättning. Alla människor har rätt till god hälsa och det är därför viktigt att forskningen görs så att vi kan adresserar den ojämlika situation som råder idag. Att utveckla kunskapen kring hur vi ska adressera minskad livskvalitet, psykisk och fysisk ohälsa anser vi överväger riskerna.

8.1 Hur görs urvalet av forskningspersoner?

För delstudie 1 och 2 kommer majoriteten av deltagarna rekryteras via social medier eller banners på hemsidor hos intresseorganisationer för olika patientföreningar som med på att delta.

Deltagarna kommer även att rekryteras från Habilitering & Hälsa Regions Stockholm, LSS-hälsan i Stockholmsstad, samt två habiliteringar från andra regioner i Sverige. Det kan även blir relevant att rekrytera från primärvården. Forskningen avser personer över 18 år med eller utan funktionsnedsättning men för studie 2, 3 och 4 vill vi främst inkludera personer med psykisk och/eller fysisk funktionsnedsättning så som autism, intellektuell funktionsnedsättning, rörelsenedsättning, förvärvad hjärnskada, dövblindhet, döva. Samsjukliga diagnoser så som ADHD och depression kan förekomma. För deltagande i studie 3 och 4 krävs att behandlaren bedömer att deltagaren (genom screening) kan delta utan behov av medföljare/stöd från anhörig eller inte har en psykosocial situation som gör det svårt att delta i projektets. I detta projekt utesluts sålunda personer som är under 18 och personer med mer omfattande funktionsnedsättning. Dessa grupper har vi för avsikt att adressera i senare studier som vi inte inkluderar i den här ansökan. Vi bedömer att vi måste göra ytterligare anpassningar av Lev-materialet för att det ska vara genomförbart och effektivt för grupper som är i behov av medföljare.

För att komma i kontakt med lämpliga personer tillfrågar vi behandlare på sjukvårdsenheter inom Hälso- och sjukvård som anmält intresse eller som varit med och utvecklat interventionsmaterialet (Habilitering & Hälsa, Regions Stockholm, LSS-hälsan Stockholms stad, Regions Skåne). Dessa aktörer träffar deltagare med dessa funktionsnedsättningar. Behandlare som deltar i studien frågar patienter om de vill vara med i en forskningsstudie muntligen och de får skriftlig information om projektet att ta ställning till i lugn och ro. Sålunda ges deltagaren tid att tänka och ett eventuellt deltagande påbörjas fört nästkommande besök.

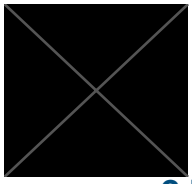

.2 Hur många forskningspersoner kommer att inkluderas i forskningsprojektet?

I delstudie 1 ingår ca 150. I delstudie 2 inkluderas 200 kontroller, 200 med autism och 200 med intellektuell funktionsnedsättning och 100 personer med antingen förvärvad hjärnskada, dövblindhet, döva eller rörelsenedsättning. I delstudie 3 och 4, 150 personer med funktionsnedsättning. Det exakta antalet är svårt att fastställa då förekomsten av individer och den utsträckning de har kontakt med vården under studien period är okänd.

8.3 Vilka urvalskriterier kommer att användas för inklusion?

I delstudie 1 behöver man vara över 18 och förstå svenska. Kontroller till delstudie 2 ska vara över 18, förstå svenska och inte ha en funktionsnedsättning. Övriga deltagare i studie 2 ska vara över 18 förstå och kunna skriva svenska, ha någon from av funktionsnedsättning eller diagnos. För delstudie 3 och 4 ska deltagarna vara över 18 år, förstå och skriva svenska, kunna delta utan medföljare, ha en diagnos eller funktionsnedsättning så som autism, intellektuell funktionsnedsättning, rörelsenedsättning, förvärvad hjärnskada, dövblindhet, vara döv. Psykiatrisk samsjuklighet samt farmakologisk behandling får förekomma.

8.4 Vilka urvalskriterier kommer att användas för exklusion?

Studie 1 och 2 har inga särskilda exklusionskriterier förutom att man behöver vara över 18 och förstå och skriva svenska. För delstudie 3 och 4 exkluderas personer som är minderåriga eller som är i behov av en medföljare. Psykisk eller psykosocial instabilitet som av behandlaren bedöms vara av den grad att deltagande inte är möjligt. Exempel på det kan vara svår depression och ångestsyndrom, missbruk och beroendesjukdomar, maniska tillstånd, psykos, förhöjd suicidrisk eller en annan svår livssituation (exempelvis hemlöshet).

8.5 Ange relationen mellan forskare och forskningspersonerna.

Forskaren kommer inte att träffa några av deltagarna. Tillsammans med ledningen på Habilitering & Hälsa Regions Stockholm tillfrågas behandlare på olika enheter om de vill delta i forskningsstudien. Det är sedan behandlarna som tillfrågar de patienter de träffar om de vill delta i studien.

8.6 Vilket försäkringsskydd finns för de forskningspersoner som deltar i forskningsprojektet?

Sedvanligt patientförsäkringsskydd föreligger deltagarna som deltar i studie 3 och 4. För de som deltar anonymt i delstudie 1 och 2 finns inget försäkringsskydd utöver det som individen själv har.

8.7 Redogör för den beredskap som finns för att hantera oväntade bifynd eller händelser under forskningsprocessen som kan äventyra forskningspersonernas säkerhet.

De deltagare som ingår i delstudie 3 och 4 kommer har en pågående kontakt med vårdpersonal och inom ramen för den kontakten finns det etablerade rutiner för att uppmärksamma ohälsa. Beträffande deltagande i delstudie 3 där en intervention ingår, finns det utöver sedvanliga rutiner,

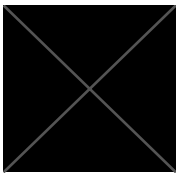

en behandlarutbildning för att kunna administrera Lev-materialet. I denna utbildning diskuteras rutiner för att kunna avbryta studien och hur behandlaren ska ta ställning till om deltagaren är i behov av annat stöd. Behandlarna i studien har en hälso-sjukvårdsutbildning och rutiner för hur samverkan med exempelvis psykiatrin ska gå till.

8.8 Kommer ekonomisk ersättning eller andra förmåner betalas ut till forskningspersonerna?

Nej

9.1 Kommer forskningspersonerna att informeras om forskningsprojektet och tillfrågas om de vill vara med eller inte?

Ja

9.1.1 [Om Ja 9.1] Hur, när (i vilket skede) och av vem informeras och tillfrågas forskningspersonerna?

Delstudie 1 och 2: Deltagare rekryteras främst via onlineplattformar med hjälp av KIs kommunikationsavdelning som erbjuder ett organiserat stöd för rekrytering till vetenskapliga studier. Via annonsmaterial tillfrågas deltagare om de vill delta i forskningsstudien. De informeras om att avsändaren är Habilitering & Hälsa, Regions Stockholm och att deltagande är frivilligt. Om de vill vara med /samtycker klickar de på en länk och fyller anonymt i formulären. För ett mindre delsamle i delstudie genomförs Lev-s som intervju ena gången. Då de inte har samma möjlighet till fullständig anonymitet inhämtas skriftligt samtycke. Dessa deltagare får ta del av annonsmaterial i väntrum och via förfrågan av behandlare. De informeras om att det är frivilligt och ges tid att överväga deltagande.

Delstudie 3 och 4: Tilltänkt forskningsperson tillfrågas av behandlare i samband med ett patientbesök. Information om studien kommer även finnas tillgängligt i väntrum på respektive enhet som deltar. Deltagare rekryteras även via intresseorganisationer. Om patienten är intresserad av att delta ges skriftlig information om studien. Patienten ombes läsa igenom information om studien i lugn och ro på egenhand efter besöket och sedan återkomma inom en vecka om de vill delta. Behandlaren svarar på eventuella frågor om studien. Skriftligt samtycke inhämtas när patienten tagit ställning.

9.2 Kommer barn under 18 år att ingå i forskningsprojektet?

Nej

9.3 Kommer forskningspersoner, vars mening på grund av sjukdom, psykisk störning, försvagat hälsotillstånd eller något annat liknande förhållande inte kan inhämtas, att ingå i forskningsprojektet?

Nej

10.1 Kommer projektet att begära ut uppgifter från ett befintligt register?

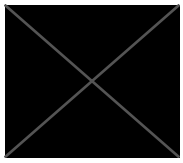

Nej

11.1 Finns det relevanta resultat från djurförsök?

Nej

11.1.2 [Om Nej 11.1] Ange anledningen till att djurförsök ej gjorts.

Djurstudier är inte applicerbart på forskningsfrågorna i någon av delstudierna.

12.1 Hur garanteras tillgång till data för forskningshuvudmannen och medverkande forskare?

Douglas Sjöwall, Tatja Hirvikoski, Elin Frögeli, Ulrika Långh och Sven Bölte samt eventuella andra forskare har direkttillgång till data . Vidare har samtliga behandlare, anställda vid de enheter som ingår som samarbetspartners, tillgång till data för enskilda individer eftersom detta kan behövas under pågående insamling.

12.2 Vem eller vilka ansvarar för databearbetning och skriftlig redovisning av resultaten?

Douglas Sjöwall, Tatja Hirvikoski, Elin Frögeli, Ulrika Långh Sven Bölte samt eventuella andra forskare är involverade i databearbetning, analys och rapportskrivning.

12.3 Hur och när planeras resultaten att offentliggöras?

Resultaten avses publiceras i en internationell vetenskaplig tidsskrift. Resultaten är av intresse även för en vidare grupp och en populärvetenskaplig beskrivning på svenska kan komma att framställas. Om screening och insats visar sig fungera, kan de på sikt spridas till andra verksamheter eller delar av landet. Metoden kommer då spridas bl.a. genom en manual, utbildning och föreläsningar.

12.4 På vilket sätt garanteras forskningspersonernas rätt till integritet när materialet offentliggörs?

Endast avidentifierade resultat på gruppnivå presenteras.

13.1 Redovisa eventuella ekonomiska överenskommelser med bidragsgivare eller andra finansiärer (namn och belopp).

Inte aktuellt.

13.2 Redovisa forskningshuvudmannens, huvudansvarig forskares och medverkande forskares egna ekonomiska intressen.

Varken huvudsökanden eller medverkande forskare har någon egen företagsverksamhet som gynnas av projektet.

Forskningsplan

Den sammanfattande beskrivningen av forskningsprojektet ska förstås av fackmän. Den kan lämpligen utformas enligt följande:
Vetenskaplig frågeställning: En redogörelse för det övergripande syftet med det föreslagna forskningsprojektet samt specifika mål (primära och sekundära frågeställningar).
Områdesöversikt: Ge ett sammandrag av egna och andras forskning och tidigare resultat inom forskningsområdet. Översikten ska tydliggöra det aktuella projektets relevans. Nyckelreferenser ska anges.
Projektbeskrivning: Gör en sammanfattning av projektets/motsvarande uppläggning. Urval av forskningspersoner, procedurer, metoder med mera ska tydligt redovisas. Det ska framgå hur metoder, urval och procedurer kan ge svar på de specifika frågeställningarna. Om flera delprojekt avses anges sekvens för genomförande och på vilket sätt ett efterföljande delprojekts uppläggning kan bero av resultaten av ett föregående.
Betydelse: Ge en kortfattad redogörelse för projektets betydelse för forskningsområdet.
Preliminära resultat: Kan i förekommande fall anges.
SKA VARA PÅ SVENSKA ELLER ENGELSKA.

Forskningsplan.docx.pdf
180.99KB

Annonsmaterial

Etikprövningsmyndigheten behöver alltid ta del av allt annonsmaterial som ska användas vid rekryteringen.
SKA VARA PÅ SVENSKA.

Annonsmaterial_delstudie_1_.docx.pdf
92.44KB

Annonsmaterial_delstudie_2.docx.pdf
97.19KB

Annonsmaterial_delstudie_3_och_4.docx.pdf
95.78KB

Annons_Facebook.docx.pdf
209.49KB

Information till forskningspersoner och samtyckesformulär

Etikprövningsmyndigheten behöver alltid ta del av all information som kommer att ges till forskningspersonen i samband med tillfrågan om deltagande. Både den information som ska ges muntligt och den som ska ges skriftligt. Om vårdnadshavare ska samtycka till deltagande ska även den information som ges till vårdnadshavarna bifogas. Om anhörig ska ges möjlighet att motsätta sig deltagande ska även den information som ges till anhörig bifogas.
Etikprövningsmyndigheten rekommenderar att vår stödmall för forskningspersonsinformation används, den hittar du på www.etikprovning.se
SKA VARA PÅ SVENSKA.

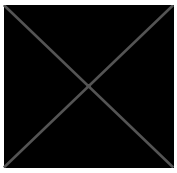

Forskningspersonsinformation_delstudie_1.docx.pdf
52.4KB

Forskningspersonsinformation_delstudie_3_och_4_.docx.pdf
63.58KB

Samtycke_delstudie_1.docx.pdf
26.49KB

Samtycke_delstudie_3_och_4.docx.pdf
36.4KB

Enkäter, frågeformulär, mm

Materialet ska vara utformat/skrivet på svenska.
SKA VARA PÅ SVENSKA.

Enkat_Nuvarande_livssituation_delstudie_1.docx.pdf
42.76KB

Enkat_Nuvarande_livssituation_delstudie_2.docx.pdf
42.61KB

Enkat_Nuvarande_livssituation_delstudie_3_och_4.docx.pdf
39.92KB

Enkat_Skattning_av_levnadsvanor_Lev-s.docx.pdf
176.1KB

Enkat_maluppfyllelse.docx.pdf
25.13KB

Enkat_TCS_behandlare.doc.pdf
58.92KB

Enkat_TCS_deltagare.doc.pdf
58.47KB

Enkat_Utvardering_session_deltagare.docx.pdf
45.8KB

Enkat_Utvardering_session_behandlare.docx.pdf
42.33KB

Enkat_Utvardering_helhet.docx.pdf
51.59KB

Enkat_WHOQOL-BREF.pdf
194.59KB

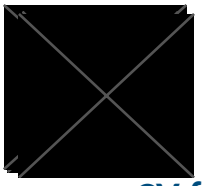

CV för ansvarig forskare

Bifoga CV för ansvarig forskare.
I undantagsfall kan icke disputerad forskare godtas om annan medverkande disputerad forskare uttalat att forskningen sker under aktivt överinseende av denne. Uttalandet intygas vid signering av disputerad forskare. CV för den disputerade ska även bifogas.
SKA VARA PÅ SVENSKA ELLER ENGELSKA.

CV_Douglas_Sjowall.docx.pdf
115.33KB

Övriga bilagor

BILAGOR SOM SKA BEAKTAS VID PRÖVNINGEN SKA VARA PÅ SVENSKA.

Screening_infor_deltagande_.docx.pdf
24.65KB

_Teoretisk_manual.docx.pdf
294.33KB

Manual_paraktiskt_genomforande.docx
622.42KB

Kompletterande bilageförteckning

Forskningsplan: Forskningsplan för fackmän
Annonsmaterial: Annonsmaterial för de olika delstudierna
Forskningspersoninfromation delstudie 1
Forskningspersoninfromation delstudie 3 och 4
Samtycke delstudie 1
Samtycke delstudie 3 och 4
Enkät Nuvarande livssituation: Bakgrundsinformation och frågor om hälsa för deltagare för de olika delstudierna
Enkät Skattning av levnadsvanor Lev-s
Enkät för måluppfyllelse
Enkät TCS behandlare: Mäter treatment credibility från behandlarens perspektiv
Enkät TCS deltagare: Mäter treatment credibility från deltagarens perspektiv
Enkät Utvärdering session deltagare: Mäter vad deltagaren tycker om de olika sessionerna av Lev
Enkät Utvärdering session behandlare: Mäter vad behandlaren tycker om att utföra de olika sessionerna i Lev
Enkät Utvärdering helhet: Mäter vad deltagaren tyckte om behandlingen som helhet
Enkät WHOQOL-BREF: Mäter livskvalitet och välbefinnande
CV ansvarig forskare
Screening inför deltagande: Används för att se om deltagaren inte uppfyller exklusionskriterier
Teoretisk manual för utveckling av Lev: Exakta formuleringar kan komma att ändras
Manual för praktisk genomförande av Lev: Exakta formuleringar kan komma att ändras

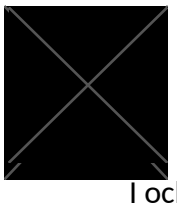

I och med att ansökan undertecknas intygar du som är ansvarig forskare samt du som är behörig företrädare följande;

- Att den information som lämnas i ansökan om etikprövning och samtliga medföljande bilagor är riktig och fullständig.
- Att verksamhetsansvariga i samtliga medverkande verksamheter är informerade om forskningsprojektets innehåll och utförande och att de har samtyckt till att delta i studien.
- Att du säkerställt att det i samtliga medverkande verksamheter finns resurser som garanterar forskningspersonernas säkerhet och integritet vid genomförandet av den forskning som beskrivs i ansökan.
- Att ansvarig forskare ges rätt att företräda huvudmannen i alla framtida kontakter med Etikprövningsmyndigheten som rör detta forskningsprojekt samt ansöka om ändringar i forskningsprojektet.
- Att du tagit del av Etikprövningsmyndighetens information om hantering av personuppgifter på myndighetens webbplats.

Behörig företrädare för forskningshuvudmannen

Joakim Lavesson

Signatur behörig företrädare

Signatur-behorig-foretradare.pdf
32.19KB

Är behörig företrädare ordinarie eller tillförordnad?

Ordinarie företrädare

Signatur huvudansvarig forskare

Signatur-huvudansvarig-forskare.pdf
30.57KB

Signatur-huvudansvarig-forskare.pdf
31.69KB

Signatur-huvudansvarig-forskare.pdf
31.46KB

Signatur-huvudansvarig-forskare.pdf
31.58KB

Kompletterande bilagor

Forskningspersonsinformation_delstudie_1_online_version_2_220708.docx.pdf
51.78KB

Forskningspersonsinformation_delstudie_1_via_klinik_version_2_220708.docx.pdf
40.22KB

Forskningspersonsinformation_delstudie_2_version_2_220708.docx.pdf
38.15KB

Forskningspersonsinformation_delstudie_3_och_4_version_2_220708.docx.pdf
55.17KB

Svar på de frågor eller synpunkter som Etikprövningsmyndigheten angett i sitt beslut, samt beskrivning av de ändringar som gjorts

Jag har nu kompletterat ansökan med forskningspersonsinformation till behandlare. Samtycke för behandlare är samma som patienter/deltagare för studie 3 och 4. Sålunda bifogas inget separat samtycke.

Kompletterande bilagor

Annonsmaterial_delstudie_1_version_2_220707.docx.pdf
153.84KB

Annonsmaterial_delstudie_2_version_2_220707.docx.pdf
154.15KB

Annonsmaterial_delstudie_3_och_4_version_2_220707.docx.pdf
162.15KB

Lev_paraktiskt_genomforande_220707.docx.pdf
567.22KB

Forskningspersonsinformation_behandlare_delstudie_3_och_4_version_220908.docx.pdf
55.56KB

Beslut och handlingar från Etikprövningsmyndigheten

Beslutsbrev och andra handlingar från Etikprövningsmyndigheten i relation till denna ansökan

2022-02920-01_Avgiftsavisering.pdf
41.36KB

2022-02920-01_Ansokan_Komplettering_till_sammantrade.pdf
45.14KB

2022-02920-01_Begaran_om_administrativt_tillagg.pdf
39.58KB

2022-02920-01_Ansokan_komplettering_till_ordforande.pdf
43.58KB

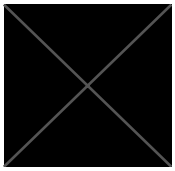

2022-02920-01_Ansokan_Godkand_med_villkor.pdf
44.64KB

Scientific research question

Aim
This project aims to generate knowledge and methods to counteract unhealthy living habits in people with disabilities. Evaluation of a screening tool (Lev-s) and a manualized intervention (Lev) is one focus, but the purpose is also to generate new knowledge about disabilities and health-related habits.

Research questions:
Study 1: What is the reliability and validity of Lev-s?

Study 2: What do health-related habits look like in individuals with and without disabilities? What is the relation to quality of life for the different habits? What habits are perceived as most important? What is the perception of available support for the different habits? What is the perception of how the disability limits pursuing health habits?

Study 3: Is Lev feasible transdiagnostically and for healthcare workers from different professions? How can Lev be developed based on feedback from participants and healthcare workers? What is the preliminary effect on living habits, and quality of life and individual goal attainment?

Study 4: How are healthy and unhealthy lifestyles created? Can we find patterns in the applied behavioral analyses done during the intervention and do they differ depending on type av disability or demographic variables?

Etikprövningsmyndigheten
2022-02920-01-269952
2022-05-23

Background

Unhealthy living habits have been recognized as a major cause of physical and mental ill-health and premature death (WHO, 2020). The heterogeneity is large between and within disabilities but the overall picture of unhealthier living habits in individuals with disabilities is very clear (WHO, 2020). Also, applicants in this proposal have shown that individuals with disabilities in Sweden, have a higher risk of premature death due to causes related to treatable unhealthy livening habits (Hirvikoski et al., 2016; Hirvikoski et al., 2021). Studies show that efforts to target health fail to reach and are not adjusted for people with enduring disabilities (e.g., Flyckt, 2020; Nationellt vårdprogram, 2022; Socialstyrelsen, 2018). Recent studies point to even less access to healthcare, decreased physical activity, changes in social habits, fewer activities, and stress among individuals with disabilities (Courtenay et al 2020; Lebrasseur et al 2021; Oomen et al., 2021). According to WHO, targeting lifestyle and habits can effectively prevent both mental and physical ill-health (WHO, 2020). Current efforts to

promote healthier habits are largely based on providing information and have not been systematically evaluated regarding feasibility and effectiveness (Socialstyrelsen, 2014). One key problem is that current efforts are often designed from the perspective of one healthcare profession, for one diagnostic group, and include only one or a few living habits. There are no methods to measure and intervene that are effective, feasible for several different risk groups (e.g., physical, mental and cognitive disabilities), that can be widely disseminated. Lev offers one procedure that can be used for several health-related habits. Lev have been developed in collaboration with researchers, healthcare workers from different professions, and patient organizations over two years. This has created a multifaceted perspective and very thorough development. During this work, we have found synergetic effects in trans-sectorial co-operation and designed a research project with high feasibility (i.e. high probability to succeed).

Theoretical background in short

- Lev implements national guidelines (Socialstyrelsen, 2018; Nationellt vårdprogram, 2022).
- The development of Lev has followed theoretical guidelines for how to develop healthcare interventions (e.g., O'Cathain et al 2019).
- Lev follows a stepped care model starting first with less demanding and more universal support. Lev includes instruction for how to coordinate more specialized support within all 10 habits.
- Lev departs from a biopsychosocial approach (WHO, 2011) meaning that we focus on the need of the individual and how we can reduce obstacles in the surrounding environment.
- This research project has include a focus on addressing interprofessional collaborative practice (IPCP). The goal of IPCP is to foster care collaboration that optimizes patient outcomes.

Etikprövningsmyndigheten
2022-02920-01-269952
2022-05-23

Project description

Lev-s and Lev

The screening tool Lev-s covers ten health-related habits with a total of 33 questions regarding sleep, nutrition, screen time, physical activity, friends, sexual health, hobbies, and consumption of alcohol, tobacco, and illegal drugs and takes 20-25 minutes to administer.

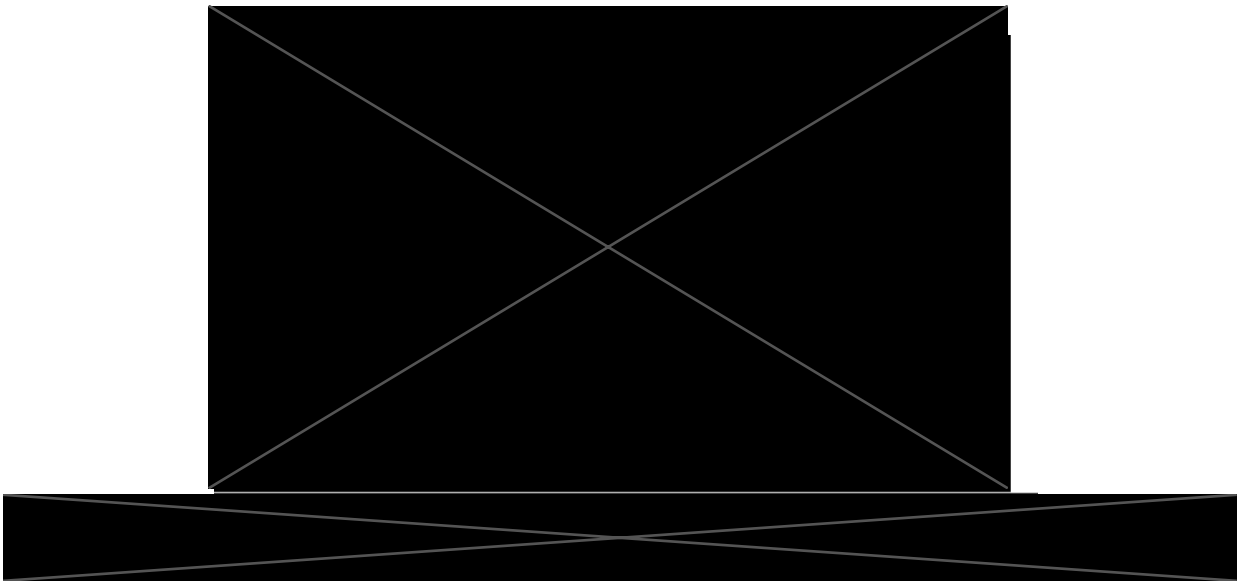

Lev includes three sessions using psychoeducation, motivational interviewing, and applied behavioral analyses. The second session can be repeated if needed. Lev-s and Lev are described in two manuals (se attatchments) that are used to ensure high treatment fidelity. In this research proposal, all included staff will receive education from the main applicant together with experienced clinicians and educators. The educational modules have been pilot tested through Microsoft Office Teams and will be delivered online.

Etikprövningsmyndigheten
2022-02920-01-269952
2022-05-23

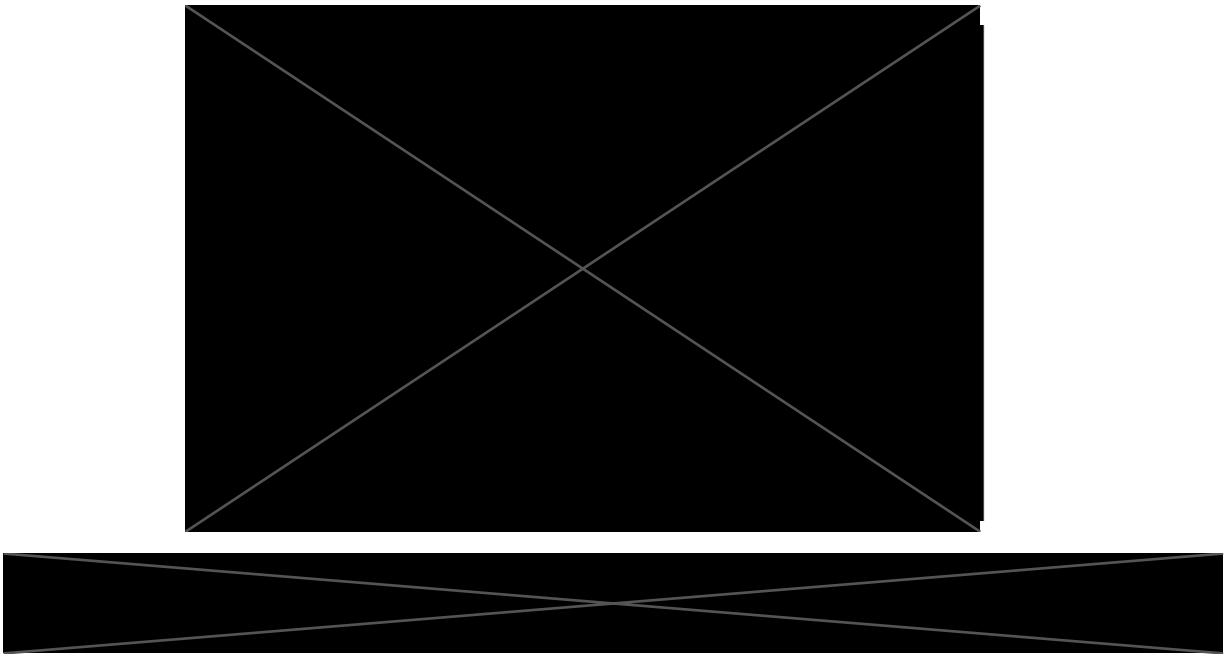

Timeline

We have conducted pilot studies and Lev-s has been evaluated in a master thesis (Wester at al., 2021) at the Karolinska Institutet. The next step is to conduct a scientific peer-reviewed evaluation to come one step closer to making Lev-s and Lev evidence-based methods. Studies 1 and 2 will begin in the fall 2022 and go on during 2023. Studies 3 and 4 will begin in January 2023 and go on during 2023.

Study 1: Psychometric properties of Lev-s: A screening tool for health-related habits.

Study 1 includes a minimum of 150 participants with and without disabilities. Data will be collected by filling out assessments online, in video meetings or at clinics in relation to the regular clinical practice. Participants may have several disabilities including physical ones.

a) What is the test-retest reliability of Lev-s self-assessments?

Reliability will be investigated by test-retest of the self-assessment version of Lev-s. The participants (n=150) will complete Lev-s at two time points 2 weeks apart to investigate to what extent they provide the same answers. A high correlation between the two time points is indicative of higher reliability.

b) What is the agreement between the self-assessments and assessments made by the healthcare worker for Lev-s?

A sub-sample will complete Lev-s first as an interview and then as a self-assessment. The interview version of Lev-s is the same but has the advantage of having the healthcare worker present to provide further explanation if participants need clarifications when needed.

c) To what extent are health-related habits correlated with quality of life?

All participants fill out current life situation form (Nuvarande livssituation), WHOQOL-BREF at one of the timepoints. WHOQOL-BREF includes four domains: physical health, psychological health, social relations, and environment. We will investigate the correlation between these four domains and the ten different health-related habits in Lev-s.

Etikprövningsmyndigheten
2022-02920-01-269952
2022-05-23

Study 2: Health-related habits in adults with and without disabilities.

We strive to include 200 controls (without disabilities) and 200 for each group of disabilities (autism, intellectual disability, acquired brain damage, movement disorder, deaf-blindness, deaf). Participants may have comorbid neuropsychological disorders (e.g., attention deficit hyperactivity disorder) or other mental health problems. It is hard to predict how many we will reach and that agree to participate from each group of disabilities. Recruitment will be done both through platforms at KI, Habilitations, and patient interest organizations.

a) How do health-related habits differ between different disabilities and in comparison to people without disabilities?

Comparisons will be done both between different disabilities and to the control group.

b) Is there an effect of demographic factors (e.g., sex, age, SES) on livening habits in individuals with disabilities?

The main effects of demographic factors, as well as interaction effects (demographic factor x group), will be investigated using regression analyses. Group will be investigated as the type of disability.

c) What habits are perceived as most important?
Participants with and without disabilities are asked to rate what they perceive as the three habits that are most important for their well-being.

d) What is the perception of available support for the different habits?
In relation to the habits participants list in question c, what is their perception of the support they receive?

e) What is the perception of how the disability limits pursuing healthier habits?
In relation to question c, what is the perception of how the participants are limited in pursuing the habits they want?

Study 3: A transdiagnostic assessment and intervention for health-related habits (Lev): An open controlled feasibility trial.

This study will include participants recruited from Habilitation & Health, Region Stockholm and it will also be open for other Habilitations and healthcare services in Sweden (total n=100). We will use the sample from studies 1 and 2 as controls. The study will be registered at clinicaltrials.org and follow the CONSORT checklist for feasibility studies 2010. All participants will fill out current life situation form (Nuvarande livssituation). The case report form includes demographic information. Participants may have several disabilities including physical ones. We will not include individuals with a more severe intellectual disability, mental or psychosocial instability to a degree that made participation impossible as judged by the health care professionals (i.e., severe psychiatric comorbidity such as ongoing substance use disorder, manic episodes, psychosis, and acute suicidality).

a) Eligibility to participate – how many of the healthcare workers that had received training in Lev started using it within 6 months? How many participants that were offered Lev agreed to take part in the intervention?

Participants will be entered in a standard flowchart to show how many patients that take part in the intervention. Healthcare workers taking part in the education will be asked after 6 months if they started using Lev.

b) What were the reasons for not taking part or not completing Lev?
Healthcare workers administering Lev will ask the declining participants for reasons to not take part in the intervention. These will be entered in the flowchart. We will also follow all healthcare workers that received education to administer Lev but that did not provide

Etikprövningsmyndigheten
2022-02920-01-269952
2022-05-23

treatments and ask to provide reasons to why. A thematic analysis of the themes will be carried out (Braun et al., 2004).

c) How many sessions and homework assignments did the participants complete?

Lev includes Lev-s, three sessions and two pre-specified home assignments. The administrating healthcare worker fills in completed sessions and assignments in the case report form. We will set the time limit to 6 months to complete the three sessions including all homework assignments.

d) Where are there any differences between healthcare professions regarding how the applied behavioral analysis was performed (e.g., were there any differences regarding strategies suggested to the participant)?

During session 2, a functional behavioral analysis is performed to generate strategies to help the participant reach their goal. We will conduct a thematic analysis to identify themes for each healthcare profession administrating Lev. This will answer questions regarding interprofessional collaborative practice and if it is possible to conduct this type of analysis in Lev or if further adjustments are needed.

Etikprövningsmyndigheten
2022-02920-01-269952
2022-05-23

e) Level of acceptability: Was Lev perceived as creditable and satisfactory by healthcare workers and participants?

Treatment credibility will be measured before Lev and after the last session for both participants and healthcare workers using a modified version of the treatment credibility scale (Borkovec et al., 1971). Treatment satisfaction will be evaluated after each session using a modified version of the Evaluation Questionnaire (Bramham et al., 2009) and after the intervention is completed (Patient Evaluation Questionnaire: Hesslinher et al, 2002; Hirvikoski et al., 2011). Session evaluations will be done by both participants and healthcare workers. Quantitative data will be presented in graphs and staple diagrams. Paired t-tests will be used to test pre-post differences for the treatment credibility measures and level of satisfaction will be compared between the three sessions.

f) Was there any part of Lev that was perceived less satisfactory by participants and healthcare workers?

A thematic analysis (Braun et al., 2004) will be performed to extract what was perceived as useful and to identify areas of improvement. This will be done both for participants and the healthcare workers using the open questions in the session and patient evaluation forms.

g) Do the intervention lead to adverse events?

Adverse events will be defined as spontaneous oral complaints or instances when patients stated that they experienced negative or unwanted effects during the intervention period. Serious adverse events will be defined as events that involved hospital care/hospitalization, etc. due to the intervention. Adverse events and serious adverse events will be recorded in the case report form, and it will be assessed by the administrating healthcare worker if these were caused by Lev.

h) Do Lev lead to healthier habits?

The Lev-s will be used to measures living habits.

i) To what extent were individual goals met?

During session 1 in the intervention, individual goals are set. The healthcare worker assesses to what extent the goals have been met using the goal attainment scale Ruble et al, 2012.

j) Did Lev lead to increased quality of life?

The intervention group fill in WHOQOL-BREF at baseline and follow-up. WHOQOL includes four domains: physical health, psychological health, social relations, and environment. Again, we will conduct both per-protocol as well as intention-to-treat analyses. Repeated measures ANOVA will be used to analyze data.

Etikprövningsmyndigheten
2022-02920-01-269952
2022-05-23

Study 4: A functional roadmap to healthier habits: A thematic analysis of themes form the functional analysis of unhealthy and healthy lifestyle behavior in adults with disabilities

This study will use data from study 3.

a) What general themes underlying unhealthy and healthy habits can be found in individuals with disabilities?

Healthcare workers are instructed to enter the identified *situations*, *behaviors*, and *short-term consequences* that unhealthy habits have for the participant. The same thing is done for healthy/desirable habits. They also enter what strategies they gave to the participant. A qualitative, thematic analysis will be performed to identify more general themes (Braun et al., 2014).

b) How do themes vary for specific living habits?

The thematic analysis will also be done separately for the different living habits.

c) How do themes vary for specific disabilities?

The thematic analysis will also be done separately for the different disabilities. Similarities and differences between the groups will be explored.

d) How do themes for questions b and c vary depending on sex?

The thematic analysis will investigate if there are differences between males and females.

The findings from study 4 will affect the adjustments of Lev.

Significance

Etikprövningsmyndigheten
2022-02920-01-269952
2022-05-23

Study 1: There are currently no scientifically evaluated screening tool that covers so many health-related habits and that can be used for different diagnostic groups. Lev-s enables comparison of several habits within one assessment and can therefore provide insight both clinically and scientifically.

Study 2: Currently, the scientific literature is limited to focusing on one or a few habits and one diagnostic group at a time. This study enables comparison of several lifestyle domains between different disabilities as well as controls without disabilities. It can identify where the need for resources is the greatest, which is lacking in both Swedish and international contexts currently.

Study 3: There is a lack of feasible and effective interventions to counteract unhealthy lifestyles. This intervention is based on one procedure/method that can be used to address several different health-related habits, transdiagnostically and by multiple healthcare professions. Lev thus provides a feasible intervention to disseminate as compared to implementing health-related habit, disability, and profession specific interventions. This could in turn improve mental and physical health for individuals with disabilities who currently suffer and die prematurely because of a healthcare system that is not aware or adjusted for their needs. Lev is designed to be used as a first step in a stepped care process. It also includes description for how to follow-up and collaborate with other parts of the healthcare system that offer basic and more specialized care. The study is relevant both for the further development of Lev and for others who want to develop similar interventions. The study also identifies opportunities and obstacles to collaboration between different care professions. This is an area which is often described as an important future avenue but that has few scientific publications.

Study 4: The study maps the reasons behind lifestyle behaviors and will provide knowledge for how we can reduce the obstacles hindering participation in areas that increase, health, quality of life and well-being. We are not aware of any previous study using this approach to use the data from the applied behavioral analysis and thematize to show patterns that could be a key in preventing and treating unhealthy habits.

A strength of this project has been the involvement of different stakeholders. We have included healthcare professionals from different healthcare organizations (Habilitering & Hälsa, Beroendecentrum Stockholm, Sluta-Röka-Linjen, Akademiskt primärvårdscentrum, Centrum för epidemiologi och samhällsmedicin-CES, Socialstyrelsen, Nationellt kunskapscenter för dövblindfrågor (NKCDB), SLSOs ungdomsmottagning, and Stockholms mansmottagning), representatives for patients and their networks (Hjärnkraft, Autism/Aspegerföreningen Stockholm, Rörelsehindrade barn och ungdomar – RBU Stockholm, and FUB Stockholm) and researchers and expert clinicians.

Preliminary results

Etikprövningsmyndigheten
2022-02920-01-269952
2022-05-23

Patient representatives
We have conducted semi structed interviews with patient representatives and identified reoccurring themes. The themes surround *the need for ongoing support* (not just information but a person-centered ongoing dialog), *inspiration* (raw models and support from individuals with the same condition), *cooperation between different parts of the healthcare system*, (there is a risk of losing the contact when the patient must explain themselves to several caregivers who often have limited knowledge about their condition), *including the network around the patient*. Also, in all interviews, Covid-19 was said to have produced more isolation and less opportunity to do activities and physical activities. We have put great effort into meeting these themes when designing the intervention.

Master thesis
The reliability of Lev-s was high (Inter-rater reliability, ICC = 0.98-0.99) and the administration has been perceived as highly feasible in a master thesis (Wester et al 2021). A vast majority of the participants stated that they would use Lev-s again and that they would recommend it to a colleague. The master thesis also identified areas of improvement that have led to adjustments.

Pilot study
Both Lev-s and Lev have been pilot tested at two habilitation centers. Both healthcare workers and patients have expressed that Lev-s provides a pedagogic overview that raises the awareness of habits and aspects in these habits that are of importance. The option to work will many different habits have resulted in that all participants have found some area that interests and motivates them. The pilot has also led to some changes. One important change has been that we express more clearly that the participant can decline to talk or answer questions in areas that they found private.

References

Borkovec et al (1971). Credibility of Analogue Therapy Rationales. J Behav Ther & Exp Psychiat 3:257-260.

Braham, J. et al. (2009). Evaluation of group cognitive behavioral therapy for adults with ADHD. *Journal of Attention Disorders*, 12, 434-41.

Braun et al (2006). Using thematic analysis in psychology. Qualitative Research in Psychology, 3(2), 77–101.

Courtenay (2020). COVID-19: challenges for people with intellectual disability. BMJ. 369:m1609.

Flyckt (2020). Insatser och stöd till personer med funktionsnedsättning (No. 2020-3–6686; p. 108). Socialstyrelsen.

Hesslinger, et al. (2002). Psychotherapy of attention deficit hyperactivity disorder in adults-a pilot study using a structured skills training program. *European archives of psychiatry and clinical neuroscience*, 252, 177-184.

Hirvikoski et al (2016) Premature mortality in autism spectrum disorder. Br J Psychiatry. 208(3):232-8.

Hirvikoski et al (2021). Association of Intellectual Disability With All-Cause and Cause-Specific Mortality in Sweden. *JAMA Netw Open*. 4(6):e2113014.

Hirvikoski, et al. (2011). Reduced ADHD symtoms in adults with ADHD after structuredskills training group: results from a randomized controlled trial. *Behaviour research and therapy*, 49, 175-185. doi: 10.1016/j.brat.2011.01.001

Lebrasseur et al (2021). Impact of COVID-19 on people with physical disabilities: A rapid review. Disabil Health J.

Nationellt system för kunskapsstyrning Hälso- och Sjukvård – Sveriges Regioner i Samverkan (2022). Nationellt vårdprogram ohälsosamma levnadsvanor – Prevention och behandling. Retrieved from https://vardgivarguiden.se/kunskapsstod/halsoframjande-arbete/regionalt-vardprogram-ohalsosamma-levnadsvanor/

O'Cathain et al (2019). Guidance on how to develop complex interventions to improve health and healthcare. BMJ Open

Etikprövningsmyndigheten
2022-02920-01-269952
2022-05-23

Oomen et al (2021). The psychological impact of the COVID-19 pandemic on adults with autism: a survey study across three countries. Molecular Autism 12, 21.

Ruble et al (2012). Goal attainment scaling as an outcome measure in randomized controlled trials of psychosocial interventions in autism. Journal of Autism and Developmental Disorders, 42, 1–10.

Skevington et al: WHOQOL Group (2004) The World Health Organization's WHOQOL-BREF quality of life assessment: psychometric properties and results of the international field trial. A report from the WHOQOL group. Qual Life Res. 2004 Mar;13(2):299-310.

Socialstyrelsen (2018). Nationella riktlinjer för prevention och behandling vid ohälsosamma levnadsvanor (No. 2018-6–24).

Socialstyrelsen (2014). Nationella riktlinjer – Utvärdering 2014 – Sjukdomsförebyggande metoder. Rekommendationer, bedömningar och sammanfattning. Retrieved from https://www.socialstyrelsen.se/globalassets/sharepointdokument/artikelkatalog/nationella-riktlinjer/2015-1-11.pdf

Wester et al (2021). Development of the Lifestyle habits Evaluation. A new instrument which primarily aims to assess the lifestyle habits of patients with functional disabilities. Master thesis.

World Health Organization. (2020). Disability and health. Disability and Health. Retrieved from https://www.who.int/news-room/fact-sheets/detail/disability-and-healt

World report on disability, 2011. World Health Organization. Available at: www.who.int. Accessed June 2021. World Health Organization Guidelines 2013: Transforming and scaling up health professionals' education and training. Geneva, WHO, 2013. Retrieved from https://apps.who.int/iris/bitstream/handle/

Etikprövningsmyndigheten
2022-02920-01-269952
2022-05-23

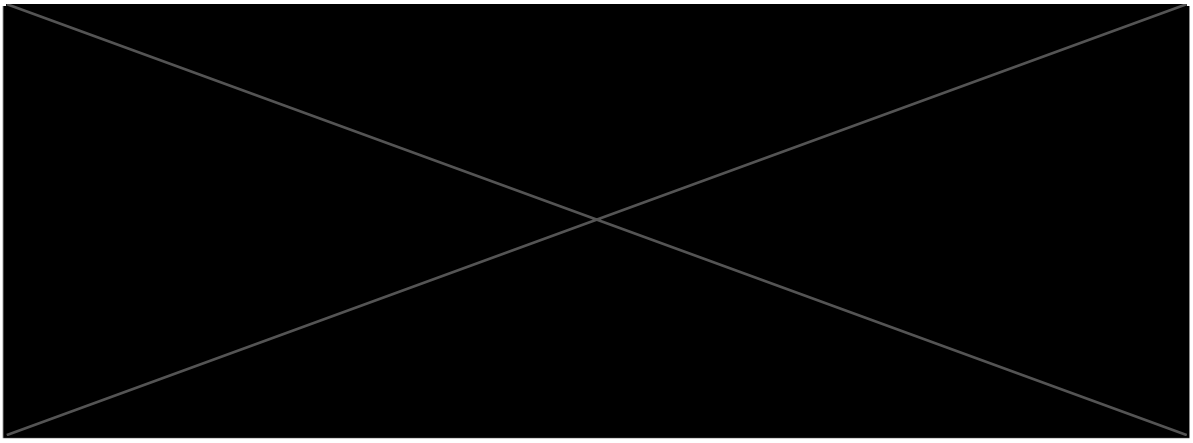

Etikprövningsmyndigheten
2022-02830-01-269976
2022-05-18

Vill du vara med i ett projekt som syftar till att mäta levnadsvanor?

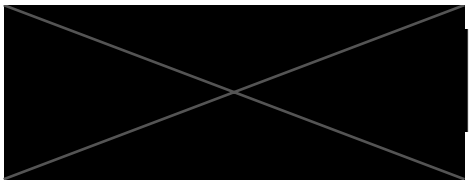

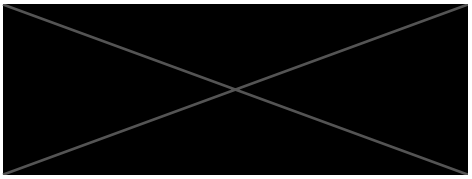

Ohälsosamma levnadsvanor leder enligt Världshälsoorganisationen (WHO) till psykisk och fysisk ohälsa. För att du, på egen hand eller tillsammans med hälso- och sjukvård, ska kunna må bättre är ett viktigt steg att kunna mäta hur dina levnadsvanor ser ut. I det här projektet utvärderar vi ett nytt frågeformulär som ska göra det enklare att mäta levnadsvanor (Lev-s). Vi behöver nu din hjälp att undersöka om frågeformuläret fungerar som det ska.

Hur går projektet till?

Om du deltar kommer du få svara på frågor vid 2 tillfällen via en länk (se nedan).

- Vid första tillfället fyller du i tre frågeformulär: ett om levnadsvanor (Lev-s), ett om livskvalitet (WHOQOL) och ett om bakgrundsformation om dig som deltar. Du deltar anonymt så vi kommer inte kunna koppla dina svar till dig.
- Efter 2 veckor fyller du i formulären igen. Det är ett sätt att testa hur säkert formuläret är. Om du inte ändrat dina levnadsvanor ska resultatet vara ungefär samma som gången innan.

Etikprövningsmyndigheten
2022-02830-01-269976
2022-05-18

Vem kan delta?

- Du behöver vara över 18 år
- Förstå svenska så att du kan förstå och svara på frågorna

Övrigt

Deltagande i projektet är helt frivilligt och du kan när som helst av vilken anledning som helst avbryta deltagande. Forskningshuvudman för projektet är Habilitering & Hälsa, Region Stockholm. Med forskningshuvudman menas den organisation som är ansvarig för projektet. Ansökan är godkänd av Etikprövningsmyndigheten, diarienummer för prövningen hos Etikprövningsmyndigheten är ange diarienummer

Vill du vara med?

Nu skriver du i din kalender, två veckor från idag, att du ska klicka på länken och fylla i frågorna igen. Det behöver inte vara exakt på dagen men det är jätteviktigt att du fyller i alla frågor en gång till.

Klicka på följande länk om du vill delta xxx.

Om du har frågor om projektet går det bra att vända sig till ansvarig för projektet är Douglas Sjöwall, forskare verksam vid Habilitering & Hälsa samt Karolinska Institutet. Douglas.sjowall@regionstockholm.se Habilitering & Hälsa, Box 454 36, 104 31 Stockholm 08-123 350 10.

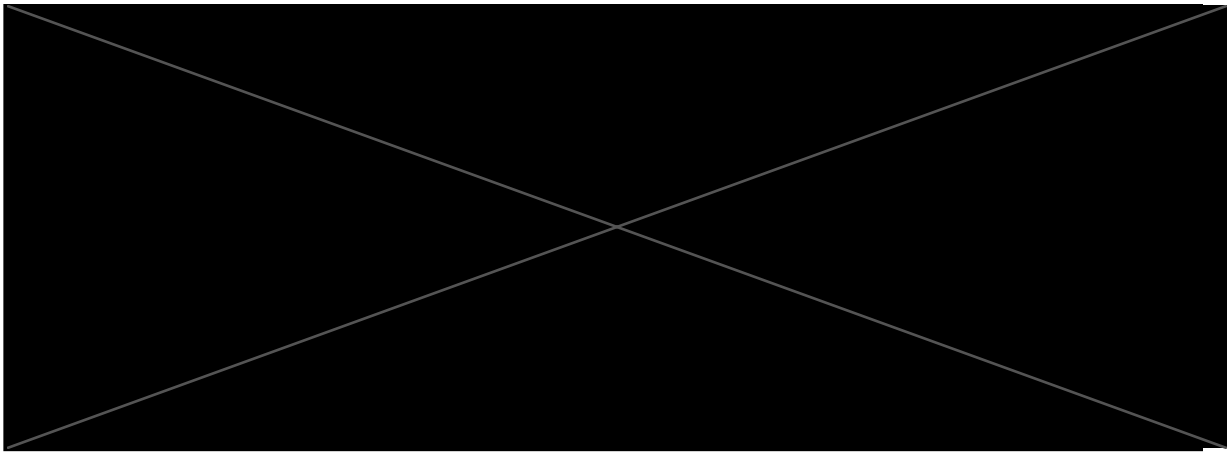

Etikprövningsmyndigheten
2022-02830-01-269977
2022-05-18

Vill du vara med i en forskningsstudie som undersöker levnadsvanor hos vuxna med och utan funktionsnedsättning?

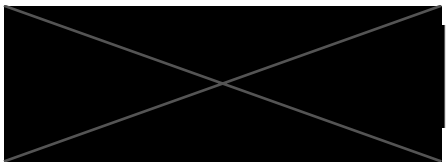

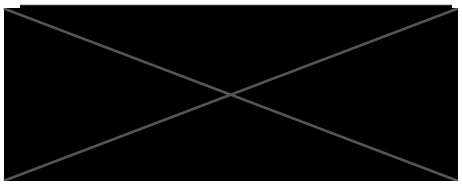

Ohälsosamma levnadsvanor leder till psykisk och fysisk ohälsa och även förtidig död enligt Världshälsoorganisationen (WHO). I det här projektet vill vi lära oss mer om levnadsvanor för att vi ska kunna utveckla bättre behandling och stöd. För det behöver vi veta mer om hur dina levnadsvanor ser ut, vilka levnadsvanor du tycker är viktigast, vilket stöd upplever att du kan få för att ändra dina levnadsvanor samt hinder du tycker finns.

Det är jätteviktigt att vi får in svar från människor med och utan diagnoser och funktionsnedsättningar så att vi kan utveckla stöd som passar alla.

Hur går projektet till?

Om du deltar kommer du få svara på frågor via en länk (se nedan).

- Du fyller du i tre frågeformulär: ett om levnadsvanor (Lev-s), ett om livskvalitet (WHOQOL) och ett om bakgrundsinformation. Du deltar anonymt så vi kommer inte kunna koppla dina svar till dig.

Etikprövningsmyndigheten
2022-02830-01-269977
2022-05-18

Vem kan delta?

- Du behöver vara över 18 år
- Förstå svenska så att du kan förstå och svara på frågorna

Övrigt

Deltagande i projektet är helt frivilligt och du kan när som helst av vilken anledning som helst avbryta deltagande. Forskningshuvudman för projektet är Habilitering & Hälsa, Region Stockholm. Med forskningshuvudman menas den organisation som är ansvarig för projektet. Ansökan är godkänd av Etikprövningsmyndigheten, diarienummer för prövningen hos Etikprövningsmyndigheten är ange diarienummer

Vill du vara med?

Klicka på följande länk om du vill delta xxx.

Om du har frågor om projektet går det bra att vända sig till ansvarig för projektet är Douglas Sjöwall, forskare verksam vid Habilitering & Hälsa samt Karolinska Institutet. Douglas.sjowall@regionstockholm.se Habilitering & Hälsa, Box 454 36, 104 31 Stockholm 08-123 350 10.

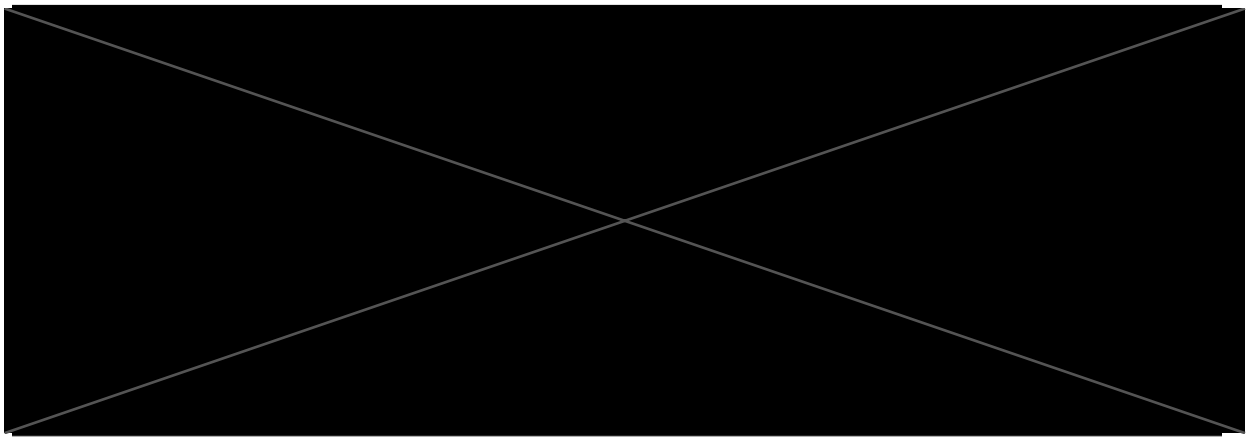

Etikprövningsmyndigheten
2022-02830-01-269979
2022-05-18

Vill du delta i en insats som syftar till att förbättra din psykiska och fysiska hälsa genom ändrade levnadsvanor?

Läs mer på nästa sida.

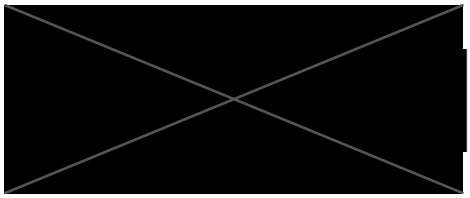

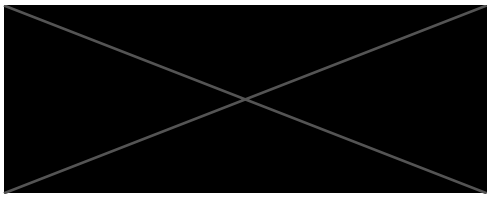

Vill du delta i ett projekt om syftar till att förbättra levnadsvanor och samtidigt bidra till forskning och utveckling?

Personer med funktionsnedsättning har sämre fysisk och psykisk hälsa och dör i förtid på grund av sämre levnadsvanor enligt Världshälsoorganisationen (WHO). Hälso- och sjukvård behöver bli bättre på att ge stöd och insatser som syftar till hälsosammare levnadsvanor hos personer med någon form av funktionsnedsättning. I det här projektet utvärderar vi en ny insats (Lev) som har som syfte att förbättra levnadsvanor. Nu är det viktigt att vi får ditt perspektiv på det är att delta i denna behandling så att vi kan fortsätta att utveckla och bättra.

Etikprövningsmyndigheten
2022-02830-01-269979
2022-05-18

Följande moment ingår i behandlingen
Behandlingen sker individuellt under 2-6 månader. Följande delar ingår:

- **Skattning av levnadsvanor:** Vi frågar dig om tobak, alkohol, kost, fysisk aktivitet, droger, sömn, sociala relationer, skärmtid, intressen/aktiviteter, sexuell hälsa. Vi visar dig hur du ligger till inom varje område. Du väljer om du vill svara.
- **Samtal om levnadsvanor:** Vi ger information och lyssnar på vad du har att säga. Du väljer en levnadsvana som du vill ändra på.
- **Genomförande:** Vi ger dig stöd och strategier anpassade för dig när du arbetar för att nå dina mål.
- **Avslut:** I slutet av behandlingen gör vi en plan för hur du kan jobba vidare på egenhand.

Eftersom det är en forskningsstudie innebär deltagande att man fyller formulär innan under och efter behandlingen. Det är för att vi ska kunna ta reda på vad du tycker om behandlingen och för att se om den påverkar din livskvalitet och välmående.

Vi söker dig som:

- vill förbättra dina levnadsvanor
- är över 18 år
- har någon form av funktionsnedsättning eller diagnos (exempelvis autism, ADHD, intellektuell funktionsnedsättning, förvärvad hjärnskada, rörelsenedsättning, dövblindhet, dövhet),
- vill ge synpunkter på insatsen så att vi kan fortsätta att utveckla den.

Övrigt

Deltagande i projektet är helt frivilligt och du kan när som helst av vilken anledning som helst avbryta deltagande. Deltagande eller beslut av att avbryta sitt deltagande påverkar inte på något vis den vård och det omhändertagande du normalt får vid den mottagning du tillhör. Den vårdpersonal som du träffar har behandlingsansvar även när du deltar i den här studien. För dig gäller att i högsta möjliga mån delta vid samtliga tillfällen. Behandlingen är gratis. Forskningshuvudman för projektet är Habilitering & Hälsa, Region Stockholm. Med forskningshuvudman menas den organisation som är ansvarig för projektet. Ansökan är godkänd av Etikprövningsmyndigheten, diarienummer för prövningen hos Etikprövningsmyndigheten är ange diarienummer

Personuppgifter, sekretess och patientförsäkring

Dina uppgifter behandlas i enlighet med Personuppgiftslagen. Personuppgiftsansvarig är din mottagning under pågående behandling som också för sedvanlig patientjournal. Alla uppgifter du lämnar skyddas av sekretess. Den fortsatta vetenskapliga bearbetningen av information från frågeformulären kommer att ske utan att enskilda personer kan identifieras. Du kan när som helst återkalla ditt samtycke. Deltagare i studien har rätt att ansöka om tillgång till den information vi sparar (journalkopia). Deltagare i projektet omfattas av patientförsäkringen.

Kontakt

Om du har frågor om projektet går det bra att vända sig till den behandlare på den enhet där du fick den här informationen eller ansvarig för projektet är Douglas Sjöwall, forskare verksam vid Habilitering & Hälsa samt Karolinska Institutet.
Douglas.sjowall@regionstockholm.se Habilitering & Hälsa, Box 454 36, 104 31 Stockholm 08-123 350 10.

Etikprövningsmyndigheten
2022-02830-01-269979
2022-05-18

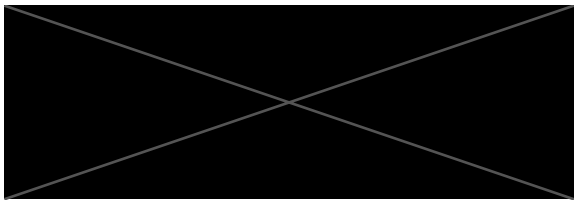

Variant 1 (mot personer utan funktionshinder), bild A (mot kvinnor)

Vill du delta i en studie som undersöker levnadsvanor hos vuxna i Sverige? Hälsosamma levnadsvanor har stor betydelse för psykisk och fysisk välmående, enligt Världshälsoorganisationen (WHO). Forskare vid Karolinska Institutet och Habilitering & Hälsa Region Stockholm, testar ett nytt frågeformulär som ska göra det enklare att utvärdera levnadsvanor och livskvalitet hos personer med och utan funktionsnedsättningar. Vill du veta mer om studien och hur man deltar? Klicka på länken nedan:

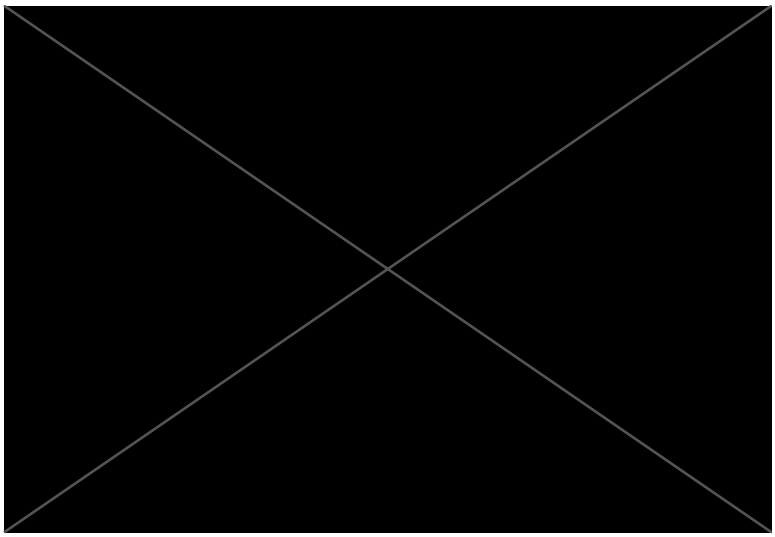

Etikprövningsmyndigheten
2022-02920-01-271950
2022-05-23

Variant 1, bild B (mot män)

Vill du delta i en studie som undersöker levnadsvanor hos vuxna i Sverige? Hälsosamma levnadsvanor har stor betydelse för psykisk och fysisk välmående, enligt Världshälsoorganisationen (WHO). Forskare vid Karolinska Institutet och Habilitering & Hälsa Region Stockholm, testar ett nytt frågeformulär som ska göra det enklare att utvärdera levnadsvanor och livskvalitet hos personer med och utan funktionsnedsättningar. Vill du veta mer om studien och hur man deltar? Klicka å länken nedan:

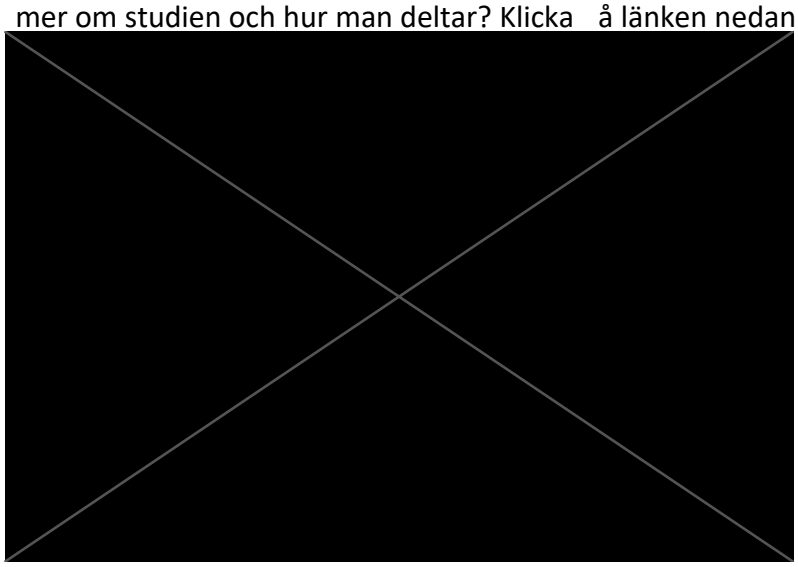

Variant 2 (mot personer med funktionshinder), bild A (mot män)

Vill du delta i en studie som undersöker levnadsvanor hos personer med och utan funktionsnedsättningar? Hälsosamma levnadsvanor har stor betydelse för psykisk och fysisk välmående, enligt Världshälsoorganisationen (WHO). Forskare vid Karolinska Institutet och Habilitering & Hälsa Region Stockholm, testar ett nytt frågeformulär som ska göra det enklare att utvärdera levnadsvanor och livskvalitet. Syftet med studien är att främja psykisk och fysisk hälsa och hjälpa utveckla förebyggande insatser inom vården. Vill du veta mer om studien och hur man deltar? Klicka på länken nedan:

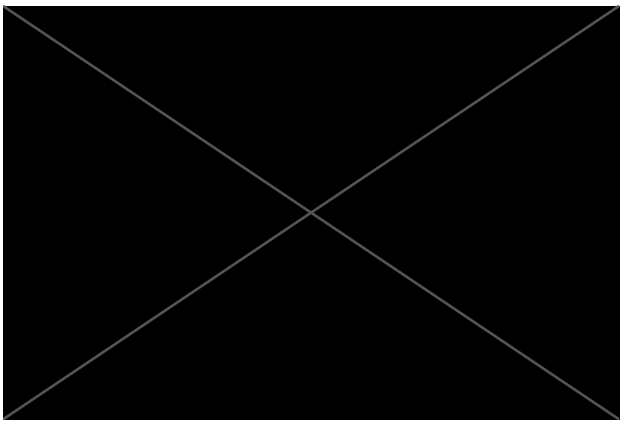

Etikprövningsmyndigheten
2022-02920-01-271950
2022-05-23

Variant 2 (mot personer med funktionshinder) bild B (mot kvinnor)

Vill du delta i en studie som undersöker levnadsvanor hos personer med och utan funktionsnedsättningar? Hälsosamma levnadsvanor har stor betydelse för psykisk och fysisk välmående, enligt Världshälsoorganisationen (WHO). Forskare vid Karolinska Institutet och Habilitering & Hälsa Region Stockholm, testar ett nytt frågeformulär som ska göra det enklare att utvärdera levnadsvanor och livskvalitet. Syftet med studien är att främja psykisk och fysisk hälsa och hjälpa utveckla förebyggande insatser inom vården. Vill du veta mer om

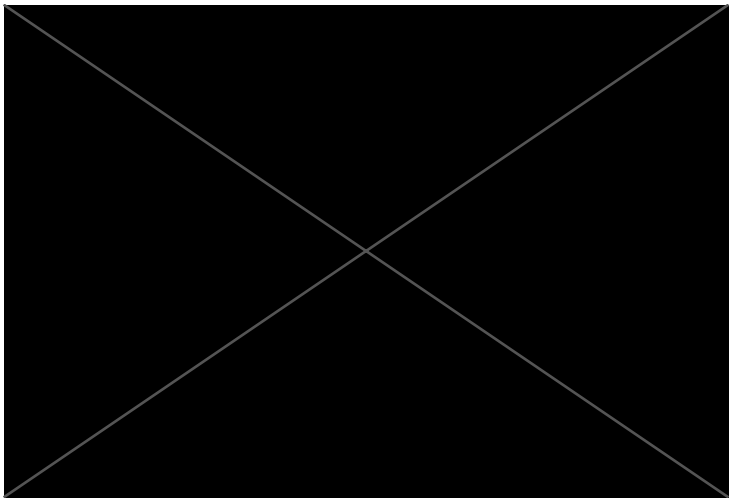

Lev-projektet: Kartläggning, bedömning och intervention av hälsorelaterade levnadsvanor hos vuxna med och utan funktionsnedsättning.

Information till forskningspersoner

Vi vill fråga dig om du vill delta i ett forskningsprojekt. I det här dokumentet får du information om projektet och om vad det innebär att delta.

Vad är det för ett projekt och varför vill ni att jag ska delta?

Ohälsosamma levnadsvanor leder enligt Världshälsoorganisationen (WHO) till psykisk och fysisk ohälsa. För att du, på egen hand eller tillsammans med hälso- och sjukvård, ska kunna må bättre är ett viktigt steg att kunna mäta hur dina levnadsvanor ser ut. I det här projektet utvärderar vi ett nytt frågeformulär som ska göra det enklare att mäta levnadsvanor (Lev-s).

Forskningshuvudman för projektet är Habilitering & Hälsa, Region Stockholm. Med forskningshuvudman menas den organisation som är ansvarig för projektet. Ansökan är godkänd av Etikprövningsmyndigheten, diarienummer för prövningen hos Etikprövningsmyndigheten är ange diarienummer

Hur går projektet till?

Om du deltar kommer du få svara på frågor vid 2 tillfällen.

- Vid första tillfället fyller du i tre formulär: ett om levnadsvanor (Lev-s) och ett om livskvalitet (WHOQOL) samt ett om bakgrundsinformation.
- Efter ca 2 veckor ber vi dig fylla i formuläret om levnadsvanor (Lev-s) igen. Det är ett sätt att testa hur säkert formuläret är. Om du inte ändrat dina levnadsvanor ska resultatet vara ungefär samma som gången innan.

Ta dig tid och fundera på om du vill vara med. Om du vill delta ska du skriva under samtycket på nästa sida. Sedan fyller du i frågorna på egen hand. Vi bokar dessutom in en tid om två veckor då vi ses online/på plats. Då kommer du få samma frågor men skillnaden är att jag ställer dem. Vi gör det för att studera om vi får samma svar.

Möjliga följder och risker med att delta i projektet

Riskerna är låga. Vissa kan uppleva en del frågor som privata men vi kommer inte berätta hur du svarat för någon annan. De områden vi frågar om är tobak, alkohol, kost, fysisk aktivitet, droger, sömn, vänner, sex, intressen, skärmtid. Du har rätt att avstå att svara på enskilda frågor om du vill. Om du skulle må sämre efter att ha svarat på frågorna kan vi sätta dig i kontakt med lämpligt stöd. Frågorna är utvalda att visa på vad som är viktigt att tänka på inom varje levnadsvana. En positiv effekt kan därför bli att man blir medveten om hur man kan leva mer hälsosamt.

Vad händer med mina uppgifter?

Etikprövningsmyndigheten
2022-02920-01-269980
2022-05-23

Lev-projektet: Kartläggning, bedömning och intervention av hälsorelaterade levnadsvanor hos vuxna med och utan funktionsnedsättning.

Svaren förvaras under datainsamlingen inlåsta i ett journalskåp. Svaren på frågorna läggs sedan in i en datafil. I stället för att lägga in ditt namn används en kod för att du ska kunna vara anonym. Excelfilen förvaras inlåst på Karolinska Institutet och på Habilitering & Hälsa, Region Stockholm och endast personer som är involverade i databearbetningen har tillgång till filen. Kodnyckeln förvaras separerad från databasen på Habilitering & Hälsa, Region Stockholm.

Dina svar och dina resultat kommer att behandlas så att inte obehöriga kan ta del av dem.

Ansvarig för dina personuppgifter är Habilitering & Hälsa, Regions Stockholm. Enligt EU:s dataskyddsförordning har du rätt att kostnadsfritt få ta del av de uppgifter om dig som hanteras i projektet, och vid behov få eventuella fel rättade. Du kan också begära att uppgifter om dig raderas samt att behandlingen av dina personuppgifter begränsas. Rätten till radering och till begränsning av behandling av personuppgifter gäller dock inte när uppgifterna är nödvändiga för den aktuella forskningen. Om du vill ta del av uppgifterna ska du kontakta Douglas Sjöwall Habilitering & Hälsa, Box 454 36, 104 31 Stockholm 08-123 350 10. Dataskyddsombud Camilla Heise Löwgren nås på camilla.heise-lowgren@regionstockholm.se Om du är missnöjd med hur dina personuppgifter behandlas har du rätt att ge in klagomål till Integritetsskyddsmyndigheten, som är tillsynsmyndighet.

Etikprövningsmyndigheten
2022-02920-01-269980
2022-05-23

Hur får jag information om resultatet av projektet?

Du behöver inte ta del av dina eller studiens resultat. Om du vill ta del av resultatet av hela projektet hänvisar vi dig till de vetenskapliga artiklar som vi kommer sammanställa. Det kan dröja några år innan studien publiceras. Kontakta projektansvarig (Douglas Sjöwall) om du har frågor gällande resultaten från projektet.

Försäkring och ersättning

Sedvanligt patientförsäkringsskydd gäller för deltagarna som rekryteras via kliniska verksamheter.

Deltagandet är frivilligt

Ditt deltagande är frivilligt och du kan när som helst välja att avbryta deltagandet. Om du väljer att inte delta eller vill avbryta ditt deltagande behöver du inte uppge varför, och det kommer inte heller att påverka din framtida vård eller behandling.

Om du vill avbryta ditt deltagande ska du kontakta den ansvariga för projektet (se nedan).

Ansvariga för projektet

Ansvarig för projektet är Douglas Sjöwall Habilitering & Hälsa, Box 454 36, 104 31 Stockholm 08-123 350 10. Douglas.sjowall@regionstockholm.se

Lev-projektet: Kartläggning, bedömning och intervention av hälsorelaterade levnadsvanor hos vuxna med och utan funktionsnedsättning.

Information till forskningspersoner

Vi vill fråga dig om du vill delta i ett forskningsprojekt. I det här dokumentet får du information om projektet och om vad det innebär att delta i Lev - genomförbarhetsstudien.

Vad är det för ett projekt och varför vill ni att jag ska delta?

Enligt Världshälsoorganisationen (WHO) kan vi må bättre och leva längre om vi har hälsosammare levnadsvanor. Hälso- och sjukvård behöver bli bättre på att ge stöd och insatser som syftar till hälsosammare levnadsvanor hos personer med någon form av funktionsnedsättning. I det här projektet utvärderar vi en ny insats (Lev) som har som syfte att förbättra levnadsvanor. Lev har utvecklats i ett samarbete mellan forskare, aktörer inom hälso- och sjukvård samt representanter för olika funktionsnedsättningar.

Vi söker dig som:

- vill förbättra dina levnadsvanor,
- har någon form av funktionsnedsättning eller diagnos (exempelvis autism, ADHD, intellektuell funktionsnedsättning, förvärvad hjärnskada, rörelsenedsättning, dövblindhet, dövhet),
- vill ge synpunkter på insatsen så att vi kan fortsätta att utveckla den.

Om du är intresserad fyller du i samtycket som finns längst bak. Du får då en individuell bedömning av en behandlare där du fick information om studien. Efter det får du besked om du kan erbjudas plats i studien.

Forskningshuvudman för projektet är Habilitering & Hälsa, Region Stockholm. Med forskningshuvudman menas den organisation som är ansvarig för projektet. Ansökan är godkänd av Etikprövningsmyndigheten, diarienummer för prövningen hos Etikprövningsmyndigheten är ange diarienummer

Etikprövningsmyndigheten
2022-02920-01-269981
2022-05-23

Hur går projektet till?

Att delta i genomförbargetsstudien innebär följande:

- **Mätning:** En utvärdering dina levnadsvanor
- **Insatsen (Lev):**
    - Fas 1: Samtal om levnadsvanor, målsättning + eventuellt stöd från annan aktör inom hälso- och sjukvård.
    - Fas 2: Fördjupad analys som leder till individuella strategier och stöd för hur du ska nå dina mål.
    - Fas 3: Avslut och ny utvärdering av levnadsvanor, målsättning och ifyllnad av självskattningsformulär. Plan för eget arbete.

Lev-projektet: Kartläggning, bedömning och intervention av hälsorelaterade levnadsvanor hos vuxna med och utan funktionsnedsättning.

De 3 faserna i Lev ges under ca 2-6 månader. Du fyller i skattningar före under och efter deltagande i insatsen. Skattningarna inkluderar frågor om livskvalitet, vad du tycker om behandlingen och dess delar samt bakgrundsinformation (exempelvis kön, din ålder, sysselsättning och om du har någon funktionsnedsättning).

Möjliga följder och risker med att delta i projektet

Riskerna är låga. Vissa kan uppleva en del frågor som privata. De områden vi frågar om är tobak, alkohol, kost, fysisk aktivitet, droger, sömn, vänner, sex, intressen, skärmtid. Du har rätt att avstå att svara på enskilda frågor om du vill och du väljer själv vilken levnadsvana du vill fokusera på. Vi kommer inte berätta för någon hur du svarat. Om du mot förmodan skulle må sämre efter att ha fått insatsen kan vi sätta dig i kontakt med lämpligt stöd. Positiva följder kan vara att du mår bättre psykiskt och fysiskt.

Vad händer med mina uppgifter?

Projektet kommer att samla in och registrera information om dig.

Svaren förvaras under datainsamlingen inlåsta i ett journalskåp. Svaren på frågorna läggs sedan in i en datafil. I stället för att lägga in ditt namn används en kod för att du ska kunna vara anonym. Datafilen förvaras inlåst på Karolinska Institutet och på Habilitering & Hälsa, Region Stockholm och endast personer som är involverade i databearbetningen har tillgång till filen. Kodnyckeln förvaras separerad från databasen på Habilitering & Hälsa, Region Stockholm.

Dina svar och dina resultat kommer att behandlas så att inte obehöriga kan ta del av dem.

Ansvarig för dina personuppgifter är Habilitering & Hälsa, Regions Stockholm. Enligt EU:s dataskyddsförordning har du rätt att kostnadsfritt få ta del av de uppgifter om dig som hanteras i projektet, och vid behov få eventuella fel rättade. Du kan också begära att uppgifter om dig raderas samt att behandlingen av dina personuppgifter begränsas. Rätten till radering och till begränsning av behandling av personuppgifter gäller dock inte när uppgifterna är nödvändiga för den aktuella forskningen. Om du vill ta del av uppgifterna ska du kontakta Douglas Sjöwall Habilitering & Hälsa, Box 454 36, 104 31 Stockholm 08-123 350 10. Dataskyddsombud Camilla Heise Löwgren nås på camilla.heise-lowgren@regionstockholm.se. Om du är missnöjd med hur dina personuppgifter behandlas har du rätt att ge in klagomål till Integritetsskyddsmyndigheten, som är tillsynsmyndighet.

Hur får jag information om resultatet av projektet?

Du behöver inte ta del av dina eller studiens resultat. Om du vill ta del av resultatet av hela projektet hänvisar vi dig till de vetenskapliga artiklar som vi kommer sammanställa. Det kan dröja några år innan studien publiceras. Kontakta projektansvarig (Douglas Sjöwall) om du har frågor gällande resultaten från projektet.

Etikprövningsmyndigheten
2022-02920-01-269981
2022-05-23

Lev-projektet: Kartläggning, bedömning och intervention av hälsorelaterade levnadsvanor hos vuxna med och utan funktionsnedsättning.

Försäkring och ersättning

Sedvanligt patientförsäkringsskydd gäller för deltagarna via de kliniska verksamheter som utför insatsen.

Ingen ersättning utgår för deltagande i projektet.

Deltagandet är frivilligt

Ditt deltagande är frivilligt och du kan när som helst välja att avbryta deltagandet. Om du väljer att inte delta eller vill avbryta ditt deltagande behöver du inte uppge varför, och det kommer inte heller att påverka din framtida vård eller behandling.

Om du vill avbryta ditt deltagande ska du kontakta den ansvariga för projektet (se nedan).

Ansvariga för projektet

Ansvarig för projektet är Douglas Sjöwall Habilitering & Hälsa, Box 454 36, 104 31 Stockholm 08-123 350 10. Douglas.sjowall@regionstockholm.se

Etikprövningsmyndigheten
2022-02920-01-269981
2022-05-23

Lev-projektet: Kartläggning, bedömning och intervention av hälsorelaterade levnadsvanor hos vuxna med och utan funktionsnedsättning

Samtycke till att delta i projektet

Jag har fått muntlig och/eller skriftlig information om studien och har haft möjlighet att ställa frågor. Jag får behålla den skriftliga informationen.

- Jag samtycker till att delta i projektet Lev delstudie 1.
- Jag samtycker till att personuppgifter inkl ifyllda formulär behandlas och sparas på det sätt som beskrivs i forskningspersonsinformationen.

Ort..... Datum.....

Underskrift.....

Namnförtydligande.....

Etikprövningsmyndigheten
2022-02920-01-269982
2022-05-23

Lev-projektet: Kartläggning, bedömning och intervention av hälsorelaterade levnadsvanor hos vuxna med och utan funktionsnedsättning

Samtycke till att delta i projektet

Jag har fått muntlig och/eller skriftlig information om studien och har haft möjlighet att ställa frågor. Jag får behålla den skriftliga informationen.

- Jag samtycker till att delta i projektet Lev genomförbarhetsstudien.
- Jag samtycker till att personuppgifter inkl ifyllda formulär behandlas och sparas på det sätt som beskrivs i forskningspersonsinformationen.

Ort..... Datum.....

Underskrift.....

Namnförtydligande.....

Etikprövningsmyndigheten
2022-02920-01-269983
2022-05-23

Lev-Bakgrundsinformation

Det är viktigt att du fyller i alla frågor vid båda tillfällena. Det hjälper oss att kunna koppla ihop dina svar samtidigt som du kan fortsätta att vara anonym.

1. Hur gammal är du?.....

2. Är du man eller kvinna (annat)?.....

3. Boendesituation (själv, med föräldrar, partner, kompis, gruppboende mm)?

……

……

4. Vad har du för funktionsnedsättning/diagnoser?

……

……

……

……

5. Utbildningsbakgrund

- Grundskola
- Gymnasieexamen
- Examen från högskola/universitet
- Annat, ange vad:……

6. Nuvarande sysselsättning och försörjning (fler alternativ kan anges)

☐ Arbetar

☐ Studerar

☐ Aktivitetsersättning/förtidspension

☐ Sjukskriven

☐ Arbetssökande

☐ Försörjningsstöd

☐ Annat, ange vad:……

Etikprövningsmyndigheten
2022-02920-01-269984
2022-05-23

Formuläret fortsätter på nästa sida!

7. Hälsa

Har Du för närvarande några fysiska sjukdomar?

- JA
- NEJ

Om JA, ange vilka (t ex migrän, ryggvärk, nackvärk eller högt blodtryck etc)?

……
……
……

8. Annan pågående behandling

Får Du någon annan typ av behandling (terapi, grupp, arbetsterapi eller annat)?

- JA
- NEJ

Om JA, beskriv vilken typ av behandling(ar):

……
……

9. Allmänt välbefinnande och mående

Skatta mellan 1 och 10 hur Du uppfattar Ditt nuvarande allmänna välbefinnande och mående:

1 2 3 4 5 6 7 8 9 10

Sämsta möjliga Bästa möjliga

Tack för din medverkan!

Etikprövningsmyndigheten
2022-02920-01-269984
2022-05-23

1. Hur gammal är du?.....

2. Är du man eller kvinna (annat)?.....

3. Boendesituation (själv, med föräldrar, partner, kompis, gruppboende mm)?

.....

.....

4. Vad har du för funktionsnedsättning/diagnoser?

.....

.....

.....

.....

5. Utbildningsbakgrund

- Grundskola
- Gymnasieexamen
- Examen från högskola/universitet
- Annat, ange vad:.....

6. Nuvarande sysselsättning och försörjning (fler alternativ kan anges)

☐ Arbetar

☐ Studerar

☐ Aktivitetsersättning/förtidspension

☐ Sjukskriven

☐ Arbetssökande

☐ Försörjningsstöd

☐ Annat, ange vad:.....

Etikprövningsmyndigheten
2022-02920-01-269985
2022-05-23

Formuläret fortsätter på nästa sida!

7. Hälsa

Har Du för närvarande några fysiska sjukdomar?

- JA
- NEJ

Om JA, ange vilka (t ex migrän, ryggvärk, nackvärk eller högt blodtryck etc)?

……
……
……

8. Annan pågående behandling

Får Du någon annan typ av behandling (terapi, grupp, arbetsterapi eller annat)?

- JA
- NEJ

Om JA, beskriv vilken typ av behandling(ar):

……
……

9. Allmänt välbefinnande och mående

Skatta mellan 1 och 10 hur Du uppfattar Ditt nuvarande allmänna välbefinnande och mående:

1 2 3 4 5 6 7 8 9 10

Sämsta möjliga Bästa möjliga

10. Vilka 3 levnadsvanor känns viktigast för dig?

1. ……

2. ……

3. ……

11. Vilka hinder upplever du som gör det svårt att leva hälsosamt gällande dessa tre levnadsvanor?

……
……
……

Etikprövningsmyndigheten
2022-02920-01-269985
2022-05-23

……
……
……

Skatta mellan 1 och 10 hur Du uppfattar stöd från hälso- och sjukvård gällande levnadsvanor utifrån följande aspekter:

12. Att uppmärksamma levnadsvanor: I vilken utsträckning frågar hälso- och sjukvård om de tre levnadsvanor du tycker är viktigast?

1 2 3 4 5 6 7 8 9 10

Aldrig Oftast

13. Att åtgärda levnadsvanor: I vilken utsträckning får du råd och stöd till förändring gällande dessa tre levnadsvanor?

1 2 3 4 5 6 7 8 9 10

Aldrig Oftast

14. Uppföljning av levnadsvanor: I vilken utsträckning följer man upp för att se om du nått dina mål kring dessa levnadsvanor?

1 2 3 4 5 6 7 8 9 10

Aldrig Oftast

Övrigt som du vill upplysa om som kan hjälpa oss utveckla bättre insatser och stöd för hälsosamma levnadsvanor?

……
……
……
……

Tack för din medverkan!

Etikprövningsmyndigheten
2022-02920-01-269985
2022-05-23

1. Personuppgifter

Namn: .....

Personnummer: .....

2. Boendesituation (själv, med föräldrar, partner, kompis, gruppboende mm)?

.....

.....

3. Vad har du för funktionsnedsättning/diagnoser?

.....

.....

.....

.....

4. Utbildningsbakgrund

- Grundskola
- Gymnasieexamen
- Examen från högskola/universitet
- Annat, ange vad:.....

5. Nuvarande sysselsättning och försörjning (fler alternativ kan anges)

☐ Arbetar

☐ Studerar

☐ Aktivitetsersättning/förtidspension

☐ Sjukskriven

☐ Arbetssökande

☐ Försörjningsstöd

☐ Annat, ange vad:.....

Etikprövningsmyndigheten
2022-02920-01-269986
2022-05-23

Formuläret fortsätter på nästa sida!

6. Hälsa

Har Du för närvarande några fysiska sjukdomar?

- JA
- NEJ

Om JA, ange vilka (t ex migrän, ryggvärk, nackvärk eller högt blodtryck etc)?

……
……
……

7. Annan pågående behandling

Får Du någon annan typ av behandling (terapi, grupp, arbetsterapi eller annat)?

- JA
- NEJ

Om JA, beskriv vilken typ av behandling(ar):

……
……

8. Allmänt välbefinnande och mående

Skatta mellan 1 och 10 hur Du uppfattar Ditt nuvarande allmänna välbefinnande och mående:

1 2 3 4 5 6 7 8 9 10

Sämsta möjliga Bästa möjliga

Etikprövningsmyndigheten
2022-02920-01-269986
2022-05-23

Tack för din medverkan!

Skattning av levnadsvanor (Lev-s)

Bakgrund

Det här formuläret syftar till att ge en översiktlig kartläggning av levnadsvanor och att identifiera var det kan finnas ett behov att göra ändringar. Det finns etablerade mer djupgående formulär inom varje område och syftet är inte att ersätta dessa utan att fungera som ett komplement.

Individanpassningar

Du kan ibland behöva anpassa den frågan för att öka förståelsen. Detta kan du göra genom att förklara frågan mer noga, anpassa språket, ställa följdfrågor och/eller referera till information som exempelvis framkommit tidigare. Du kan även läsa svarsalternativen högt.

Poängsättning

Tanken är att du välj det svarsalternativ som passar bäst utan att läsa dem för deltagaren. Läs dock svarsalternativen högt om det underlättar. På den skalan som används (0, 1, 2, 3) är 3 poäng det högsta (indikation på hälsosam vana) och 0 poäng det lägsta (indikation för ohälsosam vana). Bedömningen för varje levnadsvana placeras ut i hjulet för att synliggöra vart det finns utrymme för hälsosammare vanor. Bedömningen baseras på ibland på medelvärdet och ibland på minimum. Till din hjälp finns en excel-mall.

Återkoppling av resultat till deltagaren

Visa resultatet med hjälp av att markera ut prickar för varje levnadsvana i översiktsfiguren. Kom ihåg att vissa deltagare kan bli nedslagna av att se sina resultat. Försök att betona att levnadsvanorna inte är egenskaper utan vanor som kan ändras och att de gjort rätt som nu tagit tag i dem. Se vidare instruktion för hur du går igenom resultatet finns i anslutning till översiktsfiguren.

Information till deltagaren

Att ta tag i sina levnadsvanor kan förändra ditt liv på ett väldigt positivt sätt. Första steget är att ta reda på hur ens levnadsvanor ser ut. Levnadsvanor är inte dina egenskaper. Det är vanor som du har just nu men som kan ändras. Även om vi identifierar områden där du skulle kunna leva hälsosammare är det helt upp till dig om vi ska gå vidare och jobba med någon levnadsvana.

I frågorna om levnadsvanor ingår det frågor som av vissa kan upplevas som känsliga. Vi har tystnadsplikt och dina svar kommer inte leda till några negativa konsekvenser. De områden vi tar upp är sömn, kost, fysisk aktivitet, intressen/aktiviteter, skärmtid, vänner/familj, tobak, alkohol, droger och sexuell hälsa. Inför varje område frågar vi om det är okej att vi ställer några frågor. Du kan välja att hoppa över ett område eller att inte svara på enskilda frågor. Är det okej att jag frågar om dina levnadsvanor? Eftersträva att förmedla denna text i god tid till deltagaren så att det finns utrymme att reflektera över om man är redo att svara på frågorna inom alla områden.

Inom ramen för reliabilitetsstudien andra mättillfälle (två veckor efter det första) frågar behandlaren om deltagaren upplever att det skett någon förändring inom någon av levnadsvanorna sedan sist.

- Nej, ingen förändring
- Ja, följande har ändrats……

Etikprövningsmyndigheten
2022-02-26
2022-05-18
2022-01-269987

Tobak

Tobaksbruk innefattar konsumtion av cigaretter, pipa, cigarr, cigarill, vattenpipa samt rökfri tobak, snus och tuggtobak. E-cigaretter ingår inte i definitionen.

Är det okej om jag frågar om dina tobaksvanor?

Förekommer tobak

Har du någonsin rökt eller använt någon annan form av tobak?

- Ja, röker/snusar/använder tobak i dagsläget 0 poäng
- Ja, när det är fest 1 poäng
- Nej, men slutade för mindre än 6 månader sedan 2 poäng
- Nej, har aldrig rökt/slutade för mer än 6 månader sedan 3 poäng

Kvantitet

Hur mycket tobak använder du i dagsläget? *Om flera former av tobak används slås användningen ihop.*

- Använder tobak flera gånger dagligen 0 poäng
- Använder tobak, någon gång dagligen 1 poäng
- Använder tobak, men inte dagligen 2 poäng
- Använder inte tobak 3 poäng

Etikprövningsmyndigheten
2022-02-26 Dnr 2022-01-269987
2022-05-18

Kost

Är det okej att jag frågar om dina matvanor?

Alla frågor handlar om hur det varit den senaste månaden.

Kostindex från Socialstyrelsen

Hur ofta äter du grönsaker och/eller rotfrukter (färska, frysta eller tillagade)?

- En gång i veckan eller mer sällan 0 poäng

- Några gånger i veckan 1 poäng
- En gång per dag 2 poäng
- Två gånger per dag eller oftare 3 poäng

Hur ofta äter du frukt och/eller bär (färska, frysta, konserverade, etc.)?

- En gång i veckan eller mer sällan 0 poäng
- Några gånger i veckan 1 poäng
- En gång per dag 2 poäng
- Två gånger per dag eller oftare 3 poäng

Hur ofta äter du fisk eller skaldjur som huvudrätt, i sallad eller som pålägg? *Om du är vegan/vegetarian kan du byta ut fisk och skaldjur mot vegetabiliska alternativ (exempelvis nötter, avokado, bönor/linser).*

- Några gånger i månaden eller mer sällan 0 poäng
- En gång i veckan 1 poäng
- Två gånger i veckan 2 poäng
- Tre gånger i veckan eller oftare 3 poäng

Hur ofta äter du kaffebröd, choklad/godis, chips eller läsk/saft?

- Två gånger per dag eller oftare 0 poäng
- En gång per dag 1 poäng
- Några gånger i veckan 2 poäng
- En gång i veckan eller mer sällan 3 poäng

Hur ofta äter du frukost?

- En gång i veckan eller mer sällan 0 poäng
- Några gånger i veckan 1 poäng
- Nästan varje dag 2 poäng
- Dagligen 3 poäng

<u>Ätbeteende</u>

Ibland äter vi eller låter bli att äta av anledningar som inte har att göra med hunger. Hur ofta händer det att du äter/inte äter för att du känner dig stressad, ångestfylld, uttråkad, ledsen eller uppspelt?

- Det händer mycket ofta, varje dag 0 poäng

Etikprövningsmyndigheten
2022-02926-01-269987
2022-05-18

- Det händer ofta, några gånger i veckan 1 poäng
- Det händer ibland, några gånger i månaden 2 poäng
- Det händer sällan/aldrig 3 poäng

Alkohol

Är det okej om jag frågar om dina alkoholvanor?

Alla frågor jag ställer handlar om hur det varit senaste månaden.

Kvantitet – hur ofta

Hur ofta dricker du alkohol?

- 4 gånger /veckan eller mer 0 poäng
- 2-3 gånger i veckan 1 poäng
- 2-4 gånger i månaden 2 poäng
- 1 gång/månad eller mer sällan 3 poäng

Kvantitet – hur mycket

Hur många glas dricker du en typisk dag när du dricker alkohol? *Exempel på standardglas är 12-15 cl vin, 2 x 33 cl lättöl, 50 cl folköl, 33 cl starköl, 4 cl starksprit.*

- 7 eller fler standardglas 0 poäng
- 5-6 standardglas 1 poäng
- 3-4 standardglas 2 poäng
- 1-2 standardglas eller mindre 3 poäng

Kvantitet – vid ett tillfälle

Hur ofta dricker du som är kvinna 4 standardglas eller mer och du som är man 5 standardglas eller mer vid ett och samma tillfälle? *Exempel på standardglas är 12-15 cl vin, 2 x 33 cl lättöl, 50 cl folköl, 33 cl starköl, 4 cl starksprit.*

- Dagligen eller nästa dagligen 0 poäng
- Varje vecka 1 poäng
- Varje månad 2 poäng
- Aldrig eller mer sällan än en gång i månaden 3 poäng

Etikprövningsmyndigheten
2022-02-26
2022-05-18

Fysisk aktivitet

Är det okej att jag frågar om din fysiska aktivitet?

Alla frågor handlar om hur det varit den senaste månaden.

Fysisk träning

Hur mycket tid ägnar du en vanlig vecka åt fysisk träning? Med fysisk träning menas det som får dig att bli andfådd och/eller få högre puls, exempelvis löpning, motionsgymnastik och bollsport.

- Mindre än 30 minuter (halvtimme) 0 poäng
- 30-75 minuter (ca en halvtimme till lite mer än en timme) 1 poäng
- 75-150 minuter (en till 2,5 timmar) 2 poäng
- Mer än 150 minuter (mer än 2,5 timmar) 3 poäng

Fysisk aktivitet i vardagen

Hur mycket tid ägnar du en vanlig vecka åt vardagsmotion? Med vardagsmotion menas det som gör att du får högre puls, blir andfådd eller lite varm. Exempel på det kan vara en rask promenad, cykling eller att dammsuga.

- Mindre än 30 minuter (halvtimme) 0 poäng
- 30-150 minuter (en halvtimme-2,5 timmar) 1 poäng
- 150-300 minuter (2,5-5 timmar) 2 poäng
- Mer än 300 minuter (mer än 5 timmar) 3 poäng

Stillasittande

Hur stor andel av din vakna tid sitter/ligger du stilla på en dag? I genomsnitt. *En person som sover 8h är vaken 16h.*

- Nästan hela dagen (ca 15h) 0 poäng
- 75% av dagen (ca 12h) 1 poäng
- 50% av dagen (ca 8h) 2 poäng
- 25% av dagen (4h) 3 poäng

Avbrott i stillasittande

Etikprövningsmyndigheten
2022-02926-01-269987
2022-05-18

Hur ofta avbryter du stillasittandet med rörelse? Exempelvis, städar, går på toa, går omkring en stund.

- Var fjärde timme eller mer sällan 0 poäng
- Varannan timme 1 poäng
- En gång i timmen 2 poäng
- En gång i halvtimmen 3 poäng

Sömn

Är det okej att jag frågar om din sömn?

Alla frågor handlar om hur det varit den senaste månaden.

Etikprövningsmyndigheten
2022-02-26
2022-05-18
2022-02-26-01-269987

Duration

Hur många timmar sover du vanligtvis per natt? *Om du har svårt att veta kan det hjälpa att skriva ned när du går du lägger dig, somnar och vaknar.*

- Mindre än 5h alternativt mer än 10h 0 poäng
- 5-6h alternativt 9-10h 1 poäng
- 6-7h 2 poäng
- 7-9h 3 poäng

Kontinuitet

Har du problem med något av följande: svårt att somna, att du vaknar på natten eller för tidigt på morgonen? *Här kan du dela upp frågan i tre: ”Har du svårt att somna?” ”Har du problem med att du vaknar på natten?” ”Har du problem med att du vaknar för tidigt på morgonen?” Lägg ihop problemen.*

- Ja, 4 gånger i veckan eller mer 0 poäng
- Ganska ofta, 2-3 gånger i veckan 1 poäng
- Ganska sällan, 2-4 gånger i månaden 2 poäng
- Nej, en gång i månaden eller mer sällan 3 poäng

Regelbundenhet

Går du och lägger dig och går upp ungefär samma tid varje dag? *Med samma tid menar vi här om det skiljer mer än en timme vid sänggående/uppstigning. Eventuella skillnader mellan vardag och helg ska också räknas in.*

- Nej, sover väldigt oregelbundet 0 poäng
- Ganska sällan, har sömnrutiner men frångår dem 3-4 dagar/veckan 1 poäng
- Ganska ofta, har sömnrutiner men frångår dessa 1-2 dagar/veckan 2 poäng
- Ja, jag sover väldigt regelbundet 3 poäng

Pigghet/trötthet

Känner du att du orkar med under hela dagen? *Med ”orka med” menas att man orkar genomföra dagen och de saker man planerat. Motsatsen skulle kunna vara att man ställer in saker eller gör dem slarvigt för att man är trött på grund av sömnbrist.*

- Nej 0 poäng
- Ibland, 3-4 dagar i veckan 1 poäng
- Ofta, 5-6 dagar i veckan 2 poäng
- Ja 3 poäng

Tillfredsställelse

Hur nöjd är du med din sömn på en skala från 0-3 där 0 står för ”Missnöjd” och 3 för ”Nöjd”?

- Missnöjd (önskar stor förändring) 0 poäng
- Ganska missnöjd (det finns önskemål om förändring) 1 poäng
- Ganska nöjd (trivs bra men det finns önskemål om mindre förändring) 2 poäng
- Nöjd (önskar inga/endast marginella förändringar) 3 poäng

Intressen/aktiviteter

Är det okej att jag frågar om vad du gillar att göra för aktiviteter?

Alla frågor handlar om hur det varit den senaste månaden.

Trivsamma fritidsintressen

Hur ofta ägnar dig åt något fritidsintresse (idrott/hobby/intresse) som du mår bra av? Med ”mår bra” menar vi att du blir dig glad eller lugn av den.

- Förekommer inte, alternativt mer sällan än 1 gång/månaden 0 poäng
- Ganska sällan, 2-4 gånger i månaden 1 poäng

Etikprövningsmyndigheten
2022-02-26
2022-05-18

- Ganska ofta, 2-3 gånger i veckan 2 poäng
- Ja, 4 gånger i veckan eller mer 3 poäng

Nöjdhet med aktiviteter i vardagen (inkl. delaktighet och meningsfullhet)

Hur nöjd är du med det du gör om dagarna på en skala från 0-3 där 0 står för ”missnöjd” och 3 för ”nöjd”. Du kan även räkna in jobb/sysselsättning om det bidrar med att du känner meningsfullhet.

- Missnöjd (önskar stor förändring) 0 poäng
- Ganska missnöjd (finns önskemål om förändring) 1 poäng
- Ganska nöjd (trivs men finns önskemål om små förändringar) 2 poäng
- Nöjd (önskar ingen/endast marginella förändringar) 3 poäng

Problem gällande kvantitet

Har någon annan kommenterat hur mycket tid du ägnar åt din/dina fritidsaktiviteter?

Tex skärmtid.

- Ja, omgivningen tycker att jag oftast lägger för mycket tid 0 poäng
- Ja, omgivningen tycker att jag ibland lägger för mycket tid 1 poäng
- Väldigt sällan, men det har hänt 2 poäng
- Nej, (eller bara positiva kommentarer) 3 poäng

Etikprövningsmyndigheten
2022-02-26
Dnr 2022-01-269987
2022-05-18

Vänner/familj

Är det okej att jag frågar om dina sociala relationer?

Alla frågor handlar om hur det varit den senaste månaden.

Kvalitet på sociala relationer

Har du någon som du verkligen litar på?

- Nej 0 poäng
- Vet inte, kanske 1 poäng
- Tror det 2 poäng
- Ja 3 poäng

Ensamhet

Hur ofta känner du sig ensam?

- Alltid, alternativt 4 gånger i veckan eller mer 0 poäng
- Ofta, 2-3 gånger i veckan 1 poäng
- Ibland, 2-4 gånger i månaden 2 poäng
- Aldrig, alternativt mer sällan en 1 gång/månad 3 poäng

Tillfredställelse med sociala relationer

Hur nöjd är du med dina vänner/relationer på en skala från 0-3 där 0 står för ”missnöjd” och 3 för ”nöjd”? *Missnöjet kan både handla om en önskan att ha fler eller närmare relationer.*

- Missnöjd (önskar stor förändring) 0 poäng
- Ganska missnöjd (finns önskemål om förändring) 1 poäng
- Ganska nöjd (trivs men det finns önskemål om liten förändring) 2 poäng
- Nöjd (önskar inga/endast marginella förändringar) 3 poäng

Relationer utanför familjen

Hur många vänner har du utanför din familj? *Om du har vänner online räknas dessa om ni även ses fysiskt (IRL). Du avgör vem som räknas som vän.*

- Ingen 0 poäng
- En 1 poäng
- Två 2 poäng
- Tre eller fler 3 poäng

Sexuell hälsa

Sexualitet kan vara en viktig del av vårt välmående och njutning. Exempel på sex kan vara sex med sig själv, någon annan eller online. Varje enskild persons sexualitet är individuell: vi har olika mycket lust att ha sex och vi tycker om olika typer av sex.

Är det okej att jag frågar om din sexuella hälsa?

De två första frågorna gäller hur det varit det senaste året.

Njutning

Hur nöjd är du med ditt sexliv på en skala från 0-3 där 0 står för ”missnöjd” och 3 för ”nöjd”?

- Missnöjd (önskar stor förändring) 0 poäng
- Ganska missnöjd (finns önskemål om förändring) 1 poäng

Etikprövningsmyndigheten
2022-02-26
2022-05-18
2022-02-26-01-269987

- Ganska nöjd (trivs bra men det finns önskemål om liten förändring) 2 poäng
- Nöjd (önskar inga/endast marginella förändringar) 3 poäng

Smärta, sjukdom och hinder

Är sex svårt för dig: upplever du smärta, har du någon sjukdom eller något annat hinder som påverkar din möjlighet att ha sex?

- Ja, min förmåga att ha sex påverkas negativt av detta 0 poäng
- Ja, men jag är nöjd som det är ändå 3 poäng
- Nej, min förmåga att ha sex hindras inte av detta 3 poäng

Trygghet

Har du någonsin varit med om sex som på något sätt har varit jobbigt, till exempel våldsamt, kränkande eller utan samtycke? Här räknas såväl om du själv varit utsatt eller utsatt andra.

- Ja och jag har inte fått hjälp eller stöd 0 poäng
- Nej, men jag vill gärna få hjälp att känna mig mer trygg 1 poäng
- Ja, men jag har fått hjälp och behöver ingen 3 poäng
- Nej 3 poäng

Etikprövningsmyndigheten
2022-02226-01-269987
2022-05-18

Droger

Med droger menar vi både olagliga droger och preparat som har ett medicinskt syfte men som används för andra syften.

Är det okej att jag frågar om dina drogvanor?

Användande

Har du någonsin tagit en drog eller missbrukat medicinska preparat (ej alkohol) och i så fall, när använde du droger senast?

- Ja, för mindre än två veckor sedan 0 poäng
- Ja, för mindre än tre månader sedan 1 poäng

- Ja, för mindre än ett år sedan 2 poäng
- Nej, alternativt mer än ett år sedan 3 poäng

Kvantitet

Hur ofta under det senaste året har du använt droger eller missbrukat medicinska preparat?

- 2-3 gånger i veckan eller mer 0 poäng
- 2-4 gånger i månaden 0 poäng
- En gång i månaden eller mer sällan 1 poäng
- Aldrig 3 poäng

Skärmtid

Skärmtid kan vara att spela spel, använda sociala medier, att surfa runt på internet och titta på TV/film/serier. Skärmtid kan vara utvecklande och kul men ska inte gå ut över andra levnadsvanor.

Är det okej att vi frågar om din skärmtid?

Känner du igen dig i följande påståenden?

- **Att andra tycker att du har för mycket skärmtid**.
- **Att det är svårt att sätta gränser och att följa de gränser du sätter.**
- **Att du känner att din skärmtid leder till att du inte gör saker du borde göra (exempelvis sova, studera, jobba, intressen, fysisk aktivitet, träffa vänner).**
- **Att du använder skärm för att fly från verkligheten/slippa negativa känslor.**

- Känner igen mig i alla påståenden 0 poäng
- Känner igen mig i flera påståenden 1 poäng
- Känner igen mig ett påstående 2 poäng
- Känner inte igen mig i något påstående 3 poäng

Se översiktsfigur och vidare instruktioner för att tolka och rapportera resultaten på nästa sida!

Etikprövningsmyndigheten
2022-02-26
2022-05-18
2022-02-26-01-269987

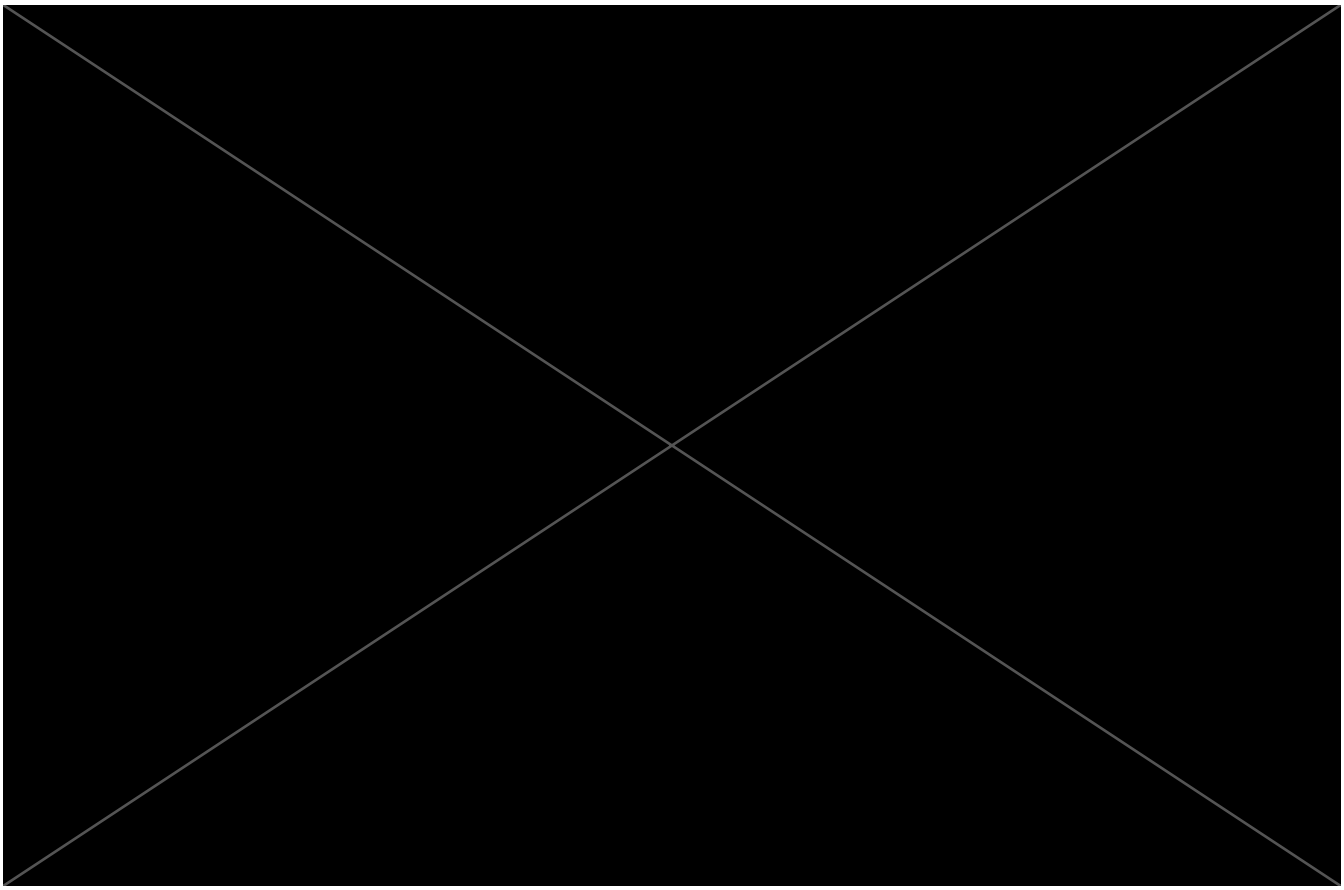

Etikprövningsmyndigheten
2022-02926-01-269987
2022-05-18

Återkoppling av resultat till deltagare

Gör en markering längst strecket för varje levnadsvana baserat på den poäng/nivå som deltagaren fick enligt Lev-s. Till din hjälp finns en Excelmall som sammanställer poängen.

Här ser du hur dina levnadsvanor ser ut just nu (peka på de små prickarna). **Levnadsvanor är inte samma sak som din personlighet. Det är vanor du har just nu. De flesta av oss har ohälsosamma levnadsvanor inom något område. Det varierar även genom livet hur väl vi lyckas prioritera det hälsosamma vanorna.**

Grönt betyder att den levnadsvanan är hälsosam.

Gult betyder att det finns risk för ohälsa. Du kan må bättre genom att ändra på dessa vanor.

Rött betyder hög risk för ohälsa. Den här levnadsvanan gör att du antagligen mår eller kommer må dåligt i framtiden. Det positiva är att du har mycket att vinna på att ta tag i röda områden.

Var resultatet som du hade förväntat dig? Vissa kan bli nedslagna av många röda fält. Upprepa att dessa poäng inte säger något om deltagarens personlighet utan att de är tillfälliga vanor. Betona att det är bra att de tagit tag i levnadsvanorna och att de kommit till rätt plats för att få stöd.

De flesta av oss behöver stöd för att ändra på våra levnadsvanor. Skulle du vilja ha vårt stöd i arbetet med att skapa hälsosammare levnadsvanor?

Journalför resultat utifrån färgskalan ovan samt om Lev ska genomföras.

Oavsett om deltagaren säger ja eller nej har du som behandlare gjort mycket genom att ta upp frågan. Många gånger blir det startskottet för en förändring lite längre fram. Tänk på att en del väljer att förändra på egen hand eller söka stöd på annat håll, medan andra kan behöva tid och kanske är mer mottagliga vid ett annat tillfälle.

Skattning av måluppfyllelse:

	Mål 1 (Huvudmål)	Mål 2	Mål 3
**När målet ska vara uppnått**	Inom 6 månader	Inom 6 månader	Inom 6 månader
**Initial nivå (0 p)**	Röker på jobbet	Lägger sig olika tider alla dagar	Äter kött 5 dagar i veckan och aldrig fisk
**Utveckling (1 p)**	Minskat rökning på jobbet	Lägger sig samma tid ca 3 dagar i veckan	Äter fisk en gång i och kött 4 dagar i veckan
**Mål uppfyllt (2 p)**	Röker inte på jobbet	Går och lägger sig samma tid på vardagar	Äter kött max 2 dagar i veckan. Äter fisk minst två dagar i veckan.
**Mer än väntat (3 p)**	Röker inte på jobbet och har minskat på rökning även i andra miljöer	Går och lägger sig samma tid på vardagar och går även upp samma tid	Äter kött en gång i och fisk två.
**Mycket mer än väntat (4 p)**	Är rökfri	Går även och lägger sig samma tid på de flesta helger	Äter kött en gång i och fisk två och har minskat på sötsaker.

Poängen bedöms baserat på huvudmålet på skalan 0-4.

I tabellen finns exempel på hur målen skulle kunna se ut för 3 olika levnadsvanor.

Fylls i av behandlare.

Etikprövningsmyndigheten
2022-02920-01-269988
2022-05-23

TREATMENT CREDIBILITY SCALE-BEHANDLARE

Yrkeskategori: ……**Datum**: ……..……

1. Hur logisk/rimlig/vettig tycker att den här insatsen (Lev) verkar?

_____

1 2 3 4 5 6 7 8 9 10

Inte alls logisk Mycket logisk

2. Hur säker är du på att den här insatsen kommer vara framgångsrik i att få deltagaren att leva hälsosammare?

_____

1 2 3 4 5 6 7 8 9 10

Inte alls säker Mycket säker

3. Med vilken grad av tillit skulle du rekommendera den här insatsen till en någon som vill leva hälsosammare?

_____

1 2 3 4 5 6 7 8 9 10

Låg grad av tillit Hög grad av tillit

4. Hur framgångsrik tror du att den här typen av insats skulle vara för personer med fysisk funktionsnedsättning?

_____

1 2 3 4 5 6 7 8 9 10

Inte alls framgångsrik Mycket framgångsrik

5. Hur förbättrad (individuell måluppfyllelse) förväntar du dig att deltagarna blir av den här av insatsen?

_____

1 2 3 4 5 6 7 8 9 10

Inte alls förbättrad Mycket förbättrad

Etikprövningsmyndigheten
2022-02920-01-269989
2022-05-23

Treatment Excpectancy/credibility questionnaire. Frågorna 1-5 är från början översatta och modifierade av professor Lars-Göran Öst och sedan modifierade för den aktuella studien.
Referens till ursprungsformuläret: Borkovec, T.D., & Nau, S.D. (1972). Credibility of analogue therapy rationales. Journal of Behavior Therapy and Experimental Psychiatry, 3(4), 257-260.

TREATMENT CREDIBILITY SCALE - DELTAGARE

Namn……Personnummer…….

1. Hur logisk/rimlig/vettig tycker att den här insatsen (Lev) verkar?

_____

1 2 3 4 5 6 7 8 9 10

Inte alls logisk Mycket logisk

2. Hur säker är du på att den här insatsen kommer vara framgångsrik i att få dig att leva hälsosammare?

_____

1 2 3 4 5 6 7 8 9 10

Inte alls säker Mycket säker

3. Med vilken grad av tillit skulle du rekommendera den insatsen till en vän som vill ändra samma levnadsvanor som du vill ändra på?

_____

1 2 3 4 5 6 7 8 9 10

Låg grad av tillit Hög grad av tillit

4. Hur framgångsrik tror du att den här insatsen skulle vara för att ändra andra levnadsvanor?

_____

1 2 3 4 5 6 7 8 9 10

Inte alls framgångsrik Mycket framgångsrik

5. Hur förbättrad förväntar du dig att bli av den här insatsen?

_____

1 2 3 4 5 6 7 8 9 10

Inte alls förbättrad Mycket förbättrad

Etikprövningsmyndigheten
2022-02920-01-269990
2022-05-23

Treatment Excpectancy/credibility questionnaire. Frågorna 1-5 är från början översatta och modifierade av professor Lars-Göran Öst. Referens till ursprungsformuläret: Borkovec, T.D., & Nau, S.D. (1972). Credibility of analogue therapy rationales. Journal of Behavior Therapy and Experimental Psychiatry, 3(4), 257-260.

Lev – Utvärdering varje tillfälle

Utvärdering av dagens möte. Möte…….

Namn……Personnummer…….……..

1. **Efter mötet idag har mina kunskaper om hur jag kan ändra mina levnadsvanor ökat**

0	1	2	3	4
inte alls	tveksamt	varken/eller	till viss del	ja, absolut

2. **Jag kommer att ha nytta av det vi gick igenom under mötet idag**

0	1	2	3	4
inte alls	tveksamt	varken/eller	till viss del	ja, absolut

3. **Mötets innehåll kändes relevant utifrån mina egna erfarenheter**

0	1	2	3	4
inte alls	tveksamt	varken/eller	till viss del	ja, absolut

4. **Jag förstod de råd och strategier jag fick som ska hjälpa min ändra mina levnadsvanor**

0	1	2	3	4
inte alls	tveksamt	varken/eller	till viss del	ja, absolut

5. **Jag känner mig säker på hur jag ska använda råden och strategierna**

0	1	2	3	4
inte alls	tveksamt	varken/eller	till viss del	ja, absolut

6. **Jag kommer använda råden och strategierna**

0	1	2	3	4
inte alls	tveksamt	varken/eller	till viss del	ja, absolut

7. **Jag blev lyssnad på behandlaren förstod min situation**

0	1	2	3	4
inte alls	tveksamt	varken/eller	till viss del	ja, absolut

8. **Jag var delaktig och bidrog till att komma med förslag för hur jag kan nå mina mål**

0	1	2	3	4
inte alls	tveksamt	varken/eller	till viss del	ja, absolut

Etikprövningsmyndigheten
2022-02920-01-269995
2022-05-23

Är det något annat du vill framföra?

_____

_____

_____

_____

Tack!

Etikprövningsmyndigheten
2022-02920-01-269995
2022-05-23

Lev – Utvärdering varje tillfälle

Utvärdering av dagens möte. Möte…….

Profession……

1. **Innehållet på denna session känns relevant**

0	1	2	3	4
inte alls	tveksamt	varken/eller	till viss del	ja, absolut

2. **Svårighetsgraden gällande att förmedla sessionens innehåll är på en lagom nivå för mig som behandlare**

0	1	2	3	4
inte alls	tveksamt	varken/eller	till viss del	ja, absolut

Är det något annat du vill framföra?

_____

_____

_____

_____

_____

_____

_____

Tack!

Etikprövningsmyndigheten
2022-02920-01-270011
2022-05-23

Lev – Utvärdering helhet

Utvärdering av Lev som helhet.

Namn……Personnummer…….

1. **Som helhet har insatsen varit tydligt inriktad på hur jag kan leva hälsosammare**

0 1 2 3 4
inte alls Ja, absolut

2. **Mina kunskaper om hur jag kan ha hälsosammare levandsvanor har ökat**

0 1 2 3 4
inte alls Ja, absolut

3. **Jag kan bättre hantera problem som är kopplade till mina levnadsvanor**

0 1 2 3 4
inte alls Ja, absolut

4. **Jag har haft möjlighet att komma med egna synpunkter under insatsen**

0 1 2 3 4
inte alls Ja, absolut

5. **Jag kan tänka mig att delta i en liknande insats i framtiden**

0 1 2 3 4
inte alls Ja, absolut

6. **Jag tycker att deltagandet i insatsen hjälpte mig på det här sättet:**

_____

_____

_____

_____

7. **Mitt slutbetyg för insatsen som helhet**

Etikprövningsmyndigheten
2022-02920-01-270014
2022-05-23

Ej godkänt	Godkänt	Väl godkänt	Mycket väl godkänt
☐	☐	☐	☐

8. **Allmänt välbefinnande och hälsa: ringa in den siffra som närmast beskriver ditt tillstånd före insatsen**

1	2	3	4	5	6	7	8	9	10
mycket dåligt									mycket bra

9. **Allmänt välbefinnande och hälsa: ringa in den siffra som närmast beskriver ditt tillstånd efter insatsen**

1	2	3	4	5	6	7	8	9	10
mycket dåligt									mycket bra

10. **Hur kunde insatsen blivit bättre?**

_____

_____

_____

_____

11. **Vad skulle jag själv ha kunnat göra på ett annat sätt?**

_____

_____

_____

_____

12. **Är det något annat du vill framföra?**

Etikprövningsmyndigheten
2022-02920-01-270014
2022-05-23

Tack för ditt utvärdering av deltagande i Lev!

Etikprövningsmyndigheten
2022-02920-01-270014
2022-05-23

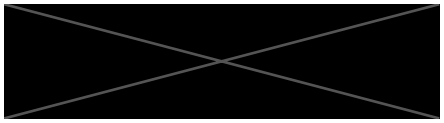

Instruktioner

Frågorna i detta formulär handlar om vad Du anser om din livskvalitet, Din hälsa och andra områden i Ditt liv. **Var snäll och besvara alla frågor**. Om du är osäker på hur Du skall besvara en fråga, **välj då det svar** som passar bäst. Det kan ofta vara det första Du tänkte på.

Utgå från dina normer, förhoppningar, glädjeämnen och oroskällor. När Du svarar ber vi Dig att Du bara tänker på **de senaste två veckorna** i Ditt liv. Till exempel, när du tänker på de två senaste veckorna, en fråga skulle kunna vara:

		Inte alls	Lite	Måttligt	I hög utsträckning	Helt och hållet
	Får du det stöd som du behöver av andra människor?	1	2	3	4	5

Etikprövningsmyndigheten
2022-02920-01-270016
2022-05-23

Du skall ringa in den siffra som bäst passar in på hur mycket stöd du fått av andra under de två senaste veckorna. Alltså, om du fick stöd i hög utsträckning av andra utifrån dina behov så skall du ringa in siffran 4 och om du inte alls fått det stöd du behöver av andra så skall du ringa in siffran 1.

Var vänlig läs varje fråga, känn efter, och ringa sen in den siffra på skalan som bäst motsvarar Ditt svar.

Tack för hjälpen

		Väldigt dåligt	Dåligt	Varken bra eller dåligt	Bra	Väldigt bra
1 (G1)	Hur värderar du din livskvalitet?	1	2	3	4	5

		Väldigt missnöjd	Missnöjd	Varken nöjd eller missnöjd	Nöjd	Väldigt nöjd
2 (G4)	Hur nöjd är du med din hälsa?	1	2	3	4	5

De följande frågorna handlar om **i vilken utsträckning/hur mycket** du har upplevt speciella saker de senaste två veckorna.

		Inte alls	Lite	Måttligt	I hög utsträckning	I extremt hög utsträckning
3 (F1.4)	I vilken utsträckning tycker du att du hindras från att göra sådant som du måste göra därför att du har värk/smärta?	1	2	3	4	5
4 (F11.3)	I vilken utsträckning behöver du medicinsk behandling av något slag för att kunna fungera i det dagliga livet?	1	2	3	4	5
5 (F4.1)	I vilken utsträckning gläder du dig åt livet?	1	2	3	4	5
6 (F24.2)	I vilken utsträckning upplever du att ditt liv är meningsfullt?	1	2	3	4	5
7 (F5.3)	Hur bra kan du koncentrera dig?	1	2	3	4	5
8 (F16.1)	Hur trygg känner du dig i vardagslivet?	1	2	3	4	5
9 (F22.1)	Hur hälsosam är den yttre miljön du lever i?	1	2	3	4	5

Etikprövningsmyndigheten
2022-02920-01-2700010
2022-05-23

De följande frågorna handlar om **i vilken omfattning** du har upplevt eller haft möjlighet att utföra vissa saker de senaste två veckorna

		Inte alls	Lite	Måttligt	I hög utsträckning	Helt och hållet
10 (F2.1)	Har du tillräcklig energi/ork för vardagslivet?	1	2	3	4	5
11 (F7.1)	Kan du acceptera hur din kropp ser ut?	1	2	3	4	5
12 (F18.1)	Har du tillräckligt med pengar för det du behöver?	1	2	3	4	5
13 (F20.1)	Hur pass tillgänglig är den information som du behöver i ditt dagliga liv?	1	2	3	4	5
14 (F21.1)	I vilken utsträckning har du möjlighet att ägna dig åt fritidsaktiviteter?	1	2	3	4	5

		Inte alls	Lite	Måttligt	I hög utsträckning	I extremt hög utsträckning
15 (F9.1)	Hur bra kan du förflytta dig?	1	2	3	4	5

Etikprövningsmyndigheten
2022-02920-01-2
2022-05-23

De följande frågorna handlar om hur **tillfredsställd, glad eller nöjd** du har känt dig över olika saker i ditt liv under de senaste två veckorna.

		Väldigt missnöjd	Missnöjd	Varken nöjd eller missnöjd	Nöjd	Väldigt nöjd
16 (F3.3)	Hur nöjd är du med din sömn?	1	2	3	4	5
17 (F10.3)	Hur nöjd är du med din förmåga att utföra dagliga aktiviteter?	1	2	3	4	5
18 (F12.4)	Hur nöjd är du med din arbetskapacitet?	1	2	3	4	5
19 (F6.3)	Hur nöjd är du med dig själv?	1	2	3	4	5
20 (F13.3)	Hur nöjd är du med ditt förhållande till andra människor?	1	2	3	4	5
21 (F15.3)	Hur nöjd är du med ditt sexliv?	1	2	3	4	5
22 (F14.4)	Hur nöjd är du med det stöd som dina vänner ger dig?	1	2	3	4	5
23 (F17.3)	Hur nöjd är du med förhållandena där du bor?	1	2	3	4	5
24 (F19.3)	Hur nöjd är du med dina möjligheter att få tillgång till hälsovård?	1	2	3	4	5
25 (F23.3)	Hur nöjd är du med de transportmedel som står till buds?	1	2	3	4	5

Frågan nedan handlar om **hur ofta** du känt eller upplevt vissa saker under de senaste två veckorna

		Aldrig	Sällan	Ganska ofta	Väldigt ofta	Alltid
26 (F8.1)	Hur ofta upplever du negativa känslor, t.ex. nedstämdhet, förtvivlan, ångest, depression?	1	2	3	4	5

Har du några kommentarer till detta frågeformulär?

Etikprövningsmyndigheten
2022-02920-01-270016
2022-05-23

TACK FÖR DIN MEDVERKAN

For more info contact: Prof Martin Eisemann, Dept of Psychology, Tromsø University, 9037 Tromsø(Norway), email: martin.eisemann@psyk.uit.no

This translation was not created by the World Health Organization (WHO). WHO is not responsible for the content or accuracy of this translation. In the event of any inconsistency between the English and the translated version, the original English version shall be the binding and authentic version.

CV Douglas Sjöwall

Degrees

2007 Master level media and communication, JMK, Stockholm University
2010 Master level in Psychology, Stockholm University
2015 PhD program, Karolinska Institutet (KI)

2015-01-23, KI, Psychology, Title: "Attention Deficit Hyperactivity Disorder in Children and Adolescents: Neuropsychological Deficits and Functional Outcomes"
https://openarchive.ki.se/xmlui/handle/10616/42334

Post docs

2015 – 2016 Postdoc, Dept of Clinical Neuroscience, Division for Psychology, KI
2017 – 2019 Postdoc, Dept of Neuroscience, KI

Current affiliations

- Project leader at research and development unit at Habilitation & Health, Region Stockholm.
- Department of Women's and Children's Health, Pediatric Neuropsychiatry Unit, Center for Neurodevelopmental Disorders at Karolinska Institutet (KIND), Karolinska Institutet, Stockholm, Sweden.
- Centre for Psychiatry Research, Region Stockholm.

Etikprövningsmyndigheten
2022-02920-01-266294
2022-05-23

Supervision of doctoral students

Nathaniel Hidalgo (Started 2020)
Hanna Agius (Started 2021)

Selected academic distinctions

- 2014 – Research article (Sjöwall et al., 2013) selected by Journal of Child Psychology and Psychiatry (JCPP) as being one of the 30 most influential articles addressing neuropsychology and ADHD (DOI: 10.1111/jcpp.12360)

SCIENTIFIC PUBLICATIONS

https://scholar.google.com/citations?user=S_b32IoAAAAJ&hl=en

Cited by		VIEW ALL
	All	Since 2017
Citations	793	609
h-index	10	10
i10-index	10	10

List of all original works

1. **Sjöwall, D**., Roth, L., Lindqvist, S., & Thorell, L.B. (2013). Multiple Deficits in ADHD: Executive Dysfunction, Delay Aversion, Reaction Time Variability and Emotional Deficits, Journal of Child Psychology and Psychiatry, 54, 619–627
2. **Sjöwall, D.,** & Thorell, L.B. (2014). Functional impairments in Attention-Deficit/Hyperactivity Disorder: The mediating role of neuropsychological functioning, *Developmental Neuropsychology, 39*, 187-204
3. **Sjöwall, D**., Backman, A., & Thorell, L.B. (2015). Neuropsychological heterogeneity in preschool ADHD: Investigating the interplay between cognitive, affective and motivation-based forms of regulation. *Journal of Abnormal Child Psychology,* 43, 669–680
4. **Sjöwall, D**., Bohlin, G., Rydell, A-M., & Thorell, L.B. (2017). Neuropsychological deficits in preschool as predictors of ADHD symptoms and academic achievement in late adolescence, *Child Neuropsychology, 23,* 111-128
5. Thorell, L.B., **Sjöwall, D**., Rydell, A-M., Diamantopoulou, S., & Bohlin, G. (2017). Emotion Regulation Difficulties, ADHD Symptoms and Peer Problems: Longitudinal Relations in Children age 6-9 ½*, Infant and Child Development,* 26, e2008.
6. Thorell, L.B., **Sjöwall, D**., Mies, G.W., & Scheres, A. (2017). Quick Delay Questionnaire: Psychometric Properties, Relations to Functional Impairments and Discrimination between Adults with ADHD and Both Clinical and Non-clinical Controls. *Psychological Assessment, 29,* 1261-1272.
7. Thorell, L.B., Holst, Y., Chistiansen, H., J.J Sandra Kooij, S.J.J., Bijlenga, D., & **Sjöwall, D.** (2017). Neuropsychological Deficits in Adults Age 60 and Above with Attention Deficit Hyperactivity Disorder. *European Psychiatry, 45,* 90-96.
8. **Sjöwall, D**. & Thorell, L.B. (2019). A critical appraisal of the role of neuropsychological deficits in preschool ADHD. *Child Neuropsychology, 25*, 60-68.
9. **Sjöwall D,** Hertz M, Klingberg T. (2017). No long-term effect of physical activity intervention on working memory or arithmetic in preadolescents. *Front Psychol 8*:1342.
10. Thorell, L, Holst, Y. & **Sjöwall, D**. (2019). Quality of life in older adults with ADHD: links to ADHD symptom levels and executive functioning deficits, *Nordic Journal of Psychiatry, 73*, 409-416. DOI: 10.1080/08039488.2019.1646804
11. **Sjöwall, D**., Thorell, L., Mandic, M & Westerstål, M. (2019) No effects of a long-term physical activity intervention on executive functioning among adolescents. *Open medicine, 4,* eCollection 2019.
12. **Sjöwall, D.,** Thorell, L. (2019). Neuropsychological deficits in relation to ADHD symptoms, quality of life and daily life functioning in young adulthood. *Applied Neuropsychology: Adult. 27,* 1-9
13. Thorell, L., Tilling H. & **Sjöwall, D.** (2020). Emotion dysregulation in adult ADHD: Introducing the Comprehensive Emotion Regulation Inventory (CERI). *Journal of clinical and experimental neuropsychology.* 42:7.
14. Judd, N., & Klingberg, T., & **Sjöwall, D. (**2021). Working memory capacity, variability and response to intervention at age 6 and its association to inattention and mathematics age 9. Cognitive development, 58.
15. Balan., A., & **Sjöwall, D.** (2022). Evaluation of an extensive deliberate practice and growth mindset intervention in 7th-grade students. *Scandinavian Journal of Educational Research.* Accepted
16. Peny-Dahlstrand, M., Hofgren, C., Lindquist, B., Lena Bergqvist, L., Himmelmann, K., Opheim, A., **Sjöwall, D.**, Brock, K., Öhrvall, A-M. (2022) The Cognitive Orientation to daily Occupational Performance (CO-OP) Approach is superior to ordinary treatment for achievement of goals and transfer effects in children with cerebral palsy and spina bifida- a randomized controlled trial. *Disability and rehabilitation.* Accepted

Etikprövningsmyndigheten
2022-02920-01-266294
2022-05-23

Screening Lev

Namn på deltagaren……

Intervjuare……

Du har anmält intresse att delta i Lev (Insats för hälsosammare levnadsvanor). Jag tänkte först berätta lite om Lev och sedan tänkte jag ställa några frågor om din nuvarande situation så att vi kan avgöra om det är en lämplig insats för dig.

Låter det okej?

Bakgrund och syfte
Lev har tagits fram för att våra levnadsvanor påverkar vår psykiska och fysiska hälsa. Personer med funktionsnedsättning har många gånger ohälsosammare levnadsvanor. Vi tror att en av anledningarna till det är att de insatser som finns idag inte är anpassade efter personer med olika funktionsnedsättningar. Vi har därför, tillsammans med representanter för olika funktionsnedsättningar, utvecklat en metod som ska vara bättre på det.

Utvärdering
För att vi ska veta att insatsen uppskattas dig och andra som får ta del av den, genomför vi en forskningsstudie. Det innebär att du får svara på frågor före, under och efter insatsen som gör att vi kan utvärdera och få information om hur vi kan förbättra insatsen. Vi ställer om dina levnadsvanor, vad du tycker om de olika delarna av behandlingen och din bakgrund (exempelvis utbildning, diagnos och funktionsnedsättning).

Låter det okej?

Etikprövningsmyndigheten
2022-02920-01-266721
2022-05-23

Ditt nuvarande mående
Nu kommer jag fråga lite om din situation och ditt mående för att vi tillsammans ska kunna bedöma om Lev är rätt för sig just nu.

Känner nedstämd och orolig i vardagen?

OM JA:

Upplever du så pass mycket nedstämdhet/oro/stress i din vardag att det ställer till det för dig? Hur?

Upplever du att du mår så pass dåligt att du har tankar på att du inte vill leva (ta ditt liv)?

OM JA:

Jag hör att du rapporterar så pass mycket nedstämdhet att jag föreslår att du kommer för en fördjupad bedömning på mottagningen. (Boka besök hos läkare/psykolog/lämplig personal för fortsatt bedömning). Vi tar ställning till ditt eventuella deltagande efter besöket.

OM NEJ:

Då är nästa steg att vi bokar in dig för det första mötet. På det mötet fyller du läser och fyller i detta smycke om du inte redan gjort det. Vi genomför kartläggningen av dina levnadsvanor och du får fylla i några formulär som tillhör forskningsstudien.

Genomförande
Vi kommer att ses på en mottagning eller via videomöten. Vi kommer att ha minst 4 möten på tider som passar dig. Dessa möten kommer vara utspridda över 2-6 månader.

Har du några frågor?

Vid ytterligare frågor som du inte kan svara på kan du hänvisa till Douglas Sjöwall som är ansvarig för studien: douglas.sjowall@regionstockholm.se

Bedömer du att deltagaren kan gå vidare och göra Lev?

Ja
Nej
Om nej; anledning……

Etikprövningsmyndigheten
2022-02920-01-266721
2022-05-23

2022

Etikprövningsmyndigheten
2022-02-10 2022-01-266722
2022-05-09

Lev - Insats för hälsosammare levnadsvanor: teoretisk bakgrund

EN TRANSDIAGNOSTISK INSATS FÖR VUXNA MED FUNKTIONSNEDSÄTTNING

HABILITERING & HÄLSA, REGION STOCKHOLM

Innehåll

Förord....2
Del I....4
Inledning....4
Riktlinjer....4
Riskgrupper....5
Stegvis vård....5
Vårdprocesser....7
Definition av levnadsvanor....8
Definition av Hälsa....9
Hur levnadsvanor påverkar hälsa....9
Funktionsnedsättning och levnadsvanor....10
Utveckling av Lev....11
Teorier och antaganden om hur insatsen ska fungera....12
Beteendestödsplan....19
Samverkan....20
Uppföljning....20
Referenser....21
Syfte....22
Övergripande målsättning för deltagare....22
Övergripande målsättning för behandlare....22
Övergripande principer....22
Del 2....23
Det praktiska genomförandet av Lev....23
Behandlarens roll....23
Vem han ge insatsen?....23
För vilka är detta en lämplig insats?....23
Upplägget för Lev....24
Utrustning....24
Deltagarmaterial....24
Åtgärdsplan....25
....27
Del 3....27
Utbildning....27

Etikprövningsmyndigheten
2022-02-10
2022-05-09
2022-01-266722

Förord

”Lev – Insats för hälsosammare levnadsvanor”, har utformats på initiativ av Habilitering & Hälsa, Region Stockholm. Lev syftar till att implementera Socialstyrelsen ”Nationella riktlinjer för prevention och behandling vid ohälsosamma levnadsvanor” samt Nationellt vårdprogram för ohälsosamma levnadsvanor – prevention och behandling (2022). Lev har också tagit inspiration från FNs ”Sustainable Developmental Goals” 2030, mål 3: “Ensure healthy lives and promote well-being for all at all ages”. Inom alla dessa tre riktlinjer framgår det tydligt att en särskild, mer omfattande satsning, måste ske för personer med funktionsnedsättning. Det framgår också att levnadsvanor måste prioriteras högre än vad det gör idag och att preventiva arbetssätt måste blir vanligare.

Likt Socialstyrelsen fokuserar Lev på Tobak, Alkohol, kost och fysisk aktivitet. Utöver detta tas ytterligare 6 levnadsvanor upp. Anledningen för att ta upp fler områden är att levnadsvanor påverkar varandra (Nationellt vårdprogram). Genom att uppmärksamma flera levnadsvanor kan därför arbetet bli mer effektivt för de fyra områden som Socialstyrelsen prioriterar. De levnadsvanor som tas upp i Lev har alla dokumenterad vetenskaplig effekt på psykisk och/eller fysisk hälsa

Etikprövningsmyndigheten
2022-02-10
Dnr 2022-01-266722
2022-05-09

De råd och arbetssätt som används i Lev följer Nationellt vårdprogram och gäller alla människor. Dock har vi ett tydligare fokus på hur samverkan, samarbete och uppföljning ska ske för att minska bördan på deltagaren. Lev kan därför användas på alla människor som behöver stöd för att förändra sina levnadsvanor men har tagits fram i första hand för personer med funktionsnedsättning.

Ett biopsykosocialt synsätt ligger till grund för insatsen vilket betyder att individens levnadsvana ses som en konsekvens av individuella och kontextuella faktorer. En viktig uppgift för behandlaren är identifiera hur man kan stötta deltagaren utifrån båda dessa perspektiv. En guidande princip för detta arbete ska vara delaktighet. Detta gäller både i arbetet med behandlaren men också i relation till de levnadsvanor som tas upp.

En grundläggande metod i utvecklingen av Lev har varit samverkan mellan olika aktörer: forskare, behandlare, ledningen på Habilitering & Hälsa, Region Stockholm, intresseorganisationer för patienter, regionala programområden (RPO) i Stockholm samt flera vårdaktörer med uppdrag gällande levnadsvanor och hälsa. Vi kommer i förvaltningen av Lev att fortsätta utveckla insatsen allteftersom mer synpunkter kommer in från alla dessa aktörer för att göra insatsen mer genomförbar och effektiv.

Så här använder du manualerna:

Det finns två manualer. *Den teoretiska manualen* (som du läser nu) är det första man läser när man genomför sin utbildning och syftet är att ge en bakgrund till insatsen och en övergripande bild av hur man genomför Lev. I den teoretiska manualen beskrivs även den utbildning som föreslås genomföras innan man börjar använda Lev. Nästa steg är att ta del av ”*Manualen för praktiskt genomförande*”. Där står det ordagrant hur man kan lägga upp mätning och behandling. Denna del är viktig när man tränar för att kunna ge Lev och när man förbereder sig inför mötet med deltagaren. Tanken är inte att man behöver följa den praktiska manualen ordagrant. Den är snarare exempel på hur du kan formulera dig. I stället utgår man ifrån deltagarens arbetsbok. Arbetsboken följer samma struktur som manualen för det praktiska genomförandet men innehåller kortare formuleringar. Här i skriver ni gemensamt ner mål och strategier som ni kommer överens om.

Förhoppningsvis hjälper Lev dig som behandlare att ta initiativ till fler samtal om fler levnadsvanor och även följa upp dessa samtal i högre utsträckning än idag. Man behöver inte vara expert för att kunna ta initiativ till samtal om hälsa. Det viktiga är att man har tillräckliga kunskaper för att lyfta frågan, ge enkla råd och hänvisa till eventuell insats som behövs och följa upp deltagaren och stötta i genomförandet.

Avslutningsvis vill vi tacka alla som varit med och bidragit till Lev. Inte minst alla behandlare på Habilitering och Hälsa, Region Stockholm samt intresseorganisationer för de olika funktionedsättningar som Habiliteringen träffar. Ett stort tack riktas även till alla andra delar av hälso- och sjukvården i Stockholm och runt om i Sverige som varit med och diskuterat hur Lev ska utformas för att bli en genomförbar och effektiv insats som förbättrar levnadsvanor.

Etikprövningsmyndigheten
2022-02-08
2018-01-266722
2022-05-09

Del I

Inledning

Ohälsosamma levnadsvanor påverkar våra stora folksjukdomar och leder till död i förtid (WHO, 2018). Personer som inte röker, äter hälsosamt, är måttligt fysiskt aktiva och har en låg alkoholkonsumtion lever i snitt 14 år längre (Nationellt vårdprogram, 2022) Detta exempel inkluderar inte andra levnadsvanor som också kan bidra till att förlänga livet, förbättra livskvalitén och den psykiska och fysiska hälsan. Potentialen i att leva hälsosamt är inte på något sätt marginell, den är stor.

Ohälsosamma levnadsvanor är vanligt och den stora majoriteten människor har under sitt liv ohälsosamma levnadsvanor som ger upphov till sänt livskvalitet och psykiskt och fysiskt lidande. Att ha en funktionsnedsättning innebär ännu högre risk för ohälsosamma levnadsvanor (WHO, 2020). De allra flesta människor vet att de borde leva hälsosammare men det är svårt att omsätta det i förändrade beteenden. Många gånger är det svårt att prioritera det som är bäst på lång sikt framför det som känns bäst i stunden. Det kan handla om att minska eller avstå från alkohol, tobak eller onyttig mat som skadligt på lång sikt. Att ha hälsosamma levnadsvanor är inte bara att avstå lockelser, det är också att leva fullt ut och vara aktiv. Det kan handla om att odla sina intressen, vara sexuellt aktiv och att känna samhörighet med vänner. En viktig utgångspunkt i Lev är att försöka förmedla att levnadsvanor är möjligheter att fylla på med energi och livsglädje.

Etikprövningsmyndigheten
2022-02-10
2022-01-266722
2022-05-09

Riktlinjer

Utformandet av Lev tar avstamp i Socialstyrelsens Nationella riktlinjer för prevention och behandling vid ohälsosamma levnadsvanor (2018) Nationellt vårdprogram vid ohälsosamma levnadsvanor, Ohälsosamma levnadsvanor – prevention och behandling 2022. I de nationella riktlinjerna framgår det att hälso- och sjukvård ska jobba både preventivt och behandlande. Till skillnad från dessa riktlinjer inkluderar Lev fler levnadsvanor. Detta motiveras av att det finns forskningsstöd för hur de inkluderade levnadsvanorna påverkar hälsa. Levnadsvanorna påverkar även varandra och ett antagande bakom Lev är att möjligheten att adressera fler levnadsvanor ökar vår möjlighet att vara ett stöd för deltagaren. Dessutom kan det vara så att motivationen inte finns att jobba med de fyra prioriterade levnadsvanorna och genom att erbjuda fler kan vi bättre anpassa oss efter individens behov.

Manualen innehåller inget rangordningssystem för vilket område som ska prioriteras först. Vi uppmanar varje hälso- och sjukvårdsaktör att löpande ta del av Socialstyrelsens och Nationellt vårdprograms riktlinjer. Det går utmärkt att bara fokusera de prioriterade områdena, tobak, alkohol, kost och fysisk aktivitet men den lokala vårdaktören kan välja att ta fasta på flera levnadsvanor. Arbetetsättet är det samma oavsett vilken

levnadsvana det gäller: uppmärksamma, åtgärda och följ upp. Det är därför lätt att implementera Lev stegvis.

Riskgrupper

Hälso- och sjukvården ska erbjuda stöd till alla som har ohälsosamma levnadsvanor men Socialstyrelsens riktlinjer (2018) och Nationellt vårdprogram (2022) betonar vikten av att fokusera på riskgrupper. Dessa riskgrupper är:

1. Vuxna med särskild risk
    a) Sjukdom (t.ex. diabetes, astma, KOL, cancer, hjärtkärlsjukdom, långvarig smärta, schizofreni, depression)

    b) Fysisk, psykisk eller kognitiv funktionsnedsättning

    c) Social sårbarhet (t.ex. låg socioekonomisk ställning)

    d) Biologiska riskmarkörer (t.ex. högt blodtryck, blodfettsrubbningar, övervikt, fetma)

    e) Andra riskfaktorer (t.ex. flera ohälsosamma levnadsvanor samtidigt, en olycksfallsskada

2. Vuxna som ska genomgå en operation

3. Barn och unga

4. Gravida

5. Äldre

Lev är framtagen för att fungera för personer som har flera av dessa riskprofiler. Dock är den nuvarande manualen utformad för personer över 18 år och som inte är beroende av en medföljare. En viktig utgångspunkt är att fler åtgärder och en mer omfattande uppföljning behöver ske för riskgrupperna i arbetet med levnadsvanor.

Stegvis vård

Att jobba med levnadsvanor har stor potential att minska framtida ohälsa och därmed vårdköer (Socialstyrelsen, 2018). Socialstyrelsen beskriver hur arbetssättet ”uppmärksamma - åtgärder – följ upp” kan ske på tre nivåer (se figur 1 nedan). **Enkla råd** ska kunna ges av all vårdpersonal till deltagare med konstaterade ohälsosamma vanor gällande tobak, alkohol, kost och fysisk aktivitet. Att ge enkla råd motsvarar den minst kunskaps- och tidskrävande åtgärden. Märk väl att även denna nivå inkluderar att man följer upp deltagaren. Nästa nivå är **rådgivande samtal** och det

Etikprövningsmyndigheten
2022-02-10
2022-05-09

omfattas av att behandlaren utför samtalet mer i dialogform och inkluderar motiverande strategier. Uppföljningen kan ske flera gånger vid behov. Den mest omfattande nivån är **kvalificerat rådgivande samtal** och inkluderar att kartläggning (att uppmärksamma), åtgärder och uppföljning sker personcentrerat, teoribaserat och med goda sakkunskaper inom den levnadsvanan man fokuserar på. För att resurserna ska räcka till kan man testa med nivån enkla råd och i uppföljningen bedöma om det är motiverat att fortsätta med rådgivande samtal/kvalificerat rådgivande samtal. Utifrån Nationellt vårdprogram samt ett omfattande internationellt forskningsstöd (WHO, 2020) bör det vara mer regel än undantag att man planerar in minst rådgivande samtal för personer med funktionsnedsättning.

De olika delarna av Lev är utformade för att kunna användas flexibelt. Att ge information är bra men det finns väldigt starkt stöd för att chanserna till förändring av levnadsvanor ökar om deltagaren jobbar aktivt med hemuppgifter och att behandlaren följer upp och uppmärksammar detta arbete.

- **Uppmärksamma/bedömning:** Lev-s innehåller 33 frågor om 10 levnadsvanor. Att ställa dessa frågor och diskutera svaren med deltagaren blir ett första steg i att göra deltagaren medveten om levnadsvanor och vad som är viktiga aspekter att tänka på. Behandlaren får samtidigt en fördjupad bedömning som kan vara värdefull även vid andra insatser.

- **Psykoedukation:** Nästa steg är att ge fas 1 som innehåller generella råd om vikten av hälsosamma levnadsvanor och specifika, standardiserade råd om den levnadsvanan som deltagaren vill fokusera på. I fas 1 får deltagaren uppgifter att jobba med och tips om vidare stöd. Behandlaren följer upp deltagarens hemuppgifter på distans. Denna fas motsvarar enkla råd men är mer omfattande då den även innehåller generella råd om levnadsvanor, målformulering, samt utförs enligt principerna för motiverande samtal.

- **Genomförande:** Fas 2 innebär personcentrerade samtal i dialog baserat på tillämpad beteendeanalys. Denna fas kan repeteras för att underlätta genomförande av målbeteenden. Behandlaren följer upp hemuppgifter på distans. Denna fas är lämplig att ges även om deltagaren hänvisats till annan vårdgivare eller stöd. Detta mer omfattande psykologiska stöd är efterfrågat av flera intresseorganisationer till personer med funktionsnedsättning då kunskapen i vården är ojämn gällande specifika funktionsnedsättningar. Denna fas motsvarar rådgivande samtal. Det som skiljer den från kvalificerat rådgivande samtal är att

Etikprövningsmyndigheten
2022-02-10
2022-05-09
2018-01-266722

behandlaren inte behöver ha fördjupade ämneskunskaper kring den levnadsvana man följer upp.

- **Utvärderingen och vidmakthållande:** Fas 3 innebär att man följer upp deltagarens mål. Behandlaren kan här välja att avsluta insatsen genom att göra en plan för vidmakthållande samt starta upp ett nytt område som deltagaren ska jobba vidare med på egen hand. Möjlighet finns även att genomföra de 3 faserna igen om patientens behov av stöd är stort. Om man valt att implementera alla 10 levnadsvanor i Lev görs även en eftermätning med Lev-s. Att genomföra Lev-s igen blir en nödvändig repetition om att det finns många levnadsvanor som kan bidra till ökad psykisk och fysisk hälsa. Fas tre motsvarar även den rådgivande samtal.

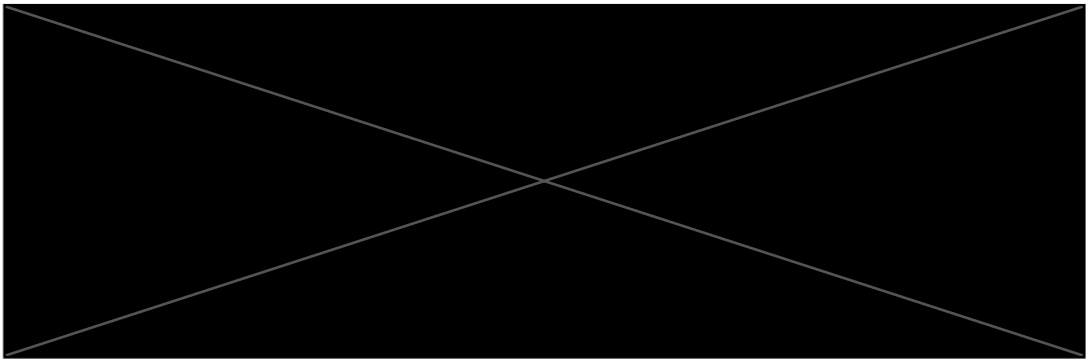

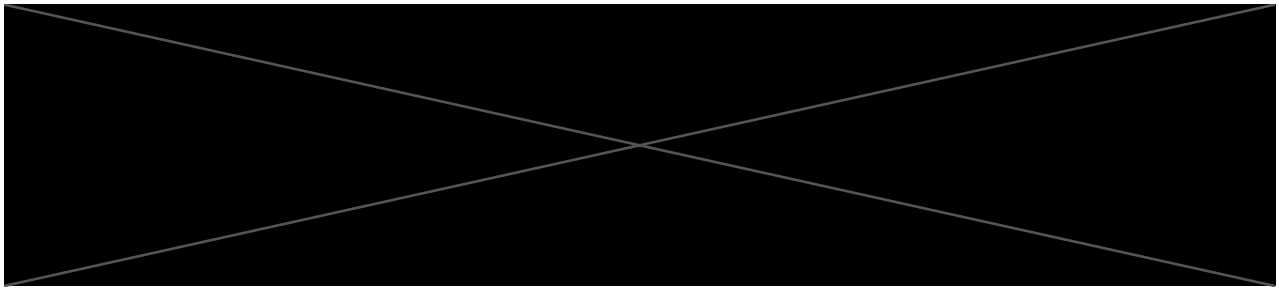

Etikprövningsmyndigheten
2022-02-10
2022-05-09

Vårdprocesser

Lev inkluderar många levnadsvanor och kommer ibland att överlappa med redan etablerade insatser inom vårdorganisationen som står inför att implementerar insatsen. Nedan beskrivs några alternativ för hur man kan använda Lev:

- **Lev används endast för levnadsvanor där insatser inte finns**. Exempel: Om organisationen har en insats för att behandla sömnproblematik fortsätter man som vanligt med det men använder Lev för att kunna behandla problem gällande övriga levnadsvanor som man inte har befintliga insatser för.

- **Lev används utifrån en stegvis vårdmodell**. Exempel: Organisationen har en insats på kvalificerad rådgivande nivå för att behandla sömnproblematik men det är kö till de behandlare som ger insatsen. Lev möjliggör att fler yrkesprofessioner kan behandla

problem med sömnen på rådgivande nivå och kan därför korta tiden till behandlingsstart.

- **Lev hänvisar till extern insats på kvalificerad rådgivande nivå.** Exempel: Efter bedömning med Lev-s och att man genomfört fas 1 hänvisar behandlaren till Sluta-röka-linjen som ger insats på kvalificerat rådgivande nivå. Man följer dock deltagaren och ser till att den externa insatsen fungerar via fas 2 och genomför fas 3 för att få till motivation att jobba med ytterligare en levandsava när sluta-röka-linjens behandling är klar.

Varje vårdorganisation som står inför att implementera Lev behöver bestämma på vilket sätt Lev ska komplettera övriga insatser.

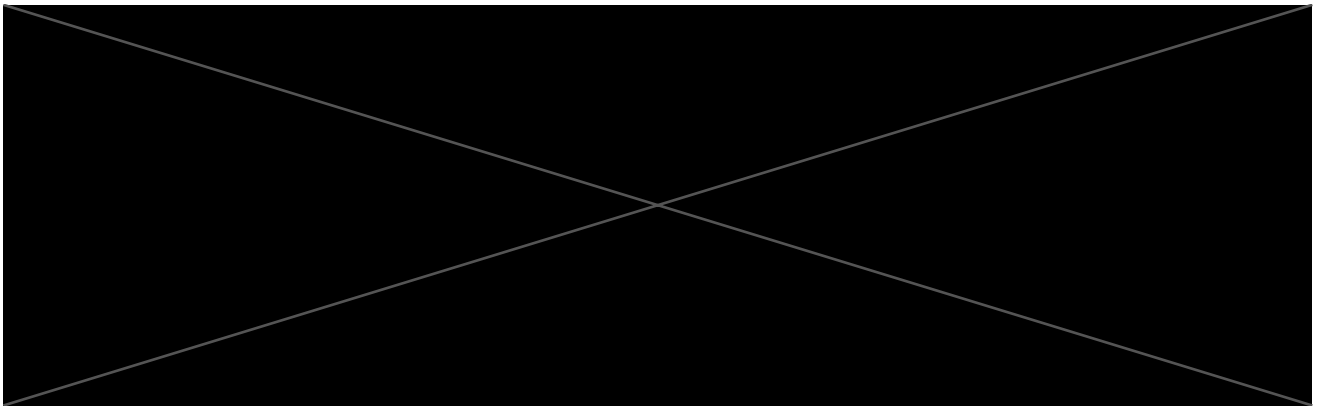

Etikprövningsmyndigheten
2022-02-10
2022-05-09
2018-01-266722

Definition av levnadsvanor

Levnadsvanor är det vi gör varje dag, ofta utan att tänka på det. Levnadsvanor är inte del av personligheten utan saker vi lärt oss och som vi därför kan förändra. Det finns flera olika definitioner av levnadsvanor. I den här insatsen har vi valt 10 levnadsvanor som ger oss möjlighet att på påverkar vår psykiska, fysiska hälsa sociala välbefinnande:

(Illustration av levandasvanorna)

- Fysisk aktivitet: Vi behöver röra på oss för att må bra.
- Tobak, alkohol, droger: Dessa är saker vi ska undvika.
- Kost: Du behöver äta varierat, regelbundet och lagom mycket.
- Sömn: Om du sover lagom mycket.
- Intressen: Vi kan må bättre av att ha roligt och göra något vi gillar.
- Skärmtid: Spel och sociala medier är kul men bör inte ta tid från annat (exempelvis fysisk aktivitet och att träffa vänner ansikte mot ansikte).
- Vänner: Här har vi olika behov men att inte känna sig ensam kan vara väldigt viktigt för hälsan.

- Sexuell hälsa: Även här har vi olika behov men sex kan vara en källa till njutning och det är viktigt att man känner sig trygg och bekväm med sin sexualitet.

Definition av Hälsa

Hälsa är fysiskt, psykiskt och socialt välbefinnande (WHO, 1946). I Lev definierar vi hälsa för deltagaren som att må bra fysiskt (i kroppen) och psykiskt (exempelvis vara glad, lugn) för att göra definitionen av hälsa lättare att förstå. Det sociala välbefinnandet uppmärksammas i stället som en av de 10 levnadsvanorna. Definitionen av hälsa problematiseras senare i insatsen genom att påpeka att det som får oss att må bra i stunden (exempelvis alkohol, tobak, onyttig mat) inte alltid är det som är bra på lång sikt. Även omvänt, det som kan upplevas som en utmaning i stunden (att träna på gym, att våga testa något nytt) kan vara nödvändigt för att må bra på sikt.

Hur levnadsvanor påverkar hälsa

Levnadsvanor kan påverka hälsa på flera olika sätt. Ofta leder psykisk ohälsa till sämre levnadsvanor i en ond spiral (se figur 2 nedan).

Etikprövningsmyndigheten
2022-02-10
2022-05-09

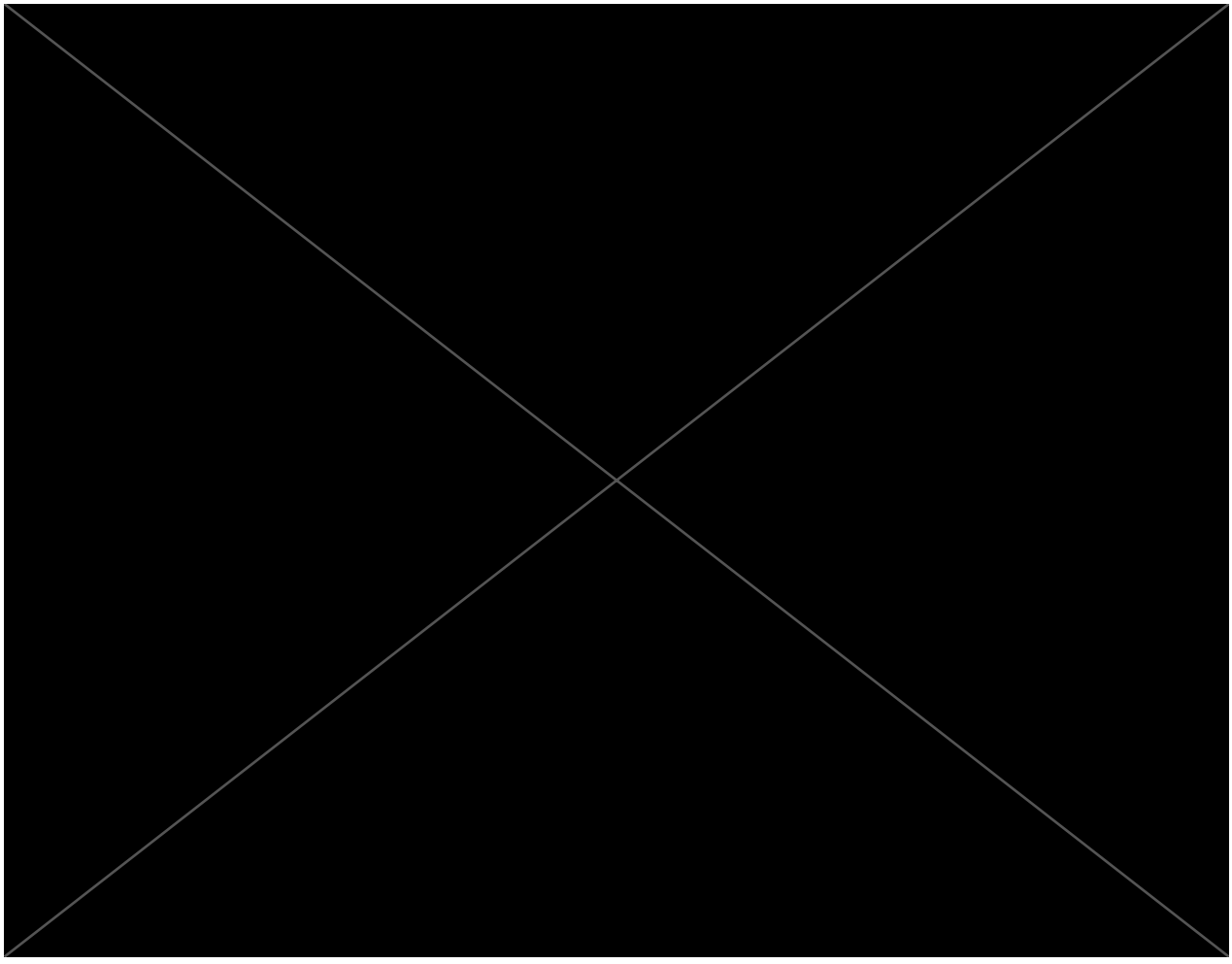

Det är viktigt att hitta sätt att bryta passiviteten så att levnadsvanorna kan bidra till att stärka den psykiska hälsan. Att jobba med levnadsvanor genom så kallad ”beteendeaktivering” är ett vanligt förekommande inslag i Kognitiv beteendeterapi (KBT) och har visat goda effekter på behandling av depression och ångest (Stein et al., 2020). Om man kan bryta den onda spiralen och genomföra fler hälsosamma aktiviteter ökar de möjligheterna för en framtida god fysisk hälsa. Nedan vissas hur positiva förändringar i en levnadsvana kan påverka andra levnadsvanor.

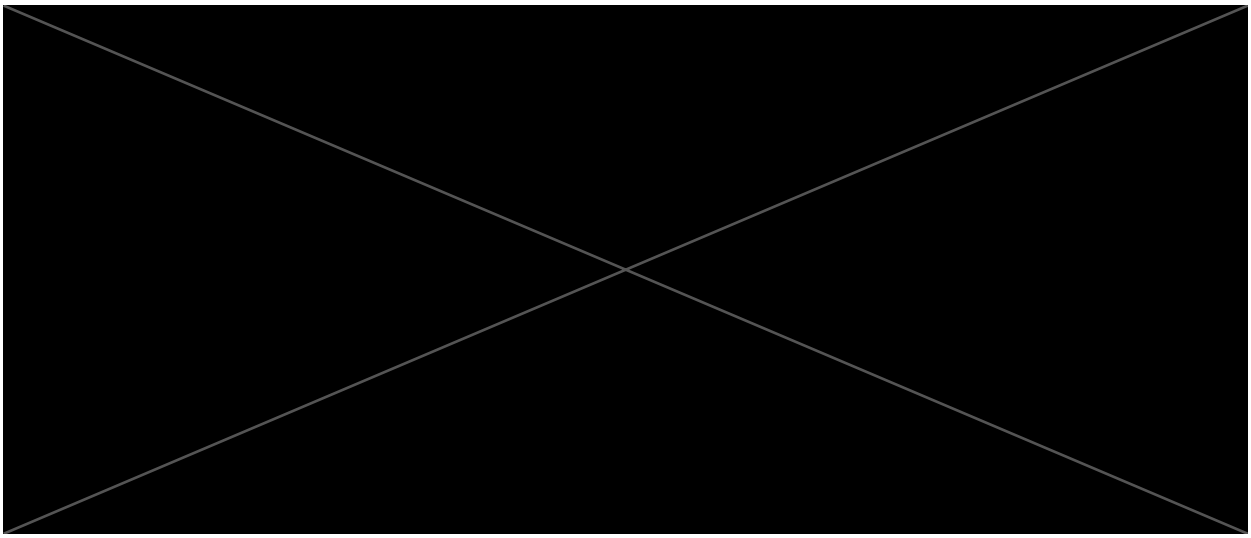

Var och en av de olika levnadsvanorna som uppmärksammas i Lev kan bidra till förbättrad psykisk eller fysisk hälsa. I manualen för praktiskt genomförande och i deltagarens arbetsbok finns enkla råd om varje levnadsvana.

Etikprövningsmyndigheten
2022-02-10
Dnr 2018-01-266722
2022-05-09

Funktionsnedsättning och levnadsvanor

Att ha en funktionsnedsättning innebär ökad risk för att hamna i en ond spiral. Både levnadsvanor och hälsa är generellt sämre jämfört med personer utan funktionsnedsättning (WHO, 2020). I Lev utgår vi ifrån ett biopsykosocialt synsätt (WHO, 2011) där individens unika förutsättningar och den omgivande miljön analyseras för att förstå vilka hinder som finns på vägen till att skapa hälsosamma levnadsvanor. Livshändelser, den omgivande miljön, psykologiska och fysiologiska förutsättningar måste alla inkluderas i analysen för att skapa bättre förutsättningar för hälsosamma levnadsvanor. Alla människor, oavsett sätt att fungera, har rätt till god hälsa. Detta är nödvändigt för att kunna leva ett liv i värdighet och är en grundläggande del av våra mänskliga rättigheter (WHO, 1946). Det innebär att en viktig uppgift för behandlaren blir att identifiera och undanröja hinder. Samtidigt är det viktigt vara lyhörd på det som fungerar och stärka individens tilltro till sig själv.

Utveckling av Lev

Levnadsvanor har pekats ut som ett område som ska prioriteras inom alla delar av hälso- och sjukvård. Riskgrupper, så som grupper med psykiska, kognitiva och fysiska funktionedsättningar ska prioriteras då dessa grupper har högre risk för ohälsosamma levnadsvanor. Habilitering & Hälsa, Regions Stockholm tog därför initiativ att jobba för att implementera dessa Socialstyrelsens riktlinjer (2018). Utifrån uppdraget, där det bland annat ingår att **”främja en aktiv och hälsosam livsstil hos patienten i syfte att förebygga psykisk och fysisk ohälsa”,** beslutade ledningen att inkludera fler levnadsvanor än vad som uppmärksammas i Socialstyrelsens riktlinjer. Ytterligare levnadsvanor som inkluderades var de som forskningsstudier visat att de har en påverkan på hälsan. En styrgrupp bildades innehållande projektledare, habiliteringsläkare, FoUU-chef samt sektionschef/del av ledningen. Styrgruppens kompetens omfattade bland annat god kunskap om nationella riktlinjer kring hälsa och levnadsvanor, utformning och implementering av likande insatser samt inblick i den egna organisationens arbetssätt och långsiktiga plan.

Efter diskussion i styrgruppen och genom att söka litteratur kunde det konstateras att det inte fanns någon insats som uppfyllde kriterier som identifierades som viktiga:

- **Vetenskapligt utvärderad**: Det saknas manualiserade insatser som har utvärderats beträffande genomförbarhet och effektivitet*.
- **Transdiagnostisk**: Det saknas insatser som är utformade att fungera för en heterogen population där ett flertal olika psykiska och fysiska funktionsnedsättningar kan förekomma.
- **Interprofessionell**: Det saknas insatser som kan genomföras av flertalet professioner som verkar inom vården.
- **Psykologiskt stöd:** Det finns information om hur man borde leva men få insatser som stöttar behandlare i genomförande och uppföljning av detta arbete.
- **Samverkan:** Det saknas tydliga instruktioner och rutiner för hur samverkan kan ske med andra aktörer som ger vård och stöd.

*Lev har när den här versionen skrivs, inte genomgått vetenskaplig utvärdering.

Det finns emellertid god tillgång på riktlinjer och informationsmaterial i Sverige. Vi har haft som utgångspunkt att använda och bygga vidare på det material som redan används inom vården idag. I utvecklingen av denna manual har vi därför använt information från Nationellt vårdprogram 2022, 1177, viss.se och från andra specialiserade aktörer inom varje levnadsvana när vi utformat de standardiserade så kallade ”enkla råd” som ges till deltagaren. Det finns även goda kunskaper om levnadsvanor inom vården idag. En viktig funktion för Lev är därför att ta

Etikprövningsmyndigheten
2022-02-10
2022-05-09
Dnr 2021-01-266722

vara på den kunskapen och ge förslag på vilka aktörer som finns att tillgå så att det blir enklare och tydligare vem som ska göra vad.

För att insatsen ska bli relevant, enkel att använda samt passa in med befintliga arbetssätt, har behandlare från olika yrkesprofessioner (arbetsterapeuter, logopeder, psykologer, fysioterapeuter, kuratorer och specialpedagoger) deltagit utvecklingen.

Avgörande för utvecklingen har även var de möten som skett med intresseorganisationer för olika funktionsnedsättningar. Baserat på dessa möten identifierades följande teman:

- **Psykologiskt stöd:** Man efterlyser att få stöd som fokuserar på att ändra själva beteendet (levnadsvanan). Även om kunskap behövs är det primära problemet inte att få information om hur man borde leva utan stöd och övningar i hur man gör det.
- **Hopp och inspiration:** Betoningen ska vara på hur man kan må bättre snarare än vad som händer om man inte gör ”rätt”. Man efterlyser även att bli inspirerad. Exempelvis genom att få veta om någon annan i likande situation som lyckats ändra sina levnadsvanor.
- **Långsiktighet:** Man efterlyser att det stöd man får ska följas upp över tid för att det ska vidmakthållas.
- **Samverkan:** Man efterlyser att någon tar ett ansvar och följer upp deltagaren även när vård och stöd från annan aktör ges. Vidare efterlyses en bättre kommunikation mellan olika aktörer så att deltagaren inte gång på gång måste informera om sin situation och funktionsnedsättning.
- **Nätverk:** Man föreslår att insatsen ska inkludera nätverket runt deltagaren så att flera personer kan ge stöd och öka chansen att förändring äger rum.

Etikprövningsmyndigheten
2022-02-10
2022-05-09
2018-01-266722

Utvecklingen har också inkluderat samverkan med externa experter (exempelvis forskare) som fått granska insatsen även under utvecklingsfasen.

Teorier och antaganden om hur insatsen ska fungera

Baserat på en iterativ utvecklingsprocess som inkluderade aktörerna ovan beslutades det om att utveckla en insats som inkluderade följande:

1) Bedömning som ger möjlighet att uppmärksamma många levnadsvanor, 2) psykoedukation om vikten av levnadsvanor utifrån standardiserade enkla råd, 3) metoder så som ”Motiverande samtal” och ”Tillämpad beteendeanalys” för att engagera och stötta i genomförandet,

4) beskrivning av hur samverkan och samarbete kan ske mellan olika aktörers, 5) uppföljning under genomförandet och efter insatsen för att öka chansen för vidmakthållande av hälsosamma levnadsvanor. Dessa delar beskrivs mer utförligt nedan.

Uppmärksamma/Bedöma

Att fråga om något är ett sätt att kommunicera att det är viktigt. Bara att ställa frågorna om levnadsvanor kan därför leda till en viss beteende-förändring (Wilding et al., 2019). Vilka frågor man ställer pekar på vad som är viktigt att tänka på inom en levnadsvana och på så sätt kan deltagaren och behandlare få vägledning kring vad de kan fokusera på. Att få en initial bedömning ger även möjlighet att följa utveckling över tid samt ökade chanser att utvärdera om det stöd man ger har någon effekt. Självklart ger det även utökad möjlighet att upptäcka större trender så som vilka grupper som behöver ett fördjupat stöd (Powell et al., 2019). Det finns en risk med att deltagarna kan känna sig nedslagen om de får låga poäng. Det är viktigt att man kommunicerar att levnadsvanor inte är egenskaper utan att de går att ändra på. Fokus bör ligga på att väcka hopp och att det finns stor potential till förbättrad hälsa snarare än att man gjort fel.

Etikprövningsmyndigheten
2022-02-10
2022-05-09

Utveckling Lev-s

Utvecklingen av frågorna påbörjades och utvärderades i samband med en masteruppsats på psykologprogrammet vid Karolinska Institutet (Wester & Stålhand, 2021). Endast ett fåtal frågor inkluderades inom varje område för att det skulle vara möjligt att få en översikt över många levnadsvanor. Bedömningsdelen (Lev-s) bör därför ses som en översiktlig bedömning (även kallat screening) som man gör initialt och som kan kompletteras med mer djupgående mätningar. Frågor valdes efter vad som identifierats som de viktigaste delarna inom varje levandsavana av WHO, Socialstyrelsen, Nationellt vårdprogram, meta-analyser eller av expert inom området. För ”Kost” och ”Alkohol” anpassades etablerade index, Audit-C (Berman et al., 2017) kostindex (Socialstyrelsen, 2014) passa att passa in i Lev-s.

För att undvika att behöva utveckla olika gränsvärden och normer för den tilltänka heterogena, transdiagnostiska populationen, användes en kvasi-absolut skala i likhet med ”Psychopathological Rating Scale” (CPRS) (Svanborg & Åsberg, 2001). Svarsalternativen är ordnade efter en ordinal likertskala från 0-3 där poäng mellan 0 till och med 1 indikerar hög risk för framtida ohälsa, över 1 till och med 2; risk för framtida ohälsa och över 2 upp till 3; bidrar till god hälsa. Varje skalsteg är definierat vilket minskar risken för mätfel. Även om poängbedömningen är noggrant diskuterade med experter inom varje område är det huvudsakliga syftet inte att ge en exakt och utförlig kartläggning inom varje levnadsvana. Behandlaren bör kommunicera att syftet med Lev-s är att upptäcka inom vilket område man bör titta mer. Sålunda är resultat från Lev-s ingen

diagnos utan en indikation på hur det ser ut just nu och vart det skulle kunna löna sig att fördjupa sig.

I första hand prioriterades att fråga om och definiera svarsalternativ utifrån vad som kan anses objektivt hälsosamt (det vill säga övertygande stöd i forskning). Exempel på detta är att forskning har visat vilken mat som är nyttig och hur mycket sömn och fysisk aktivitet vi behöver. Det eftersträvades även att frågorna skulle ta fasta på olika aspekter. För områden där det inte finns ett objektivt hälsosamt alternativ användes deltagarens subjektiva tillfredställelse/nöjdhet. Exempel på detta är att forskning visat att god sexuell hälsa och goda relationer är viktigt men det är olika från person till person vad det innebär.

Diskussioner fördes med behandlare och experter för att inkludera ytterligare aspekter som kunde vara viktiga att ta hänsyn till kopplade olika funktionsnedsättningar.

Psykoedukation

Psykoedukation är strukturerad information som syftar till att ge deltagaren kunskap och strategier för att bättre kunna möta olika situationer. Psykoedukation kan utgöra en fristående intervention och vara effektiv i sig och den kan också användas som del i en behandling bestående av flera delar. Informationen kan leda till förändring och den kan också hjälpa till att öka motivationen för att göra en kommande ansträngning för att ändra ett beteende (Hedman-Lagerlöf et al., 2019). Första fasen i Lev bygger till största delen på psykoedukation och kan ges fristående. I Socialstyrelsens riktlinjer (2018) lägger man vikt vid att hälso- och sjukvården ska kunna ge enkla råd om levnadsvanor. I linje med dessa riktlinjer innehåller Lev standardiserade enkla råd bestående av rubrikerna ”Rekommendation”, ”Varför det är viktigt”, ”Exempel på vad du kan göra själv”. Råden bygger i stor utsträckning på den information som finns på 1177 men har även justerats av experter inom varje område. Dessa råd kan ges muntligen av behandlaren, i textform och som korta filmer* så att informationen kan repeteras hemma samt delas i deltagarens nätverk om så önskas. Även om man väljer att endast ge första fasen i Lev ska man ändå sätta mål med deltagaren, ge hemuppgifterna samt följa upp.

Det är viktigt att notera att det framgår av Socialstyrelsens riktlinjer (2018) att psykoedukationen bör vara grunden men att mer omfattande åtgärder på rådgivande och kvalificerat rådgivande nivå även ska äga rum när man träffar individer från riskgrupper.

*Alla filmer är inte klara när den här versionen skrivs.

Etikprövningsmyndigheten
2022-02-10
2022-05-09

Etisk reflektion

Patienten behöver information för att kunna vara delaktig. Informationen ska anpassas så att den blir begriplig och det bör finnas utrymme för reflektion kring vårdval. De flesta människor tycker att det är positivt att föra ett samtal om levnadsvanor men enskilda personer kan uppleva vissa frågor som integritetskränkande. När man frågar eller informerar om levnadsvanor är det viktigt att beakta deltagarens integritet men det får inte ske på bekostnad att man fortsättningsvis undviker att informera och föra en dialog om levnadsvanor (Socialstyrelsen, 2018). Behandlaren bör informera deltagaren att anledningen till att frågorna ställs är för att vi ska öka chanserna att förbättra livskvalitet och hälsa. Det är viktigt att informera deltagaren att han eller hon av avstå att svara på frågor.

Motiverande strategier och samtal

För att öka chansen att samtalet mellan behandlare och deltagare har effekt bör behandlaren ha kunskap om hur man ökar motivation till förändring (Nationellt vårdprogram, 2022). Målet är att upprätta ett **partnerskap** mellan behandlaren och deltagaren genom att **lyssna** och **ge värde åt deltagarens berättelse**. Även under den psykoedukativa fasen är det viktigt med dialogform genom att bland annat fråga hur ett påstående stämmer in på deltagarens erfarenheter. Behandlaren ska se deltagaren som en beslutskapabel och jämbördig partner som är en viktig nyckel i hur kommande råd kan anpassas.

Etikprövningsmyndigheten
2022-02-10
2018-01-266722
2022-05-09

I Lev används motiverande strategier i alla faser. Det betyder att det är viktigt att fokusera på deltagarens egna skäl och motivation till att vilja förändra levnadsvanor. Behandlaren ska uppmuntra till att deltagaren berättar med egna ord om erfarenheter, önskningar och vilka fördelar de ser med att förändra sina levnadsvanor. Ett viktigt verktyg är att använda sig av motiverande samtal när deltagaren inte ännu är motiverad eller är ambivalent. Målet är att få till så kallat **förändringsprat**. För att nå dit kan det vara bra att ha ett **empatiskt förhållningssätt, ge hopp, stärka tilltron till den egna förmågan att genomföra en förändring**. Vidare är det bra med **öppna frågor** som inte kan besvaras med ett ja eller nej utan som öppnar upp möjlighet till en dialog.

- Vad är det som gör att du villa prata om levnadsvanor?
- Berätta om hur du skulle vilja att din hälsa var i framtiden.
- Hur ser du på att göra en liten förändring av din fysiska aktivitet?

Eftersom det är viktigt att visa att du lyssnar kan behandlaren använda sig av **sammanfattningar** av det deltagaren sagt. **Upprepa eller använd enkla synonymer** till det som sagts och försöka att lyfta fram underliggande budskap och göra reflektioner.

Om en bestående förändring av en levnadsvana ska bli till är motivationen hos deltagaren avgörande. När motivation hos deltagaren utforskas är följande tre frågor viktiga (Nationellt vårdprogram, 2022):

- Hur viktig är förändringen är för personen.
- Hur stor tilltro har man till att klara av att göra förändringen.
- Hur beredd är man att göra förändringen.

Om behandlaren är osäker gällande motivationen finns det en bilaga i manualen för det praktiska genomförandet som stöttar behandlaren i att utforska motivationen ytterligare.

Motstånd

Det kan ibland uppstå motstånd. Manualen för det praktiska genomförandet innehåller exempel på hur behandlaren kan hantera motstånd. Läs dessa exempel innan du träffar en deltagare och var beredd på att du kan behöva komma på nya exempel som passar den person du pratar med. Motståndet kan även bero på att behandlaren gått för fort fram så som att ge råd och lösningar för tidigt i processen. Det kan också vara att delmålen är för stora. Jobba snarare med små genomförbara steg så att deltagaren får ökad tilltro till sig själv. Eftersträva att deltagaren själv får komma med lösningar när han eller hon är mogen för det. Kritisera inte deltagaren eller dennes personlighet. Ohälsosamma levnadsvanor är inte personens egenskaper utan ”problem” ni ska lösa tillsammans. Under utbildningen får behandlaren handledning parallellt med att han eller hon genomför Lev. Under dessa tillfällen kan svåra situationer som uppstått diskuteras.

Etikprövningsmyndigheten
2022-02-10
2022-05-09
2018-01-266722

Etisk reflektion

Det kan vara bra att reflektera över hur långt man bör driva frågan om beteendeförändringar gällande en levnadsvana när man möter motstånd (Socialstyrelsen, 2018). I denna insats föreslås att förhållningssättet ska vara att motivera och ge adekvat information om risker och möjligheter med förändrade levnadsvanor. Det är i slutändan upp till deltagaren att välja område och utsikterna är bättre om motivationen är hög (Nationellt vårdprogram, 2022). Om deltagaren inte vill just nu ska det respekteras och behandlaren ska berömma deltagaren för att hen tagit sig tid och lyssnat (samt eventuella steg som tagits) och berätta att de är välkomna tillbaka och diskutera levnadsvanor på sina villkor. Samtalet kan ha satt i gång en tankeprocess i deltagaren och möjligheten är sannolikt större att fler hälsosamma beslut fattas i framtiden.

Tillämpad beteendeanalys

Att veta att man bör ändra en levnadsvana är tyvärr ofta inte tillräckligt. För att få till en beteendeförändring krävs ofta kraftfullare verktyg. I framför allt fas 2 i Lev inkluderas analys av beteenden för att öka

möjligheten för deltagaren att nå sina mål. Nedan följer en kort bakgrund till de olika delar vi inkluderat i Lev. Lev riktar sig till olika professioner inom hälso-och sjukvård och vi har därför strävat efter att hitta en nivå som ska fungera även utan förkunskaper. För den behandlare som behärskar fler nyanser av den tillämpade beteendeanalysen än vad som föreslås i manualen för Lev går det givetvis bra att applicera dessa.

Belöning

I samtalet med deltagaren lyfts ”**belöning**” och vilken roll den spelar. Det mesta av vårt beteende har vi lärt oss för att det, förenklat sätt, gett oss någon from av belöning. Så är det med våra levnadsvanor också. Vi äter godsaker för att de får oss att må bra i stunden. Att känna till det kan hjälpa oss att förstå varför vi skapat både ohälsosamma och hälsosamma levnadsvanor. Det kan även hjälpa oss förstå att dessa levnadsvanor inte är del av vår personlighet utan något vi lärt oss (Karlsson, 2010). Att veta det kan i sin tur hjälpa oss tro på förändring när vi står inför att minska en ohälsosam levnadsvana eller lägga till en ny hälsosam levnadsvana. När vi pratar om belöning i Lev tar vi även upp hur ”att undvika obehag” också är en form av belöning. Att vi dricker alkohol, shoppar, spelar eller äter för att slippa ha tråkigt eller undvika obehag är exempel på denna typ av belöning. Ofta är vi inte medvetna om att vi så ofta styrs av belöning i våra val (Karlsson, 2010). Det är viktigt att deltagaren involveras genom att behandlaren frågar hur det stämmer in samt ber om deltagarens egna exempel.

Etikprövningsmyndigheten
2022-02-10
2022-05-09

Att lära sig prioritera det långsiktiga framför det kortsiktiga

En viktig del i att välja hälsosamma levnadsvanor är att lära sig prioritera det som är bra för oss på lång sikt. Lev lyfter fram exempel på levnadsvanor som kan kännas bra i stunden men som är dåliga för oss på lång sikt. Enligt samma princip nämns exempel som kan kännas jobbiga i stunden (exempelvis fysisk aktivitet) men som får oss att må bra på längre sikt. En viktig uppgift för behandlaren är att ge deltagaren en mer levande relation till sitt framtida jag.

Funktionell kartläggning

I Lev används en förenklad version av en funktionell kartläggning. Med detta moment är det viktigt att behandlaren ”flyttar” det oönskade beteendet från individen och ser det mer som en konsekvens av samspelet med närmiljön. Att minska skuld och skam kan leda till mer energi och motivation att genomföra förändringar. I en funktionell kartläggning är en diagnos aldrig förklaringen. Det vi är ute efter är konkreta observerbara beteenden och funktionen de fyller (Karlsson, 2010).

Behandlaren och deltagaren försöker förstå vad som händer innan och efter det **oönskade beteendet**. Två viktiga syften med denna analys är att identifiera **”situationer”** där man frestas till att fatta ohälsosamma

beslut och att möjliggöra önskade beteenden genom att ge deltagaren **underlättande strategier**.

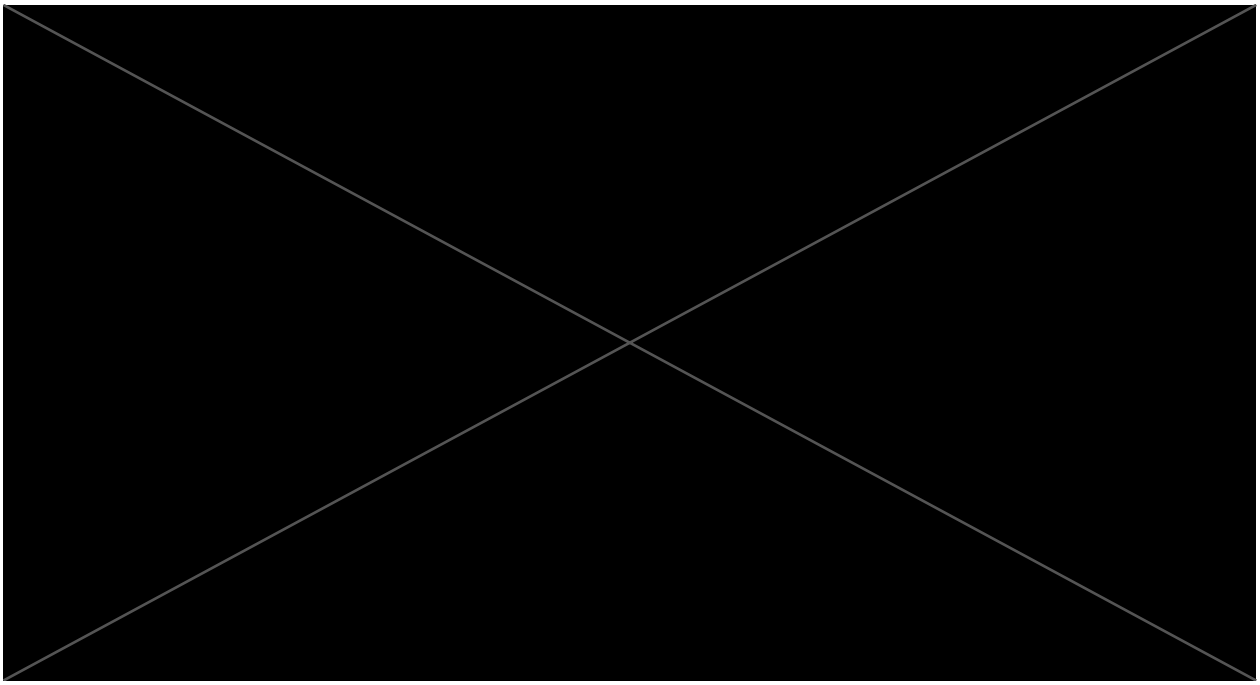

Etikprövningsmyndigheten
2022-02-10
2022-05-09

Det brukar vara enklast att börja med att skriva ned det problematiska beteendet (till exempel tar en cigarett). Nästa fråga handlar om att behandlaren frågar vad som hände innan – vilken **situation** deltagaren befann sig i. Det finns i regel många situationer men behandlaren väljer en i taget (till exempel är med en vän som röker). Nu frågar behandlaren deltagaren vilken **kortsiktig konsekvens** (belöning) det får deltagaren att känna. Behandlaren ger förslag om deltagaren inte kommer på något (till exempel samhörighet, mår bra, slapp vara kvar inne med en främling). Nästa steg är att lyfta fram den **långsiktiga konsekvensen** (ökad risk för sjukdomar). De flesta av oss vet riskerna men vi gör det ändå. Försök lägga fokus på att lyfta fram möjligheterna (till exempel bättre kondition, känna sig starkare, inte bli sjuk lika ofta). I manualen för det praktiska genomförandet finns exempel för hur man fyller i ”situation”, ”beteende”, ”kortsiktig konsekvens” ”långsiktig konsekvens” för varje levnadsvana. Där finns också exempel på strategier som du kan ge deltagaren. Exemplen är i första hand till för utbildningssyfte. Eftersträva att skapa personcentreade exempel tillsammans med deltagaren. Repetera analysen för några olika situationer och skriv ned allt i arbetsboken så att deltagaren kan titta på det i lugn och ro i hemmiljö. Genomför sedan analysen av önskade beteenden.

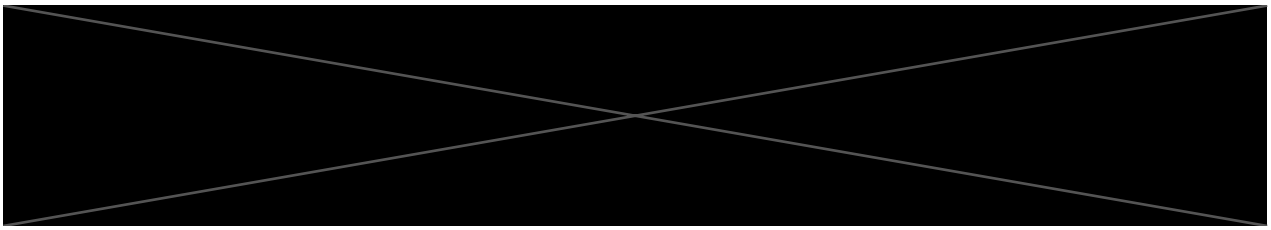

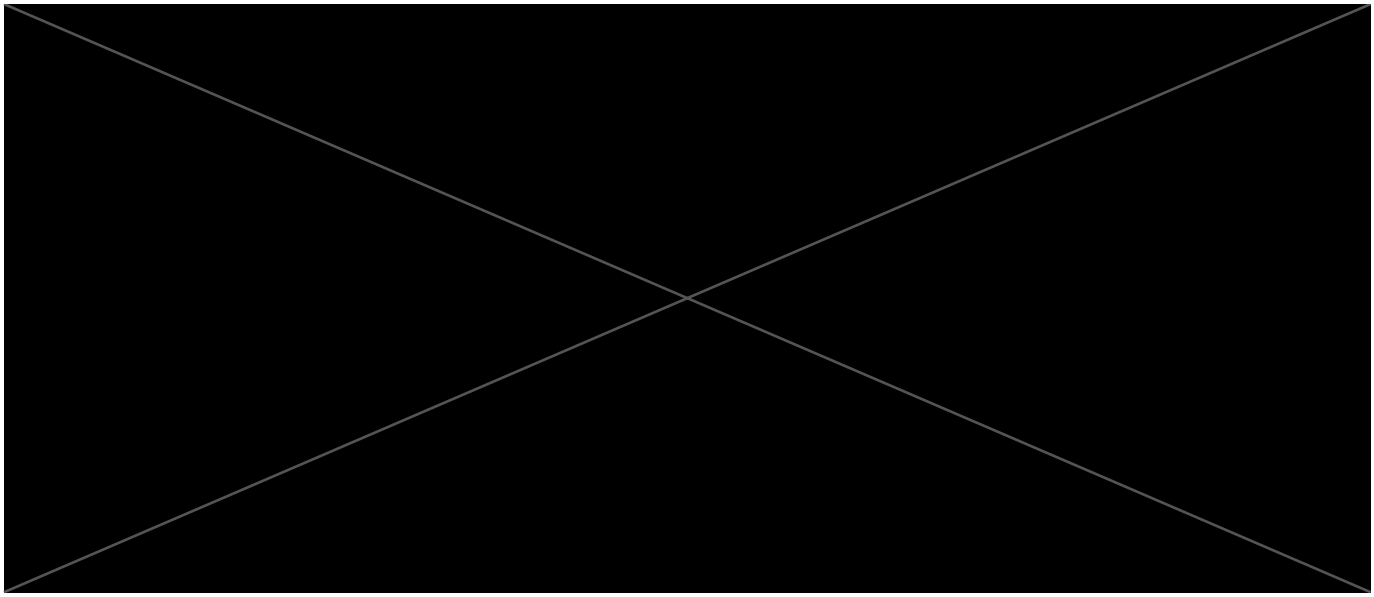

Beteendestödsplan

Syftet med den funktionella analysen är att kunna ge deltagaren strategier för att skapa alternativa beteenden (Karlsson, 2010). I Lev ges tips om hur deltagaren kan **undvika att hamna i utmanande situationer** där ohälsosamma beteenden brukar uppstå. Vidare ges strategier som ska **underlätta önskade beteenden**. Det är viktigt att involvera deltagaren så att strategierna är rimliga och genomförbara. Börja med små steg. Stäm av med deltagaren om han eller hon känner i igen sig i analysen. Behandlaren behöver reflektera över vilken färdighetsträning som kan behövas. Exempelvis kan behandlaren öva med deltagaren på hur man tackar nej eller hanterar frustration (Karlsson, 2010). Om möjlighet finns kan det underlätta om behandlaren följer med till den miljö där beteendet ska implementeras och visar och övar med deltagaren. Vidare kan nätverket runt deltagaren hjälpa till att underlätta beteendestödsplanen. Ett sätt att lära sig är att titta på andra som har hälsosamma vanor. Hur gör de? Kan du fråga dem? Deltagaren kan behöva stöd att generalisera nya beteenden till nya kontexter. Kanske har man precis lärt sig att äta hälsosamt till lunch men slarvar oftare till middagen. Vissa personer har svårt att generalisera beteenden till nya sammanhang och behandlaren kan därför behöva stötta deltagaren i denna process.

Etikprövningsmyndigheten
2022-02-10
2022-05-09

Uppföljning under fas 2

Det är viktigt att deltagaren blir aktiv mellan mötena med behandlaren (Karlsson 2010). I Lev får deltagaren i hemuppgift att skriv ned när han eller hon använder de strategier som de skrivit ned. De får även i uppgift att skriva ned exempel på när de lyckas stå emot kortsiktiga belöningar (de ni listat arbetsboken) för att i stället få de långsiktiga. Behandlaren följer upp och ger positiv förstärkning och stöd på distans. Fas 2 kan genomföras flera gånger för deltagare som behöver det.

Samverkan

All hälso- och sjukvårdspersonal ska ha kompetens att uppmärksamma ohälsosamma levnadsvanor men också ha kunskap om lokala rutiner för hänvisning till insatser på kvalificerad nivå inom eller utanför den egna organisationen. En viktig del i utvecklingsarbetet av Lev har varit att diskutera med andra aktörer som ger insatser på kvalificerad nivå hur hänvisning ska ske. I manualen för det praktiska genomförandet finns information om vem som ger insats på kvalificerad rådgivande nivå och tips om hur hänvisning ska ske. Denna del måste givetvis anpassas beroende på vilken vårdorganisation som implementerat Lev. Det ska framgå tydligt hur samverkan och samarbete ska ske. Även när du hänvisat till extern aktör ska du följa upp och fråga deltagaren om den nya vårdkontakten fungerar.

I samtalen med olika intresseorganisationer för patientgrupper framkom att det fanns ett behov av bättre samverkan mellan olika vårdaktörer och andra former av stöd. Det lyftes fram att personer med funktionsnedsättning ofta behöver redogöra för hela sin historia vid varje ny vårdkontakt och att det därför blir mindre tid kvar till att få adekvat vård. Kunskapsbrister och otillräckligt bemötande hos vårdpersonal bidrar till personer med funktionsnedsättning undviker att söka vård (SKL, 2018).

Etikprövningsmyndigheten
2022-02-10
2018-01-266722
2022-05-09

Uppföljning

Uppföljning beskrivs som en viktig förutsättning för att insatser som syftar till att förbättra levnadsvanor ska vara effektiva (Socialstyrelsen, 2018). Uppföljning görs vid flera tillfällen under det att Lev genomförs. Dels genom att genomföra de tre faserna, dels genom att anpassa uppföljning mellan dessa tillfällen efter deltagarens behov av stöd (Nationellt vårdprogram, 2022). Uppföljningen som sker mellan faserna är korta och kan vara i from av ett telefonsamtal eller via digital behandlingsplattfrom. En viktig funktion för uppföljningen är att motivera deltagaren att vara aktiv och jobba mellan det att han eller hon träffar behandlaren. Uppföljningen sker genom att behandlaren ger återkoppling på de hemuppgifter deltagaren fått. Om hemuppgifterna inte genomförs bör behandlaren boka in fas 2 för att kunna få i gång deltagaren med hjälp av den tillämpade beteendeanalysen. Uppföljning sker även när hänvisning skett till extern aktör som ger insats på kvalificerad nivå.

Vidmakthållande

Som en del i planen för vidmakthållande bör uppföljningar göras vid 3, 6 och 12 månader efter avslutad insats (Nationellt vårdprogram, 2022). I uppföljningen kan bland annat följande frågor användas:

- Hur känns det nu, efter en tid?

- Vad behöver du för stöd för att fortsätta med ditt nya beteende?
- Varför är det viktigt för dig att fortsätta välja det nya beteendet?
- Om du började med att ta tag i ytterligare en levnadsvana, vilken skulle det vara?

Behandlaren kan även besluta att genomföra insatsen igen men med fokus på en annan levnadsvana än den som deltagaren jobbat med hittills. Att repetera insatsen kan öka chansen för att deltagaren på sikt lär sig jobba självständigt med sina levnadsvanor. Ett annat alternativ är att låta deltagaren på egenhand ta del av de enkla råd som ges under fas 1 för en ny levnadsvana. Behandlaren kan i stället följa upp på distans och låta deltagaren göra de tre faserna själv och endast ge feedback på hemuppgifterna.

Referenser

- American Educational Research Association, American Psychological Association, & National Council on Measurement in Education. (2014). *STANDARDS for Educational and Psychological Testing*.
- Berman, A. H., Wennberg, P., & Kallmén, H. (2017). *AUDIT och DUDIT. Identifiera problem med alkohol och droger* (2.1). Gothia Förlag.
- Constitution of the World Health Organization. Hämtad den 17 december 2021 från https://www.who.int/about/governance/constitution.
- Hedman-Lagerlöf, E., & Axelsson, E. (2019). Assessment of health anxiety. In E. Hedman-Lagerlöf (Ed.), *The clinician's guide to treating health anxiety: Diagnosis, mechanisms, and effective treatment*. Elsevier Academic Press. https://doi.org/10.1016/B978-0-12-811806-1.00005-6
- Karlsson, P. (2010). Beteendestöd I vardagen: handbok I tillämpad beteendeanalys. Natur & Kultur.
- Nationella riktlinjer för prevention och behandling vid ohälsosamma levnadsvanor: stöd för styrning och ledning. Stockholm: Socialstyrelsen; 2019. Hämtad 2022-01-11 från https://www.socialstyrelsen.se/regler-och-riktlinjer/nationella-riktlinjer/slutligariktlinjer/levnadsvanor
- Powell, H. S., Greenberg, D. L. (2019). Screening for unhealthy diet and exercise habits: The electronic health record and a healthier population. Prev Med Rep. 8, 14:100816. doi: 10.1016/j.pmedr.2019.01.020.
- Socialstyrelsen. (2014). *Enkla råd för bra matvanor*. https://www.socialstyrelsen.se/globalassets/sharepoint-dokument/artikelkatalog/kunskapsstod/2014-6-24.pdf
- SKL (2018). Hur skapar vi bättre hälsa för personer med funktionsnedsättning? Hämtad 210202 https://skr.se/integrationsocialomsorg/socialomsorg/funktionshinder/battrehalsa.24420.html
- Stein, A. T., Carl, E., Cuijpers, P., Karyotaki, E., Smits, J. A. J. (2020) Looking beyond depression: a meta-analysis of the effect of behavioral activation on depression, anxiety, and activation. Psychol Med. 51. 1491-1504. doi: 10.1017/S0033291720000239.
- Svanborg, P., & Asberg, M. (2001). A comparison between the Beck Depression Inventory (BDI) and the self-rating version of the Montgomery Asberg Depression Rating Scale (MADRS). *Journal of Affective Disorders*, *64*, 203–216. https://doi.org/10.1016/s0165-0327(00)00242-1
- Wester, G., & Stålhand, F. (2021). *Development of the Lifestyle habits Evaluation. A new instrument which primarily aims to assess the lifestyle habits of patients with functional disabilities.* Master thesis.
- Wilding, S., Conner, M., Prestwich, A., Lawton, R., Sheeran, P. (2019) Using the question-behavior effect to change multiple health behaviors: An exploratory randomized controlled trial, Journal of Experimental Social Psychology, 81, 53-60.
- World report on disability, 2011. World Health Organization. Available at: www.who.int.

Etikprövningsmyndigheten
2022-02-18
2022-01-266722
2022-05-09

- World Health Organization. (2018). The World Health Organization: Working for better health, for everyone. [Brochure]. https://www.who.int/about/what-we-do/who-brochure
- World Health Organization. (2020). Disability and health. Disability and Health. https://www.who.int/news-room/fact-sheets/detail/disability-and-healt

- Uppdatera referenslistan när allt är klart.

Syfte

- Att möjliggöra att fler behandlare inom olika vårdyrken tar initiativ till att uppmärksamma, åtgärda och följa upp levnadsvanor.
- Att underlätta samverkan och samarbete mellan olika hälso- och sjukvårdsaktörer.

Övergripande målsättning för deltagare

- Ökad allmän kunskap om koppling mellan levnadsvanor och psykisk och fysisk hälsa samt socialt välbefinnande.
- Ökad motivation till att skapa hälsosamma levnadsvanor.
- Bättre insikt i vilka situationer som leder till ohälsosamma levnadsvanor.
- Att lära sig fler strategier för att kunna prioritera långsiktig hälsa.
- Ökad självständighet och initiativ kring egna levnadsvanor.
- Hälsosammare levnadsvanor.

Etikprövningsmyndigheten
2022-02-10
2018-01-266722
2022-05-09

Övergripande målsättning för behandlare

- Fler initiativ till samtal om levnadsvanor med deltagare.
- Utökat samverkan/samarbete med andra aktörer som ger insatser kring hälsorelaterade levnadsvanor.
- En helhetsbild av deltagarens levnadsvanor.
- Kunskap om hur man ger råd och uppföljning för flera levnadsvanor.

Övergripande principer

- Hopp - Att väcka hopp om hur levnadsvanor kan bidra till bättre hälsa snarare än hot om möjliga konsekvenser.
- Att inkludera en metod som möjliggör att arbeta med flera olika levnadsvanor (uppmärksamma – åtgärda - följa upp).
- Aktivt engagemang och delaktighet från deltagaren.
- Ansvar – att ge ett empatiskt och psykologiskt stöd över tid.
- Alla människor oavsett funktion, har rätt till hälsosamma levnadsvanor.
- Minska skuld och skam.

Del 2

Det praktiska genomförandet av Lev

Behandlarens roll

Den som genomför Lev ansvarar för att följa manualen för det praktiska genomförandet. Det är viktig att vara väl förberedd så att uppmärksamheten kan riktas mot deltagaren snarare än följa texten ordagrant. Behandlaren har även i uppgift att kalla till möten och följa upp hemuppgifter mellan varje besök. Behandlaren har ansvaret för att skriva ut material till deltagaren (eller lägga detta i en digital plattform). En annan viktig uppgift är att ta ansvar i hänvisningen till annan vårdaktör samt följa upp både under och efter att insats genomförts.

Vem han ge insatsen?

Lev kan ges av vårdpersonal oavsett profession. Behandlaren ska ha genomgått utbildning i Lev.

För vilka är detta en lämplig insats?

Lev är en transdiagnostisk insats vilket betyder att den ska fungera för flertalet diagnoser. Dock ska deltagaren vara över 18 år, inte vara beroende av en medföljare. Psykiatriska diagnoser eller funktionsnedsättningar (till exempel depression, ångest, autism eller ADHD) hos deltagaren är inget hinder för insatsen, så länge de inte är av den grad att de hindrar aktivt deltagande. Annan pågående läkemedelsbehandling går bra. Annan pågående insats är inget hinder. Det kan exempelvis vara lämpligt att behandlare och deltagare bestämmer en lämplig extern insats för att förstärka det arbete som ni gör. Ett exempel på detta är att ringa Sluta-Röka-Linjen om ni har som mål att minska rökning. Lev är dock en insats som fungerar mest effektivt om deltagaren jobbar aktivt mellan möten med behandlare. Behandlaren bör därför ta hänsyn till vad och jobba för att motivationen är tillräcklig.

Deltagaren ska inte få behandlingen om han eller hon har en för allvarlig psykiatrisk samsjuklighet så som pågående missbruk, maniska episoder, psykos eller suicidalitet. Om du som behandlare blir osäker på en deltagarens mående är det viktigt att ta upp det och funderar över om Lev är en lämplig insats just nu. Ibland är det bättre att skjuta upp insatsen till ett senare tillfälle då behandlaren har lättare att hjälpa deltagaren till att bli motiverad nog för att kunna nå sina mål. Om deltagaren vill avsluta pågående behandling bör du som behandlare komma ihåg att ni trots allt kan ha lagt grunden för att jobba med levnadsvanor i framtiden. Du bör alltid berömma de framsteg eller försök som skett. Följ även upp deltagaren så att han eller hon kan få annan relevant insats.

Etikprövningsmyndigheten
2022-02-18
2018-01-266722
2022-05-09

Upplägget för Lev

Lev ges individuellt. Längden på insatsen är flexibel men bör ges över 2-6 månader. Det bör finnas tillräckligt med tid för att deltagaren ska hinna jobba med övningar mellan de olika faserna. Insatsen bör inte ta så lång tid att målen som sätts i början av insatsen börjar kännas avlägsna. Lev innehåller följande delar:

- **Bedömning - Lev-s** tar ca 25-35 minuter för en person i det normala begåvningsspannet.

- **Fas 1 – Psykoedukation** tar ca 1h men kan delas in i två träffar om det passar deltagaren bättre.
    - Behandlaren följer upp hemuppgift och ger återkoppling ca 10 min.

- **Fas 2 – Genomförande** tar ca 1h och kan genomföras flera gånger vid behov.
    - Behandlaren följer upp hemuppgifter och ger återkoppling ca 10 min.

- **Fas 3 – Uppföljning** tar ca 1h och inkluderar eftermätning.

- **Vidmakthållande** uppföljning sker vid 3, 6 och 12 månader.

Etikprövningsmyndigheten
2022-02-10
2022-05-09
2018-01-266722

Utrustning

Insatsen kan genomföras manuellt eller via digital plattform. Digital plattform kan användas oavsett om sker på distans eller på samma plats som behandlaren. Fördelen med det är att deltagaren då direkt lär sig vart arbetsboken, övningar samt enkla råd finns. Det kan vara bra i en senare fas då deltagaren kan jobba själv med materialet. Deltagaren kan med fördel använda sin egen dator så att han eller hon får hjälp att logga in på digital plattform. Det är inget krav men det kan vara till hjälp om det finns en White board där behandlaren kan rita och förklara.

Deltagarmaterial

Följande dokument ska skrivas ut eller läggas på digital plattform (se manualen för det praktiska genomförandet):

- Arbetsbok: Följer innehållet i behandlarens manual för genomförande men är nedkortat och innehåller bildstöd*.

*Bildstöd är i skrivande stund inte tillgängligt.

Åtgärdsplan

Åtgärdsplanen görs i anslutning till eller efter att man genomför Lev-s. Mall för åtgärdsplan finns i habiliteringsprogrammen. För att kunna göra en individcentrerad behandling noteras även specifika mål efter att man genomfört fas 1 och med individuella strategier efter fas 2.

Hur många tillfällen planeras för insatsen?

Lev innehåller 3 faser. Dock kan det vara aktuellt att genomföra fas 2 flera gånger. Hemuppgifterna ska även följas upp på distans efter fas 1 och fas 2.

Planering

Planeringen bör innehålla de 3 faserna samt hemuppgifter. Om deltagaren inte genomfört några hemuppgifter efter två veckor bör fas 2 bokas in så snart som möjligt. Om deltagaren jobbar på bra på egen hand kan det få dröja lite längre (1-2 månader efter fas 1) men fas 2 ska genomföras då det är viktigt för att uppmärksamma fungerande strategier och ge positiv förstärkning. Det kan underlätta deltagarens eget arbete för andra levnadsvanor efter insatsen.

Etikprövningsmyndigheten
2022-02-10
2022-05-09

Vidmakthållande

Som en del i planen för vidmakthållande bör uppföljningar göras vid 3, 6 och 12 månader efter fas 3 (Nationellt vårdprogram 2022). Se till att journalföra på ett sätt som möjliggör att vidmakthållande genomförs. Tillämpa etablerade rutiner för vidmakthållande. Exempel på frågor du kan ställa till deltagaren:

- Hur känns det nu, efter en tid?
- Vad behöver du för stöd för att fortsätta med ditt nya beteende?
- Varför är det viktigt för dig att fortsätta välja det nya beteendet?
- Om du började med att ta tag i ytterligare en levnadsvana, vilken skulle det vara?
- Ge deltagaren enkla råd från bilaga 1 i manualen för det praktiska genomförandet.

Utvärdering

Förutom att utvärdera måluppfyllelse ska deltagaren ges möjlighet att säga vad de tycker om Lev. Det möjliggör fortsatt förbättring av insatsen och det upplevs som validerande. Använd befintlig rutin för utvärdering men inkludera även frågorna nedan.

Samtliga frågor skattas i en fyrgradig skala: Ingen, Viss, Ganska stor, Stor.

- Har du fått tillräckligt med information om hur man kan leva hälsosamt?
- Du blev lyssnad på, fick du ge exempel om dina levnadsvanor?
- Hur nöjd är du men insatsen som helhet?
- Ge dina förslag på hur vi kan förbättra vårt stöd till dig (öppen fråga).

Journaldokumentation

Notera resultat för varje område för Lev-s samt om Lev ska genomföras (använd mallen för åtgärdsplan från habiliteringsprogrammen). Efter fas 1 noteras målsättning, planerad samverkan men annan vårdaktör/stöd. Efter fas 2 noteras beteendestrategier efter att den tillämpande beteendeanalysen genomförts.

Etikprövningsmyndigheten
2022-02-08
2018-01-266722
2022-05-09

KVÅ-koder

Hur och om KVÅ-koder används är upp till den organisation som implementerar lev. Nedan beskrivs hur KVÅ kan användas i relation till Lev. Som det framgår av tabellen nedan finns det koder för de fyra levnadsvanor som Socialstyrelsen prioriterar. Efter att man genomfört Lev fas 1 noters enkla råd. Efter fas 2 noteras ”rådgivande samtal”. Om du har fördjupad kompetens inom levnadsvanan och arbetar med deltagaren under flera tillfällen kan KVÅ-koder från kolumnen kvalificerat rådgivande samtal noteras.

Ett alternativ är att använda KVÅ ”DV030 Hälsosamtal” oavsett vilken levnadsvana som deltagaren jobbar med.

Tabell 1. Åtgärdskoder för dokumentation vid arbete med levnadsvanor

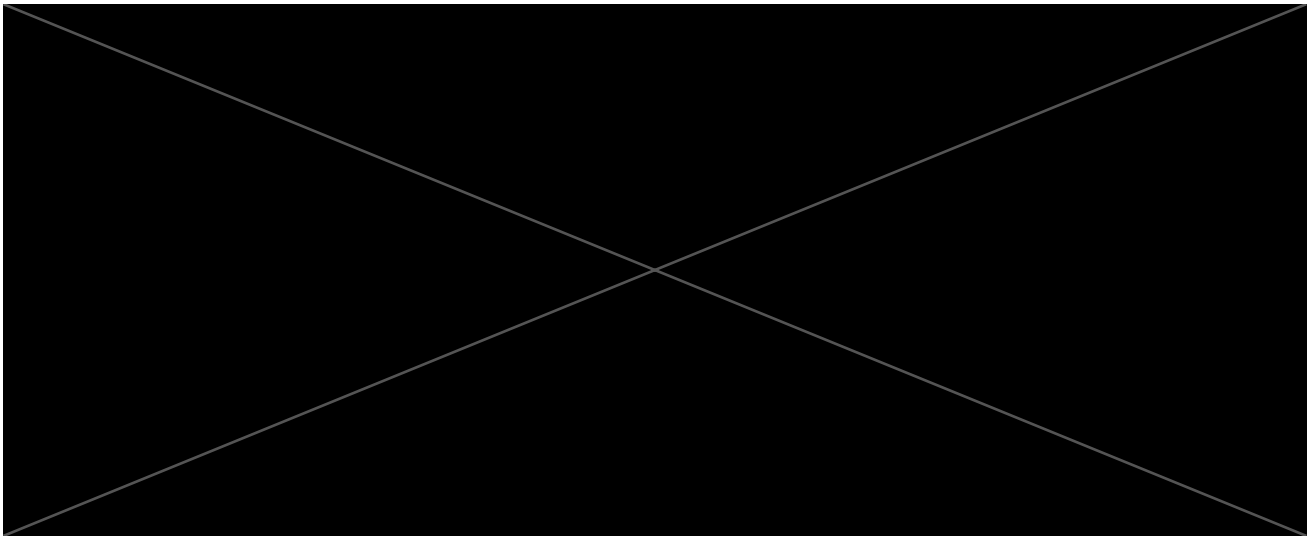

Del 3

Utbildning

Utbildningen inkluderar att behandlaren administrerar Lev-s och Lev på deltagare. Kursledaren ska därför boka in datum för kursen i god tid (minst 4 månader) innan start. Vi detta tillfälle ges information om vad utbildningen innehåller (klipp ut informationen om de olika modulerna nedan). Ge även behandlarna den teoretiska manualen vid detta tillfälle. Eftersträva att alla som deltar på kursen ligger i fas. För den som utbildar i Lev finns PowerPoint material till hjälp.

Etikprövningsmyndigheten
2022-02-10 Dnr 2018-01-266722
2022-05-09

Läs noggrant vad som förväntas av dig som behandlare så att du är förberedd till varje utbildningstillfälle. Följande steg utgör utbildning för Lev:

- Behandlarna får datum och instruktioner minst 4 månader innan start. Behandlarna bokar in flera deltagare för Lev-s i god tid. Till Modul 1 ska kursdeltagaren ha läst den teoretiska manualen.

- **Modul 1:** Introduktion och bakgrund. (Tidsåtgång ca 2h).

    - Som hemuppgift ska behandlaren läsa i manualen för det praktiska genomförandet om hur man administrerar Lev-s, titta igenom frågorna och hur man kommunicerar resultatet till deltagaren.

- **Modul 2:** Genomgång av bedömning (Lev-s). Ca 1-2 veckor efter Modul 1 ges modul 2. Kursledaren visar hur poängen från varje område räknas samman och noteras. Deltagarna delas in i par och genomför Lev-s på varandra. (Tidsåtgång 2h).

- Behandlaren genomför Lev-s på minst en riktig deltagare inom ramen för sin kliniska verksamhet. Behandlaren har möjlighet att mejla kursledaren för handledning.

- **Modul 3:** Genomgång av fas 1 (psykoedukation) i Lev. Modul 3 ges ca 2-3 veckor efter modul 2. Första timmen stämmer gruppledaren av behandlarnas erfarenheter av ett ha genomfört Lev-s. Kursledaren visar sedan manualen för det praktiska genomförandet samt deltagarens arbetsbok. Kursledaren ger handledning på de frågor som, uppstår. (Tidsåtgång 2 h)

    - Hemuppgift blir att läsa igenom fas 1 i manualen för det praktiska genomförandet och bekanta sig med deltagarens arbetsbok.
    - Behandlaren genomför fas 1 på riktig deltagare. Behandlaren har möjlighet att mejla kursledaren för handledning.

- **Modul 4:** Reflektioner från behandlarna efter att ha genomfört fas 1. Genomgång av fas 2 (genomförande). 2-3 veckor efter modul 3 ges modul 4. Kursledaren använder PowerPoint material för att gå igenom grunderna i tillämpad beteendeanalys samt motiverande samtal. (Tidsåtgång 3 h.)
    - Hemuppgift blir att planera inför mötet med deltagaren genom att ta del av och skapa egna exempel i den funktionella analysen och även försöka förutse vilken beteendestödsplan som kan bli aktuell.
    - Behandlaren genomför fas 2 på riktig deltagare. Behandlaren har möjlighet att mejla kursledaren för handledning.

- **Modul 5:** Reflektioner från behandlarna efter att ha genomfört fas 1. Genomgång av fas 3 (Utvärdering och vidmakthållande). Kursledaren går igenom de olika stegen i fas 3 utifrån manualen för det praktiska genomförandet. Tidsåtgång 1,5 h.

Etikprövningsmyndigheten
2022-02-10
2022-01-266722
2022-05-09

Signering av etikprövningsansökan

Grundansökan

Forskningshuvudman: Region Stockholm
Projekttitel: Lev-projektet: Kartläggning, bedömning och intervention av hälsorelaterade levnadsvanor hos vuxna med och utan funktionsnedsättning.

I och med att ansökan undertecknas intygar du som är behörig företrädare följande:

- Att den information som lämnas i ansökan om etikprövning och samtliga medföljande bilagor är riktig och fullständig.
- Att verksamhetsansvariga i samtliga medverkande verksamheter är informerade om forskningsprojektets innehåll och utförande och att de har samtyckt till att delta i studien.
- Att du säkerställt att det i samtliga medverkande verksamheter finns resurser som garanterar forskningspersonernas säkerhet och integritet vid genomförandet av den forskning som beskrivs i ansökan.
- Att ansvarig forskare ges rätt att företräda huvudmannen i alla framtida kontakter med Etikprövningsmyndigheten som rör detta forskningsprojekt samt ansöka om ändringar i forskningsprojektet.
- Att du tagit del av Etikprövningsmyndighetens information om hantering av personuppgifter på myndighetens webbplats.

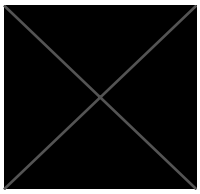

Behörig företrädare har signerat.

Signerat av JOAKIM LAVESSON (197502276616) 2022-05-23 16:38:43

Etikprövningsmyndigheten
2022-02920-01-272652
2022-05-23

Signering av etikprövningsansökan

Grundansökan

Forskningshuvudman: Region Stockholm
Projekttitel: Lev-projektet: Kartläggning, bedömning och intervention av hälsorelaterade levnadsvanor hos vuxna med och utan funktionsnedsättning.

I och med att ansökan undertecknas intygar du som är ansvarig forskare följande:

- Att den information som lämnas i ansökan om etikprövning och samtliga medföljande bilagor är riktig och fullständig.
- Att verksamhetsansvariga i samtliga medverkande verksamheter är informerade om forskningsprojektets innehåll och utförande och att de har samtyckt till att delta i studien.
- Att du säkerställt att det i samtliga medverkande verksamheter finns resurser som garanterar forskningspersonernas säkerhet och integritet vid genomförandet av den forskning som beskrivs i ansökan.
- Att du tagit del av Etikprövningsmyndighetens information om hantering av personuppgifter på myndighetens webbplats.

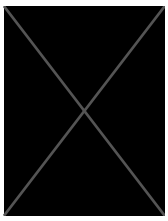

Ansvarig forskare har signerat.

Signerat av Stig Douglas Filip Sjöwall (198112210391) 2022-05-23 16:28:50

Etikprövningsmyndigheten
2022-02920-01-272644
2022-05-23

Signering av etikprövningsansökan komplettering efter beslut

Grundansökan

Forskningshuvudman: Region Stockholm
Projekttitel: Lev-projektet: Kartläggning, bedömning och intervention av hälsorelaterade levnadsvanor hos vuxna med och utan funktionsnedsättning.

I och med att ansökan undertecknas intygar du som är ansvarig forskare samt du följande:

- Att den information som lämnas i ansökan om etikprövning och samtliga medföljande bilagor är riktig och fullständig.
- Att verksamhetsansvariga i samtliga medverkande verksamheter är informerade om forskningsprojektets innehåll och utförande och att de har samtyckt till att delta i studien.
- Att du säkerställt att det i samtliga medverkande verksamheter finns resurser som garanterar forskningspersonernas säkerhet och integritet vid genomförandet av den forskning som beskrivs i ansökan.
- Att du tagit del av Etikprövningsmyndighetens information om hantering av personuppgifter på myndighetens webbplats.

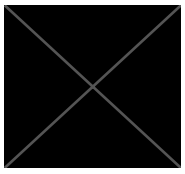

Ansvarig forskare har signerat.

Signerat av Stig Douglas Filip Sjöwall (198112210391) 2022-07-07 15:37:57

Etikprövningsmyndigheten
2022-02920-01-290895
2022-07-07

Signering av etikprövningsansökan administrativt tillägg

Grundansökan

Forskningshuvudman: Region Stockholm
Projekttitel: Lev-projektet: Kartläggning, bedömning och intervention av hälsorelaterade levnadsvanor hos vuxna med och utan funktionsnedsättning.

I och med att ansökan undertecknas intygar du som är ansvarig forskare samt du följande:

- Att den information som lämnas i ansökan om etikprövning och samtliga medföljande bilagor är riktig och fullständig.
- Att verksamhetsansvariga i samtliga medverkande verksamheter är informerade om forskningsprojektets innehåll och utförande och att de har samtyckt till att delta i studien.
- Att du säkerställt att det i samtliga medverkande verksamheter finns resurser som garanterar forskningspersonernas säkerhet och integritet vid genomförandet av den forskning som beskrivs i ansökan.
- Att du tagit del av Etikprövningsmyndighetens information om hantering av personuppgifter på myndighetens webbplats.

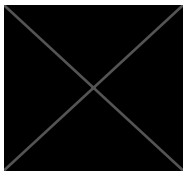

Ansvarig forskare har signerat.

Signerat av Stig Douglas Filip Sjöwall (198112210391) 2022-07-08 10:58:36

Etikprövningsmyndigheten
2022-02920-01-291222
2022-07-08

Signering av etikprövningsansökan komplettering efter beslut

Grundansökan

Forskningshuvudman: Region Stockholm
Projekttitel: Lev-projektet: Kartläggning, bedömning och intervention av hälsorelaterade levnadsvanor hos vuxna med och utan funktionsnedsättning.

I och med att ansökan undertecknas intygar du som är ansvarig forskare samt du följande:

- Att den information som lämnas i ansökan om etikprövning och samtliga medföljande bilagor är riktig och fullständig.
- Att verksamhetsansvariga i samtliga medverkande verksamheter är informerade om forskningsprojektets innehåll och utförande och att de har samtyckt till att delta i studien.
- Att du säkerställt att det i samtliga medverkande verksamheter finns resurser som garanterar forskningspersonernas säkerhet och integritet vid genomförandet av den forskning som beskrivs i ansökan.
- Att du tagit del av Etikprövningsmyndighetens information om hantering av personuppgifter på myndighetens webbplats.

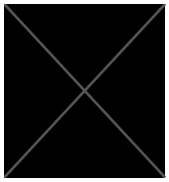

Ansvarig forskare har signerat.

Signerat av Stig Douglas Filip Sjöwall (198112210391) 2022-09-09 09:50:38

Etikprövningsmyndigheten
2022-02920-01-305701
2022-09-09

Lev-projektet: Kartläggning, bedömning och intervention av hälsorelaterade levnadsvanor hos vuxna med och utan funktionsnedsättning.

Information till forskningspersoner

Vi vill fråga dig som är över 18 år om du vill delta i ett forskningsprojekt. I det här dokumentet får du information om projektet och om vad det innebär att delta.

Vad är det för ett projekt och varför vill ni att jag ska delta?

Ohälsosamma levnadsvanor leder enligt Världshälsoorganisationen (WHO) till psykisk och fysisk ohälsa. För att du, på egen hand eller tillsammans med hälso- och sjukvård, ska kunna må bättre är ett viktigt steg att kunna mäta hur dina levnadsvanor ser ut. I det här projektet utvärderar vi ett nytt frågeformulär som ska göra det enklare att mäta levnadsvanor (Lev-s).

Forskningshuvudman för projektet är Habilitering & Hälsa, Region Stockholm. Med forskningshuvudman menas den organisation som är ansvarig för projektet. Ansökan är godkänd av Etikprövningsmyndigheten, diarienummer för prövningen hos Etikprövningsmyndigheten är ange diarienummer

Hur går projektet till?

Om du deltar kommer du få svara på frågor vid 2 tillfällen. Länk till studien finns längst ned i detta dokument.

- Vid första tillfället fyller du i tre formulär: ett om levnadsvanor (Lev-s) och ett om livskvalitet (WHOQOL) samt ett om bakgrundsinformation.
- Efter ca 2 veckor fyller klicka du på länken igen och i formuläret om levnadsvanor (Lev-s) en gång till. Det är ett sätt att testa hur säkert formuläret är. Om du inte ändrat dina levnadsvanor ska resultatet vara ungefär samma som gången innan. Skriv upp i din kalender att du ska gå in igen om två veckor och fylla i.

Ta dig tid och fundera på om du vill vara med.

Möjliga följder och risker med att delta i projektet

Riskerna är låga. Vissa kan uppleva en del frågor som privata men vi kommer inte berätta hur du svarat för någon annan. De områden vi frågar om är tobak, alkohol, kost, fysisk aktivitet, droger, sömn, vänner, sex, intressen, skärmtid. Du har rätt att avstå att svara på enskilda frågor om du vill. Frågorna är utvalda att visa på vad som är viktigt att tänka på inom varje levnadsvana. En positiv effekt kan därför bli att man blir medveten om hur man kan leva mer hälsosamt. Om du skulle må sämre efter att ha svarat på frågorna ska du kontakta en läkare eller psykolog för stöd.

Vad händer med mina uppgifter?

Svaren förvaras i en databas på ett USB-minne inlåst i ett skåp. Vi är inte intresserade enskilda individer utan kommer analysera svaren på gruppnivå. Du ska inte fylla i namn eller

Etikprövningsmyndigheten
2022-02920-01-291211
2022-07-08

personnummer. Vi använder svaren du ger i bakgrundsinformationen för att para ihop mätningarna från de olika tillfällena. Även om vi inte är intresserade av det och inte kommer publicera några sådana uppgifter är vi skyldiga att informera dig om att det är möjligt att kombinationen av unika svar kan göra det möjligt att identifiera vem du är i vissa fall. Ingen obehörig kommer att få ta del av dina svar.

Behandlingen av dina personuppgifter är nödvändigt för att utföra forskning som är av allmänt intresse (GDPR, Art 6, p. 1e). Ansvarig för dina personuppgifter är Habilitering & Hälsa, Regions Stockholm. Enligt EU:s dataskyddsförordning har du rätt att kostnadsfritt få ta del av de uppgifter om dig som hanteras i projektet, och vid behov få eventuella fel rättade. Du kan också begära att uppgifter om dig raderas samt att behandlingen av dina personuppgifter begränsas. Det är dock väldigt sannolikt att vi inte kommer kunna identifiera vem du är och därför kommer ha svårt att identifiera dina uppgifter. Rätten till radering och till begränsning av behandling av personuppgifter gäller dock inte när uppgifterna är nödvändiga för den aktuella forskningen. Data sparas i 10 år. Om du vill ta del av uppgifterna ska du kontakta Douglas Sjöwall Habilitering & Hälsa, Box 454 36, 104 31 Stockholm 08-123 350 10. Dataskyddsombud Camilla Heise Löwgren nås på camilla.heise-lowgren@regionstockholm.se Om du är missnöjd med hur dina personuppgifter behandlas har du rätt att ge in klagomål till Integritetsskyddsmyndigheten, som är tillsynsmyndighet.

Etikprövningsmyndigheten
2022-02920-01-291211
2022-07-08

Hur får jag information om resultatet av projektet?

Du behöver inte ta del av studiens resultat. Om du vill ta del av resultatet av hela projektet hänvisar vi dig till de vetenskapliga artiklar som vi kommer sammanställa. Det kan dröja några år innan studien publiceras. Kontakta projektledare (Douglas Sjöwall) om du har frågor gällande resultaten från projektet.

Försäkring och ersättning

Vi erbjuder inte ersättning och kan inte erbjuda försäkring då vi inte vet vem du är.

Deltagandet är frivilligt

Ditt deltagande är frivilligt och du kan när som helst välja att avbryta din medverkan. Om du väljer att inte delta eller vill avbryta ditt deltagande behöver du inte uppge varför, och det kommer inte heller att påverka din framtida vård eller behandling.

Ansvariga för projektet

Projektledare för projektet är Douglas Sjöwall Habilitering & Hälsa, Box 454 36, 104 31 Stockholm 08-123 350 10. Douglas.sjowall@regionstockholm.se

Klicka på länken nedan om du samtycker till att delta i studien:

Länk till elektronisk enkät

Lev-projektet: Kartläggning, bedömning och intervention av hälsorelaterade levnadsvanor hos vuxna med och utan funktionsnedsättning.

Information till forskningspersoner

Vi vill fråga dig som är över 18 år om du vill delta i ett forskningsprojekt. I det här dokumentet får du information om projektet och om vad det innebär att delta.

Vad är det för ett projekt och varför vill ni att jag ska delta?

Ohälsosamma levnadsvanor leder enligt Världshälsoorganisationen (WHO) till psykisk och fysisk ohälsa. För att du, på egen hand eller tillsammans med hälso- och sjukvård, ska kunna må bättre är ett viktigt steg att kunna mäta hur dina levnadsvanor ser ut. I det här projektet utvärderar vi ett nytt frågeformulär som ska göra det enklare att mäta levnadsvanor (Lev-s).

Forskningshuvudman för projektet är Habilitering & Hälsa, Region Stockholm. Med forskningshuvudman menas den organisation som är ansvarig för projektet. Ansökan är godkänd av Etikprövningsmyndigheten, diarienummer för prövningen hos Etikprövningsmyndigheten är ange diarienummer

Hur går projektet till?

Om du deltar kommer du få svara på frågor vid 2 tillfällen.

- Vid första tillfället fyller du i tre formulär: ett om levnadsvanor (Lev-s) och ett om livskvalitet (WHOQOL) samt ett om bakgrundsinformation.
- Efter ca 2 veckor fyller du i formuläret om levnadsvanor (Lev-s) igen. Det är ett sätt att testa hur säkert formuläret är. Om du inte ändrat dina levnadsvanor ska resultatet vara ungefär samma som gången innan.

Ta dig tid och fundera på om du vill vara med. Om du vill delta ska du skriva under samtycket på nästa sida. Sedan fyller du i frågorna på egen hand. Vi bokar dessutom in en tid om två veckor då vi ses online/på plats. Då kommer du få samma frågor men skillnaden är att vi ställer frågorna. Vi gör det för att studera om vi får samma svar när deltagare gör själva som när vi ställer frågorna.

Möjliga följder och risker med att delta i projektet

Riskerna är låga. Vissa kan uppleva en del frågor som privata men vi kommer inte berätta hur du svarat för någon annan. De områden vi frågar om är tobak, alkohol, kost, fysisk aktivitet, droger, sömn, vänner, sex, intressen, skärmtid. Du har rätt att avstå att svara på enskilda frågor om du vill. Om du skulle må sämre efter att ha svarat på frågorna kan vi sätta dig i kontakt med lämpligt stöd. Frågorna är utvalda att visa på vad som är viktigt att tänka på inom varje levnadsvana. En positiv effekt kan därför bli att man blir medveten om hur man kan leva mer hälsosamt.

Vad händer med mina uppgifter?

Etikprövningsmyndigheten
2022-02920-01-291213
2022-07-08

Lev-projektet: Kartläggning, bedömning och intervention av hälsorelaterade levnadsvanor hos vuxna med och utan funktionsnedsättning.

Svaren förvaras under datainsamlingen inlåsta i ett journalskåp. Svaren på frågorna läggs sedan in i en datafil. I stället för att lägga in ditt namn används en kod. Excelfilen med koppling mellan kod och ditt namn förvaras inlåst på Karolinska Institutet och på Habilitering & Hälsa, Region Stockholm. Ingen obehörig kommer att få ta del av dina svar. Kodnyckeln förvaras separerad från databasen på Habilitering & Hälsa, Region Stockholm.

Behandlingen av dina personuppgifter är nödvändigt för att utföra forskning som är av allmänt intresse (GDPR, Art 6, p. 1e). Ansvarig för dina personuppgifter är Habilitering & Hälsa, Regions Stockholm. Enligt EU:s dataskyddsförordning har du rätt att kostnadsfritt få ta del av de uppgifter om dig som hanteras i projektet, och vid behov få eventuella fel rättade. Du kan också begära att uppgifter om dig raderas samt att behandlingen av dina personuppgifter begränsas. Rätten till radering och till begränsning av behandling av personuppgifter gäller dock inte när uppgifterna är nödvändiga för den aktuella forskningen. Data sparas i 10 år. Om du vill ta del av uppgifterna ska du kontakta Douglas Sjöwall Habilitering & Hälsa, Box 454 36, 104 31 Stockholm 08-123 350 10. Dataskyddsombud Camilla Heise Löwgren nås på camilla.heise-lowgren@regionstockholm.se Om du är missnöjd med hur dina personuppgifter behandlas har du rätt att ge in klagomål till Integritetsskyddsmyndigheten, som är tillsynsmyndighet.

Etikprövningsmyndigheten
2022-02920-01-291213
2022-07-08

Hur får jag information om resultatet av projektet?

Du behöver inte ta del av dina eller studiens resultat. Om du vill ta del av resultatet av hela projektet hänvisar vi dig till de vetenskapliga artiklar som vi kommer sammanställa. Det kan dröja några år innan studien publiceras. Kontakta projektledare (Douglas Sjöwall) om du har frågor gällande resultaten från projektet.

Försäkring och ersättning

Sedvanligt patientförsäkringsskydd gäller för deltagarna som rekryteras via kliniska verksamheter.

Deltagandet är frivilligt

Ditt deltagande är frivilligt och du kan när som helst välja att avbryta deltagandet. Om du väljer att inte delta eller vill avbryta ditt deltagande behöver du inte uppge varför, och det kommer inte heller att påverka din framtida vård eller behandling.

Om du vill avbryta ditt deltagande ska du kontakta den ansvariga för projektet (se nedan).

Ansvariga för projektet

Projektledare är Douglas Sjöwall Habilitering & Hälsa, Box 454 36, 104 31 Stockholm 08-123 350 10. Douglas.sjowall@regionstockholm.se

Lev-projektet: Kartläggning, bedömning och intervention av hälsorelaterade levnadsvanor hos vuxna med och utan funktionsnedsättning.

Information till forskningspersoner

Vi vill fråga dig som är över 18 år om du vill delta i ett forskningsprojekt. I det här dokumentet får du information om projektet och om vad det innebär att delta.

Vad är det för ett projekt och varför vill ni att jag ska delta?

Ohälsosamma levnadsvanor leder enligt Världshälsoorganisationen (WHO) till psykisk och fysisk ohälsa. Forskare vid Karolinska Institutet och Habilitering & Hälsa Region Stockholm, undersöker hur levnadsvanor ser ut och vilka levnadsvanor deltagarna med olika bakgrund tycker känns viktigast. Vi undersöker även vilket ytterligare stöd deltagarna är i behov av för att kunna leva hälsosammare. Syftet med studien är att vården i Sverige ska bli bättre på att ge stöd som leder till bättre levnadsvanor.

Forskningshuvudman för projektet är Habilitering & Hälsa, Region Stockholm. Med forskningshuvudman menas den organisation som är ansvarig för projektet. Ansökan är godkänd av Etikprövningsmyndigheten, diarienummer för prövningen hos Etikprövningsmyndigheten är ange diarienummer

Etikprövningsmyndigheten
2022-02920-01-291214
2022-07-08

Hur går projektet till?

Om du deltar kommer du få fylla i i tre formulär: ett om levnadsvanor (Lev-s) och ett om livskvalitet (WHOQOL) samt ett om bakgrundsinformation och vilka levnadsvanor du tycker är viktigast och vilket ytterligare stöd du skulle vilja ha.

Ta dig tid och fundera på om du vill vara med. Om du vill delta klickar du på följande länken längst ned.

Möjliga följder och risker med att delta i projektet

Riskerna är låga. Vissa kan uppleva en del frågor som privata men vi kommer inte berätta hur du svarat för någon annan. De områden vi frågar om är tobak, alkohol, kost, fysisk aktivitet, droger, sömn, vänner, sex, intressen, skärmtid. Du har rätt att avstå att svara på enskilda frågor om du vill. Frågorna är utvalda att visa på vad som är viktigt att tänka på inom varje levnadsvana. En positiv effekt kan därför bli att man blir medveten om hur man kan leva mer hälsosamt. Om du skulle må sämre efter att ha svarat på frågorna bör du ta kontakt med läkare eller psykolog.

Vad händer med mina uppgifter?

Svaren förvaras i en databas på ett USB-minne inlåst i ett skåp. Vi är inte intresserade enskilda individer utan kommer analysera svaren på gruppnivå. Du ska inte fylla i namn eller personnummer. Även om vi inte är intresserade av det och inte kommer publicera några sådana uppgifter är vi skyldiga att informera dig om att det är möjligt att kombinationen av

unika svar kan göra det möjligt att identifiera vem du är i vissa fall. Ingen obehörig kommer att få ta del av dina svar.

Ansvarig för dina personuppgifter är Habilitering & Hälsa, Regions Stockholm. Enligt EU:s dataskyddsförordning har du rätt att kostnadsfritt få ta del av de uppgifter om dig som hanteras i projektet, och vid behov få eventuella fel rättade. Du kan också begära att uppgifter om dig raderas samt att behandlingen av dina personuppgifter begränsas. Det är dock väldigt sannolikt att vi inte kommer kunna identifiera vem du är och därför kommer ha svårt att identifiera dina uppgifter. Rätten till radering och till begränsning av behandling av personuppgifter gäller dock inte när uppgifterna är nödvändiga för den aktuella forskningen. Data sparas i 10 år. Om du vill ta del av uppgifterna ska du kontakta Douglas Sjöwall Habilitering & Hälsa, Box 454 36, 104 31 Stockholm 08-123 350 10. Dataskyddsombud Camilla Heise Löwgren nås på camilla.heise-lowgren@regionstockholm.se Om du är missnöjd med hur dina personuppgifter behandlas har du rätt att lämna klagomål till Integritetsskyddsmyndigheten, som är tillsynsmyndighet.

Hur får jag information om resultatet av projektet?

Du behöver inte ta del av studiens resultat. Om du vill ta del av resultatet av hela projektet hänvisar vi dig till de vetenskapliga artiklar som vi kommer sammanställa. Det kan dröja några år innan studien publiceras. Kontakta projektansvarig (Douglas Sjöwall) om du har frågor gällande resultaten från projektet.

Försäkring och ersättning

Vi erbjuder inte ersättning och kan inte erbjuda försäkring då vi inte vet vem du är.

Deltagandet är frivilligt

Ditt deltagande är frivilligt och du kan när som helst välja att avbryta deltagandet. Om du väljer att inte delta eller vill avbryta ditt deltagande behöver du inte uppge varför, och det kommer inte heller att påverka din framtida vård eller behandling.

Ansvariga för projektet

Projektledare är Douglas Sjöwall Habilitering & Hälsa, Box 454 36, 104 31 Stockholm 08-123 350 10. Douglas.sjowall@regionstockholm.se

Klicka på länken nedan om du samtycker till att delta i studien:

Länk till elektronisk enkät

Etikprövningsmyndigheten
2022-02920-01-291214
2022-07-08

Lev-projektet: Kartläggning, bedömning och intervention av hälsorelaterade levnadsvanor hos vuxna med och utan funktionsnedsättning.

Information till forskningspersoner

Vi vill fråga dig om du vill delta i ett forskningsprojekt. I det här dokumentet får du information om projektet och om vad det innebär att delta i Lev - genomförbarhetsstudien. Om du vill delta efter att ha läst igenom informationen, skriver du under samtycket längst bak och ger till behandlaren som du fick information om studien av.

Vad är det för ett projekt och varför vill ni att jag ska delta?

Enligt Världshälsoorganisationen (WHO) kan vi må bättre och leva längre om vi har hälsosammare levnadsvanor. Hälso- och sjukvård behöver bli bättre på att ge stöd och insatser som syftar till hälsosammare levnadsvanor hos personer med någon form av funktionsnedsättning. I det här projektet utvärderar vi en ny insats (Lev) som har som syfte att förbättra levnadsvanor. Lev har utvecklats i ett samarbete mellan forskare, aktörer inom hälso- och sjukvård samt representanter för olika funktionsnedsättningar.

Vi söker dig som:

- vill förbättra dina levnadsvanor,
- har någon form av funktionsnedsättning eller diagnos (exempelvis autism, ADHD, intellektuell funktionsnedsättning, förvärvad hjärnskada, rörelsenedsättning, dövblindhet, dövhet),
- vill ge synpunkter på insatsen så att vi kan fortsätta att utveckla den.

Om du är intresserad fyller du i samtycket som finns längst bak. Du får då en individuell bedömning av en behandlare där du fick information om studien. Efter det får du besked om du kan erbjudas plats i studien.

Forskningshuvudman för projektet är Habilitering & Hälsa, Region Stockholm. Med forskningshuvudman menas den organisation som är ansvarig för projektet. Ansökan är godkänd av Etikprövningsmyndigheten, diarienummer för prövningen hos Etikprövningsmyndigheten är ange diarienummer

Hur går projektet till?

Att delta i genomförbargetsstudien innebär följande:

- **Mätning:** En utvärdering dina levnadsvanor
- **Insatsen (Lev):**
    - Fas 1: Samtal om levnadsvanor, målsättning + eventuellt stöd från annan aktör inom hälso- och sjukvård.
    - Fas 2: Fördjupad analys som leder till individuella strategier och stöd för hur du ska nå dina mål.

Etikprövningsmyndigheten
2022-02920-01-291215
2022-07-08

Lev-projektet: Kartläggning, bedömning och intervention av hälsorelaterade levnadsvanor hos vuxna med och utan funktionsnedsättning.

- Fas 3: Avslut och ny utvärdering av levnadsvanor, målsättning och ifyllnad av självskattningsformulär. Plan för eget arbete.

De 3 faserna i Lev ges under ca 2-6 månader. Du fyller i skattningar före under och efter deltagande i insatsen. Skattningarna inkluderar frågor om livskvalitet, vad du tycker om behandlingen och dess delar samt bakgrundsinformation (exempelvis kön, din ålder, sysselsättning och om du har någon funktionsnedsättning).

Möjliga följder och risker med att delta i projektet

Riskerna är låga. Vissa kan uppleva en del frågor som privata. De områden vi frågar om är tobak, alkohol, kost, fysisk aktivitet, droger, sömn, vänner, sex, intressen, skärmtid. Du har rätt att avstå att svara på enskilda frågor om du vill och du väljer själv vilken levnadsvana du vill fokusera på. Vi kommer inte berätta för någon hur du svarat. Om du mot förmodan skulle må sämre efter att ha fått insatsen kan vi sätta dig i kontakt med lämpligt stöd. Positiva följder kan vara att du mår bättre psykiskt och fysiskt.

Vad händer med mina uppgifter?

Projektet kommer att samla in och registrera information om dig.

Svaren förvaras under datainsamlingen inlåsta i ett journalskåp. Svaren på frågorna läggs sedan in i en datafil. I stället för att lägga in ditt namn används en kod som en säkerhetsåtgärd för att skydda din identitet. Kodnyckeln, som kan koppla ihop din kod med ditt namn, förvaras separerad från databasen på Habilitering & Hälsa, Region Stockholm. Datafilen förvaras inlåst på Karolinska Institutet och på Habilitering & Hälsa, Region Stockholm. Ingen obehörig kommer att få ta del av dina svar.

Behandlingen av dina personuppgifter är nödvändigt för att utföra forskning som är av allmänt intresse (GDPR, Art 6, p. 1e). Ansvarig för dina personuppgifter är Habilitering & Hälsa, Regions Stockholm. Enligt EU:s dataskyddsförordning har du rätt att kostnadsfritt få ta del av de uppgifter om dig som hanteras i projektet, och vid behov få eventuella fel rättade. Du kan också begära att uppgifter om dig raderas samt att behandlingen av dina personuppgifter begränsas. Rätten till radering och till begränsning av behandling av personuppgifter gäller dock inte när uppgifterna är nödvändiga för den aktuella forskningen. Data sparas i 10 år. Om du vill ta del av uppgifterna ska du kontakta Douglas Sjöwall Habilitering & Hälsa, Box 454 36, 104 31 Stockholm 08-123 350 10. Dataskyddsombud Camilla Heise Löwgren nås på camilla.heise-lowgren@regionstockholm.se. Om du är missnöjd med hur dina personuppgifter behandlas har du rätt att ge in klagomål till Integritetsskyddsmyndigheten, som är tillsynsmyndighet.

Hur får jag information om resultatet av projektet?

Etikprövningsmyndigheten
2022-02920-01-291215
2022-07-08

Lev-projektet: Kartläggning, bedömning och intervention av hälsorelaterade levnadsvanor hos vuxna med och utan funktionsnedsättning.

Du behöver inte ta del av dina eller studiens resultat. Om du vill ta del av resultatet av hela projektet hänvisar vi dig till de vetenskapliga artiklar som vi kommer sammanställa. Det kan dröja några år innan studien publiceras. Kontakta projektansvarig (Douglas Sjöwall) om du har frågor gällande resultaten från projektet.

Försäkring och ersättning

Sedvanligt patientförsäkringsskydd gäller för deltagarna via de kliniska verksamheter som utför insatsen.

Ingen ersättning utgår för deltagande i projektet.

Deltagandet är frivilligt

Ditt deltagande är frivilligt och du kan när som helst välja att avbryta deltagandet. Om du väljer att inte delta eller vill avbryta ditt deltagande behöver du inte uppge varför, och det kommer inte heller att påverka din framtida vård eller behandling.

Ansvariga för projektet

Projektledare för projektet är Douglas Sjöwall Habilitering & Hälsa, Box 454 36, 104 31 Stockholm 08-123 350 10. Douglas.sjowall@regionstockholm.se

Etikprövningsmyndigheten
2022-02920-01-291215
2022-07-08

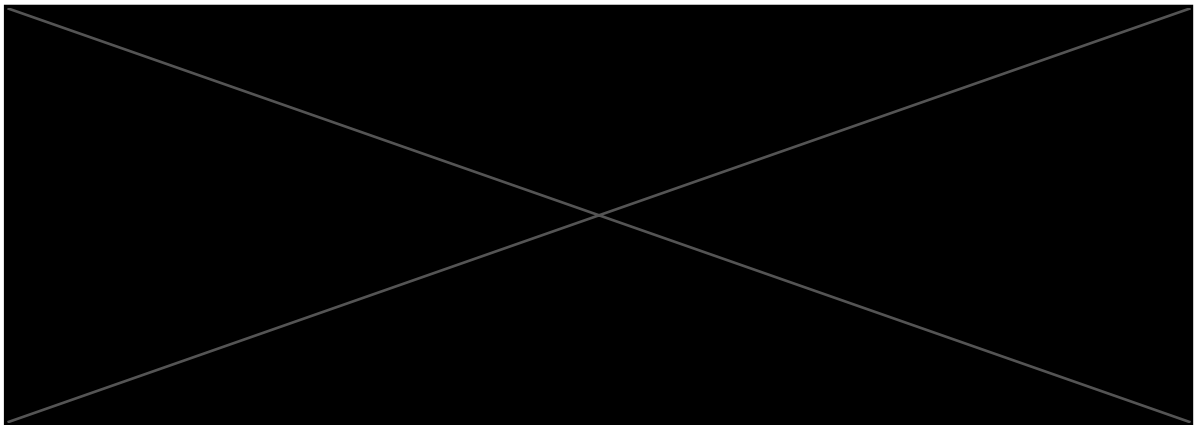

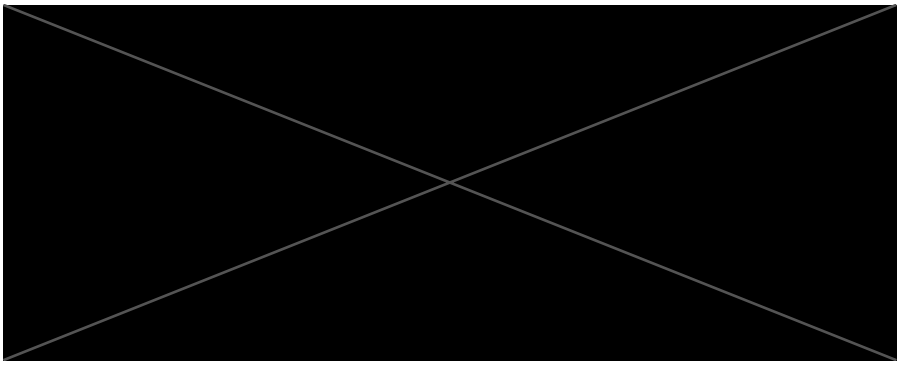

Etikprövningsmyndigheten
2022-02920-01-290872
2022-07-07

Vill du vara med i ett projekt som syftar till att mäta levnadsvanor?

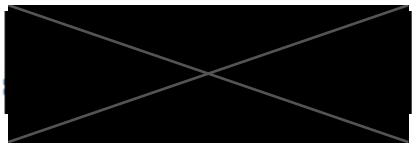

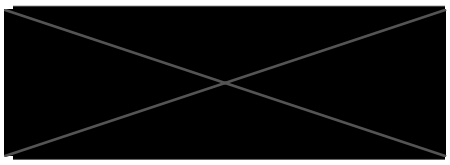

Vill du delta i en studie som undersöker levnadsvanor hos vuxna i Sverige? Hälsosamma levnadsvanor har stor betydelse för psykisk och fysisk välmående, enligt Världshälsoorganisationen (WHO). Forskare vid Karolinska Institutet och Habilitering & Hälsa Region Stockholm, testar ett nytt frågeformulär som ska göra det enklare att utvärdera levnadsvanor och livskvalitet hos personer med och utan funktionsnedsättningar.

Vill du veta mer om studien och hur man deltar? Klicka på länken nedan:

Länk till forskningspersonsinformation

Om du har frågor om projektet går det bra att vända sig till ansvarig för projektledare Douglas Sjöwall, forskare verksam vid Habilitering & Hälsa samt Karolinska Institutet. Douglas.sjowall@regionstockholm.se Habilitering & Hälsa, Box 454 36, 104 31 Stockholm 08-123 350 10.

Etikprövningsmyndigheten
2022-02920-01-290872
2022-07-07

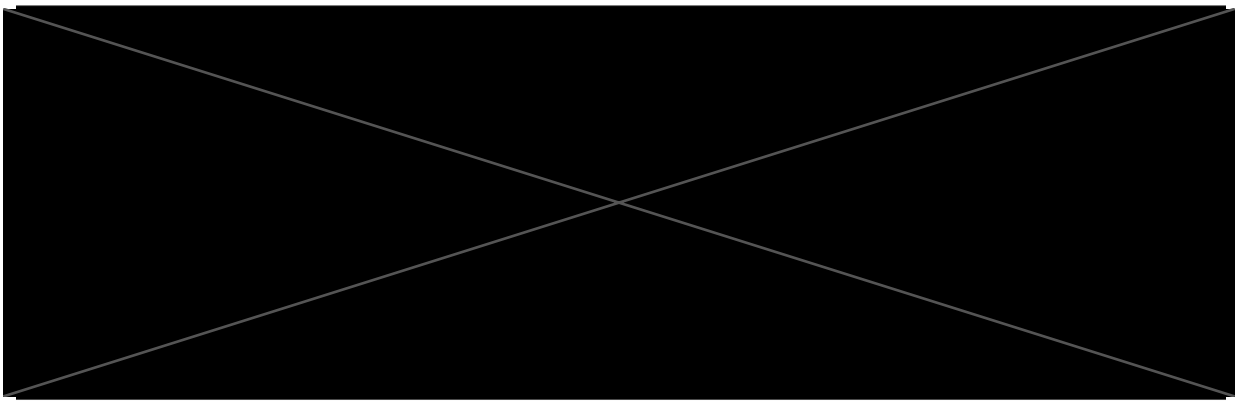

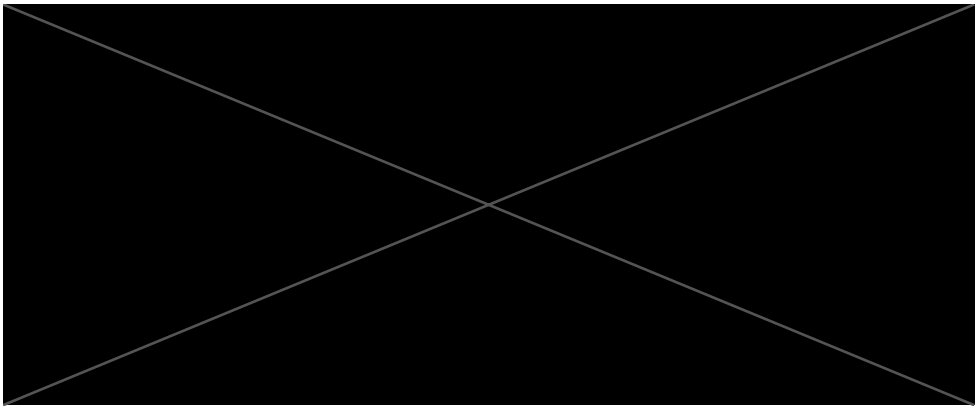

Etikprövningsmyndigheten
2022-02920-01-290873
2022-07-07

Vill du vara med i en forskningsstudie som undersöker levnadsvanor hos vuxna med och utan funktionsnedsättning?

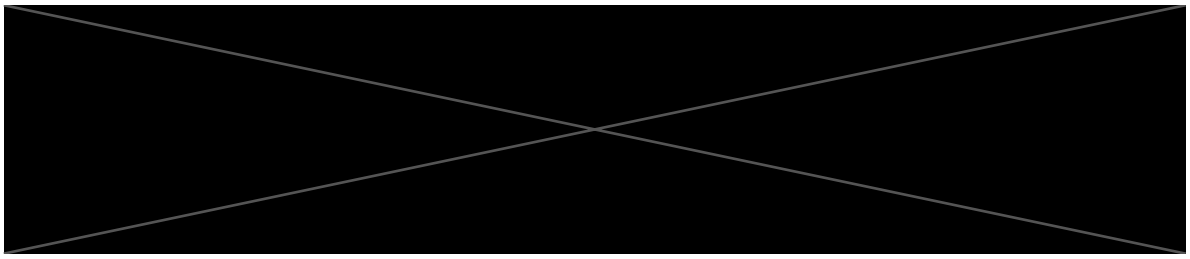

Vill du delta i en studie som undersöker levnadsvanor hos vuxna i Sverige? Hälsosamma levnadsvanor har stor betydelse för psykisk och fysisk välmående, enligt Världshälsoorganisationen (WHO). Forskare vid Karolinska Institutet och Habilitering & Hälsa Region Stockholm, undersöker hur levnadsvanor ser ut och vilka levnadsvanor som deltagarna tycker känns viktigast. Vi undersöker även vilket ytterligare stöd deltagarna vill ha för att kunna leva hälsosammare.

Vill du veta mer om studien och hur man deltar? Klicka på länken nedan:

Länk till forskningspersonsinformation

Om du har frågor om projektet går det bra att vända sig till projektledare Douglas Sjöwall, forskare verksam vid Habilitering & Hälsa samt Karolinska Institutet. Douglas.sjowall@regionstockholm.se Habilitering & Hälsa, Box 454 36, 104 31 Stockholm 08-123 350 10.

Etikprövningsmyndigheten
2022-02920-01-290873
2022-07-07

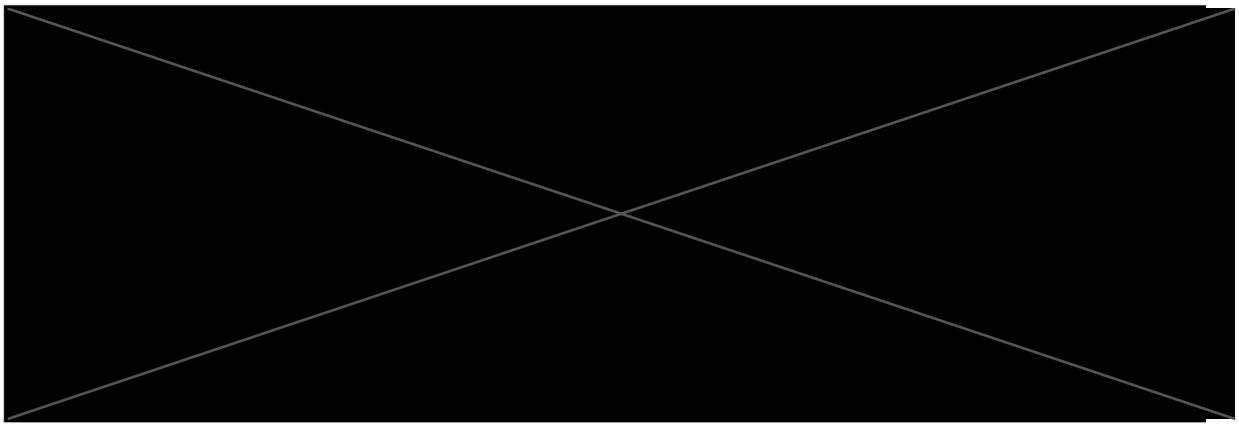

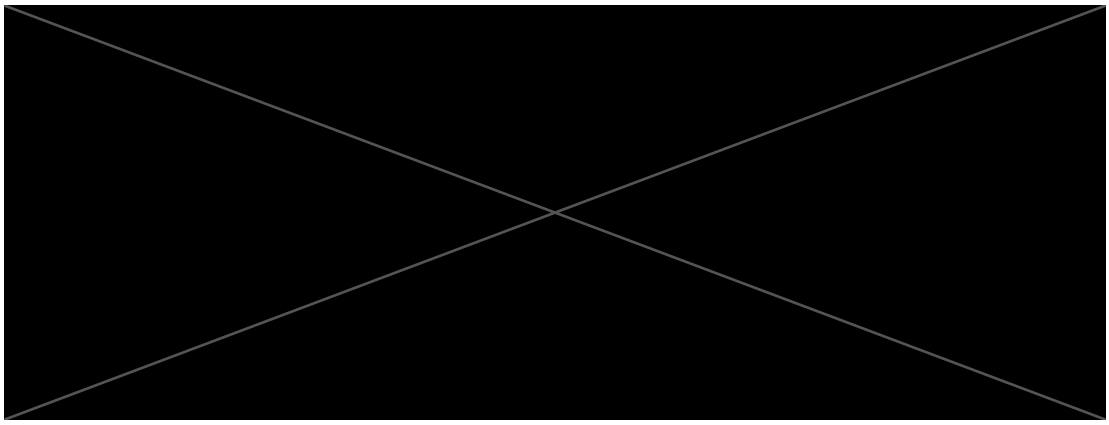

Etikprövningsmyndigheten
2022-02920-01-290874
2022-07-07

Vill du delta i en insats som syftar till att förbättra din psykiska och fysiska hälsa genom ändrade levnadsvanor?

Läs mer på nästa sida.

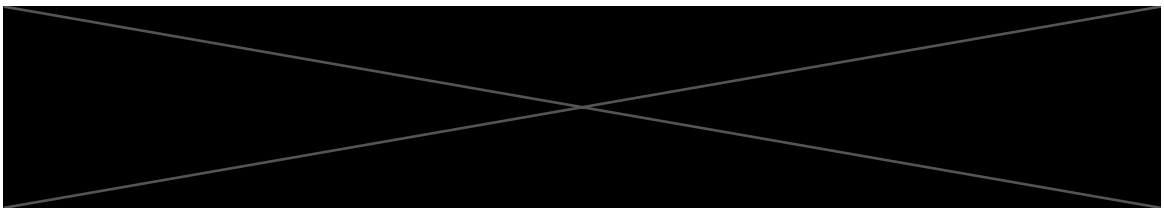

Vill du delta i ett projekt om syftar till att förbättra levnadsvanor och samtidigt bidra till forskning och utveckling?

Hälsosamma levnadsvanor har stor betydelse för psykisk och fysisk välmående, enligt Världshälsoorganisationen (WHO). Forskare vid Karolinska Institutet och Habilitering & Hälsa Region Stockholm, har utvecklat ett nytt sätt att stötta personer över 18 år som vill förbättra sina levnadsvanor. Nu är det viktigt att vi får testa om det fungerar och även ge deltagarna möjlighet att komma med synpunkter på hur vi kan fortsätta att förbättra insatsen.

Vill du veta mer om studien och hur man deltar? Klicka på länken nedan:

Länk till forskningspersonsinformation

Om du har frågor om projektet går det bra att vända sig till projektledare Douglas Sjöwall, forskare verksam vid Habilitering & Hälsa samt Karolinska Institutet. Douglas.sjowall@regionstockholm.se Habilitering & Hälsa, Box 454 36, 104 31 Stockholm 08-123 350 10.

Etikprövningsmyndigheten
2022-02920-01-290874
2022-07-07

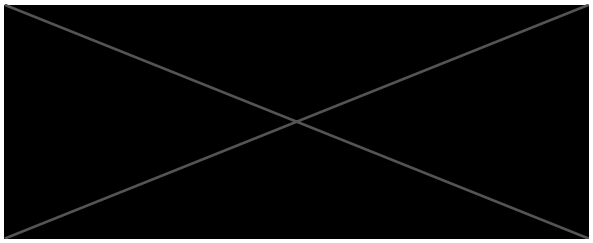

2022

Etikprövningsmyndigheten
2022-02920-01-290879
2022-07-07

Lev – Insats för hälsosammare levnadsvanor: praktiskt genomförande

En transdiagnostisk insats för vuxna

HABILITERING & HÄLSA, REGION STOCKHOLM

Så här använder du manualen....3
Skattning av levnadsvanor (Lev-s)....4
Fas 1: Lyft frågan om levnadsvanor....16
Introduktion till deltagarens arbetsmaterialmaterial....16
Agenda....17
Val av område (alternativ start)....19
Målsättning....19
Samverkan....20
Journalskrivning och efterarbete....21
Fas 2: Stöd i genomförande....21
Agenda....22
Funktionell analys....23
Beteendestödsplan....23
Journalskrivning och efterarbete....24
Fas 3 – Uppföljning och vidmakthållande....25
Målsättning (Uppföljning)....25
Val av nytt område....26
Målsättning (ny)....26
Journalskrivning och efterarbete....27
Enkla råd – bilaga 1....27
Tobak....28
Kost....30
Alkohol....34
Fysisk aktivitet....37
Droger....40
Skärmtid....43
Sömn....45
Intressen/Aktiviteter/Sysselsättning....47
Sexuell hälsa....49
Vänner/Familj....53
Exempel på beteendeanalys inklusive tips på strategier– bilaga 2....55
Tobak....55

Etikprövningsmyndigheten
2022-02920-01-290879
2022-07-07

Kost....57
Alkohol....60
Fysisk aktivitet....62
Droger....64
Skärmtid....66
Sömn....68
Intressen....70
Sexuell hälsa....72
Vänner/Familj....74
Utforska motivation – bilaga 3....76

Etikprövningsmyndigheten
2022-02920-01-290879
2022-07-07

Så här använder du manualen

Det finns två manualer*. Den teoretiska manualen* (som du bör ha läst nu) är det första man läser när man genomför sin utbildning och syftet är att den som läser ska få en bakgrund till insatsen och en övergripande bild av hur man genomför Lev. I den teoretiska manualen beskrivs även den utbildning som föreslås genomföras innan man börjar använda Lev. Nästa steg är att ta del av *”Manualen för praktiskt genomförande”* (manualen som du läser nu). Först i denna manual ligger Lev-s som du administrerar innan du ger Lev. Sedan kommer beskrivning av de tre faserna i Lev. Här står det ordagrant hur man kan lägga upp mätning och behandling. Denna del är viktig när man tränar för att kunna ge Lev och när man förbereder sig inför mötet med deltagaren. Tanken är inte att man ska följa den praktiska manualen ordagrant. Det är exempel på hur du kan formulera dig. I stället utgår man ifrån deltagarens *arbetsbok*. Arbetsboken följer samma struktur som manualen för det praktiska genomförandet men innehåller kortare formuleringar som deltagaren ska ha framför sig under genomgången. Här i skriver ni gemensamt ner mål och strategier som ni kommer överens om. Sist men inte minst, använd din erfarenhet och prioritera kommunikationen så att du får med dig deltagaren i samtalet.

Lycka till!

Etikprövningsmyndigheten
2022-02920-01-290879
2022-07-07

Skattning av levnadsvanor (Lev-s)

Bakgrund

Det här formuläret syftar till att ge en översiktlig kartläggning av levnadsvanor och att identifiera var det kan finnas ett behov att göra ändringar. Det finns etablerade mer djupgående formulär inom varje område och syftet är inte att ersätta dessa utan att fungera som ett komplement.

Individanpassningar

Du kan ibland behöva anpassa den frågan för att öka förståelsen. Detta kan du göra genom att förklara frågan mer noga, anpassa språket, ställa följdfrågor och/eller referera till information som exempelvis framkommit tidigare. Du kan även läsa svarsalternativen högt.

Poängsättning

Tanken är att du välj det svarsalternativ som passar bäst utan att läsa dem för deltagaren. Läs dock svarsalternativen högt om det underlättar. På den skalan som används (0, 1, 2, 3) är 3 poäng det högsta (indikation på hälsosam vana) och 0 poäng det lägsta (indikation för ohälsosam vana). Bedömningen för varje levnadsvana placeras ut i hjulet för att synliggöra vart det finns utrymme för hälsosammare vanor. Bedömningen baseras på ibland på medelvärdet och ibland på minimum. Till din hjälp finns en excel-mall.

Återkoppling av resultat till deltagaren

Visa resultatet med hjälp av att markera ut prickar för varje levnadsvana i översiktsfiguren. Kom ihåg att vissa deltagare kan bli nedslagna av att se sina resultat. Försök att betona att levnadsvanorna inte är egenskaper utan vanor som kan ändras och att de gjort rätt som nu tagit tag i dem. Följ upp deltagare som blir besvikna över sitt resultat. Se vidare instruktion för hur du går igenom resultatet finns i anslutning till översiktsfiguren.

Information till deltagaren

Att ta tag i sina levnadsvanor kan förändra ditt liv på ett väldigt positivt sätt. Första steget är att ta reda på hur ens levnadsvanor ser ut. Levnadsvanor är inte dina egenskaper. Det är vanor som du har just nu men som kan ändras. Även om vi identifierar områden där du skulle kunna leva hälsosammare är det helt upp till dig om vi ska gå vidare och jobba med någon levnadsvana.

I frågorna om levnadsvanor ingår det frågor som av vissa kan upplevas som känsliga. Vi har tystnadsplikt och dina svar kommer inte leda till några negativa konsekvenser. De områden vi tar upp är sömn, kost, fysisk aktivitet, intressen/aktiviteter, skärmtid, vänner/familj, tobak, alkohol, droger och sexuell hälsa. Inför varje område frågar vi om det är okej att vi ställer några frågor. Du kan välja att hoppa över ett område eller att inte svara på enskilda frågor. Är det okej att jag frågar om dina levnadsvanor? Eftersträva att förmedla denna text i god tid till deltagaren så att det finns utrymme att reflektera över om man är redo att svara på frågorna inom alla områden.

Etikprövningsmyndigheten
2022-02920-01-290879
2022-07-07

Tobak

Tobaksbruk innefattar konsumtion av cigaretter, pipa, cigarr, cigarill, vattenpipa samt rökfri tobak, snus och tuggtobak. E-cigaretter ingår inte i definitionen.

Är det okej om jag frågar om dina tobaksvanor?

Förekommer tobak

Har du någonsin rökt eller använt någon annan form av tobak?

- Ja, röker/snusar/använder tobak i dagsläget 0 poäng
- Ja, när det är fest 1 poäng
- Nej, men slutade för mindre än 6 månader sedan 2 poäng
- Nej, har aldrig rökt/slutade för mer än 6 månader sedan 3 poäng

Kvantitet

Hur mycket tobak använder du i dagsläget? *Om flera former av tobak används slås användningen ihop.*

- Använder tobak flera gånger dagligen 0 poäng
- Använder tobak, någon gång dagligen 1 poäng
- Använder tobak, men inte dagligen 2 poäng
- Använder inte tobak 3 poäng

Etikprövningsmyndigheten
2022-02920-01-290879
2022-07-07

Kost

Är det okej att jag frågar om dina matvanor?

Alla frågor handlar om hur det varit den senaste månaden.

Kostindex från Socialstyrelsen

Hur ofta äter du grönsaker och/eller rotfrukter (färska, frysta eller tillagade)?

- En gång i veckan eller mer sällan 0 poäng
- Några gånger i veckan 1 poäng

- En gång per dag 2 poäng
- Två gånger per dag eller oftare 3 poäng

Hur ofta äter du frukt och/eller bär (färska, frysta, konserverade, etc.)?

- En gång i veckan eller mer sällan 0 poäng
- Några gånger i veckan 1 poäng
- En gång per dag 2 poäng
- Två gånger per dag eller oftare 3 poäng

Hur ofta äter du fisk eller skaldjur som huvudrätt, i sallad eller som pålägg? *Om du är vegan/vegetarian kan du byta ut fisk och skaldjur mot vegetabiliska alternativ (exempelvis nötter, avokado, bönor/linser).*

- Några gånger i månaden eller mer sällan 0 poäng
- En gång i veckan 1 poäng
- Två gånger i veckan 2 poäng
- Tre gånger i veckan eller oftare 3 poäng

Hur ofta äter du kaffebröd, choklad/godis, chips eller läsk/saft?

- Två gånger per dag eller oftare 0 poäng
- En gång per dag 1 poäng
- Några gånger i veckan 2 poäng
- En gång i veckan eller mer sällan 3 poäng

Hur ofta äter du frukost?

- En gång i veckan eller mer sällan 0 poäng
- Några gånger i veckan 1 poäng
- Nästan varje dag 2 poäng
- Dagligen 3 poäng

<u>Ätbeteende</u>

Ibland äter vi eller låter bli att äta av anledningar som inte har att göra med hunger. Hur ofta händer det att du äter/inte äter för att du känner dig stressad, ångestfylld, uttråkad, ledsen eller uppspelt?

- Det händer mycket ofta, varje dag 0 poäng

Etikprövningsmyndigheten
2022-02920-01-290879
2022-07-07

- Det händer ofta, några gånger i veckan 1 poäng
- Det händer ibland, några gånger i månaden 2 poäng
- Det händer sällan/aldrig 3 poäng

Alkohol

Är det okej om jag frågar om dina alkoholvanor?

Alla frågor jag ställer handlar om hur det varit senaste månaden.

Kvantitet – hur ofta

Hur ofta dricker du alkohol?

- 4 gånger /veckan eller mer 0 poäng
- 2-3 gånger i veckan 1 poäng
- 2-4 gånger i månaden 2 poäng
- 1 gång/månad eller mer sällan 3 poäng

Kvantitet – hur mycket

Hur många glas dricker du en typisk dag när du dricker alkohol? *Exempel på standardglas är 12-15 cl vin, 2 x 33 cl lättöl, 50 cl folköl, 33 cl starköl, 4 cl starksprit.*

- 7 eller fler standardglas 0 poäng
- 5-6 standardglas 1 poäng
- 3-4 standardglas 2 poäng
- 1-2 standardglas eller mindre 3 poäng

Kvantitet – vid ett tillfälle

Hur ofta dricker du som är kvinna 4 standardglas eller mer och du som är man 5 standardglas eller mer vid ett och samma tillfälle? *Exempel på standardglas är 12-15 cl vin, 2 x 33 cl lättöl, 50 cl folköl, 33 cl starköl, 4 cl starksprit.*

- Dagligen eller nästa dagligen 0 poäng
- Varje vecka 1 poäng
- Varje månad 2 poäng
- Aldrig eller mer sällan än en gång i månaden 3 poäng

Etikprövningsmyndigheten
2022-02920-01-290879
2022-07-07

Fysisk aktivitet

Är det okej att jag frågar om din fysiska aktivitet?

Alla frågor handlar om hur det varit den senaste månaden.

Fysisk träning

Hur mycket tid ägnar du en vanlig vecka åt fysisk träning? Med fysisk träning menas det som får dig att bli andfådd och/eller få högre puls, exempelvis löpning, motionsgymnastik och bollsport.

- Mindre än 30 minuter (halvtimme) 0 poäng
- 30-75 minuter (ca en halvtimme till lite mer än en timme) 1 poäng
- 75-150 minuter (en till 2,5 timmar) 2 poäng
- Mer än 150 minuter (mer än 2,5 timmar) 3 poäng

Etikprövningsmyndigheten
2022-02920-01-290879
2022-07-07

Fysisk aktivitet i vardagen

Hur mycket tid ägnar du en vanlig vecka åt vardagsmotion? Med vardagsmotion menas det som gör att du får högre puls, blir andfådd eller lite varm. Exempel på det kan vara en rask promenad, cykling eller att dammsuga.

- Mindre än 30 minuter (halvtimme) 0 poäng
- 30-150 minuter (en halvtimme-2,5 timmar) 1 poäng
- 150-300 minuter (2,5-5 timmar) 2 poäng
- Mer än 300 minuter (mer än 5 timmar) 3 poäng

Stillasittande

Hur stor andel av din vakna tid sitter/ligger du stilla på en dag? I genomsnitt. *En person som sover 8h är vaken 16h.*

- Nästan hela dagen (ca 15h) 0 poäng
- 75% av dagen (ca 12h) 1 poäng
- 50% av dagen (ca 8h) 2 poäng
- 25% av dagen (4h) 3 poäng

Avbrott i stillasittande

Hur ofta avbryter du stillasittandet med rörelse? Exempelvis, städar, går på toa, går omkring en stund.

- Var fjärde timme eller mer sällan 0 poäng
- Varannan timme 1 poäng
- En gång i timmen 2 poäng
- En gång i halvtimmen 3 poäng

Sömn

Är det okej att jag frågar om din sömn?

Alla frågor handlar om hur det varit den senaste månaden.

Etikprövningsmyndigheten
2022-02920-01-290879
2022-07-07

Duration

Hur många timmar sover du vanligtvis per natt? *Om du har svårt att veta kan det hjälpa att skriva ned när du går du lägger dig, somnar och vaknar.*

- Mindre än 5h alternativt mer än 10h 0 poäng
- 5-6h alternativt 9-10h 1 poäng
- 6-7h 2 poäng
- 7-9h 3 poäng

Kontinuitet

Har du problem med något av följande: svårt att somna, att du vaknar på natten eller för tidigt på morgonen? *Här kan du dela upp frågan i tre: ”Har du svårt att somna?” ”Har du problem med att du vaknar på natten?” ”Har du problem med att du vaknar för tidigt på morgonen?” Lägg ihop problemen.*

- Ja, 4 gånger i veckan eller mer 0 poäng
- Ganska ofta, 2-3 gånger i veckan 1 poäng
- Ganska sällan, 2-4 gånger i månaden 2 poäng
- Nej, en gång i månaden eller mer sällan 3 poäng

Regelbundenhet

Går du och lägger dig och går upp ungefär samma tid varje dag? *Med samma tid menar vi här om det skiljer mer än en timme vid sänggående/uppstigning. Eventuella skillnader mellan vardag och helg ska också räknas in.*

- Nej, sover väldigt oregelbundet 0 poäng
- Ganska sällan, har sömnrutiner men frångår dem 3-4 dagar/veckan 1 poäng
- Ganska ofta, har sömnrutiner men frångår dessa 1-2 dagar/veckan 2 poäng
- Ja, jag sover väldigt regelbundet 3 poäng

Pigghet/trötthet

Känner du att du orkar med under hela dagen? *Med ”orka med” menas att man orkar genomföra dagen och de saker man planerat. Motsatsen skulle kunna vara att man ställer in saker eller gör dem slarvigt för att man är trött på grund av sömnbrist.*

- Nej 0 poäng
- Ibland, 3-4 dagar i veckan 1 poäng
- Ofta, 5-6 dagar i veckan 2 poäng
- Ja 3 poäng

Tillfredsställelse

Hur nöjd är du med din sömn på en skala från 0-3 där 0 står för ”Missnöjd” och 3 för ”Nöjd”?

- Missnöjd (önskar stor förändring) 0 poäng
- Ganska missnöjd (det finns önskemål om förändring) 1 poäng
- Ganska nöjd (trivs bra men det finns önskemål om mindre förändring) 2 poäng
- Nöjd (önskar inga/endast marginella förändringar) 3 poäng

Intressen/aktiviteter

Är det okej att jag frågar om vad du gillar att göra för aktiviteter?

Alla frågor handlar om hur det varit den senaste månaden.

Trivsamma fritidsintressen

Hur ofta ägnar dig åt något fritidsintresse (idrott/hobby/intresse) som du mår bra av? Med ”mår bra” menar vi att du blir dig glad eller lugn av den.

- Förekommer inte, alternativt mer sällan än 1 gång/månaden 0 poäng
- Ganska sällan, 2-4 gånger i månaden 1 poäng

Etikprövningsmyndigheten
2022-02920-01-290879
2022-07-07

- Ganska ofta, 2-3 gånger i veckan 2 poäng
- Ja, 4 gånger i veckan eller mer 3 poäng

Nöjdhet med aktiviteter i vardagen (inkl. delaktighet och meningsfullhet)

Hur nöjd är du med det du gör om dagarna på en skala från 0-3 där 0 står för ”missnöjd” och 3 för ”nöjd”. Du kan även räkna in jobb/sysselsättning om det bidrar med att du känner meningsfullhet.

- Missnöjd (önskar stor förändring) 0 poäng
- Ganska missnöjd (finns önskemål om förändring) 1 poäng
- Ganska nöjd (trivs men finns önskemål om små förändringar) 2 poäng
- Nöjd (önskar ingen/endast marginella förändringar) 3 poäng

Problem gällande kvantitet

Har någon annan kommenterat hur mycket tid du ägnar åt din/dina fritidsaktiviteter?

Tex skärmtid.

- Ja, omgivningen tycker att jag oftast lägger för mycket tid 0 poäng
- Ja, omgivningen tycker att jag ibland lägger för mycket tid 1 poäng
- Väldigt sällan, men det har hänt 2 poäng
- Nej, (eller bara positiva kommentarer) 3 poäng

Etikprövningsmyndigheten
2022-02920-01-290879
2022-07-07

Vänner/familj

Är det okej att jag frågar om dina sociala relationer?

Alla frågor handlar om hur det varit den senaste månaden.

Kvalitet på sociala relationer

Har du någon som du verkligen litar på?

- Nej 0 poäng
- Vet inte, kanske 1 poäng
- Tror det 2 poäng
- Ja 3 poäng

Ensamhet

Hur ofta känner du sig ensam?

- Alltid, alternativt 4 gånger i veckan eller mer 0 poäng
- Ofta, 2-3 gånger i veckan 1 poäng
- Ibland, 2-4 gånger i månaden 2 poäng
- Aldrig, alternativt mer sällan en 1 gång/månad 3 poäng

Tillfredställelse med sociala relationer

Hur nöjd är du med dina vänner/relationer på en skala från 0-3 där 0 står för ”missnöjd” och 3 för ”nöjd”? *Missnöjet kan både handla om en önskan att ha fler eller närmare relationer.*

- Missnöjd (önskar stor förändring) 0 poäng
- Ganska missnöjd (finns önskemål om förändring) 1 poäng
- Ganska nöjd (trivs men det finns önskemål om liten förändring) 2 poäng
- Nöjd (önskar inga/endast marginella förändringar) 3 poäng

Relationer utanför familjen

Hur många vänner har du utanför din familj? *Om du har vänner online räknas dessa om ni även ses fysiskt (IRL). Du avgör vem som räknas som vän.*

- Ingen 0 poäng
- En 1 poäng
- Två 2 poäng
- Tre eller fler 3 poäng

Etikprövningsmyndigheten
2022-02920-01-290879
2022-07-07

Sexuell hälsa

Sexualitet kan vara en viktig del av vårt välmående och njutning. Exempel på sex kan vara sex med sig själv, någon annan eller online. Varje enskild persons sexualitet är individuell: vi har olika mycket lust att ha sex och vi tycker om olika typer av sex.

Är det okej att jag frågar om din sexuella hälsa?

De två första frågorna gäller hur det varit det senaste året.

Njutning

Hur nöjd är du med ditt sexliv på en skala från 0-3 där 0 står för ”missnöjd” och 3 för ”nöjd”?

- Missnöjd (önskar stor förändring) 0 poäng
- Ganska missnöjd (finns önskemål om förändring) 1 poäng

- Ganska nöjd (trivs bra men det finns önskemål om liten förändring) 2 poäng
- Nöjd (önskar inga/endast marginella förändringar) 3 poäng

Smärta, sjukdom och hinder

Är sex svårt för dig: upplever du smärta, har du någon sjukdom eller något annat hinder som påverkar din möjlighet att ha sex?

- Ja, min förmåga att ha sex påverkas negativt av detta 0 poäng
- Ja, men jag är nöjd som det är ändå 3 poäng
- Nej, min förmåga att ha sex hindras inte av detta 3 poäng

Trygghet

Har du någonsin varit med om sex som på något sätt har varit jobbigt, till exempel våldsamt, kränkande eller utan samtycke? Här räknas såväl om du själv varit utsatt eller utsatt andra.

Etikprövningsmyndigheten
2022-02920-01-290879
2022-07-07

- Ja och jag har inte fått hjälp eller stöd 0 poäng
- Nej, men jag vill gärna få hjälp att känna mig mer trygg 1 poäng
- Ja, men jag har fått hjälp och behöver ingen 3 poäng
- Nej 3 poäng

Droger

Med droger menar vi både olagliga droger och preparat som har ett medicinskt syfte men som används för andra syften.

Är det okej att jag frågar om dina drogvanor?

Användande

Har du någonsin tagit en drog eller missbrukat medicinska preparat (ej alkohol) och i så fall, när använde du droger senast?

- Ja, för mindre än två veckor sedan 0 poäng
- Ja, för mindre än tre månader sedan 1 poäng

- Ja, för mindre än ett år sedan 2 poäng
- Nej, alternativt mer än ett år sedan 3 poäng

Kvantitet

Hur ofta under det senaste året har du använt droger eller missbrukat medicinska preparat?

- 2-3 gånger i veckan eller mer 0 poäng
- 2-4 gånger i månaden 0 poäng
- En gång i månaden eller mer sällan 1 poäng
- Aldrig 3 poäng

Skärmtid

Skärmtid kan vara att spela spel, använda sociala medier, att surfa runt på internet och titta på TV/film/serier. Skärmtid kan vara utvecklande och kul men ska inte gå ut över andra levnadsvanor.

Är det okej att vi frågar om din skärmtid?

Känner du igen dig i följande påståenden?

- **Att andra tycker att du har för mycket skärmtid**.
- **Att det är svårt att sätta gränser och att följa de gränser du sätter.**
- **Att du känner att din skärmtid leder till att du inte gör saker du borde göra (exempelvis sova, studera, jobba, intressen, fysisk aktivitet, träffa vänner).**
- **Att du använder skärm för att fly från verkligheten/slippa negativa känslor.**

- Känner igen mig i alla påståenden 0 poäng
- Känner igen mig i flera påståenden 1 poäng
- Känner igen mig ett påstående 2 poäng
- Känner inte igen mig i något påstående 3 poäng

Se översiktsfigur och vidare instruktioner för att tolka och rapportera resultaten på nästa sida!

Etikprövningsmyndigheten
2022-02920-01-290879
2022-07-07

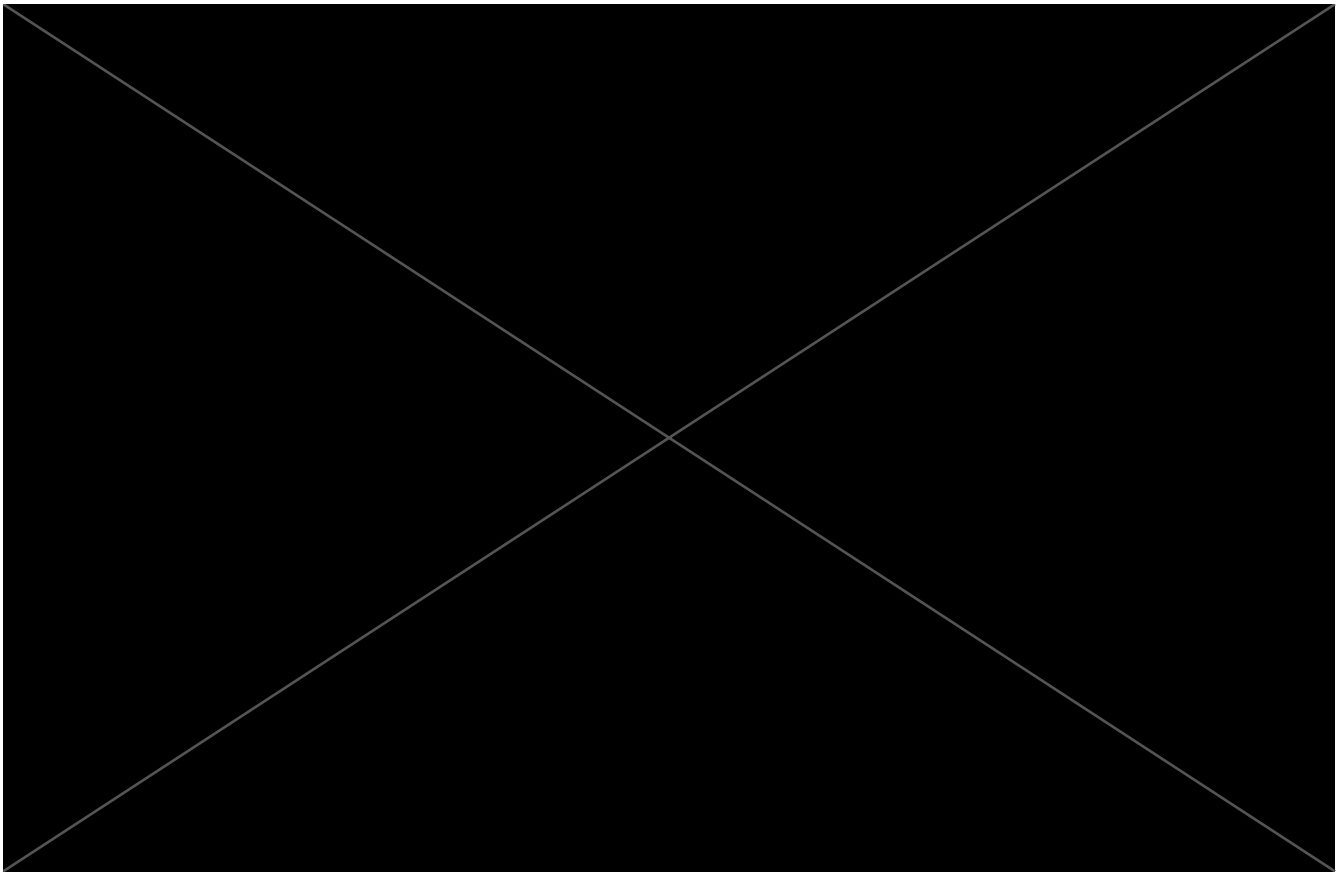

Etikprövningsmyndigheten
2022-02920-01-290879
2022-07-07

Återkoppling av resultat till deltagare

Gör en markering längst strecket för varje levnadsvana baserat på den poäng/nivå som deltagaren fick enligt Lev-s. Till din hjälp finns en Excelmall som sammanställer poängen.

Här ser du hur dina levnadsvanor ser ut just nu (peka på de små prickarna). **Levnadsvanor är inte samma sak som din personlighet. Det är vanor du har just nu. De flesta av oss har ohälsosamma levnadsvanor inom något område. Det varierar även genom livet hur väl vi lyckas prioritera det hälsosamma vanorna.**

Grönt betyder att den levnadsvanan är hälsosam.

Gult betyder att det finns risk för ohälsa. Du kan må bättre genom att ändra på dessa vanor.

Rött betyder hög risk för ohälsa. Den här levnadsvanan gör att du antagligen mår eller kommer må dåligt i framtiden. Det positiva är att du har mycket att vinna på att ta tag i röda områden.

Var resultatet som du hade förväntat dig? Vissa kan bli nedslagna av många röda fält. Upprepa att dessa poäng inte säger något om deltagarens personlighet utan att de är tillfälliga vanor. Betona att det är bra att de tagit tag i levnadsvanorna och att de kommit till rätt plats för att få stöd.

De flesta av oss behöver stöd för att ändra på våra levnadsvanor. Skulle du vilja ha vårt stöd i arbetet med att skapa hälsosammare levnadsvanor?

Journalför resultat utifrån färgskalan ovan samt om Lev ska genomföras.

Oavsett om deltagaren säger ja eller nej har du som behandlare gjort mycket genom att ta upp frågan. Många gånger blir det startskottet för en förändring lite längre fram. Tänk på att en del väljer att förändra på egen hand eller söka stöd på annat håll, medan andra kan behöva tid och kanske är

mer mottagliga vid ett annat tillfälle. Följ upp deltagare som blir nedslagna eller besvikna och förmedla kontakt till läkare eller psykolog.

Fas 1: Lyft frågan om levnadsvanor

Denna fas syftar till att informera om kopplingen mellan levnadsvanor och hälsa. Syftet är också att välja en levnadsvana, sätta mål och att behandlaren och deltagaren planerar tillsammans hur dessa mål ska nås.

Presentation

Hej jag är … Kom ihåg att möta deltagaren utifrån de förutsättningar hen har. Många vet att de borde leva hälsosammare men har svårt att göra det.

- **Jag vill övertyga dig om att bättre levnadsvanor är bland det mest effektiva vi kan göra för vår hälsa.**
- **De flesta av oss gör inte gör allt ”rätt” eller det som är mest hälsosamt. Det viktiga är man försöker.**

Etikprövningsmyndigheten
2022-02920-01-290879
2022-07-07

Tips för en lyckad dialog genom insatsen:

- Lyssna och sammanfatta vad deltagaren säger.
- Normalisera situationen och tänk på att det som deltagaren berättar inte ska leda till negativa konsekvenser eller kritik.
- Ha ett empatiskt förhållningssätt med fokus på att lyssna till det som deltagaren berättar.
- Fokusera på de levnadsvanor som tas upp inom Lev. Deltagaren kan ha ett stort behov av att ta upp andra viktiga saker men som blir för mycket att adressera inom ramen för Lev.

Introduktion till deltagarens arbetsmaterialmaterial

Logga in på godkänd digital behandlingsplattformen med deltagaren och säkerställ att han eller hon blir bekant med strukturen. Alternativt väljer ni att arbeta med utskrivet pappersmaterial.

Detta är ditt arbetsmaterial. Visa arbetsboken**. Här i finns:**

- **ditt resultat från mätningen av dina levnadsvanor**
- **korta beskrivningar av det vi går igenom**
- **dina mål (som vi sätter om en liten stund)**
- **strategier som du ska använda för att nå målen**
- **hemuppgifter**

Ta med dig arbetsboken varje gång vi ses.

Vi kommer även att ha kontakt mellan besöken så att vi kan vara ett stöd när du genomför hemuppgifter. Hur vill du att vi håller kontakten under insatsen; via digital vårdplattfrom eller telefon?

Agenda

Det här ska vi göra idag:

- **Vi ska prata om hur levnadsvanor påverkar din hälsa.**
- **Vi tittar på dina levnadsvanor (som vi har frågat om tidigare) och väljer en levnadsvana som du vill ändra på.**
- **Vi går igenom hur vi och andra kan stötta dig att nå till hälsosammare levnadsvanor.**

Låter det okej?

Definition av hälsa och levnadsvanor

Två viktiga ord jag kommer använda är ”hälsa” och ”levnadsvanor”.

Många tänker att hälsa är att må bra fysiskt (i kroppen) och psykiskt (exempelvis vara glad, lugn). Vad tänker du på när du hör ordet hälsa? Sammanfatta kort det deltagaren säger.

Etikprövningsmyndigheten
2022-02920-01-290879
2022-07-07

Levnadsvanor påverkar vår hälsa. Hur du lever varje dag, hur mycket du rör på dig och vad du äter är exempel på levnadsvanor. Alla har vi någon ohälsosam vana som påverkar hur vi mår och när vi gör något åt den mår vi bättre.

Det finns lite olika definitioner av levnadsvanor. Vad tänker du på när du hör ordet levnadsvanor?

Vad känner du till om hur levnadsvanor påverkar vår hälsa?

Sammanfatta kort det deltagaren säger.

Levnadsvanor går att ändra

Levnadsvanor är inte dina egenskaper. Det är saker du lärt dig och det går därför att ändra.

De flesta av våra levnadsvanor blir till av för att något känns bra. När vi gör något som känns bra fortsätter vi ofta att göra det.

När vi gör något ofta blir det lättare och lättare och det blir nästan som en del av oss. Ex: Om vi är vana att ta bussen känns det svårt att ta cykeln dit vi ska.

MEN, det går att ändra på vanor även om det kan vara svårt. Levnadsvanorna är saker vi lärt oss och vi kan därför lära oss nya vanor. Exempel: När vi slutat ta bussen och istället cyklat i ett par veckor kommer det kännas mycket enklare än innan.

Variera med exempel som du tror är bra för den du möter.

Andra exempel:

- Ta en frukt/nötter istället för att äta sötsaker.
- Ta trapporna istället för hissen.
- Byta ut alkohol mot träning.
- Alltid lägga sig samma tid istället för att bestämma det beroende på vad du gör.

Hur levnadsvanor påverkar hälsa

Med levnadsvanor menar vi alltså det du brukar göra. Att göra förändringar i hur du lever ditt liv kan få dig att:

- **Må bättre psykiskt – bli lugnare och gladare**
- **Må bättre fysiskt – minska risken för sjukdomar och kroppsligt obehag och göra att du orkar mer**

Kortsiktiga konsekvenser

Visa ändringar känner du direkt, exempelvis om du sover dåligt så känner du dig trött. Om du har kul med vänner känner du dig direkt gladare.

Långsiktiga konsekvenser

Andra saker märker man efter längre tid, exempelvis om du röker, dricker alkohol eller äter onyttigt känns det ofta bra i stunden men du kommer få sämre hälsa i framtiden. Det kan handla om att bli sjuk oftare, orka mindre och riskera allvarliga sjukdomar som cancer, diabetes och hjärtkärlsjukdomar.

Ett annat exempel är om du ökar din fysiska aktivitet. Det kan kännas tungt i början under själva träningspasset (på kort sikt) men efter ett tag går det lättare och du kommer må bättre och bli friskare (på lång sikt).

Etikprövningsmyndigheten
2022-02920-01-290879
2022-07-07

När det gäller dig, har du märkt att din hälsa påverkas av dina levnadsvanor?

Gör du något som känns bra på kort sikt men som du vet är sämre på lång sikt?

Sammanfatta.

Levnadsvanor som påverkar din hälsa

Vi har valt ut levnadsvanor som är viktiga för alla människor för att må bra.

Alla behöver inte exakt samma sak men alla kan göra ändringar som gör att man mår bättre.

Dessa områden är:

- **Fysisk aktivitet: Vi behöver röra på oss för att må bra.**
- **Tobak, alkohol, droger: Dessa är saker vi ska undvika.**
- **Kost: Du behöver äta varierat, regelbundet och lagom mycket.**
- **Sömn: Om du sover lagom mycket.**
- **Intressen: Vi kan må bättre av att ha roligt och göra något vi gillar.**
- **Skärmtid: Spel och sociala medier är kul men bör inte ta tid från annat (exempelvis fysisk aktivitet och att träffa vänner ansikte mot ansikte).**

- **Vänner: Här har vi olika behov men att inte känna sig ensam kan vara väldigt viktigt för hälsan.**
- **Sexuell hälsa: Även här har vi olika behov men sex kan vara en källa till njutning och det är viktigt att man känner sig trygg och bekväm med sin sexualitet.**

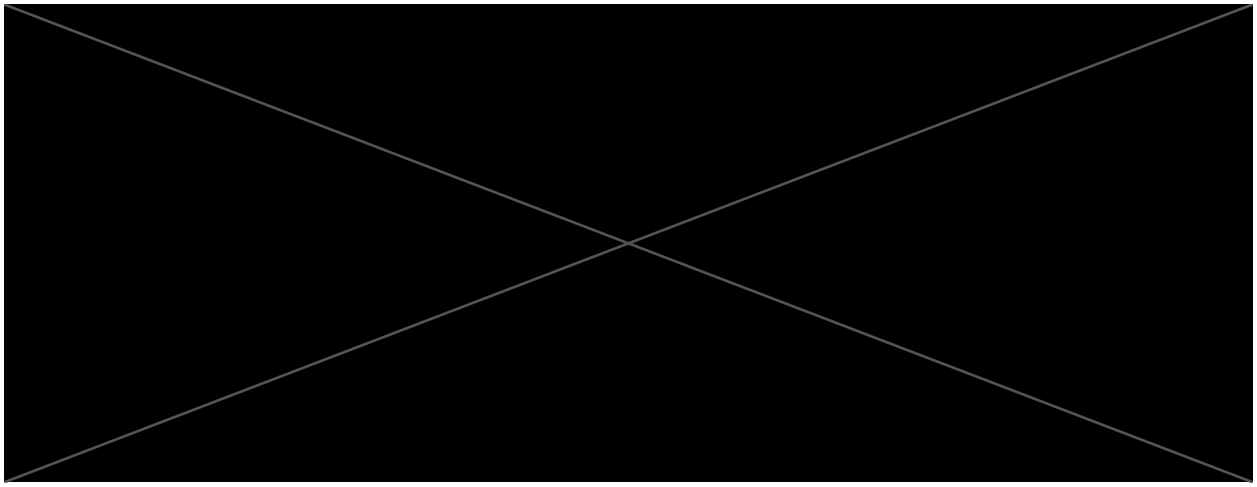

Figur 2. Kugghjul som visar hur förändring inom en levnadsvana kan leda till förändring inom en annan.

Etikprövningsmyndigheten
2022-02920-01-290879
2022-07-07

Ta fram deltagarens resultat utplacerat i översiktsfiguren i Lev-s. Om ni redan bestämt område kan ni hoppa ned till rubriken ”Målsättning/Åtgärdsplan”.

I figuren ser du hur dina levnadsvanor ser ut nu (peka på de små prickarna). **Färgerna är till för att vi ska veta om det finns saker vi kan göra för att du ska må bättre.**

Grönt betyder att den levnadsvanan är hälsosam.

Gult betyder att det finns saker du kan ändra på som kan få dig att må bättre.

Rött betyder att den här levnadsvanan gör att du antagligen mår eller kommer må dåligt. Det positiva är att du har mycket att vinna på att ta tag i röda områden.

Val av område (alternativ start)

Om du inte redan bestämt ett område så ska du göra det nu. Välj helst ett område som är rött eller gult men det är viktigt att du känner dig motiverad. I din arbetsbok finns så tips/råd om alla levnadsvanor. Visa vart dessa finns i arbetsboken. Visa motsvarande film* eller läs råden för deltagaren.

***Filmerna är inte ännu klara.**

I bilaga 1 finns exempel på hur du kan hantera vanliga motståndsargument och var man kan få vidare stöd på kvalificerad nivå. Om du behöver mer stöd för att undersöka motivationen hos deltagaren, gå till bilaga 3: ”Motivation”.

Målsättning

Om ni redan gjort en åtgärdsplan och satt upp generella mål för hälsa bör ni komplettera med individspecifika, SMARTA mål.

Om du ska remittera externt

Det kan vara svårt för dig som behandlare att veta vad som är rimliga och lämpliga mål gällande alla levnadsvanor. Om du ska remittera externt utanför organisationen kan målen därför t.ex. fokusera på att skapa den kontakten och genomföra den behandlingen. Under fas två kan du notera specifika mål som den externa vårdgivaren/organisationen satt upp med deltagaren.

Nu ska du sätta upp mål.

Lyssna på deltagaren, justera enligt SMART-principen och skriv in dem i åtgärdsplanen.

- Specifikt, tydligt och konkret.
- Mätbart i tid, kvantitet eller kvalitet.
- Accepterat, attraktivt.
- Realistiskt (om deltagaren föreslår ett orealistiskt mål: försök få hen att välja det första steget för att komma dit.
- Tidsatt, när målet ska uppnås.

Minska externa hinder

- **Vilka hinder finns i miljön omkring dig som kan göra det svårt för dig att utvecklas mot ditt mål?**
- **Vilka möjligheter finns i miljön omkring dig som kan hjälpa dig utvecklas mot ditt mål?**

Minska interna hinder

- **Alla blir frustrerade när det känns svårt. I vilka situationer finns det risk att du ger upp eller inte jobbar mot ditt mål?**
- **Vilka av dina styrkor kan hjälpa dig att nå ditt mål?**

Etikprövningsmyndigheten
2022-02920-01-290879
2022-07-07

Samverkan

Planera med deltagaren hur samverkan med andra behöver ske. Det kan exempelvis vara hjälp att få kontakt med föreningar eller stöd i genomförandet av kommunens personal. Om det gäller samverkan med annan vårdaktör finns instruktioner hur du skriver remiss för varje område i bilaga 1.

Närverk runt deltagaren

När man ska genomföra förändringar är det väldigt bra att ta hjälp av människor runt omkring.

Exempel på hur man kan be om stöd:

- **Vill du springa/ta en promenad med mig?**
- **Jag försöker äta hälsosamt, kan du hjälpa mig genom att inte bjuda mig på kakor utan frukt när vi fikar?**

Vi kan också hjälpa dig genom att prata med dina anhöriga eller någon som stöttar dig. Är det någon du vill att vi kontaktar och berättar om de tips vi gett dig? Om deltagaren vill, ring upp eller mejla de enkla råd som du gett deltagaren gällande vald levnadsvana (textform bilaga 1 eller som film).

Hemuppgift

Bra jobbat idag! Nu har vi börjat. Om cirka två veckor hörs vi via alltid öppet/telefon. Då ska du berätta vad du gjort/försökt göra för att komma närmare dina mål. Genomför de enkla råden under rubriken ”vad du kan göra själv”. Det kan givetvis vara andra saker också. Även små saker räknas! Det viktigaste är att du kommer igång, inte att det stora förändringar på kort tid.

Nedan finns ett enkelt tips för varje levnadsvana. (Läsa det tips som är för den valda levandsvanan).

- Avbryta stillasittande. (Fysisk aktivitet)
- Äta en extra grönsak i veckan. (Kost)
- Dämpa ljuset två timmar innan läggdags. (Sömn)
- Lägga mobilen i ett annat rum när du gör något annat i 5 minuter. (Skärmtid)
- Prata med en vän om att du vill minska på tobak/alkohol/droger.
- Avsätt tid då du ska göra en aktivitet som du verkligen tycker om. (Intressen)
- Titta på en av länkarna om sexuell hälsa som du fått tips om. (Sexuell hälsa)
- Beröm någon vän/familjemedlem som gör något bra/snällt. (Sociala relationer/vänner)

Jag hör av mig genom stöd- och behandlingsplattformen för en kortare avstämning om cirka 2 veckor för att se hur du kommit i gång.

Om deltagaren inte kommit igång så bokar du in fas 2 så snart som möjligt. Annars kan du låta det gå ca veckor 4 så att deltagaren hinner jobba lite själv.

Etikprövningsmyndigheten
2022-02920-01-290879
2022-07-07

Journalskrivning och efterarbete

- Notera vald målsättning om det inte redan är gjort
- Vidare remiss: Se bilaga 1 om viktiga saker att inkludera till den du remitterar till.
- Kontakta nätverk kring deltagaren om hen önskat det.
- Stäm av med deltagaren om 2 veckor och ge positiv förstärkning av genomförda beteendeförändringar eller försök.
- Förbered nästa möte genom att titta på fas 2 i manualen. Boka in det så snart som möjligt som du ser att deltagaren inte kommit igång.

Bra jobbat! ☺

Slut fas 1

Fas 2: Stöd i genomförande

Denna fas kan ges flera gånger och syftar till att undanröja hinder och öka motivationen hos deltagaren. Läs alltid igenom instruktionerna och förbered innan mötet med deltagaren.

Lyssna

Hur har det gått sedan med dina levnadsvanor sedan vi pratade sist? Ta dig tid att verkligen lyssna den du pratar med. Avgränsa samtalet till levnadsvanor.

Uppdatering av målsättning

Kommer du ihåg vad vi satte för mål förra gången vi sågs? Om deltagaren träffat annan vårdgivare som ett led i att nå målen så kan du uppdatera, om nya mål eller förändring av målsättning skett. Fråga deltagaren.

Fokusera på positiva beteendeförändringar

Fokusera på det som deltagaren klarat av för att stärka tillit till den egna förmågan. Om de inte ännu klarat av något, beröm att de har försökt. Tänk på att små saker räknas!

Om deltagaren har nått alla målen kan ni jobba med att förstå varför det gått bra och även jobba med vidmakthållande.

Agenda

Idag kommer vi först prata om hur det kommer sig att vi lär oss vissa levnadsvanor. Då kommer vi fokusera på vad du kan ändra för att komma närmare dina mål.

Efter det kommer vi också prata om vad det finns omkring dig som hindrat dig. Saker som du har svårt att påverka men som vi tillsammans kanske kan hitta någon form av lösning på.

Är det okej att vi pratar om hur våra levnadsvanor blir till?

Etikprövningsmyndigheten
2022-02920-01-290879
2022-07-07

Hur belöning styr våra levnadsvanor

Belöning

Hur dina levnadsvanor ser ut är inte en del av din personlighet. Levnadsvanor är saker vi lärt oss och vi kan därför lära oss nya.

De flesta av våra levnadsvanor blir till av för att något känns bra. När vi gör något som känns bra fortsätter vi ofta att göra det.

Det kan vara hälsosamma saker som att äta, umgås med vänner eller utöva intressen. Det kan också vara att få beröm från andra när vi gjort något bra. Chansen är stor att vi fortsätter med det som känns bra i stunden.

Har du något exempel på levnadsvanor som du känner en belöning av att göra?

Att lära sig prioritera det långsiktiga framför det kortsiktiga

Tyvärr känner vi välbehag/belöning även i situationer som inte är bra för oss på längre sikt. Det kan vara ohälsosamma vanor som att röka, dricka alkohol, för mycket skärmtid, stanna uppe och kolla på film när vi borde sova eller äta för mycket onyttig mat. De flesta av oss tycker att det är svårt att inte välja det som känns bra i stunden.

Har du några exempel på när du väljer det som känns bra (är belönande) i stunden men som inte är så hälsosamt på längre sikt? Led in på levnadsvanan som deltagaren jobbar med.

Belöning genom att undvika

En annan likande situation är när vi gör något för att undvika att ha tråkigt eller känna obehag/ångest. Om vi är rökare vill vi ibland ta en cigarett för att slippa abstinens/ångest/att ha tråkigt. På samma sätt kan vi dricka alkohol, shoppa, spela eller äta för att slippa ha tråkigt eller undvika obehag.

Har du något exempel på när du gör något för att undvika att ha tråkigt? Led in på levnadsvanan som deltagaren jobbar med.

Funktionell analys

Nu ska vi titta på de saker som inte fungerat och på de saker som fungerat gällande dina hemuppgifter. Börja med beteendet. Fråga sedan var dom hände precis innan för att få grepp om situationen. Du väljer själv om du vill börja med oönskade eller önskade beteenden men kom ihåg att det många gånger är bra att avsluta med något som fungerat för att bygga tillit till den egna förmågan.

Du kan förbereda dig innan mötet med exempel från bilaga 2. Tre viktiga aspekter av denna övning är att 1) förstärka vikten av långsiktiga konsekvenser 2) att identifiera ”riskfyllda” situationer där man frestas till att fatta ohälsosamma beslut 3) underlätta önskade beteenden.

Etikprövningsmyndigheten
2022-02920-01-290879
2022-07-07

Oönskat beteende

Situation/stimuli	Beteende	Kortsiktig konsekvens	Långsiktig konsekvens
**Strategier:**			

Önskat beteende

Situation/stimuli	Beteende	Kortsiktig konsekvens	Långsiktig konsekvens
**Strategier:**			

Beteendestödsplan

Situationer

Vi ska nu titta närmare på hur du kan undvika att hamna i för många situationer där du frestas fatta ohälsosamma beslut. Om du vill kan du förbereda innan mötet genom att titta på exempel på strategier i bilaga 2.

Vi fokuserar först på de situationer som var utmanande och försöker komma på vad du kan göra för att inte hamna där lika ofta. Skriv in strategierna i arbetsboken.

Lyft fram den strategi som deltagaren använde för att möjliggöra det önskade beteenden så att den strategin kan användas igen.

Beteenden

Nu ska vi skriva ned saker som gör det enklare att välja det önskade beteendet. Utgå ifrån kolumnen ”beteende” under önskat beteende. Diskutera vad som kan vara saker som underlättar och möjliggöra önskade beteenden. Se exempel i bilaga 2. Skriv in strategierna i arbetsboken.

Är det något annat runt omkring dig som har hindrat dig från att komma närmare ditt mål?

Vad eller vilka i din omgivning har hjälpt dig komma närmare dina mål? Det är ofta en bra idé att genomföra förändringar med hjälp av andra. Skriv in strategier i arbetsboken.

Färdighetsbrister

- Fundera på om det finns hinder i form av färdighetsbrister hos deltagaren (t.ex. problem med uppmärksamhet, minne) och hur du skulle kunna möjliggöra nödvändig inlärning.
- Förenkla/gör delmålen enklare så att deltagaren klarar av att komma vidare och bygga självförtroende.
- Öva på att hantera frustration exempelvis genom avslappningstekniker
- Överväg att öva med deltagaren i den miljö delmålen ska uppnås.

Etikprövningsmyndigheten
2022-02920-01-290879
2022-07-07

Avslut

Kom ihåg att väcka hopp! **Bra jobbat idag!** Konkretisera, vad gjorde deltagaren som var bra**. Nu vet vi ännu mer om hur vi kan förbättra …** (aktuell levnadsvana)**. Vi vet att det kan komma motgångar och vi vet att det finns sätt att hantera dessa. Vi vet att du har styrkor som kan hjälpa dig som till exempel…** Beröm deltagaren för de konkreta förändringar som skett.

Du får nu i uppgift att skriv in i arbetsboken när du använder de strategier vi listat. Skriv även exempel på när du lyckas stå emot kortsiktiga belöningar (de ni listat i arbetsboken) **för att i stället få de långsiktiga. Jag checkar in och kollar hur det går om cirka två veckor.**

Beröm deltagaren för de konkreta förändringar som skett. Berätta för deltagaren att ni nästa möte (fas 3) kommer mäta alla levnadsvanor (Lev) igen och välja ett nytt område som hen kan jobba med själv eller med ditt stöd.

Om deltagarens motivation bedöms vara för låg för att ta tag i sina levnadsvanor, bedöm om annan insats är lämplig eller om kontakt behövs med annan del av vården. Försök att se det som ni gjort hittills som en start och att det kanske kommer gå att tag i levnadsvanorna i framtiden. Förmedla kontakt till läkare eller psykolog om måendet bedöms vara dåligt.

Journalskrivning och efterarbete

- Samverkan: Skriv eventuell remiss till annan vårdgivare.
- Kontakta nätverk kring deltagaren om hen önskat det.
- Återkoppla/motivera om två veckor.

- Besluta om Fas 2 ska ges ännu en gång eller när ni ska boka in fas 3. Fas 2 kan ges igen om deltagaren inte kommit närmare målet. Fas 3 ges när målet är nått eller om du vill avbryta insatsen och fokusera på något annat.
- Boka in förberedelse av nästa möte genom att titta på fas 2 eller 3 i manualen.

  Bra jobbat! ☺

Slut fas 2

Etikprövningsmyndigheten
2022-02920-01-290879
2022-07-07

Fas 3 – Uppföljning och vidmakthållande

Denna fas syftar till att följa upp målsättning och att påbörja arbete med nya vanor på egen hand.

Lyssna

Hur har det gått sedan sist med dina levnadsvanor?

Målsättning (Uppföljning)

Gå till arbetsboken.

Nu ska vi kolla hur mycket närmare dina mål du kommit. Läs upp målen och stäm av med deltagaren. Fokusera på det som deltagaren klarat av för att stärka tillit till den egna förmågan. Använd åtgärdsplanen i fyllde i vid tillfälle 1.

Eftermätning med ”Lev-s”

Genomför hela Lev-s. Om det skett förbättringar på andra områden lyfter du fram dessa och berömmer deltagaren för det. Om det skett försämring på något område kan du föreslå att deltagaren tar tag i detta område.

Val av nytt område

Du vet nu hur du ska jobba med den levnadsvana som du valt hittills. Det kan du fortsätta med när du nu ska välja en ny levnadsvana. Om du senare vill jobba med fler levnadsvanor kan du själv välja det. Allt material finns i din arbetsbok.

Alla levnadsvanor kan påverka din psykiska och fysiska hälsa, så det finns stora fördelar att fortsätta med hälsosamma levnadsvanor. Är det någon levnadsvana du funderat över? Titta tillsammans på resultatet av eftermätningen.

När deltagaren inte vill

Bedöm om någon annan insats är nödvändig. Var även uppmärksam på om låg motivation även kan innebära att deltagaren behöver stöd eller behandling för sitt psykiska mående.

Vill deltagaren inte prata vidare om någon speciell levnadsvana trots problem så tänk att du gjort vad du kunnat och notera detta i journalen. Lyft frågan om levnadsvanor vid ett senare tillfälle.

Etikprövningsmyndigheten
2022-02920-01-290879
2022-07-07

Enkla råd och hopp om vald levnadsvana

I texterna/filmerna i arbetsboken hittar du även råd gällande andra levnadsvanor. Du kan även kontakta oss om du behöver hjälp. Precis som innan stämmer vi av om cirka två veckor för att se om du kommit i gång. Om du vill kommer jag alltså kunna fortsätta att ge dig återkoppling på dina hemuppgifter/övningar men på distans. Vill du det?

Målsättning (ny)

Innan du börjar jobba själv tänkte jag att vi skulle sätta mål för den nya levnadsvanan. Använd mallen för åtgärdsplan Lev.

Formulera SMARTA mål:

- Specifikt, tydligt och konkret.
- Mätbart i tid, kvantitet eller kvalitet.
- Accepterat, attraktivt.
- Realistiskt (om deltagaren föreslår ett orealistiskt mål: försök få hen att välja det första steget för att komma dit).
- Tidsatt, när målet ska uppnås.

Minska externa hinder

- **Vilka hinder finns i miljön omkring dig som kan göra det svårt för dig att utvecklas mot ditt mål?**
- **Vilka möjligheter finns i miljön omkring dig som kan hjälpa dig utvecklas mot ditt mål?**

Minska interna hinder

- **Alla blir frustrerade när det känns svårt. I vilka situationer finns det risk att du ger upp eller inte jobbar mot dit mål?**
- **Vilka av dina styrkor kan hjälpa dig att nå ditt mål?**

Närverk runt deltagaren

När man ska genomföra förändringar är det väldigt bra att ta hjälp av människor runt omkring.

Be om hjälp och berätta för andra vilka dina mål är.

Avslut

Bra jobbat!

Jag hör av mig för en kortare avstämning om cirka två veckor för att se hur du kommit igång. Även små saker räknas!

Etikprövningsmyndigheten
2022-02920-01-290879
2022-07-07

Journalskrivning och efterarbete

- Vidare remiss till annan vårdaktör
- Utvärdera åtgärdsplanen
- Kontakta nätverk kring deltagaren om deltagaren önskat det.
- Följ upp om två veckor. Bestäm då avstämning om några månader där du kollar hur det går.
- Kom ihåg att du bör fortsätta att följa upp deltagarens levnadsvanor så att du främjat ett aktiv och hälsosamt liv på sikt!
- Upprätta plan för vidmakthållande.

Slut fas 3

Enkla råd – bilaga 1

Att genomföra Lev motsvarar att ge ”enkla råd” när man genomför fas 1 inkl. uppföljning av hemuppgift. När man dessutom genomför fas 2 och 3 motsvarar Lev ”rådgivande samtal”.

Råden i bilaga 1 är sådana enkla råd som du kan ge muntligen och som är del av deltagarens arbetsbok. Enkla råd (*Rekommendation*, *Varför det är viktigt*, *Exempel på vad du kan göra”)* finns även som filmer*. Det är ofta enklare att visa filmerna än att behöva läsa upp texten.

I bilaga 1 finns även tips på hur du kan förbereda motståndsargument. Under rubriken ”Insatser och stöd” står det förslag på insatser på rådgivande och kvalificerat rådgivande nivå samt till aktörer utan för vården som kan vara ett stöd**. Att få till en bra samverkan och att stötta i upprättandet av en kontakt vid hänvisning är en av de viktigaste principerna i Lev. Observera att det inte alltid finns insats på kvalificerat rådgivande nivå och att det innebär att du som behandlare då bör ta ett större ansvar.

*Filmerna är under bearbetning.

**Varje vårdorganisation som implementerar Lev måste noggrant gå igenom hur, när och till vem man ska vända sig för kvalificerat rådgivande samtal samt annat stöd utanför sjukvården. Denna samverkan är efterfrågad av representanter för patientorganisationerna samt i Nationellt vårdprogram, 2022.

Tobak

Rekommendation

Den allmänna rekommendationen är att avstå allt tobaksbruk. För dig som ska opereras eller är gravid är det extra viktigt.

Varför det är viktigt

Fördelarna med att minska/sluta med rökning.

- Även om du rökt länge så börjar din kropp att läka snabbt efter att du slutat.
- Redan inom några veckor förbättras till exempel blodcirkulation, lungfunktion och sårläkning.
- Mellan en månad och ett år efter rökstopp förbättras lungorna successivt och risken för hjärtinfarkt halveras.
- Sömnen blir bättre på sikt.
- Ju längre tid som går desto fler hälsofördelar:
    - Risken för stroke är halverad inom fem år.
    - Inom tio år är risken för flera cancerformer halverad.
    - Rökhostan försvinner.
    - Blodtryck och puls sjunker.
    - Risken för tandlossning minskar.
    - Skelettet blir starkare.

Fördelar med att sluta snusa:

- Du minskar risken för flera cancerformer.
- Du minskar risken för diabetes typ 2 (gäller snusare som använder minst fem snusdosor i veckan).
- Om du är gravid ökar du chansen att ditt barn överlever och blir starkt. Bland annat ökar snusning risken för havandeskapsförgiftning, låg födelsevikt, för tidig förlossning, missfall eller att fostret dör i magen.

Exempel på vad du kan göra

- Fundera på om du vill avbryta all tobaksanvändning direkt eller om du vill minska mer långsamt.
- Bestäm ett datum för när du ska sluta.
- Skriv ner alla fördelar med att sluta på en lista. Det kan hjälpa dig att bli motiverad.
- Berätta för andra om vilka dina skäl för att sluta är.
- Börja med att inte använda tobak på vissa platser. Håll upp på de platser där du tillbringar mest tid.
- Skaffa nikotinläkemedel eller något av de receptbelagda läkemedlen mot rökning.

Etikprövningsmyndigheten
2022-02920-01-290879
2022-07-07

- Sluta på din stoppdag.
- Ät regelbundet och drick vatten ofta.
- Sysselsätt dig och håll dig fysiskt aktiv.
- Belöna dig och ta fasta på att det du gör är det bästa du kan göra för din hälsa.
- Be om stöd från familj och vänner.
- Håll ut! Det kan kännas motigt ett tag, men fundera på vilka vinster ditt stopp inneburit hittills och varför det är viktigt för dig att fortsätta vara tobaksfri.
- Ha kontakt med sluta-röka-linjen. Alla samtal är gratis och du kan vara anonym. Många som tar hjälp lyckas göras en förändring och mår bättre.

Vanliga motståndsytteranden och exempel på hur man hanterar dem

Deltagaren: - Jag har försökt men det gick inte/är svårt

- Det gick inte just då men du var jättemodig/stark som provade. Vad lärde du dig av det? Hur många dagar/timmar fungerade det och vad gjorde du då?

Deltagaren: - Jag ska sluta men inte just nu…

- Just nu känns det kanske svårt samtidigt som du vill sluta. Vad skulle behöva hända för att du ska ta ett beslut att sluta? Det finns hjälp att få när du tar beslutet.

Andra användbara MI-fraser:

- Det är vanligt att man får pröva flera gånger innan man hittar ett sätt som passar.
- Du är envis och ger inte upp.
- Du har gjort svåra förändringar tidigare såsom…
- Du kände dig stressad när jag tog upp frågan. Berätta, hur går dina tankar?
- Du tycker om att röka men samtidigt ställer det till det för dig på olika sätt, t.ex. hostan på natten, det är dyrt, lukten…
- Hur kan jag stödja dig bäst i det här?
- Du har verkligen ansträngt dig för att sluta/minska
- Om motivation saknas: Finns det något som är mindre bra med att du röker/snusar?
- Om du skulle vilja testa igen i framtiden, hur skulle du göra då i stället?

Om deltagaren inte är tillräckligt motiverad att sluta med tobak: Byt fokus till en annan levnadsvana. Lyft frågan i framtiden igen.

- Du vill få till en förändring och bli rökfri på sikt. Just nu låter det som att du är mer motiverad att ta tag i något annat (till exempel fysisk aktivitet, kost…). Säg till när du vill ta upp frågan om tobak igen så finns vi här som stöd.

Oavsett hur långt ni kommer har du som behandlare gjort mycket genom att ta upp frågan. Många gånger blir det startskottet för en förändring lite längre fram. Tänk på att en del väljer att förändra på egen hand eller söka stöd på annat håll, medan andra kan behöva tid och kanske är mer mottagliga vid ett annat tillfälle.

Etikprövningsmyndigheten
2022-02920-01-290879
2022-07-07

Insatser och stöd

Alla som vill sluta eller minska på tobak kan remitteras till sluta-röka-linjen. Följ upp deltagaren under tiden och stötta kontakten och arbetet med sluta-röka-linjen.

**Aktör**	**Typ av insats**
Sluta-röka-linjen (SRL)	Kvalificerat rådgivande samtal
Husläkarmottagning/ vårdcentral	Kvalificerat rådgivande samtal

Hur du hänvisar vidare

Remiss via TakeCare:

Notera diagnos/funktionsnedsättning och behov av anpassning samt stöd i kontakten.

Ytterligare information för eget arbete

**1177 Vårdguiden Hjälp och stöd för den som vill sluta röka eller snusa**
https://www.1177.se/Stockholm/Tema/Halsa/Alkohol-och-tobak/Hjalp-att-sluta-roka/

Kost

Rekommendation

Ät mer

- frukt och grönt
- fisk och skaldjur
- nötter och frön

Byt till

- kolhydrater gjorda på fullkorn
- nyttiga matfetter (rapsolja)

Ät mindre

- kött
- salt
- socker

Varför det är viktigt

- Mat är en källa till njutning. Det går utmärkt att njuta av hälsosam mat.
- Om du äter nyttigare kan du minska risken för:
  - Stroke
  - Hjärtinfarkt
  - Skador på njurar
  - Demens
- Om du har övervikt kan viktminskning minska risken för:

Etikprövningsmyndigheten
2022-02920-01-290879
2022-07-07

    - Cancer
    - Diabetes typ 2
    - Hjärt- och kärlsjukdomar
- Att äta hälsosammare kan påverka din energinivå och ditt humör.
- Om du har problem med ätstörningar, kan du få hjälp.
    - Även om du bara har små problem med mat kan det vara en god idé att prata med en expert för att undvika att få större problem som är svårare att bryta.

Exempel på vad du kan göra själv

- Ät på regelbundna tider.
- Eftersträva att välja matvaror med det gröna nyckelhålet när du handlar.
- Ta för vana att dricka vatten till måltider och när du är törstig.
- Ät inte för att du är uttråkad, ångestfylld, stressad, trött eller uppspelt/glad. Håll dina rutiner för måltider, så är det enklare. Hoppa inte över måltider.
- Om du har problem med ätstörningar, jobbiga tankar kring mat och ätande, bör du ta hjälp.

Etikprövningsmyndigheten
2022-02920-01-290879
2022-07-07

Vanliga motståndsyttranden och hur man hanterar dem

Deltagaren: - Ja, ja men det är inte så lätt/ det är jobbigt/jag kan inte

- Du som många andra tycker att det är svårt. När valde du senast något som var nyttigt? Hur tänkte du då? Det är ett stort framsteg att minska och inte ta bort ohälsosam mat helt och hållet. Vad säger du om det? Hur skulle det vara för dig?
- Vill du att vi pratar om hur du kan göra det enklare att äta mer hälsosamt?
    - Gör en sak i taget. Exempelvis kan du börja med att minska lite på saltet.
    - Gör det tillsammans med någon annan.
    - Be om hjälp när du inte orkar fatta bra beslut själv.
    - Sätt upp enklare delmål, t.ex:
        - Äta en extra grönsak per dag
        - Äta enligt tallriksmodellen 1 dag i veckan (sedan öka till 2, 3 osv).

Deltagaren: - Jag har ingen motivation

- Du upplever att din motivation är låg. Samtidigt ser jag en vilja till förändring i och med att du är här.
- Vill du att vi pratar om hur du kan få mer motivation?
    - Hitta ditt ”varför”: Bli piggare, hälsosam vikt, bli friskare, leva längre.
    - Fokusera på känslan efter att du gjort ett bra val, hur det känns att vara stolt över sig själv.
    - Målbild: Även om det känns svårt nu; fundera på det som fungerat förut, som du är stolt över.
    - För matdagbok. Beröm dig själv när du äter hälsosamt.
    - Be andra berömma dig när du äter hälsosamt.
    - Hitta en förebild bland någon du känner som du kan inspireras av.

Deltagaren: - Jag vet hur men jag får det ändå inte gjort

- Det är en klok insikt och det gäller de allra flesta människor. Vill du att vi pratar om hur det skulle vara lättare att ”få det gjort”?
  - Gör det enkelt:
    - Börja med en nivå du klarar. Utforska vad det är för deltagaren.
    - Välj enkla, billiga och nyttiga recept som du tycker om.
    - Fyll kylen och skafferiet med hälsosamma alternativ som du gillar.
    - Undvik att ha onyttiga matvaror hemma.
  - Rutiner, exempel:
    - Varje måndag äter jag bara vegetariskt.
    - Koppla nya rutiner till andra etablerade rutiner.

  - Gör det tillsammans med någon:
    - Berätta för din omgivning att du börjat med nya matvanor och hur de kan hjälpa dig
      - Be dem innan att helst inte erbjuda sötsaker när ni fikar.

Deltagaren: - Det är tråkigt

- Vill du att vi pratar om hur vi kan göra det roligare?
  - Finns det något med matlagning du tycker är roligt? Hitta något litet som deltagaren gillar och bygg utifrån det.
  - Testa mat från olika länder
  - Gör upp med vänner/familj att ni delar upp dagar när ni lagar mat till varandra. Då slipper du laga mat själv varje dag och du får även träffa andra.
  - Göra storkok och frys in. Då behöver du inte laga mat lika ofta.
  - Det finns färdiglagad mat som är hälsosam.
  - Måste det vara kul? Om du hittar rutiner som får dig att äta hälsosammare kanske det är värt besväret trots att det är tråkigt?

Deltagaren: - Det är för dyrt

- Det stämmer att det kan vara dyrt men det måste inte vara det. Vill du att vi pratar om hur vi kan hitta billiga och hälsosamma alternativ?
  - Storkok
  - Byt ut kött mot bönor och linser. Det är även bättre för dig och miljön.
  - Ta hjälp när du storhandlar. Planera vad du ska köpa.
  - Ät oftast egen hemlagad och inte på restaurang eller hemkört.

Deltagaren: - Det är inte gott

- Vill du att vi pratar om hur du kan hitta hälsosam mat som du gillar?
  - Utforska vilka rätter du äter ofta och hur de kan göras lite nyttigare.
  - Finns det rätter som är nyttiga som du gillar?
  - Titta på nya recept.
  - Visa bilder på mat/ kolla recept med deltagaren.

Etikprövningsmyndigheten
2022-02920-01-290879
2022-07-07

Insatser och stöd

Kostvanor styrs av många olika faktorer och beroende på problematik kan olika stöd på kvalificerat rådgivande nivå behövas av Habilitering & Hälsa och/eller andra aktörer. Se till att det stöd du hänvisar till är relevant för deltagaren du träffar.

**Aktör**	**Typ av insats**
Habilitering & Hälsa	Se habiliteringsprogram
Dietist inom Primärvårdsrehabiliterin g	Kvalificerat rådgivande samtal och specifika kostråd.
Husläkarmottagning (vårdcentral)	Rådgivande samtal, till exempel hos sjuksköterska eller distriktssköterska
Vårdcentral, psykiatrisk mottagning eller ätstörningsmottagning	Behandling av ätstörning

Hur du hänvisar vidare

Remiss via TakeCare:

Notera diagnos/funktionsnedsättning och behov av anpassning samt stöd i kontakten.

Ytterligare information för eget arbete

Förslag till övrigt material som kan vara till hjälp i dialogen med deltagaren.

H&Hs eget material
**1177 Vårdguiden Information om bra matvanor**<br>Äta för att må bra - 1177 Vårdguiden<br>1177 Vårdguiden Podd om matvanor<br>Ta hand om dig! - avsnitt 4: Matvanor - 1177 Vårdguiden<br><br>Foldern ”Bra mat och rörelse” med och utan bildstöd:<br>Bra mat och rörelse - folder.pdf | Vårdgivarguiden (vardgivarguiden.se)<br>Bra mat och rörelse_bildtabeller.pdf | Vårdgivarguiden (vardgivarguiden.se)<br>De svenska kostråden från Livsmedelsverket. Finns på svenska och lättläst svenska:<br>Kostråden - Hitta ditt sätt
https://www.1177.se/Stockholm/sjukdomar--besvar/psykiska-sjukdomar-och-besvar/atstorningar/atstorningar/

Etikprövningsmyndigheten
2022-02920-01-290879
2022-07-07

Alkohol

Rekommendation

Hur mycket man kan dricka är olika från person till person.

Riskbruk för män
Veckokonsumtion: Fler än 14 standardglas/ vecka
Intensivkonsumtion: 5 eller fler standardglas vid samma tillfälle

Riskbruk för kvinnor
Veckokonsumtion: Fler än 9 standardglas/ vecka
Intensivkonsumtion: 4 eller fler standardglas vid samma tillfälle

Exempel på ett standardglas är
– 12–15 cl vin
– 2 x 33 cl lättöl
– 50 cl folköl
– 33 cl starköl
– 4 cl starksprit

Gränserna kan vara lägre om du:

- har något hälsoproblem som kan påverkas av alkohol
- tar någon medicin
- mår psykiskt dåligt
- har alkoholproblem i släkten
- är äldre eller yngre.

Etikprövningsmyndigheten
2022-02920-01-290879
2022-07-07

Varför det är viktigt

Om du minskar på alkohol kan du:

- Minska risken för över 60 sjukdomar.
- Leva längre.
- Det är en vanlig missuppfattning att små mängder alkohol varje dag skulle vara ofarligt och till och med hälsosamt. Så är det inte.
- Minska risken för cancer.
- Få bättre omdöme, tankeförmåga, minne och reaktionsförmåga. Gäller även dig som inte dricker så mycket.
- Sova bättre.
- Du som dricker mycket och lyckas minska kan minska risken för epileptiska anfall, demens och andra hjärnsjukdomar.
- Förbättra din lever, hjärtat, skelettet, huden och magen.
- Minska risken för att drabbas av infektioner.
- Minska risken för ångest och depression.
- Minska risken för olyckor.
- Det är lätt att dricka mer och oftare än vad kroppen klarar av.

Av de saker vi har gått igenom, vilka skulle vara aktuella för dig tror du?

Exempel på vad du kan göra

- Drick inte alkohol för att släcka törsten.
- Drick vartannat glas alkoholfritt.
- Börja med en helt nykter period. Efter en sådan kan det vara lättare att förändra vanor.
- Sätt upp ett mål. Bestäm i förväg hur mycket du får dricka per tillfälle eller vecka.
- Skriv ner vad du dricker och när du dricker. Då får du bättre överblick och kontroll. Dina framsteg blir tydligare.
- Tänk igenom vilka situationer eller vilket umgänge som innebär en risk för att du dricker för mycket. Kan du ändra något?
- Tänk igenom vilka alternativ du har till att dricka alkohol vid de tillfällen då det kan vara lockande. Ha en plan, var förberedd. Kan du välja en annan dryck? Hitta andra lösningar?
- Ta hjälp från dina närstående. Berätta att du vill dricka mindre eller inte alls. Säg att du skulle uppskatta deras stöd.
- Det är olika vad som fungerar bäst. Prova vad som fungerar för dig.
- Du har möjlighet att få behandling på vårdcentral eller beroendemottagning för att förändra dina alkoholvanor.
- Vi kan hjälper dig att upprätta kontakt och stöttar dig på vägen.
- Lär dig att säga ’nej tack’ när du blir bjuden på alkohol:
    - Tack, men inte just nu eller inte ännu. Jag har bestämt att jag dricker mindre i dag.
    - Tack, men inte just nu för jag är redan berusad.
    - Nej tack, jag har en jobbig dag i morgon.
    - Nej tack, annars sover jag inte ordentligt.
    - Nej tack, jag vill vara pigg i morgon.
    - Tack, men inte just nu eller inte ännu. Jag har beslutat att skära ner på alkohol.

Källa: 1177 Vårdguiden Liv & Hälsa

Etikprövningsmyndigheten
2022-02920-01-290879
2022-07-07

Vanliga motståndsyttranden och hur man hanterar dem

Tänk på att det som deltagaren berättar inte ska leda till kritik eller negativa konsekvenser.

Deltagaren: - Ja, men det är inte så lätt…

- Det stämmer att göra en förändring brukar vara svårt samtidigt som alkohol innebär negativa konsekvenser för dig. Kan vi titta lite närmare på det?

- Det är inte så lätt samtidigt som alkohol har orsakat svårigheter för din hälsa (konkretisera inom vilka områden) och dina relationer (konkretisera hur). På vilket sätt skulle det vara viktigt för dig att ha en bättre hälsa och bättre relationer?

Deltagaren: - Jag kan koppla av, känna mer/mindre med alkohol

- Jag förstår att det kan kännas skönt och att du har det behovet. Finns det något annat som kan få dig att känna på likande sätt som är mindre skadligt än alkohol?

Deltagaren: - Jag ska sluta men inte just nu…

- Just nu känns det kanske svårt samtidigt som du vill sluta. Vad skulle behöva hända för att du ska ta ett beslut att sluta? Det finns hjälp att få när du tar beslutet.

Deltagaren: - Jag har försökt innan men det gick inte/är svårt

- Det gick inte just då, samtidigt var du jättemodig/stark som provade. Vad lärde du dig av det? Hur många dagar/timmar fungerade det och vad gjorde du då?

Deltagaren: - Det är svårt att inte vara den som dricker när andra gör det.

- Alkohol är väldigt vanligt och vi är många som känner att det förväntas av oss att dricka. Har du testat att säga till dina vänner eller din familj att du skulle vilja umgås utan att dricka?
- Träna på att säga nej tack:
  - Tack, men jag har bestämt att jag dricker mindre i dag.
  - Tack, men inte just nu, jag är redan berusad.
  - Nej tack, jag har en jobbig dag i morgon.
  - Nej tack, annars sover jag inte ordentligt.
  - Nej tack, jag vill vara pigg i morgon.
  - Tack, men jag har beslutat att skära ner på mitt drickande.

Etikprövningsmyndigheten
2022-02920-01-290879
2022-07-07

Deltagaren: - Andra användbara MI-fraser:

- Det är vanligt att man får pröva flera gånger innan man hittar ett sätt som passar.
- Du är envis och ger inte upp.
- Du har gjort svåra förändringar tidigare såsom…

Insatser och stöd

Om Lev redan testats eller inte bedöms fungera, välj då åtgärd på kvalificerat rådgivande nivå enligt nedan. Det finns också läkemedel som minskar alkoholsug, och som kan användas som komplement till rådgivande eller kvalificerat rådgivande samtal på beroendemottagning eller husläkarmottagning.

**Aktör**	**Typ av insats**
Beroendemottagningar, se 1177	Kvalificerad rådgivande nivå
Husläkarmottagningar (vårdcentraler):	Rådgivande eller kvalificerat rådgivande

Hur du hänvisar vidare

Remiss via TakeCare:

Notera diagnos/funktionsnedsättning och behov av anpassning samt stöd i kontakten.

Detta föreslår beroendecentrum Stockholm att remiss via TakeCare även kan innehålla för att förbättra kontakten.

- Aktuellt substansbruk samt omfattning.
- Görs några andra insatser som kan stötta/förebygga missbruket?
- Andra pågående insatser (exempelvis via kommunen eller psykiatrin).
- Aktuell social situation (boende, arbete, ekonomi).
- Finns suicidalitet i anamnesen?
- Våldshistorik?
- Minderåriga barn?
- Vad är viktigt för beroendecentrum att tänka på i bemötandet av deltagaren?
- Hur vill du att de samverkar /återkopplar?

Ytterligare information för eget arbete

**1177 Vårdguiden Hjälp och stöd för den som vill ändra sina alkoholvanor**<br>https://www.1177.se/Stockholm/liv--halsa/tobak-och-alkohol/sa-kan-du-andra-dina-alkoholvanor/
**Alkoholhjälpen**<br>Webbsida med webbaserad rådgivning och självhjälpsmaterial för personer som funderar på sina egna alkoholvanor eller som är orolig för någon annans drickande. Här finns fakta, tips och ett populärt diskussionsforum.<br>http://www.alkoholhjalpen.se/

Etikprövningsmyndigheten
2022-02920-01-290879
2022-07-07

Fysisk aktivitet

Rekommendation

Fysisk aktivitet är när du rör på dig med hjälp av dina muskler. Rekommendationen är:

- 2,5 - 5 timmar i veckan av fysisk aktivitet som får dig lätt andfådd (exempelvis en rask promenad)
- Alternativt: 1,5 - 2,5 timmar av högintensiv träning som får dig rejält andfådd och svettig (exempelvis löpning)
- Minst två dagar i veckan ska träningen ovan innehålla allsidig styrketräning.
- Man ska inte sitta stilla mer än ca 25% (ca 4 h) av sin vakna tid och stillasittande bör avbrytas minst en gång i halvtimmen.

Varför det är viktigt

- Du blir piggare
- Minnet kan förbättras
- Du blir starkare
- Du blir mindre stressad och orolig
- Sömnen blir bättre
- Du minskar risken för många kroppsliga sjukdomar, t.ex. hjärt-kärlsjukdom och benskörhet
- Du minskar risken för psykisk ohälsa, bland annat demens och depression.

Av de saker vi har gått igenom, vilka skulle vara aktuella för dig tror du?

Exempel på vad du kan göra själv:

- **Börja med det som är enkelt. Exempel: avbryt stillasittande varje halvtimme.**
- **Mät varje dag i en vecka hur mycket du rör dig och på vilken nivå (medel eller högintensiv).**
- **Om du rör dig mindre än riktlinjerna, öka sakta din rörelse varje dag tills du når den nivå som rekommenderas eller som du är nöjd med.**
- **Hitta det sätt att röra på dig som passar dig (hämta något i ett annat rum, sträck på armarna, eller böj dig ner mot golvet.**
- **Om du har möjlighet, ta dig dit du ska med hjälp av dina muskler.**
- **Gå av bussen eller tåget en hållplats tidigare.**
- **Gör det du tycker är roligt!**
- **Gör det tillsammans med en vän eller familjemedlem.**
- **Värm upp ordentligt, låt kroppen återhämta sig mellan träningspass.**
- **På 1177 Liv och Hälsa hittar du fler tips om fysisk aktivitet.**

Vanliga motståndsyttranden och hur man hanterar dem

Deltagaren: - Jag har testat med det fungerar inte

- **Det stämmer, du har provat tidigare och det blev inte som du ville. Vad har du lärt dig av dina försök? Vad fungerade mindre bra? Och de gånger som fungerade bra, hur/vad gjorde du då?**
- **Du kan få instruktioner. Med FaR ingår personlig vägledning. Många gymkedjor erbjuder gratis introduktion.**
- **Våga fråga andra och personal om hjälp.**
- **Ibland finns det instruktioner på youtube.**

Deltagaren: - Jag är inte motiverad

- **Det är vanligt med låg motivation. Vad skulle kunna vara bra med att komma igång med träning/mer rörelse?**
- **När det har fungerat bra tidigare, vad gjorde du då?**
- **Vad har du för egenskaper som skulle kunna få dig att träna mer?**
- **Hur skulle det kännas om du lyckades träna mer?**

Deltagaren: - Jag tycker det känns bra att leva som jag gör nu

- **Okej. Det känns bra just nu men att inte röra sig tillräckligt kan skapa problem längre fram. Finns det några nackdelar med att röra sig för lite som du tror kan drabba dig?**

Deltagaren: - Det är för jobbigt

Etikprövningsmyndigheten
2022-02920-01-290879
2022-07-07

- **Okej. Du kanske börjar på en för hög/svår nivå? Börja med något du känner att du kan klara av. Ex en repetition.**
- **Kan vi hjälpa dig hitta en aktivitet som inte är för jobbig för dig?**
- **Vissa saker är väldigt jobbiga i stunden men det brukar kännas skönt när man är klar med träningspasset.**
- **Det som är jobbigt i stunden kan vara viktigt för att du ska må bra på lång sikt.**

Deltagaren: - Det är egentligen min familj/andra som tycker att jag rör mig för lite

- **Varför tror du de tycker att det är ett problem? Finns det något av det de säger som du håller med om?**
- **Ett förslag är att du bjuder in den som oroar sig att träna med dig.**

Deltagaren: - Det finns inte aktiviteter som passar mig/jag vet inte hur man gör

- **Okej. Vill du att jag försöker hjälpa till att hitta aktiviteter som passar dig? Finns det något du gjort som du gillat tidigare/finns det något du skulle vilja pröva på?**

Deltagaren: - Det är för tråkigt

- **Okej. Vad har du testat? Får jag föreslå några andra aktiviteter?**
- **Har du testat att träna med en kompis?**

Deltagaren: - Jag har inte tid

- **Är det okej att jag föreslår några saker som har med tid och träning att göra?**
- **Finns det någon annan aktivitet som du lägger mycket tid på idag som känns onödig?**
- **Träna korta pass.**
- **Träna med en vän/familjemedlem.**
- **Du kan träna hemma, avbryta stillasittande oftare.**
- **Även små saker räknas. Börja med 5 minuter varje dag.**

Deltagaren: - Jag kommer inte igång

- **Att komma igång kan vara svårt. Börja med något litet som du klarar och som går fort. Det kan vara hur litet som helst. Det viktiga är att du gör det till en rutin.**
- **Skapa rutiner. Bestäm en speciell dag och en tid den dagen då du tränar. Lägg fram träningskläder dagen innan**
- **Bestäm träff med någon att träna med.**

Deltagaren: - Det är för dyrt

Etikprövningsmyndigheten
2022-02920-01-290879
2022-07-07

- **Är det okej om jag föreslår aktiviteter som är gratis eller som du kan ha råd med? T.ex. pass på youtube, utegym, promenad/springa/rulla. Träna hemma.**

Deltagaren: - Jag känner mig värdelös/skäms för min kropp

- **Du är inte ensam med att känna dig värdelös eller skämmas.**
- **Vill du att vi pratar om hur du kan acceptera dig som du är?**
- **Flytta fokus från att undvika att känna obehag till att eftersträva förståelse för dig själv.**

Insatser och stöd

Ge insats enligt habiliteringsprogrammen men hänvisa också till aktörer utanför vården.

Se även https://www.fyss.se/ tips för dig som är vårdpersonal

**Aktör**	**Typ av insats**
Habilitering & Hälsa	Se habprogrammen
Habilitering & Hälsa	FaR

Etikprövningsmyndigheten
2022-02920-01-290879
2022-07-07

Hur du hänvisar vidare

Notera diagnos/funktionsnedsättning och behov av anpassning samt stöd om du hänvisar vidare.

Ytterligare information för eget arbete

**https://fritidsnatet.se/**
**Rekommendera lämpliga föreningar du känner till**
**1177 Vårdguiden Information och tips om fysisk aktivitet**<br>**Fysisk aktivitet och träning - 1177 Vårdguiden**
H&H har material för olika målgrupper.

Droger

Rekommendation

Vi avråder från användning av droger och receptbelagda mediciner som inte förskrivits av vården.

Varför är det är viktigt

Nu tänkte jag berätta lite kort om fördelarna med att minska/sluta med Cannabis.

Om det gäller användning av andra droger, hänvisa till beroendecentrum.

Om du slutar minskar på Cannabis kan du:

- **Bli gladare, mindre nedstämd**

- **Minska din ångest**
- **Minska din oro**
- **Sova bättre**
- **Bli bättre på att ta initiativ, mindre passiv**
- **Förbättra din inlärningsförmåga**
- **Förbättra ditt minne**
- **Få lättare att koncentrera dig**
- **Minska risken för personlighetsförändring och psykossjukdom**
- **Minska risken för långvarig trötthet/seghet**

Av de saker vi har gått igenom, vilka skulle vara aktuella för dig tror du?

Beroendeframkallande läkemedel:

Om du märker att dina läkemedel inte längre har samma effekt bör du prata med din läkare om det.

Det gäller t.ex. om du har:

- **Ökad ångest trots lugnande medel.**
- **Sämre sömn trots sömnmedel.**
- **Ökad smärta trots smärtlindrande medicin.**

Fortsatt användande kan alltså bidra till att förvärra de symptom som läkemedlet egentligen är tänkt att minska. Du kan ha hamnat i en ond cirkel som kan vara svår att bryta på egen hand.

Stämmer något av detta in på dig?

Det kan finnas risker med att sluta tvärt. Tala därför med din läkare för att få råd om hur du bäst kan trappa ner det läkemedlet du fått problem med.

Etikprövningsmyndigheten
2022-02920-01-290879
2022-07-07

Exempel på vad du kan göra

- **Det är jättebra att få stöd från andra personer som kan peppa dig.**
- **Det finns ställen där du kan få kontakt med andra som också har eller haft problem med droger, till exempel Anonyma narkomaner.**
- **Ta hjälp från vården. Du är inte ensam om ditt problem och det finns effektivt stöd att få exempelvis från beroendecentrum.**
- **Undvik miljöer där du har svårt att stå emot suget till droger.**
- **Försök jobba med andra levnadsvanor, t.ex. fysisk aktivitet. Det kan ge dig energi och motivation att vilja göra fler hälsosamma förändringar.**

Vanliga motståndsyttranden och hur man hanterar dem

Tänk på att det som deltagaren berättar inte ska leda till kritik eller negativa konsekvenser.

Deltagaren: - Ja, ja men det är inte så lätt…

- **Det stämmer att genomföra en förändring brukar vara svårt. <u>Samtidigt innebär</u> drogerna idag negativa konsekvenser för dig. Om du pratar med en expert på beroendecentrum kan de berätta om de negativa konsekvenserna.**

- **Det är inte lätt, men drogerna har orsakat svårigheter för din hälsa (konkretisera inom vilka områden) och dina relationer (konkretisera hur). På vilket sätt skulle det vara viktigt för dig att ha en bättre hälsa och bättre relationer?**

Deltagaren: - Jag kan koppla av, känna mer/mindre med droger

- **Jag förstår att det kan kännas skönt och att du har det behovet. Finns det något annat som kan få dig att känna på likande sätt men som är mindre skadligt än droger?**
- **Jag förstår att det kan kännas skönt i stunden men är du medveten om konsekvenserna på lite längre sikt? Skulle det vara okej om du pratade med någon expert om detta så kan du bestämma dig sen?**

Deltagaren: - Men det hjälper mot smärta

- **Jag förstår att du har ont och det ska du få hjälp med. Det är viktigt att du följer din läkares instruktion för vilken medicin och hur mycket du ska ta för att lindra smärtan. Det finns en risk att även läkemedel har negativa effekter om de används på fel sätt. Är det okej om du kontaktar din läkare så ni kan diskutera hur du kan få hjälp med din smärta på ett säkert sätt?**

Etikprövningsmyndigheten
2022-02920-01-290879
2022-07-07

Deltagaren: - Men Cannabis är ju lagligt på många plaster? Cannabis är inte så farligt.

- **Jag förstår att det kanske känns skönt/kul/avslappnade i stunden men Cannabis kan leda till ångest, nedstämdhet, sämre sömn, sämre koncentration och minne. Det finns saker som är lagliga som inte är bra för oss. Alkohol och tobak är exempel på det. Kan du tänka dig att ringa och prata med någon som är expert?**

Vill patienten inte prata vidare har du gjort vad du kunnat och kan notera detta i journalen. Många gånger blir det startskottet för en förändring.

Om deltagaren inte är tillräckligt motiverad att sluta med droger: Byt fokus till en annan levnadsvana. Lyft frågan i framtiden igen.

Insatser och stöd

Om Lev redan testats eller inte bedöms fungera, välj då åtgärd på kvalificerat rådgivande nivå enligt nedan.

**Aktör**	**Typ av insats**
Beroendemottagningar, se 1177.	Kvalificerat rådgivande nivå
Elevhälsan (för unga vuxna som inte vill ha kontakt med beroendemottagningar)	

Hur du hänvisar vidare

Remiss via TakeCare:

Notera diagnos/funktionsnedsättning och behov av anpassning samt stöd i kontakten.

Detta föreslår beroendecentrum Stockholm att remiss via TakeCare även kan innehålla för att förbättra kontakten.

- Aktuellt substansbruk samt omfattning.
- Görs några andra insatser som kan stötta/förebygga missbruket?
- Andra pågående insatser (exempelvis via kommunen eller psykiatrin).
- Aktuell social situation (boende, arbete, ekonomi).
- Finns suicidalitet i anamnesen?
- Våldshistorik?
- Minderåriga barn?
- Vad är viktigt för beroendecentrum att tänka på i bemötandet av deltagaren?
- Hur vill du att de samverkar /återkopplar?

Ytterligare information för eget arbete

droghjälpen.se Nationell stödlinje dit även vårdpersonal kan höra av sig för att få stöd och vägledning i frågan om droganvändning (gäller alla droger, även läkemedel).
cannabishjälpen.se Information om cannabis och vart en kan få hjälp (förvaltas av Beroendecentrum Stockholm).
Drugsmart
Anonyma narkomaner

Etikprövningsmyndigheten
2022-02920-01-290879
2022-07-07

Skärmtid

Rekommendation

Skärmtid kan vara utvecklande och bra men ska inte gå ut över andra levnadsvanor. Vi kan även stötta dig i hur du kan vara juste på internet.

Varför det är viktigt

Positivt:

- **Skärmtid kan ge dig tillgång till pedagogiskt material för lärande.**
- **Det kan innebära rolig underhållning.**
- **Det kan hjälpa dig komma i kontakt med andra.**

Negativt:

- **För mycket skärmtid kan få dig att bli för stillasittande.**
- **Din smartphone kan störa din koncentration när du ska göra något annat om du inte lägger bort den.**

- **Det kan få dig att inte ha tillräckligt mycket mänsklig kontakt.**
- **Det kan få dig att inte utveckla andra intressen.**
- **Det kan få dig att slarva med mat.**
- **Det kan få dig att sova sämre.**
- **Du kan göra andra och dig själv illa om du skriver elaka saker eller besöker olagliga sidor.**
- **Att spela om pengar på nätet (nätcasinon) kan vara svårt att avsluta.**

Av de saker vi har gått igenom, vilka skulle vara aktuella för dig tror du?

Exempel på vad du kan göra

- **Gör inte saker som du ångrar efteråt.**
- **Lägg bort din smartphone när du gör andra saker (äter, träffar vänner, fokuserar på något annat mm). Gärna i ett annat rum.**
- **Öka din fysiska aktivitet (gärna med vänner eller familj om det passar dig).**
- **Ta pauser när du spelar och rör på dig.**
- **När du spelar, gör det i samma rum som någon annan så att ni kan hjälpa varandra ta pauser.**
- **Planera din skärmtid:**
  - **Vad ska du göra?**
  - **Hur länge ska du ha skärmtid?**
  - **Bestäm innan hur mycket du ska spela/använda sociala medier. Det kan kännas ovant men det är bra att begränsa användandet till en eller några gånger per dag.**

- **Utvärdera din skärmtid:**
  - **Blev det som du hade tänkt dig?**
  - **Missade du att göra andra saker som du hade tänkt för att skärmtiden tog för lång tid?**
- **Sätt gränser:**
  - **Spelpaus.se är en webbplats för att stänga av sig från spel och direktmarknadsföring från licenserade spelbolag. Gäller spel online, i butik, på bana, Vegas och Casino Cosmopol.**

Vanliga motståndsyttranden och hur man hanterar dem

Deltagaren: - Ja, men det är inte så lätt

- **Det stämmer att minska på något som är roligt är inte alltid så lätt, samtidigt skulle du få mer tid över för annat som får dig att må bra både fysiskt och psykiskt.**
- **Du behöver inte tro på vad jag säger. Skulle du vilja göra ett experiment och prova minskad skärmtid under en vecka och se hur det skulle påverka dig?**

Deltagaren: - Jag träffar vänner online som jag inte hade träffat annars.

- **Det är jättebra att du träffar vänner via skärmen. Jag säger inte att du ska sluta med det roliga du gör på din skärm men det är viktigt att det finns tid kvar för andra aktiviteter och hälsosamma levnadsvanor.**

Etikprövningsmyndigheten
2022-02920-01-290879
2022-07-07

- **Det finns många fördelar med att vara online helt klart. Skulle du kunna tänka dig att träffa de vänner du endast brukar träffa digitalt och ”spela” tillsammans?**

Deltagaren: - Jag vill inte minska på min skärmtid

- **Okej, men kan du tänka dig**
  - **att ta fler rörelsepauser så att du inte blir stillasittande?**
  - **att inte spela för sent så att sömnen inte blir för påverkad?**
  - **att äta på regelbundna tider och hälsosam mat?**

Insatser och stöd

I habprogrammen finns insatser för att skapa struktur i vardagen. Det finns ingen specifik behandling i dagsläget gällande ohälsosamma skärmvanor. Om deltagaren har problem med spel om pengar kan ni vända er till beroendemottagningar, se 1177.

Ytterligare information för eget arbete

Ge förslag på relevant informationsmaterial.

Sömn

Rekommendation

Längd: 7-9 timmar per natt. Regelbundenhet: Du bör lägga dig och gå upp samma tider varje dag. Kontinuitet: Sov helst inte middag. Max 20 min om du måste.

Varför det är viktigt

(Se även checklistan gällande sömn i vårdplaneringsmodulen.)

- **Du minskar risken för att bli sjuk, du förbättrar ditt immunförsvar**
- **Du blir gladare och piggare**
- **Du får lättare att koncentrera dig**

- **Du kan hantera stress bättre**
- **Du minskar risken för oro och ångest**
- **Du minskar risken för allvarliga sjukdomar på lång sikt: diabetes, hjärt-kärlsjukdomar mm.**

Av de saker vi har gått igenom, vilka skulle vara aktuella för dig tror du?

Etikprövningsmyndigheten
2022-02920-01-290879
2022-07-07

Exempel på vad du kan göra själv:

Här kan du välja att ge Habilitering & Hälsas åtgärd för sömn. Om du bedömer att det inte behövs eller om deltagaren vill jobba på egen hand kan du gå igenom exemplen nedan.

- **Minska stressen: avslappningsövningar och mindfulness.**
- **Rör på dig. Fysisk aktivitet gör att du blir naturligt trött på kvällen. Det kan även motverka stress, ångest och depression vilket också underlättar för din sömn.**
- **Stillsamma aktiviteter före sängdags. Försök att ta det lugnt 2 timmar innan du lägger dig.**
- **Undvik ljus på kvällen. Minska ner på ljuset från din mobil eller datorskärm och använd rullgardin på sommarhalvåret.**
- **Undvik koffein, nikotin och alkohol. Undvik kaffe, te, läsk eller energidryck sex timmar innan du lägger dig.**
- **Låt sängen vara en sovplats. Jobba inte i sängen. Se till att ditt sovrum är mörkt och svalt. Ligg inte sömnlös i sängen mer än 30 min. Gå upp och gör något avkopplande som att läsa eller lyssna på musik. Gå tillbaka när du känner dig trött igen.**

Vanliga motståndsyttranden och hur man hanterar dem

En vanlig anledning till motstånd är att man vill ha snabb lösning; ”quick fix”. Man är för trött för att man inte sover vilket gör att man inte orkar ta tag i det. Du behöver därför motivera till att det kan ta tid. Det krävs ibland täta uppföljningar för att motivera tillräckligt.

Här är det svårt att nå hälsovinster på en gång. Om man provar sig fram kommer man kanske att sova dåligt minst i en vecka ändå. Ett sätt att motivera trots de negativa konsekvenserna på kort tid kan vara att förstärka en styrka eller självförtroende. Många känner att deras självförtroende växer när de inte ger upp trots att det inte är så lätt i början.

Etikprövningsmyndigheten
2022-02920-01-290879
2022-07-07

Deltagaren: - Alla saker ni radar upp känns verkningslösa och jag har testat dem.

- **Den första veckan får man inte alltid någon effekt. Det kan kännas som att du aldrig kommer att kunna sova bättre. Det vi vet är att efter ca en vecka blir det bättre när man följer rekommendationerna. Vad skulle hjälpa dig att inte ge upp när du provar under en vecka?**
- **Hur kan vi stötta dig igenom detta viktiga arbete med din sömn?**
- **Vad bra att du har testat. Vilken fysisk aktivitet testade du?**
  - **Får jag föreslå konditionsträning: att du promenerar, cyklar, springer så att din puls går upp och du blir andfådd. Om du gjort det, kan du tänka dig att testa att lägga in fler pass eller träna lite längre perioder?**
  - **Har du testat yoga?**
  - **Träna på morgonen eller senast eftermiddagen. Kvällsträningen kan göra vissa piggare.**

Deltagaren: - Jag orkar inte ta tag i min sömn.

- **Jag förstår att det är jobbigt och att trötttheten gör det svårt att orka göra saker som skulle kunna ge dig bättre sömn. Hur kan jag stötta dig för att orka försöka mer?**

Insatser och stöd

Vid problem med sömn ges inte Lev i första hand utan här används sedvanliga rutiner vid Habilitering & Hälsa. Insats för sömn kan även ges via Lev om det föreligger särskilda skäl så som lång kö till eller om deltagaren inte vill gå vidare med sedvanlig behandling.

**Aktör**	**Typ av insats**
Habilitering & Hälsa	Se habprogrammen. (Kvalificerat rådgivande insats).
Husläkarmottagning (vårdcentral)<br>Psykiatrisk mottagning vid samtidigt psykiatriskt tillstånd som kräver specialistvård	• Sömnskola<br>• Psykologisk behandling<br>• Läkemedel<br>• Övriga levnadsvanor

Hur du hänvisar vidare

Remiss via TakeCare:

Notera diagnos/funktionsnedsättning och behov av anpassning samt stöd i kontakten.

Etikprövningsmyndigheten
2022-02920-01-290879
2022-07-07

Ytterligare information för eget arbete

**1177 vårdguiden**<br>Information om hur man kan förbättra sin sömn<br>https://www.1177.se/Stockholm/liv--halsa/stresshantering-och-somn/
Se habprogrammen för val av övningar och material

Intressen/Aktiviteter/Sysselsättning

Rekommendation

- **Att ha intressen i vardagen som känns meningsfulla (som till exempel ger en känsla av delaktighet med andra/samhället, och att du genom att göra aktiviteten förstår eller formar vem du är).**
- **Att ha intressen som känns trivsamma (att det är roligt, mysigt eller får dig att känna positiva känslor).**

Varför det är viktigt

Positivt:

- **Att göra aktiviteter som du tycker är trivsamma och meningsfulla kan**
  - **motverka stress, oro och nedstämdhet**

- **förbättra din sömn**
- **göra dig lyckligare**
- **förbättra din fysiska hälsa (exempelvis BMI, blodtryck)**
- **ge dig en känsla av tillhörighet/delaktighet**

Negativt

- **Att göra någon aktivitet för mycket kan**
  - **ta viktig tid från andra viktiga aktiviteter och levnadsvanor**
  - **innebära att du inte får tid att återhämta dig (efter fysisk träning eller mentalt krävande aktiviteter)**

Av de saker vi har gått igenom, vilka skulle vara aktuella för dig tror du?

Exempel på vad du kan göra själv

Detta är ett område där Habilitering & Hälsa ger insatser på kvalificerad nivå. Om du bedömer att det inte behövs eller om deltagaren vill jobba på egen hand kan du gå igenom exemplen nedan i fas 1 eller 2. Lägg även dessa exempel i deltagarens stöd- och behandlingsplattform.

- **Gör någon aktivitet som du mår bra av varje dag.**
- **Ta kontakt med intresseföreningar/organisationer eller din kommun och låt dem komma med tips på vad du kan göra.**
- **Hitta en vän/familjemedlem eller bekant som gillar att göra samma aktivitet som du och bestäm en rutin (en dag/tid då ni ses).**
- **Hitta balans mellan kravfyllda aktiviteter och aktiviteter som får dig att må bra.**
- **Hitta balans så att din roliga aktivitet inte går ut över andra viktiga aktiviteter och levnadsvanor.**
- **Följ ditt hjärta. Vad gör dig nyfiken och vad skulle kännas kul för dig att göra?**
- **Försök förstå varför saker du egentligen vill göra inte blir av.**
- **Berätta för andra vad du vill göra så de kan peppa dig så att det blir av.**

Etikprövningsmyndigheten
2022-02920-01-290879
2022-07-07

Strategier från MI för att hantera motstånd eller öka motivation

Deltagaren: - Ja, ja men det är inte så lät

- **Du är modig som provar trots att det inte är så lätt. På vilket sätt skulle vi kunna stötta dig?**

Deltagaren: - Mina förutsättningar har förändrats och jag kan inte längre göra det jag vill

- **Det stämmer att dina förutsättningar har ändrats. Det kan vara en viktig sak att prata om och vi bokar gärna in stöd för det. Samtidigt som du utifrån hur det är nu vill göra roliga och meningsfulla saker. Kan vi prata om saker du kan göra idag?**

Deltagaren: - Jag fattar inte hur jag ska göra

- **Vad bra att du säger det. Är det okej om vi gör ett försök att försöka förstå tillsammans?**
  - **Vilket är ditt mål?**
  - **Hur ska du nå målet?**
  - **Testa.**
  - **Utvärdera, hur gick det?**
  - **Justera din plan för hur du ska målet om det behövs.**
  - **Testa igen.**

Deltagaren: - Jag vet vad jag vill göra men det blir inte av

- **Vad bra att du vet. Då har du kommit långt. Kan vi prata om hur vi kan öka chansen för att det blir av?**

Deltagaren: - Jag orkar inte

- **Du känner dig energilös och trött. Samtidigt visar forskning att du får energi av att göra saker som du tycker om eller är meningsfulla. Kan vi prata om vilka aktiviteter som i nuläget tar energi och vilka som skulle kunna fylla på med energi?**

Etikprövningsmyndigheten
2022-02920-01-290879
2022-07-07

Insatser och stöd

Ge insats enligt habiliteringsprogrammen men hänvisa också till aktörer utanför vården. Om du hänvisar externt ska du fort fortsätt följa deltagaren via Lev.

**Aktör**	**Typ av insats**
Habilitering & Hälsa	Se habiliteringsprogrammen

Ytterligare information för eget arbete

**https://fritidsnatet.se/**
**Rekommendera lämpliga föreningar**

Sexuell hälsa

Rekommendation

Sexualitet är väldigt individuellt. Det finns tre områden som man kan tänka på för att komma fram till om man vill göra någon förändring.

- **<u>Njutning</u> – Om du inte är nöjd kan det vara en anledning att få stöd och tips om hur du kan njuta mer.**
- **<u>Smärta, sjukdomar, hinder</u> – Om sex är svårt för dig kan du få hjälp och information som kan förändra din situation till det bättre.**
- **<u>Trygghet</u> – Sex ska kännas bra för dig och den du eventuellt har sex med. Är du osäker kan det vara bra att få tips och stöd kring detta.**

Varför det är viktigt

- **Sex kan**
  - **få dig att njuta och må bra i stunden**
  - **minska stress på längre sikt**
  - **förbättra din sömn**
  - **förbättra ditt immunförsvar**
  - **förbättra din fysiska hälsa (exempelvis hjärtat)**
  - **förbättra ditt humör**
  - **minska smärta**
  - **öka känslan av närhet om du har det men någon annan**
- **Sex ska kännas bra innan, under och efter för dig och den som du eventuellt har sex med.**

Etikprövningsmyndigheten
2022-02920-01-290879
2022-07-07

Av de saker vi har gått igenom, vilka skulle vara aktuella för dig tror du?

Exempel på vad du kan göra själv

Om du vill börja själv. Här hittar du mer information om följande områden:

- Njutning: umo.se, youmo.se, rfsu.se, rfsl.se, fliken ”sexualitet” på funktionshindersguiden.se, 1177.se fliken”sexlust och kåthet”.
- Smärta, sjukdomar, hinder: 1177/vårdguiden
- Trygghet: umo.se, youmo.se, rfsu.se, rfsl.se, polisen, origo.se

Vanliga motståndsyttranden och hur man hanterar dem

Sträva efter professionella värderingar och förhållningsätt. Du ska inte vara privat och använda dig själv som exempel. Om du inte har en utbildning inom detta område är din uppgift främst att lyfta frågan (exempelvis genom att ställa frågorna som finns i levandsvaneevaluering, Lev), och berätta att detta är något som deltagare kan få hjälp med. Din uppgift är sedan att hjälpa deltagaren att komma i kontakt med rätt stöd. Förslag på stöd finner du under ”stöd och åtgärder bilaga 2”.

Deltagaren: - Jag tycker det är väldigt svårt, pinsamt, obekvämt.

- **Ja det är många som upplever att det är svårt att prata om sexuell hälsa. Det kan kännas jobbigt, pirrigt osv. Men det brukar finnas en nyfikenhet och önskan också.**
- **De personer som ger stöd i sexuell hälsa är riktigt duktiga på det. De är vana att möta personer som du och de respekterar att du tycker det är ovant att prata om vissa saker. Det är på dina villkor.**

Deltagaren: - Jag har försökt tidigare men det har inte gått bra (Njutning)

- **Okej, det var bra att du var modig och gjorde det. Tyvärr blir det ofta inte som vi har förväntat oss, särskilt inte i början.**
- **Vad mina kollegor brukar vara bra på är att berätta om många fler alternativ man kan testa som man inte hade tänkt på innan.**

Deltagaren: - När jag tidigare pratat om min sexualitet med vårdpersonal har de bemött mig dömande.

- **När du tidigare sökt stöd har du inte fått ett professionellt bemötande. Det är förståeligt att du är tveksam till att det finns hjälp att få i vården. Habilitering & Hälsas syn på sexualitet är att den kan ta sig många olika uttryck och att alla har rätt till god sexuell hälsa. Nu när jag berättat det, skulle du vilja prata vidare?**

Deltagaren: - Jag tror inte att det går att uppleva sexuell njutning med min funktionsnedsättning.

Etikprövningsmyndigheten
2022-02920-01-290879
2022-07-07

- **Sexuell njutning kan upplevas på många olika sätt och behöver inte alltid förknippas med samlag och orgasm. Det finns många anpassningar, hjälpmedel och tips som andra personer med din funktionsnedsättning tycker har fungerat bra som stöd för deras sexuella hälsa. Om du vill kan jag hjälpa dig att komma i kontakt med kollegor som är bra på de här anpassningarna.**

Deltagaren: - Jag vågar inte göra en undersökning för jag ser så konstig ut där nere.

- **Jag tror att många kan känna igen sig i din känsla av att ens könsorgan ser avvikande ut. Barnmorskorna och läkarna på mottagningarna gör många undersökningar och vet att variationen av hur könsorgan kan se ut är stor.**

Deltagaren: - Mina föräldrar skulle bli arga om jag pratade om sexuell hälsa med er. (Även vuxna över 18 har föräldrar som styr sina barn).

- **Det vi säger här stannar här och det är bara du som bestämmer om du vill prata eller inte. Vi har träffat andra personer som varit i ungefär samma situation som du. Får jag berätta lite om vart man kan vända sig för att få stöd kring detta? (Jag kan också hjälpa dig att få kontakt med origo, polisen, socialtjänsten eller andra om det behövs.)**

Deltagaren: - Jag måste hela tiden förklara så mycket om mitt kön, min identitet osv så jag orkar inte lyfta frågan längre.

- **Du beskriver att du känner ett ansvar att lära andra om din könsidentitet och din sexuella läggning, och att det ibland är jobbigt och hindrar dig att söka stöd för din sexuella hälsa. Jag tänker att det inte ska vara ett krav att komma ut varje gång man söker råd. Du bestämmer själv hur mycket du vill berätta för andra och när.**

Deltagaren: - Det blir så pinsamt för andra om jag frågar om min sexuella hälsa.

- **Du har uppfattat att andra blir generade när du ställt frågor om din njutning. Jag tänker att vi människor ofta är rädda att svara fel när vi inte har så mycket kunskap. Får jag berätta lite för dig hur vi jobbar med sexuell hälsa här, och vilka vi hänvisar till när vi tror man kan få bättre hjälp på någon annan mottagning?**

Deltagaren: - Jag kommer ändå aldrig att träffa någon.

- **Du berättar att det inte är aktuellt för dig att ha sex med någon annan. Sex med sig själv ligger till grund för god sexuell hälsa hos många oavsett om man kommer att träffa någon partner eller inte. Men det är du som bestämmer hur stor plats sexualiteten får ta i ditt liv. Om det inte passar att jobba med den sexuella hälsan just nu så går det jättebra att komma tillbaka till frågan vid ett senare tillfälle.**

Etikprövningsmyndigheten
2022-02920-01-290879
2022-07-07

Insatser och stöd

Om du hänvisar externt fortsätt följa deltagaren via Lev.

Aktör	Typ av insats	För vem
H&H	Se habprogrammen	Alla
Mansmottagningen<br>08-123 393 90	Provtagning<br>Stödsamtal sexualitet	Över 23, män och transpersoner
Ungdomsmottagning<br>27 st i regionen<br>www.umo.se<br>YOUMO	Provtagning<br>Stödsamtal bl a om sexuell hälsa	Under 23
Stockholms mottagning för sexuell och reproduktiv hälsa<br>08-123 405 00	Provtagning<br>Stödsamtal sexualitet	Över 18
Barnmorske-mottagninar.se	Allmänt för kvinnor	Kvinnor
Spinalis<br>08-555 44 250	För personer med ryggmärgsskada	
Origo<br>08-508251 20	Resurscentrum mot hedersrelaterat förtryck och våld	13-26 år

Hur du hänvisar vidare

Remiss via TakeCare:

Notera diagnos/funktionsnedsättning och behov av anpassning samt stöd i kontakten.

Ytterligare information för eget arbete

Njutning: umo.se, youmo.se, rfsu.se, rfsl.se, fliken ”sexualitet” på funktionshindersguiden.se<br>**1177 Vårdguiden Information om sexuell hälsa**<br>Sexuell hälsa - 1177 Vårdguiden (t.ex. fliken”sexlust och kåthet”)<br>Könsidentitet och sexuell läggning - 1177 Vårdguiden
Könsorgan - 1177 Vårdguiden (smärta, sjukdomar, hinder)<br>Våld, övergrepp och sexuella trakasserier - 1177 Vårdguiden
Trygghet: umo.se, youmo.se, rfsu.se, rfsl.se, polisen, origo.se
Appen (eller hemsidan) ”Mitt privatliv” Privatliv (mittprivatliv.se)

Etikprövningsmyndigheten
2022-02920-01-290879
2022-07-07

Vänner/Familj

Rekommendation

Hur man vill ha sina relationer är individuellt. En del trivs med att umgås mycket med andra, andra trivs bättre för sig själva. För att komma fram till om man vill göra någon förändring i sina egna relationer finns några saker man kan tänka på:

- **Om man är nöjd med sina relationer: Att träffa andra människor kan vara roligt och ge trygghet men alla har olika behov.**
- **Att ha någon man litar på: Alla vill inte ha många vänner men man brukar må bra av att kunna prata med någon som man litar på.**
- **Att inte känna sig ensam. Ensamhet är något som många mår dåligt av.**

Varför det är viktigt

- **Relationer kan ge känsla av meningsfullhet, att man känner sig behövd.**
- **Relationer kan ge trygghet och stöd.**
- **Goda relationer kan minska psykiska problem som exempelvis depression, ångest, minnesproblem och stress.**
- **Goda relationer kan till och med minska risken för kroppsliga sjukdomar såsom stroke, hjärt-kärlsjukdomar och demens.**

Exempel på vad du kan göra själv

- **Skyll inte på dig själv om du känner dig ensam.**
- **Om något positivt händer, dela det med andra. Det behöver inte vara stora saker. Testa att göra det genom att berätta det som hänt för någon istället för att dela via sociala medier.**

- **Träffa vänner ansikte mot ansikte (IRL) och inte bara via skärm.**
- **Engagera dig i en förening (exempelvis idrottsförening, intresseorganisation, kyrkan).**
- **Var aktiv (inte passiv) när du är online. Chatta och interagera med andra.**
- **Ge tips och hjälp i forum på internet. Andra kan behöva ditt stöd.**
- **Anstränga dig för att skapa goda/fungerande relationer till din familj och dina vänner.**
- **Var snäll mot dig själv!**

Vanliga motståndsyttranden och exempel på hur man kan hantera dem

Deltagaren: - Jag har väldigt svårt att förstå mig på sociala regler

- **Du säger att du har svårt att förstå hur du ska umgås med andra så att det känns bra. Skulle du vilja att det var på ett annat sätt? I så fall, skulle du vilja träna på att förstå andra bättre?**
- **Det brukar vara lättare att umgås kring en aktivitet. Har du något intresse som du skulle kunna med andra?**
- **Många känner som du, att det är svårt att förstå sig på sociala regler. Om man är intresserad, så går det att träna sig i detta. Exempelvis lära sig mer om sociala regler, småprat, hur man påbörjar och avslutar samtal, vad man kan göra för att få det att fungera ”smidigare” i sociala kontakter. Ibland kan det mer handla om att hitta bra former för umgänge, där man känner sig mer trygg, och välja sammanhang och personer som det fungerar bättre med.**

Deltagaren: - Jag har social fobi

- **Tack för att du delar med dig. Det är många som precis som du kan känna oro och obehag i vissa sociala situationer. Det blir lätt så att man undviker dem. Man kan ha social fobi och ändå vilja träffa andra om det gick. Är det så för dig? Vill du att vi pratar om hur du skulle kunna göra det utan att det blir för jobbigt?**

Deltagaren: - Jag orkar inte umgås med andra. Det tar för mycket av min energi.

- **Att umgås med andra kan vara väldigt jobbigt. Eller att träffa många människor samtidigt, eller träffas i en stimmig miljö. Att umgås med andra på ett sätt som passar en själv kan istället ge energi. Vill du att vi pratar om hur det skulle kunna funka för dig?**

Deltagaren: - Jag har försökt men folk tycker inte om mig

- **Du upplever att folk inte tycker om dig och det måste kännas jobbigt. Du ska inte behöva känna så. Vill du att vi pratar om hur du skulle kunna känna annorlunda i mötet med andra?**

Deltagaren: - Jag har massa vänner

- **Om du känner dig nöjd och inte vill göra någon förändring så kan vi fokusera på någon annan levnadsvana, men anledningen till att vi tar upp det är att du svarade att du kände dig ensam. Stämmer det? I så fall, vill du att vi pratar om hur du kan känna dig mindre ensam?**

Etikprövningsmyndigheten
2022-02920-01-290879
2022-07-07

Deltagaren: - Ja, men det är inte så lätt

- **Det stämmer, samtidigt som du längtar efter en kompis/en partner. Vad säger du om att träna lite på hur man närmar sig någon som kanske blir en kompis? Eller hitta mötesplatser där du kan träffa andra som har liknande intressen som du?**

Deltagaren: - Jag har försökt och det går inte

- **Det var modigt av dig att försöka och det går inte alltid som vi vill. Va det något av det du testade som fungerade lite? Om inte, ibland kan man behöva försöka flera gånger. Det kan hända att vi kommer på något nytt som du inte testat. Vill du att vi testar igen?**

Insatser och stöd

Ge insats enligt habiliteringsprogrammen men hänvisa också till aktörer utanför vården. Om du hänvisar externt fortsätt följa deltagaren via Lev.

**Aktör**	**Typ av insats**
Habilitering & Hälsa	Se habprogrammen

Ytterligare information för eget arbete

H&Hs eget material
Tipsa om lämpliga intresseföreningars aktiviteter och möteslokaler mm.
Tipsa om lämpliga föreningar, aktiviteter och träffpunkter i samhället utifrån personens intressen.

Etikprövningsmyndigheten
2022-02920-01-290879
2022-07-07

Slut på bilaga 1

Exempel på beteendeanalys inklusive tips på strategier– bilaga 2

Denna bilaga är till för dig som behandlare för att få exempel på och övning i hur man kan synliggöra beteenden och konsekvenser. Följande bilaga läser du innan du träffar deltagaren.

Tobak

Kom ihåg att samverka med den som ger eventuell insats externt och fokusera på det som kompletterar deras verksamhet:

- Att de finns en fortsatt kontakt.
- Att andra levnadsvanor som stöttar minskningen av tobak fungerar.

Oönskat beteende

Situation/stimuli	Beteende	Kortsiktig konsekvens	Långsiktig konsekvens
Ska ringa sluta-röka-linjen<br><br><u>Strategi:</u><br>• Be en vän fråga om du gjort det.<br>• Skriv in i din kalender	Ringer inte	• Slipper ta en ny social kontakt<br>• Slipper den ansträngning det innebär att minska på tobak<br>• Ångest för att inte ha följt planen	• Ökad risk för sjukdomar såsom cancer, hjärtkärlsjukdomar och KOL.<br>• Sämre kondition<br>• Bli sjuk oftare
Känner stress<br><br><u>Strategi:</u><br>• Gå en promenad<br>• Träna<br>• Kan du undvika situationen som leder till stress?	Använder tobak	• Stressen minskar<br>• Ångest för att inte ha följt planen	• Ökad risk för sjukdomar såsom cancer, hjärtkärlsjukdomar och KOL.<br>• Sämre kondition<br>• Bli sjuk oftare
Umgås med vän som röker<br><br><u>Strategi:</u><br>• Fråga <u>innan</u> ni ses om hen kan röka när du inte märker det.<br>• Bestäm innan och träna på vad du ska göra när frestelsen uppstår<br>• Ta ett nikotinpreparat	Använder tobak	• Samhörighet<br>• Mår bra	• Ökad risk för sjukdomar såsom cancer, hjärtkärlsjukdomar och KOL.<br>• Sämre kondition<br>• Bli sjuk oftare

Etikprövningsmyndigheten
2022-02920-01-290879
2022-07-07

Önskat beteende

Situation/stimuli	Beteende	Kortsiktig konsekvens	Långsiktig konsekvens
Ska ringa sluta-röka-linjen	Ringer/tar kontakt<br><br><u>Underlättande strategier:</u><br>• Boka in i din kalender<br>• Skriv ned numret	• Ångest eller ansträngning<br>• Minskad ångest efter att ha följt planen	• Minskad risk för sjukdomar<br>• Bättre kondition
Känner stress	• Ta en kort promenad<br>• Ta ett nikotinpreparat<br>• Drick rogivande te<br><br><u>Underlättande</u>	• Frustration<br>• Stolthet för att ha stått emot begäret	• Ökad förmåga att stå emot<br>• Minskad risk för sjukdomar<br>• Bättre kondition

	<u>strategier:</u><br>• Se till att ha kläder för alla väder och sköna promenadskor<br>• Se till att ha med dig nikotinpreparat<br>• Gör tedrickande till en rutin du gillar (köp fin kopp, gör det med en vän)		
Umgås med vän som röker	• Tar ett nikotintuggummi<br>• Säger att du inte vill fresta dig själv, ber att få tala vidare när hen rökt klart<br>• Ber att hen hjälper dig peppa dig att stå emot<br><br><u>Underlättande strategier:</u><br>• Träna på att stå emot innan.	• Frustration<br>• Känslan av att du missar något gott<br>• Stolthet för att du stått emot begäret	• Ökad förmåga att stå emot<br>• Minskad risk för sjukdomar<br>• Bättre kondition

Etikprövningsmyndigheten
2022-02920-01-290879
2022-07-07

Kost

Oönskat beteende

**Situation/stimuli**	**Beteende**	**Kortsiktig konsekvens**	**Långsiktig konsekvens**
Du är hungrig och ska bestämma vad du ska äta?<br><br><u>Strategi:</u><br>• Försök att bestämma innan du blir hungrig<br>• Se till att ha bara nyttiga alternativ hemma<br>• Gå till restaurang som har vegetariska	Du väljer ett onyttigt alternativ	Det är gott och får dig att känna tillfredsställelse	Då ökar risken för en lång rad av sjukdomar

eller fiskalternativ			
Du är egentligen inte hungrig men är uttråkad och går till kylskåpet<br><br><u>Strategi:</u><br>• Drick vatten<br>• Ha bestämda tider när du äter<br>• Ta en promenad<br>• Ha en skål med grönsaker att äta	Du gör några mackor som du äter	Du motverkar den tråkiga känslan men belöningen du känner av de goda mackorna	Då ökar risken för en lång rad av sjukdomar
Du ska handla<br><br><u>Strategi:</u><br>• Bestäm innan och skriv på en lista<br>• Beställ hem mat via nätet<br>• Köp baserat på de rekommendationer du fått: exempelvis fisk och grönt	Du köper en massa, fika, snacks och onyttig mat	Du tänker på allt gott du ska äta och blir glad av det	Med alla dessa godsaker hemma är det svårare att fatta hälsosamma beslut.

Etikprövningsmyndigheten
2022-02920-01-290879
2022-07-07

Önskat beteende

**Situation/stimuli**	**Beteende**	**Kortsiktig konsekvens**	**Långsiktig konsekvens**
Du är hungrig och ska bestämma vad du ska äta?	Det är ofta svårare att fatta bra beslut när man är hungrig men du väljer fisk och grönt<br><br><u>Underlättande strategier:</u><br><br>• Fråga personalen efter nyttiga goda alternativ<br>• Skriv ned en lista på saker nyttiga rätter du tycker är goda. Se till att alltid ha den listan med dig i mobilen och använd den när inspirationen är	• Trist/svårt att inte få välja det allra godaste<br>• Efter middagen är du sannolikt nöjd ändå	• Du minskar risken för en lång rad av sjukdomar<br>• Ökad tilltro till din förmåga

	låg<br>• Gå inte ut för hårt. Bättre att lägga till någon liten hälsosam vana varje vecka och sedan fortsätta så<br>• Äta regelbundet och hoppa inte över måltider		
Du är egentligen inte hungrig men är uttråkad och går till kylskåpet	Du inser att det är för att du har tråkigt/känner dig frustrerad och gör en kopp te och några morotsstavar<br><br><u>Underlättande strategier:</u><br>• Se till att bra alternativ: Te, grönsaker, nötter.<br>• Ha inte saker som frestar dig: exempelvis godis, läsk eller chips-	• Trist/svårt att inte få välja det allra godaste	• Du minskar risken för en lång rad av sjukdomar<br>• Ökad tilltro till din förmåga
Du ska handla	Du <u>planerar innan</u> du åker till affären vad du ska köpa. Du köper några väl valda godsaker men inte så mycket som tidigare<br><br><u>Underlättande strategier:</u><br>• Ha alltid listor i mobilen med bra ingredienser och råvaror att köpa<br>• Ha en lista i mobilen där du skrivit ned listan över saker du brukar handla	• Trist/svårt att inte få välja det allra godaste<br>• Spännande att testa nya rätter	• Färre godsaker hemma som frestar dig<br>•

Etikprövningsmyndigheten
2022-02920-01-290879
2022-07-07

Alkohol

Kom ihåg att samverka med den som ger eventuell insats externt och fokusera på det som kompletterar deras verksamhet:

- Att det finns en fortsatt kontakt.
- Fråga om det är något annat deltagaren vill ha stöd kring som kan komplettera annan insats. Exempelvis andra levnadsvanor.

Oönskat beteende

Situation/stimuli	Beteende	Kortsiktig konsekvens	Långsiktig konsekvens
Ska kontakta beroendecentrum<br><br><u>Strategi:</u><br><br>• Se till att vara utvilad vid tidpunkten du ska ringa	Ställer in	• Slippa ta en ny social kontakt<br>• Slipper den ansträngning det innebär att minska/sluta med alkohol<br>• Ångest för att inte ha följt planen	• Ökad risk för över 60 sjukdomar inklusive cancer<br>• Sämre omdöme, tankeförmåga, minne och reaktionsförmåga.<br>• Sämre sömn<br>• Ökad risk för att bli deprimerad<br>• Risken för olyckor
Umgås med vän som du brukade dricka med<br><br><u>Strategi:</u><br>• Fråga innan ni ses om vännen kan undvikas att ta dricka när du är med.<br>• Träna innan vad du ska göra när frestelsen uppstår<br>• Träffa inte vännen om hen inte respekterar att avstå alkohol när du är med	Dicker	• Kul/avkopplande<br>• Samhörighet<br>• Ångest	• Ökad risk för över 60 sjukdomar inklusive cancer<br>• Sämre omdöme, tankeförmåga, minne och reaktionsförmåga.<br>• Sämre sömn<br>• Ökad risk för att bli deprimerad<br>• Risken för olyckor
Har varit drogfri ett tag men blir sugen<br><br><u>Strategi:</u>	Bestämmer dig för att göra ett undantag och dricka	• Spännande/kul/ skönt<br>• Ångest för att inte ha följt planen	• Ökad risk för fler undantag/återfall

Etikprövningsmyndigheten
2022-02920-01-290879
2022-07-07

• Går det att undvika miljön som utlöste suget?<br>Träna innan på vad du ska göra			

Önskat beteende

**Situation/ stimuli**	**Beteende**	**Kortsiktig konsekvens**	**Långsiktig konsekvens**
Ska kontakta beroendecentrum	Ringer/tar kontakt<br><br><u>Underlättande strategier:</u><br>• Träna på att ringa<br>• Var utvilad när du ska ringa<br>• Bestäm en bra plats i god tid	• Först ångest eller ansträngning<br><br>• Sedan stolt	• Minskar risken för över 60 sjukdomar inklusive cancer<br>• Bättre omdöme, tankeförmåga, minne och reaktionsförmåga.<br>• Bättre sömn<br>• Minskad risk för depression<br>• Minskad risk för olyckor
Umgås med vän som tar droger	• Tackar nej<br>• Byte till miljö/vän där suget känns mindre<br><br><u>Underlättande strategier:</u><br>• Träna på att tacka nej innan<br>• Börja med alternativa aktiviteter/miljöer att vistas i	• Abstinens<br>• Känslan av att missa något kul/belönande<br>• Sedan stolt över att du följt planen	• Ökad förmåga att stå emot<br>• Minskar risken för över 60 sjukdomar inklusive cancer<br>• Bättre omdöme, tankeförmåga, minne och reaktionsförmåga.<br>• Bättre sömn<br>• Minskar risk för depression<br>• Minskar risk för olyckor
Har varit drogfri ett tag men blir sugen	• Står emot suget<br>• Ringer en stödlinje/en vän och ber om stöd<br>• Gör en annan aktivitet som du tycker är spännande<br><br><u>Underlättande strategier:</u><br>• Lägg in numret till stödlinjen innan.<br>• Testa att ringa stödlinjen innan<br>• Börja med den	• Känslan av att missa något kul/belönande<br>• Sedan stolt över att du följt planen	• Ökad förmåga att stå emot<br>• Minskad risk för<br>o Nedstämd<br>o Ångest<br>o Sämre sömn<br>o Passivitet<br>o Sämre inlärningsförmåga<br>o Sämre minne<br>o Sämre koncentration<br>o Personlighetsförändringar<br>o Utmattning

Etikprövningsmyndigheten
2022-02920-01-290879
2022-07-07

	alternativa aktiviteten redan nu		

Fysisk aktivitet

Oönskat beteende

Situation/stimuli	Beteende	Kortsiktig konsekvens	Långsiktig konsekvens
Du är på bussen och hade tänkt gå av en hållplats tidigare för att få motion<br><br><u>Strategi:</u><br><br>• Gör det med en vän så att ni kan peppa varandra<br>• Börja med denna rutin där du tycker sträckan känns lätt att genomföra<br>• Välj en sträcka där du är nyfiken på att titta på något<br>• Välj en sträcka där du kan passa på att handla något du behöver<br>• Va ute i god tid<br>• Sätt dig nära utgången på bussen	Du åker vidare hela vägen	• Du slipper ansträngningen det innebär att gå/rulla<br>• Du får mer tid över	Sämre kondition vilket leder till<br>• Sämre fysisk hälsa<br>• Ökad risk för sjukdomar
Du känner att du borde gå till gymmet/träna<br><br><u>Strategi:</u><br>• Lägg fram träningskläder<br>• Börja med enklare pass/motion<br>• Bestäm en dag och tid du alltid går	Du skjuter på det	• Du slipper fysisk ansträngning<br>• Du slipper träna med andra<br>• Kan göra annat som är mer belönande i stunden	• Ökad risk att du skjuter upp träningen i framtiden<br>• Sämre fysisk hälsa<br><br>• Ökad risk för sjukdomar

Etikprövningsmyndigheten
2022-02920-01-290879
2022-07-07

Du har suttit ned i en timme utan att röra på dig<br><br>Strategi:<br>• Sätt en klocka som påminner dig att resa på dig varje halvtimme	Du sitter kvar	• Du slipper ansträngningen det innebär att avbryta stillasittandet<br>• Du kan fortsätta med det du håller på med (jobb/film/läsning)	• Sämre fysisk hälsa<br>• Ökad risk för sjukdomar

Önskat beteende

**Situation/stimuli**	**Beteende**	**Kortsiktig konsekvens**	**Långsiktig konsekvens**
Du är på bussen och hade tänkt gå av en hållplats tidigare för att få motion	Du går av och tar dig på egen hand dit du ska<br><br>Underlättande strategier:<br>• Gör det med en vän så att ni kan peppa varandra<br>• Sätt dig nära utgången på bussen<br>• Ha kläder efter väder<br>• Lyssna på något i hörlurar	• Viss ansträngning av rörelsen<br>• Det tar längre tid<br>• Det känns härligt när du är klar	Du har motverkat risk för<br>• fysisk ohälsa<br>• psykisk ohälsa
Du känner att du borde gå till gymmet/träna	Du går och tränar<br><br>Underlättande strategier:<br><br>• Se till att äta ordentligt innan så att du orkar ta ut dig<br>• Träna med en vän<br>• Be att få hjälp med instruktioner för hur man gör	• Fysisk ansträngande<br>• Du upplever att det är jobbigt socialt	Du har motverkat risk för<br>• fysisk ohälsa<br>• psykisk ohälsa
Du har suttit ned i en timme utan att röra på dig	Du avbryter och utför en hushållssyssla/rör på dig 5 minuter	• Du tappar lite fokus kring det du häll på med	• Bättre koncentration i längden<br>• Mindre risk för fysisk ohälsa

Etikprövningsmyndigheten
2022-02920-01-290879
2022-07-07

	<u>Underlättande strategier:</u><br><br>• Ställ fram dammsugaren<br>• Sätt en klocka för påminnelse		

Droger

Kom ihåg att samverka med den som ger eventuell insats externt och fokusera på det som kompletterar deras verksamhet:

- Att det finns en fortsatt kontakt.
- Att andra levnadsvanor som stöttar minskningen av droger fungerar.

Oönskat beteende

**Situation/stimuli**	**Beteende**	**Kortsiktig konsekvens**	**Långsiktig konsekvens**
Ska kontakta beroendecentrum<br><br><u>Strategi:</u><br><br>• Se till att vara utvilad vid tidpunkten du ska ringa	Ställer in	• Slippa ta en ny social kontakt<br>• Slipper den ansträngning det innebär att minska/sluta med droger<br>• Ångest för att inte ha följt planen	• Nedstämd<br>• Ångest<br>• Sämre sömn<br>• Passivitet<br>• Sämre inlärningsförmåga<br>• Sämre minne<br>• Sämre koncentration<br>• Personlighetsförändringar<br>• Utmattning
Umgås med vän som tar droger<br><br><u>Strategi:</u><br>• Fråga innan ni ses om vännen kan undvikas att ta droger när du är med.<br>• Träna innan vad du ska göra när frestelsen uppstår<br>• Träffa inte vännen om hen inte respekterar	Tar droger	• Kul/avkopplande<br>• Samhörighet<br>• Ångest efter	• Nedstämd<br>• Ångest<br>• Sämre sömn<br>• Passivitet<br>• Sämre inlärningsförmåga<br>• Sämre minne<br>• Sämre koncentration<br>• Personlighetsförändringar<br>• Utmattning

Etikprövningsmyndigheten
2022-02920-01-290879
2022-07-07

att avstå droger när du är med			
Har varit drogfri ett tag men blir sugen<br><br><u>Strategi:</u><br>• Går det att undvika miljön som utlöste suget?<br>• Träna innan på vad du ska göra	Bestämmer dig för att göra ett undantag och ta droger	• Spännande/kul/ skönt<br>• Ångest för att inte ha följt planen	• Ökad risk för fler undantag/återfall

Önskat beteende

**Situation/ stimuli**	**Beteende**	**Kortsiktig konsekvens**	**Långsiktig konsekvens**
Ska kontakta beroendecentrum	Ringer/tar kontakt<br><br><u>Underlättande strategier:</u><br>• Träna på att ringa<br>• Va utvilad när du ska ringa<br>• Bestäm en bra plats i god tid	• Först ångest, ansträngning<br>• Sedan stolt	Minskad risk för:<br>• Nedstämd<br>• Ångest<br>• Sämre sömn<br>• Passivitet<br>• Sämre inlärningsförmåga<br>• Sämre minne<br>• Sämre koncentration<br>• Personlighetsförändringar<br>• Utmattning
Umgås med vän som tar droger	• Tackar nej<br>• Byter till miljö/vän där suget känns mindre<br><br><u>Underlättande strategier:</u><br>• Träna på att tacka nej innan<br>• Fundera på alternativa aktiviteter/miljöer att vistas i	• Abstinens<br>• Känslan av att missa något kul/belönande<br>• Sedan stolt över att du följt planen	• Ökad förmåga att stå emot<br>• Minskad risk för<br>○ Nedstämd<br>○ Ångest<br>○ Sämre sömn<br>○ Passivitet<br>○ Sämre inlärningsförmåga<br>○ Sämre minne<br>○ Sämre koncentration<br>○ Personlighetsförändringar<br>○ Utmattning
Har varit drogfri ett tag men blir sugen	• Står emot suget<br>• Ringer en stödlinje/en vän och ber om stöd<br>• Gör en annan aktivitet som du tycker är	• Känslan av att missa något kul/belönande<br>• Sedan stolt över att du	• Ökad förmåga att stå emot<br>• Minskad risk för<br>○ Nedstämdhet<br>○ Ångest<br>○ Sämre sömn<br>○ Passivitet

Etikprövningsmyndigheten
2022-02920-01-290879
2022-07-07

	spännande<br><br><u>Underlättande strategier:</u><br>• Lägg in numret till stödlinjen innan<br>• Börja med den alternativa aktiviteten redan nu	följt planen	○ Sämre inlärningsförmåga<br>○ Sämre minne<br>○ Sämre koncentration<br>○ Personlighetsförändringar<br>○ Psykossjukdom<br>○ Utmattning

Skärmtid

Oönskat beteende

**Situation/stimuli**	**Beteende**	**Kortsiktig konsekvens**	**Långsiktig konsekvens**
Spelar/använder sociala medier under många timmar<br><br><u>Strategi:</u><br>• Bestäm innan hur länge du ska spela<br>• Bestäm vad du ska göra för rolig sak när du slutar spela	Fortsätter	• Kul<br>• Slipper ha tråkigt<br>• Verklighetsflykt	• Slarvar med andra levnadsvanor vilket leder till sämre hälsa
Du har något som du borde göra<br><br><u>Strategi:</u><br>• Börja med det du ”borde göra” men gör något mycket enkelt som att: lägga fram det du behöver<br>• Lägg skärm i ett annat rum	Använder skärm i stället	• Slipper ta tag i något som du borde göra<br>• Ångest för att du inte följt din plan	• Hamnar efter med saker du borde göra
Du blir sugen på att spela på nätcasino<br><br><u>Strategi:</u><br>• Bestäm innan mellan vilka	Du börjar spela utan någon plan för hur länge du ska spela eller hur mycket du får förlora/vinna	• Spännande<br>• Kul	• Du förlorar pengar<br>• Du riskerar att hamna i ett spelberoende

Etikprövningsmyndigheten
2022-02920-01-290879
2022-07-07

• tider du får spela och hur mycket pengar du får spendera<br>• Bestäm något kul du ska göra när du är klar med ditt spelande			

Önskat beteende

**Situation/stimuli**	**Beteende**	**Kortsiktig konsekvens**	**Långsiktig konsekvens**
Spelar/använder sociala medier under många timmar	Avbryter för annan aktivitet<br><br><u>Underlättande strategier:</u><br>• Sätt klockan så att den ringer när du ska sluta<br>• Bestäm något roligt när du avbryter<br>• Börja med ett nytt intresse som du kan växla med skärmtiden	• Ansträngning att ta tag i något som är jobbigare<br>• Stolt för att du följt din plan	• Se positiva konsekvenser av:<br>• Sömn<br>Rörelse<br>• Rutiner för mat<br>• Sociala relationer
Du har något du borde göra	Ägnar dig åt det du borde göra<br><br><u>Underlättande strategier:</u><br>• Börja med något mycket enkelt (det du tycker är minst jobbigt)<br>• Lägg skärmar i ett annat rum<br>• Berätta för en anhörig att du känner dig stolt som tog tag i det jobbiga	• Ansträngning att ta tag i något som är jobbigare<br>• Stolt för att du följt din plan	• Du hinner göra fler saker som du planerat
Du blir sugen på att spela på nätcasino	• Du avstår från att spela<br><br><u>Underlättande</u>	• Jobbigt att stå emot spelsuget<br>• Nöjd för att du följer din plan	• Minskad risk att förlora pengar<br>• Minskad risk för spelberoende

Etikprövningsmyndigheten
2022-02920-01-290879
2022-07-07

	strategier:<br><br>• Du gör en annan aktivitet som du gillar<br>• Ringer stödlinje/vän som kan stötta dig		

Sömn

Oönskat beteende

**Situation/stimuli**	**Beteende**	**Kortsiktig konsekvens**	**Långsiktig konsekvens**
Det är en timme kvar till din planerade liggtid<br><br>Strategi:<br><br>• Du ser till att ha motionerat tidigare under dagen<br>• Du undviker att sova middag<br>• Du släcker ned, och ägnar dig åt lugna aktiviteter	Börjar med något engagerande och fortsätter längre än din planerade läggtid	Du har kul/känner dig tillfredsställd	• Trött dagen efter<br>• Fortsatt dålig sömn på grund av bristande rutiner
Du har planerat in motionspass som ska bidra till att du sover bättre och det börjar bli dags att börja<br><br>Strategi:<br>• Ta på dig träningskläderna<br>• Se till att ha ett enklare pass du kan genomföra dagar du är extra trött<br>• Planera in passet på en tid då du brukar ha energi även dagar du fått för lite sömn	Du skippar passet för att du är för trött	Du slipper ansträngningen	Fortsatt dålig sömn

Etikprövningsmyndigheten
2022-02920-01-290879
2022-07-07

Du funderar på att göra något av de enkla råden du lärt dig kan påverka sömnen<br><br><u>Strategi:</u><br>• Planera in i förväg att testa ”rådet” tillsammans med någon<br>• Börja med något av det du tycker är enkelt att genomföra	Det blir inte av	Du slipper ansträngningen det innebär att planera och genomföra<br>• lugna aktiviteter före sänggående<br>• Undvika koffein, nikotin och alkohol<br>• Minskande på skärmtid på kvällen<br>• Avslappningsövningar	Fortsatt dålig sömn

Önskat beteende

**Situation/stimuli**	**Beteende**	**Kortsiktig konsekvens**	**Långsiktig konsekvens**
Det är en timme kvar till din planerade liggtid	Du släcker ned, och ägnar dig åt lugna aktiviteter<br><br><u>Underlättande strategier:</u><br>• Bestäm innan vilka de lugna aktiviteterna ska vara.	• Tråkigt, brist på stimulans<br>• Lugn, avslappning	Ökade chanser till bättre sömn
Du har planerat in motionspass som ska bidra till att du sover bättre och det börjar bli dags att börja	Du genomför passet<br><br><u>Underlättande strategier:</u><br>• Ät i god innan passet så att du orkar<br>• Genomför passet med en kompis	• Fysiskt och mentalt ansträngande<br>• Skönt efteråt	• Ökade chanser till bättre sömn<br>• Minskad risk för sjukdomar efter bättre sömn och även fysisk aktivitet
Du funderar på att göra något av de enkla råden du lärt dig kan påverka sömnen	Du börjar med ett av råden<br><br><u>Underlättande strategier:</u><br>• Du genomför det i samband med en annan rutin som du redan har som fungerar (till exempel efter att du borstat tänderna	Det är ansträngande att försöka och du kanske känner att du missar något i stunden	Ökade chanser till bättre sömn

Etikprövningsmyndigheten
2022-02920-01-290879
2022-07-07

	går du runt och släcker ned ljuset.		

Intressen

Oönskat beteende

Situation/stimuli	Beteende	Kortsiktig konsekvens	Långsiktig konsekvens
Du har, som ofta, tid över och funderar på vad du ska göra<br><br><u>Strategi:</u><br>• Lägg mobilen i ett annat rum<br>• Skriv en lista på saker du är nyfiken på att göra<br>• Skriv även en lista på vad som är första steget för att börja med önskade aktiviteter	Du fastnar i att skrolla på mobilen	Du roas av innehållet i mobilen	• Stress<br>• Sömnproblem<br>• Nedstämdhet<br>• Försämrad fysisk hälsa<br>• Ensamhet
Du har jobbat hela dagen och det är dags att åka hem<br><br><u>Strategi:</u><br><br>• Bestäm när och hur du ska börja med en aktivitet efter jobbet<br>• Förbered för aktiviteten redan på morgonen så det blir enklare att börja	Du åker hem men fortsätter att jobba	• Du behöver inte styra upp en annan aktivitet	• Stress<br>• Sömnproblem<br>• Nedstämdhet<br>• Försämrad fysisk hälsa<br>• Ensamhet
Du har bestämt dig för att skaffa dig en sysselsättning men vet inte hur det ska gå till	Du känner att det inte finns något som passar dig och ger upp	• Uppgivenhet	• Stress<br>• Sömnproblem<br>• Nedstämdhet<br>• Försämrad fysisk

Etikprövningsmyndigheten
2022-02920-01-290879
2022-07-07

<u>Strategi:</u><br><br>• Ta kontakt med intresseförening och fråga om tips<br>• Titta på klipp på youtube för att se hur man gör. Gärna klipp med människor som liknar dig			hälsa<br>• Ensamhet

Önskat beteende

**Situation/stimuli**	**Beteende**	**Kortsiktig konsekvens**	**Långsiktig konsekvens**
Du har, som ofta, tid över och funderar på vad du ska göra	• Du ringer en vän och ni går på museum<br>• Du går på Quiz anordnat av en intresseförening du fått tips om<br><br><u>Underlättande strategier:</u><br><br>• Se till att vara utvilad så att det blir en roligare upplevelse	• Du har det roligt/trevligt<br>• Du träffar andra	• Motverkar stress<br>• förbättra din sömn<br>• göra dig lyckligare<br>• förbättra din fysiska hälsa (exempelvis BMI, blodtryck)<br><br>• ge dig en känsla av tillhörighet/delaktighet
Du har jobbat hela dagen och det är dags att åka hem	Du åker hem och tappar upp ett varmt bad och löser ett korsord<br><br><u>Underlättande strategier:</u><br><br>• Lägg bort jobbdator och mobil	Avkopplande	• Motverkar stress<br>• förbättra din sömn<br>• göra dig lyckligare<br>• förbättra din fysiska hälsa (exempelvis BMI, blodtryck)
Du har bestämt dig för att skaffa dig en sysselsättning men vet inte hur det ska gå till	Du tar kontakt med intresseförening och ser vilka aktiviteter de har	• Nöjd över att du tagit tag i något du vill ändra på<br>• Förväntansfull	• Motverkar stress genom återhämtning<br>• Förbättra din sömn<br>• Göra dig lyckligare<br>• Förbättra din fysiska

Etikprövningsmyndigheten
2022-02920-01-290879
2022-07-07

	Underlättande strategier:<br><br>• Ta fram papper och penna<br>• Fråga om de kan mejla dig mer information		hälsa

Sexuell hälsa

Oönskat beteende

**Situation/stimuli**	**Beteende**	**Kortsiktig konsekvens**	**Långsiktig konsekvens**
Du skulle vilja veta mer om hur du kan njuta av sex<br><br>Strategi:<br><br>• Du surfar in på länkarna du fått tips om	Du tycker det känns pinsamt så du avstår att ta tag i saken	Du slipper pinsamheten	Du missar:<br>• att njuta och må bra i stunden<br>• minska stress<br>• förbättrad sömn<br>• förbättrat immunförsvar<br>• förbättrad din fysiska hälsa (exempelvis hjärtat)<br>• förbättrat ditt humör<br>• minskad smärta<br>• öka känslan av närhet om du har det men någon annan
Du känner dig osäker kring vad som är rätt och fel gällande det sex du har<br><br>Strategi:<br><br>• Du bokar in ett möte med någon av de kontakter du fått.	Du fortsätter ändå	Du slipper ta tag i det jobbiga ämnet	Sex ska kännas säkert för dig och den du har sex med. Genom att inte be om stöd tar du en risk att göra dig själv eller någon annan ledsen.
Du har smärta i samband med sex<br><br>Strategi:	Du gör inget åt saken	Du slipper känna dig annorlunda i mötet med vårdpersonal	Du går miste om eventuell hjälp som finns att få

Etikprövningsmyndigheten
2022-02920-01-290879
2022-07-07

• Du bokar in ett möte med någon av de kontakter du fått via stöd- och behandlingsplattformen<br>• Skriv in numret till rådgivaren och bestäm ett klockslag när du ska ringa			

Önskat beteende

Situation/stimuli	Beteende	Kortsiktig konsekvens	Långsiktig konsekvens
Du skulle vilja veta mer om hur du kan njuta av sex	Du surfar in på någon av länkarna som du fått från Habiliteringen gällande njutning<br><br><u>Underlättande strategier:</u><br><br>• Förbered dig på att alla tips inte kommer passa dig.<br>• Se till att hitta en tid och plats där du får vara i fred.	• Det känns lite pinsamt<br>• Spänning	Du hittar sätt att njuta som i sin tur:<br>• minskar stress<br>• förbättrar sömn<br>• förbättrar immunförsvar<br>• förbättrar din fysiska hälsa<br>• förbättrar ditt humör<br>• minskar smärta<br>• öka känslan av närhet om du har det men någon annan
Du känner dig osäker kring vad som är rätt och fel gällande det sex du har	• Du avslutar/pausar det sex du osäker kring<br><br><u>Underlättande strategier:</u><br><br>• Du frågar vårdpersonal om vart du kan vända dig för att få svar på frågor om sexuell hälsa<br><br>• Du surfar in på någon av länkarna som du	• Det känns jobbigt och lite läskigt	Du lär dig om vad tryggt och säkert sex är och kan på så sätt minska din ångest samt njuta fullt ut

Etikprövningsmyndigheten
2022-02920-01-290879
2022-07-07

	fått från Habiliteringen gällande trygghet		
Du har smärta i samband med sex	Du tar kontakt med de vårdaktörer som din kontakt rekommenderar att du vänder dig till<br><br><u>Underlättande strategier:</u><br><br>• Se till att hitta en tid och plats där du får vara i fred	• Pinsamhet<br>• En känsla att något är fel/annorlunda	Du får hjälp att behandla smärtan/hitta alternativa sätt som du kan njuta på

Etikprövningsmyndigheten
2022-02920-01-290879
2022-07-07

Vänner/Familj

Oönskat beteende

Situation/stimuli	Beteende	Kortsiktig konsekvens	Långsiktig konsekvens
Du vill träffa fler vänner och har möjlighet att vara med på en fika<br><br><u>Strategi:</u><br>• Försök förstå vad det jobbiga är och se om du kan anpassa fikan genom att exempelvis sätta dig bredvid någon du känner	Du går inte på fikan	Slipper ansträngningen det kan innebära för sig att umgås	• Fortsatt ensamhet<br>• Du missar de hälsosamma effekter som sociala relationer kan ge
Du vill ha någon du kan lita på<br><br><u>Strategi:</u><br>• Börja med att berätta något som inte är så jätteprivat	Du gör inget för att skapa en sådan relation	Slipper utmaningen och det lite läskiga det innebär att berätta personliga saker för någon annan	• Ensamhet<br>• Du missar de hälsosamma effekter som sociala relationer kan ge

för den person du litar mest på idag<br>• Fråga någon som du känner väl om du skulle kunna få prata om något som är lite privat			
Du känner dig ensam och trött<br><br><u>Strategi:</u><br>• Försök att vara utvilad när du träffar bekanta. Då ökar chansen att du får ut mer av att ses<br>• Ses en liten stund om du inte orkar så länge	Du fastnar framför skärmen istället för att träffa någon	Slipper ansträngningen det kan innebära att hitta vem du ska träffa och vad ni ska göra.	• Fortsatt ensamhet<br>• Du får inte den energi som att träffa vänner ger<br>• Fortsatt trötthet

Etikprövningsmyndigheten
2022-02920-01-290879
2022-07-07

Önskat beteende

**Situation/stimuli**	**Beteende**	**Kortsiktig konsekvens**	**Långsiktig konsekvens**
Du vill träffa fler vänner och har möjlighet att vara med på en fika	Du går på fikan<br><br><u>Underlättande strategier:</u><br><br>• Planera in något du vill fråga. Exempelvis vad de som deltar ska göra i helgen eller om någon har ett bra tips på någon bra serie/film/bok?	• Trött efter att ha behövt anstränga dig för att förstå sociala regler<br>• Spännande/kul	• Förutsatt att du får tid till återhämtning känns fikan mindre ansträngande<br>• Du har möjlighet att träffa vänner<br>• Mindre ensamhet
Du vill ha någon du kan lita på	Du frågar någon du har känt länge om du kan berätta personliga saker utan att hen berättar det för andra?<br><br><u>Underlättande strategier:</u>	• Utelämnande känsla/läskigt att ge någon annan det förtroendet<br>• Skönt att känna att någon finns där som kan lyssna	• Mindre ensamhet<br>• Trygghet<br>• En fördjupad vänskaps<br>• Fysiska och psykiska hälsofördelar

	• Välj ett tillfälle när ni är själva och har tid<br>• Om någon berättar något för dig som är hemligt bör du inte berätta det för andra		
Du känner dig ensam och trött	Du hör av dig till en vän och ni går på en promenad<br><br>Underlättande strategier:<br>Fundera på om du har någon som bor/jobbar nära som du kan gå med på lunchen	• Ansträngande att inte ligga kvar på soffan<br>• Skönt att få se en vän och prata och promenera<br>• Mer energi	• Mindre ensamhet<br>• Fysiska och psykiska hälsofördelar<br>• Mer energi

Etikprövningsmyndigheten
2022-02920-01-290879
2022-07-07

Utforska motivation – bilaga 3

Hjälp deltagaren resonera sig fram till ett svar och använd de tre frågorna om motivation nedan.

Välj det område där deltagaren har störst chans att lyckas (där motivationen är högst).

Välj gärna ett område som du tror kan ha positiv inverkan på annan pågående insats.

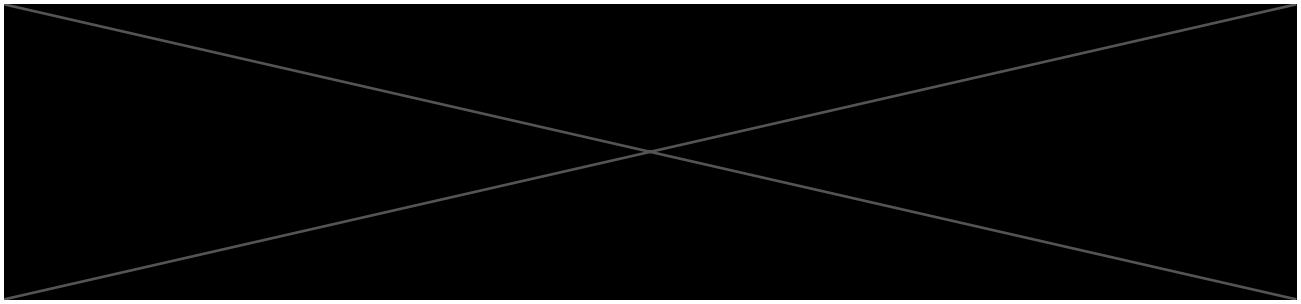

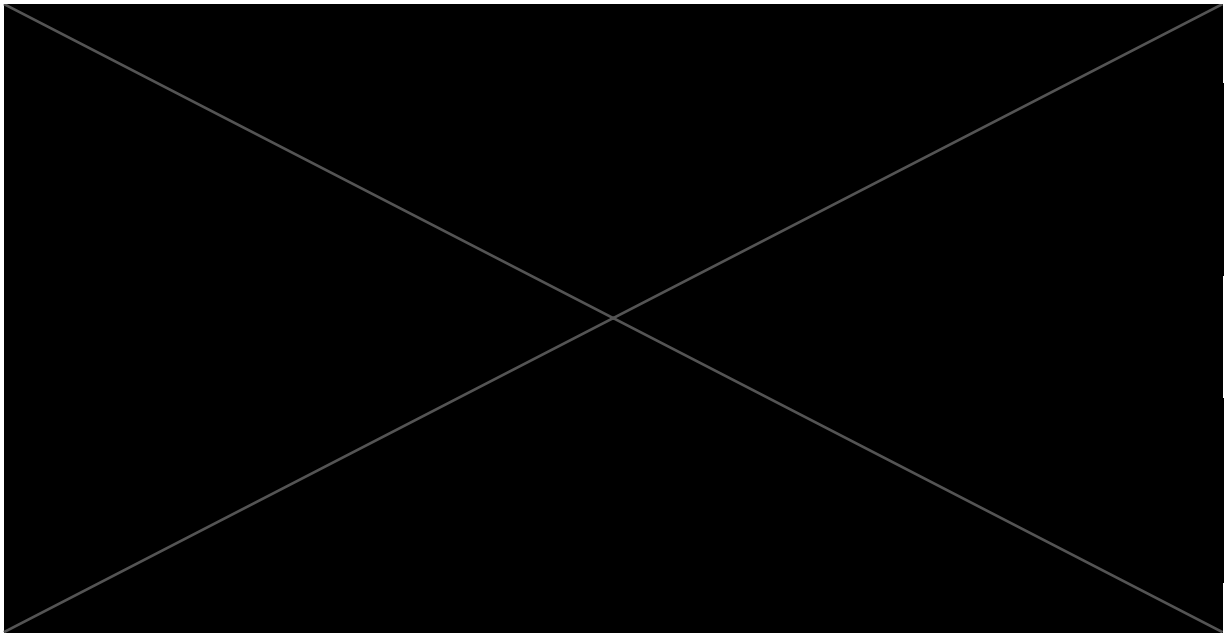

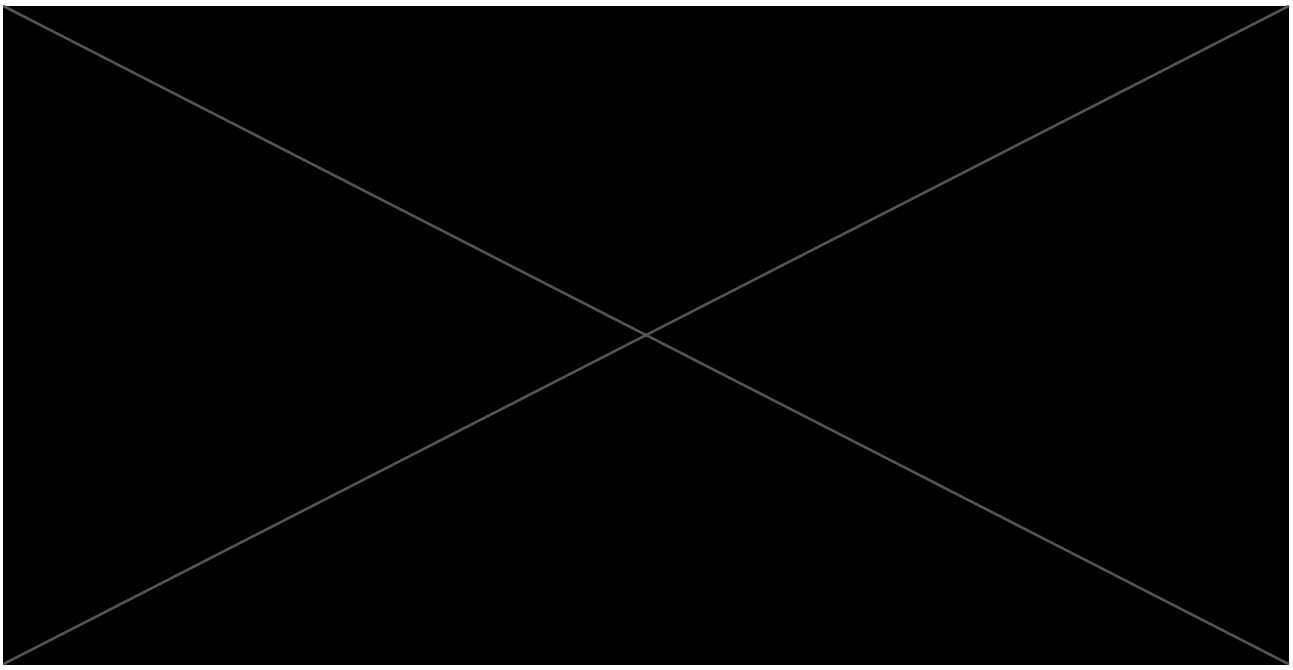

Etikprövningsmyndigheten
2022-02920-01-290879
2022-07-07

- **Vad skulle krävas för en högre siffra?** Denna följdfråga kan ge svar om eventuella hinder – något ni kan arbeta vidare med.

- **Vad skulle vara till hjälp för dig för att du skulle kunna säga en högre siffra?** Denna fråga kan identifiera vilket stöd vi eller någon annan kan ge.

Vill deltagaren inte prata vidare om någon speciell levnadsvana trots problem så tänk att du gjort vad du kunnat och notera detta i journalen. Lyft frågan om levnadsvanor vid ett senare tillfälle.

Etikprövningsmyndigheten
2022-02920-01-290879
2022-07-07

Lev-projektet: Kartläggning, bedömning och intervention av hälsorelaterade levnadsvanor hos vuxna med och utan funktionsnedsättning.

Information till behandlare

Vi vill fråga dig om du vill delta i ett forskningsprojekt. I det här dokumentet får du information om projektet och om vad det innebär för dig som behandlare att delta i Lev – genomförbarhetsstudien. Om du vill delta efter att ha läst igenom informationen, skriver du under samtycket längst bak och Douglas Sjöwall som är projektledare för studien.

Vad är det för ett projekt och varför vill vi att du ska delta?

Enligt Världshälsoorganisationen (WHO) kan vi må bättre och leva längre om vi har hälsosammare levnadsvanor. Hälso- och sjukvård behöver bli bättre på att ge stöd och insatser som syftar till hälsosammare levnadsvanor hos personer med någon form av funktionsnedsättning. I det här projektet utvärderar vi en ny insats (Lev) som har som syfte att förbättra levnadsvanor. Lev har utvecklats i ett samarbete mellan forskare, aktörer inom hälso- och sjukvård samt representanter för olika funktionsnedsättningar.

Vi söker dig som har en hälso- och sjukvårdsutbildning och som träffar följande patientgrupper som har någon form av funktionsnedsättning eller diagnos (exempelvis autism, ADHD, intellektuell funktionsnedsättning, förvärvad hjärnskada, rörelsenedsättning, dövblindhet, dövhet),

Forskningshuvudman för projektet är Habilitering & Hälsa, Region Stockholm. Med forskningshuvudman menas den organisation som är ansvarig för projektet. Ansökan är godkänd av Etikprövningsmyndigheten, diarienummer för prövningen hos Etikprövningsmyndigheten är ange diarienummer

Etikprövningsmyndigheten
2022-02920-01-305699
2022-09-09

Hur går projektet till?

Att delta i genomförbargetsstudien innebär att du genomgår en utbildning på distans vid 5 tillfällen (tid ca 10.5h). På utbildningen lär du dig Lev. Lev består av följande moment:

- **Mätning:** En utvärdering patientens levnadsvanor
- **Insatsen (Lev):**
    - Fas 1: Samtal om levnadsvanor, målsättning + eventuellt stöd från annan aktör inom hälso- och sjukvård.
    - Fas 2: Fördjupad analys som leder till individuella strategier och stöd för hur du ska nå dina mål.
    - Fas 3: Avslut och ny utvärdering av levnadsvanor, målsättning och ifyllnad av självskattningsformulär. Plan för eget arbete.

De 3 faserna i Lev ges under ca 2-6 månader parallellt med din utbildning. Förutom att du administrerar skattningar till patienten så fyller även du som behandlare i skattningar före under och efter deltagande i insatsen. Skattningarna inkluderar frågor om vad du tycker om behandlingen och vilken profession du har. Förutom skattningarna kommer vi analysera hur

olika vårdprofessioner fyller i den funktionella analysen i fas 2. Vi är inte intresserade att veta hur just du svarat utan om det är någon skillnad på gruppnivå mellan olika professioner. Svaren på dessa frågor kan hjälpa oss avgöra om vi behöver utveckla/förtydliga detta moment.

Möjliga följder och risker med att delta i projektet

Riskerna är låga. Att administrera och själv besvara forskningsfrågorna kommer ta viss tid i anspråk och det är viktigt att din medverkan är förankrad med din chef så att du får avsätta tid för det för att undvika stress. Förhoppningsvis leder din medverkan till att du får nya kunskaper kring hur du kan hjälpa andra till hälsosammare levnadsvanor.

Vad händer med mina uppgifter?

Projektet kommer att samla in och registrera information om dig.

Svaren förvaras under datainsamlingen inlåsta i ett journalskåp. Svaren på frågorna läggs sedan in i en datafil. I stället för att lägga in ditt namn används en kod som en säkerhetsåtgärd för att skydda din identitet. Kodnyckeln, som kan koppla ihop din kod med ditt namn, förvaras separerad från databasen på Habilitering & Hälsa, Region Stockholm. Datafilen förvaras inlåst på Karolinska Institutet och på Habilitering & Hälsa, Region Stockholm. Ingen obehörig kommer att få ta del av dina svar.

Behandlingen av dina personuppgifter är nödvändigt för att utföra forskning som är av allmänt intresse (GDPR, Art 6, p. 1e). Ansvarig för dina personuppgifter är Habilitering & Hälsa, Regions Stockholm. Enligt EU:s dataskyddsförordning har du rätt att kostnadsfritt få ta del av de uppgifter om dig som hanteras i projektet, och vid behov få eventuella fel rättade. Du kan också begära att uppgifter om dig raderas samt att behandlingen av dina personuppgifter begränsas. Rätten till radering och till begränsning av behandling av personuppgifter gäller dock inte när uppgifterna är nödvändiga för den aktuella forskningen. Data sparas i 10 år. Om du vill ta del av uppgifterna ska du kontakta Douglas Sjöwall Habilitering & Hälsa, Box 454 36, 104 31 Stockholm 08-123 350 10. Dataskyddsombud Camilla Heise Löwgren nås på camilla.heise-lowgren@regionstockholm.se. Om du är missnöjd med hur dina personuppgifter behandlas har du rätt att ge in klagomål till Integritetsskyddsmyndigheten, som är tillsynsmyndighet.

Hur får jag information om resultatet av projektet?

Du behöver inte ta del av dina eller studiens resultat. Om du vill ta del av resultatet av hela projektet hänvisar vi dig till de vetenskapliga artiklar som vi kommer sammanställa. Det kan dröja några år innan studien publiceras. Kontakta projektansvarig (Douglas Sjöwall) om du har frågor gällande resultaten från projektet.

Etikprövningsmyndigheten
2022-02920-01-305699
2022-09-09

Lev-projektet: Kartläggning, bedömning och intervention av hälsorelaterade levnadsvanor hos vuxna med och utan funktionsnedsättning.

Försäkring och ersättning

Ingen extra försäkring finns kopplad till projektet utöver den eventuella försäkring du har via din arbetsplats.

Ingen ersättning utgår för deltagande i projektet.

Deltagandet är frivilligt

Ditt deltagande är frivilligt och du kan när som helst välja att avbryta deltagandet. Om du väljer att inte delta eller vill avbryta ditt deltagande behöver du inte uppge varför, och det kommer inte heller att påverka din framtida position på din arbetsplats.

Ansvariga för projektet

Projektledare för projektet är Douglas Sjöwall Habilitering & Hälsa, Box 454 36, 104 31 Stockholm 08-123 350 10. Douglas.sjowall@regionstockholm.se

Etikprövningsmyndigheten
2022-02920-01-305699
2022-09-09

Avgiftsavisering

Etikprövningsmyndigheten har tagit emot din ansökan med titel Lev-projektet: Kartläggning, bedömning och intervention av hälsorelaterade levnadsvanor hos vuxna med och utan funktionsnedsättning. om etikprövning. Ansökan har diarienummer 2022-02920-01 vilket alltid ska anges i framtida kontakter i ärendet.

Avgiften för ansökan, som är 5000 kronor, ska omgående betalas in enligt nedan:

- Inbetalning sker till bankgironummer 406-1107
- Vid inbetalning ska OCR-nummer 2022029200138 anges som referens.
- Inga andra bokstäver eller siffror får anges i raden för referens.

Först när ärendet kompletterats enligt ovan kommer vi att påbörja handläggningen.

Etikprövningsmyndigheten
Telefon: 010 - 475 08 00
Webbplats: www.etikprovning.se

BESLUT

2022-06-22

Sökande forskningshuvudman
Region Stockholm

Forskare som genomför projektet
Stig Douglas Filip Sjöwall

Projekttitel
Lev-projektet: Kartläggning, bedömning och intervention av hälsorelaterade levnadsvanor hos vuxna med och utan funktionsnedsättning.

Uppgifter om ansökan
Ansökan inkom till Etikprövningsmyndigheten 2022-05-23 och blev valid 2022-06-02.

Etikprövningsmyndigheten beslutar enligt nedan.

BESLUT

Etikprövningsmyndigheten
2022-02920-01-286950
2022-06-29

Etikprövningsmyndigheten begär att sökanden kompletterar ansökan enligt följande:

Etikprövningsmyndigheten ska vid sin prövning bland annat väga de möjliga risker som forskningen kan medföra för forskningspersonerna (i form av exempelvis skada eller sjukdom, integritetsintrång, stress och inverkan på välbefinnande) mot den förväntade nyttan av forskningen. För att kunna göra en sådan bedömning behöver myndigheten klar och tydlig information om projektet och det planerade genomförandet. Etikprövningsmyndigheten vill därför se en utökad diskussion om eventuella risker för forskningspersonerna vid deltagande i framförallt interventionsstudien, t ex negativa känslor av misslyckande i samband med att deltagare inte når sina mål, och hur man avser att hantera dessa. Vidare är den bifogade annonsen utformad likt en forskningspersonsinformation och anger att forskningspersonerna samtycker till medverkan efter annonsen. Myndigheten vill se att forskningspersonerna samtycker till medverkan efter att de tillgodosett sig hela forskningspersonsinformationen.
Det behöver också utformas ett informationsbrev till de behandlare som medverkar i projektet.

I all information till forskningspersoner ska:
1. Hänvisningar till anonymitet och liknande begrepp strykas då dessa är missvisande eller felaktiga.
2. Den rättsliga grunden för inhämtande av personuppgifter anges (t.ex. "Behandlingen av dina personuppgifter är nödvändigt för att utföra forskning som är av allmänt intresse (GDPR, Art 6, p. 1e).").
3. Det anges hur länge data lagras.
4. Rätt personuppgiftsansvarig anges.
5. Stryk "endast personer som är involverade i databearbetningen har tillgång" och ersätt med "Ingen obehörig kommer att få ta del av dina svar".

ompletteringen ska ha kommit in till Etikprövningsmyndigheten senast den 2022-07-27.

Etikprövningsmyndigheten tar sedan upp ärendet till prövning vid ett kommande sammanträde. Om kompletteringen inte kommer in i tid kommer myndigheten att pröva ärendet utifrån det underlag som finns nu.

Kompletteringen görs i Ethix.

Beslut om komplettering innebär att ansökan låses upp för redigering. De begärda kompletteringarna görs direkt i respektive fält, systemet kommer markera upp vilka ändringar som gjorts. Ska kompletterande bilagor laddas upp görs detta under rubriken 'Av myndighetsbeslut begärda komplettering'. Markera ändringar i reviderade bilagor. Besvara kompletteringspunkterna och beskriv i avsett fält för att förtydliga vilka ändringar som gjorts. När kompletteringarna är genomförda ska ansökan signeras och skickas in på nytt.

På Etikprövningsmyndighetens vägnar

Johan Rosén
Ordförande

Etikprövningsmyndigheten
2022-02920-01-286950
2022-06-29

Beslutet har fattats av följande personer:

Ordförande
Johan Rosén

Ledamöter med vetenskaplig kompetens
Lena Almqvist (psykologi, barn- och ungdomsforskning, vetenskaplig sekreterare)
Önver Cetrez (religions- och kulturpsykologi, vetenskaplig sekreterare)
Oscar Pripp (Etnologi, antropologi, sociologi, föredragande)
Maria Eriksson Baaz (Statsvetenskap)
Minna Gräns (rättsvetenskap)
Maria Harder (Vårdvetenskap)
Niklas Jakobsson (Nationalekonomi)
Anna Lindström (språk och social interaktion)

Ledamöter som företräder allmänna intressen
Kenneth Johannesson
Håkan Krantz
Barbro Larsson
Pia Milton
Gert Ohlsson

Beslutet sänds till

Ansvarig forskare: Stig Douglas Filip Sjöwall

Forskningshuvudmannens företrädare: Joakim Lavesson

Begäran om administrativt tillägg

2022-07-08

Sökande forskningshuvudman
Region Stockholm

Forskare som genomför projektet
Stig Douglas Filip Sjöwall

Projekttitel
Lev-projektet: Kartläggning, bedömning och intervention av hälsorelaterade levnadsvanor hos vuxna med och utan funktionsnedsättning.

Uppgifter om ansökan
Ansökan inkom till Etikprövningsmyndigheten 2022-05-23.

Etikprövningsmyndigheten begär administrativt tillägg av din ansökan om etikprövning enligt följande:

Hej

Etikprövningsmyndigheten
2022-02920-01-291193
2022-07-08

Etikprövningsmyndigheten har tagit emot din ansökan om etikprövning med dnr 2022-02920-01 och titel "Lev-projektet: Kartläggning, bedömning och intervention av hälsorelaterade levnadsvanor hos vuxna med och utan funktionsnedsättning.". Ansökan har validerats och följande administrativa tillägg behöver göras:

1. Ni har inte markerat ändringarna i forskningspersonsinformationerna. Det gör lättast med annan färg. Har ni ändrat hela forskningspersonsinformationen förklara det under fliken av myndigheten begärt administrativt tillägg.

Logga in i Ethix och åtgärda det ovan beskrivna senast 2022-07-15. Ansvarig forskare måste signera när tillägget ska skickas in och när myndigheten mottagit det kommer handläggningen att fortsätta.

E-posta eventuella frågor till registrator@etikprovning.se.

Med vänlig hälsning

Malin Norrblom

Handläggare

Etikprövningsmyndigheten

Telefon: 010-475 08 00

Webbplats: www.etikprovning.se

Begäran sänds till

Ansvarig forskare: Stig Douglas Filip Sjöwall

Etikprövningsmyndigheten
2022-02920-01-291193
2022-07-08

BESLUT

2022-08-24

Sökande forskningshuvudman
Region Stockholm

Forskare som genomför projektet
Stig Douglas Filip Sjöwall

Projekttitel
Lev-projektet: Kartläggning, bedömning och intervention av hälsorelaterade levnadsvanor hos vuxna med och utan funktionsnedsättning.

Uppgifter om ansökan
Ansökan inkom till Etikprövningsmyndigheten 2022-05-23 och blev valid 2022-06-02. Ansökan är tidigare behandlad vid sammanträde 2022-06-22. Av myndigheten begärd komplettering enligt beslut inkom 2022-07-07.

Etikprövningsmyndigheten beslutar enligt nedan.

BESLUT

Etikprövningsmyndigheten begär att sökanden kompletterar ansökan enligt följande:

Ett informationsbrev till de behandlare som medverkar i projektet som forskningspersoner ska utformas.

Kompletteringen ska ha kommit in till Etikprövningsmyndigheten senast den 2022-09-28.

Etikprövningsmyndigheten ger ordföranden i uppdrag att fatta beslut i ärendet när kompletteringen har kommit in. Om kompletteringen inte inkommer i tid kommer Etikprövningsmyndigheten att pröva ärendet utifrån det underlag som finns nu.

Kompletteringen görs i Ethix.

Beslut om komplettering innebär att ansökan låses upp för redigering. De begärda kompletteringarna görs direkt i respektive fält, systemet kommer markera upp vilka ändringar som gjorts. Ska kompletterande bilagor laddas upp görs detta under rubriken 'Av myndighetsbeslut begärda komplettering'. Markera ändringar i reviderade bilagor. Besvara kompletteringspunkterna och beskriv i avsett fält för att förtydliga vilka ändringar som gjorts. När kompletteringarna är genomförda ska ansökan signeras och skickas in på nytt.

Etikprövningsmyndigheten
2022-02920-01-303880
2022-09-03

På Etikprövningsmyndighetens vägnar

Johan Rosén
Ordförande

Beslutet har fattats av följande personer:

Ordförande
Johan Rosén

Ledamöter med vetenskaplig kompetens
Lena Almqvist (psykologi, barn- och ungdomsforskning, vetenskaplig sekreterare)
Önver Cetrez (religions- och kulturpsykologi, vetenskaplig sekreterare)
Oscar Pripp (Etnologi, antropologi, sociologi, föredragande)
Maria Eriksson Baaz (Statsvetenskap)
Minna Gräns (rättsvetenskap)
Maria Harder (Vårdvetenskap)
Niklas Jakobsson (Nationalekonomi)
Mikael Laaksoharju (Människa-datorinteraktion, AI)
Anna Lindström (språk och social interaktion)
Per Sandin (Tillämpad etik, bioetik, miljöetik, forskningsetik)

Ledamöter som företräder allmänna intressen
Håkan Krantz
Pia Milton
Gert Ohlsson
Rigmor Åkesson

Etikprövningsmyndigheten
2022-02920-01-303880
2022-09-03

Beslutet sänds till

Ansvarig forskare: Stig Douglas Filip Sjöwall

Forskningshuvudmannens företrädare: Joakim Lavesson

BESLUT

2022-09-13

Sökande forskningshuvudman
Region Stockholm

Forskare som genomför projektet
Stig Douglas Filip Sjöwall

Projekttitel
Lev-projektet: Kartläggning, bedömning och intervention av hälsorelaterade levnadsvanor hos vuxna med och utan funktionsnedsättning.

Uppgifter om ansökan
Ansökan inkom till Etikprövningsmyndigheten 2022-05-23 och blev valid 2022-06-02. Ansökan är tidigare behandlad vid sammanträde 2022-08-24. Av myndigheten begärd komplettering enligt beslut inkom 2022-09-09.

Etikprövningsmyndigheten beslutar enligt nedan.

BESLUT

Etikprövningsmyndigheten godkänner den forskning som anges i ansökan, med följande villkor:

I all information till forskningspersonerna ska det anges hur länge data sparas.

Det här beslutet kan överklagas hos Överklagandenämnden för etikprövning. Hur man överklagar framgår av bifogad anvisning.

På Etikprövningsmyndighetens vägnar

Johan Rosén
Ordförande

Beslutet har fattats av följande personer:

Ordförande
Johan Rosén

Beslutet har fattats efter föredragning av vetenskaplig sekreterare
Lena Almqvist

Etikprövningsmyndigheten
2022-02920-01-307370
2022-09-13

Beslutet sänds till

Ansvarig forskare: Stig Douglas Filip Sjöwall

Forskningshuvudmannens företrädare: Joakim Lavesson

Etikprövningsmyndigheten
2022-02920-01-307370
2022-09-13

Hur man överklagar Etikprövningsmyndighetens beslut

Vem får överklaga?

Det är forskningshuvudmannen som får överklaga Etikprövningsmyndighetens beslut om det har gått sökanden emot. Överklagandet ska vara skriftligt. Skrivelsen ska vara undertecknad av behörig företrädare för forskningshuvudmannen.

Om forskaren överklagar ska en fullmakt från forskningshuvudmannen bifogas.

När ska beslutet senast överklagas?

Överklagandet ska ha kommit in till Etikprövningsmyndigheten inom tre veckor från den dag då forskningshuvudmannen fick del av beslutet.

Vad ska överklagandet innehålla?

Överklagandet ska innehålla uppgifter om

1. klagandens namn, person- eller organisationsnummer, adress, telefonnummer och e-postadress
2. det beslut som överklagas (dag för beslut, projekttitel och diarienummer)
3. hur ni anser att myndighetens beslut ska ändras och skälen till att beslutet bör ändras.

Var ska överklagandet skickas?

Överklagandet ska ställas till Överklagandenämnden för etikprövning. Men det ska skickas eller lämnas till Etikprövningsmyndigheten.

Om överklagandet har kommit in i rätt tid överlämnar myndigheten överklagandet och handlingarna till Överklagandenämnden för etikprövning.

Etikprövningsmyndigheten
2022-02920-01-307370
2022-09-13
